# Vascular Endothelial Growth Factor B Modulates Cardiac Functions via Ferroptosis Pathways in Post-Myocardial Infarction

**DOI:** 10.3390/cells14201642

**Published:** 2025-10-21

**Authors:** Sai Manasa Varanasi, Ankit Sabharwal, Shreyartha Mukherjee, Huzaifa Muhammad, Riya Kar, Carter Magnano, Anya Dorairaj, Enfeng Wang, Shamit Dutta, Pritam Das, Stephen C. Ekker, Ying Wang, Debabrata Mukhopadhyay, Ramcharan Singh Angom

**Affiliations:** 1 Department of Biochemistry and Molecular Biology, Mayo Clinic College of Medicine and Sciences, Jacksonville, FL 32224, USA; varanasi.saimanasa@mayo.edu (S.M.V.);; 2 Department of Biochemistry and Molecular Biology, Mayo Clinic College of Medicine and Science, Rochester, MN 55905, USA; 3 Department of Pediatrics, Dell Pediatric Research Institute, Dell Medical School, The University of Texas at Austin, Austin, TX 78712, USA; 4 Center for Rare Disease, Dell Medical School, The University of Texas at Austin, Austin, TX 78712, USA; 5 Department of Quantitative Health Sciences, Mayo Clinic, Jacksonville, FL 32224, USA; mukherjee.shreyartha@mayo.edu; 6 College of Medicine, Alfaisal University, Riyadh 11533, Saudi Arabia; 7 Department of Molecular Biosciences, The University of Texas at Austin, Austin, TX 78712, USA; 8 Department of Cardiovascular Medicine, Mayo Clinic, Rochester, MN 55905, USA

**Keywords:** *vascular endothelial growth factor B (VEGFB)*, Neuropilin-1 (*NRP1*), myocardial infarction, hypoxia/ischemia, cardiomyocytes, mitochondrial dysfunction, zebrafish, Ferroptosis

## Abstract

Myocardial infarction (MI) remains a leading cause of mortality worldwide, yet effective cardioprotective strategies remain limited in clinical settings. *Vascular endothelial growth factor B (VEGFB)* has emerged as a promising therapeutic candidate in MI, but the role of its co-receptor, Neuropilin-1 (*NRP1*), in cardiomyocyte (CM) survival under ischemic stress remains poorly understood. Here, we investigated VEGFB-NRP1 signaling using an in vivo zebrafish model of cardiac injury as well as in vitro hypoxia models in CMs. We demonstrated that *VEGFB* overexpression conferred protection against ischemic injury and enhanced cardiac regeneration in the zebrafish heart. Mechanistically, we showed that VEGFB treatment enhances CM viability through reducing reactive oxygen species (ROS), ferroptosis activation, and preserving mitochondrial integrity. We also demonstrated that *NRP1* knockdown in the CMs abolished the VEGFB-mediated protective effects, indicating the significant role of NRP1 signaling in VEGFB-induced cardioprotective effects in MI. Lastly, using transcriptome analysis, we confirmed that VEGFB induces anti-apoptotic and anti-ferroptosis gene programs in CMs in response to hypoxic stress. Collectively, our findings provide mechanistic insight into cell death activation pathways, including ferroptosis, in response to ischemic stress and further validate the therapeutic potential of VEGFB in promoting CM survival in ischemic heart disease.

## 1. Introduction

Cardiovascular diseases (CVDs) remain the leading cause of mortality worldwide, significantly impacting both physical and mental well-being [1,2]. The key risk factors contributing to CVDs include dyslipidemia, smoking, diabetes, and obesity [3], and therapeutic interventions targeting these risk factors have been effective in reducing CVD-related morbidity and mortality. Among CVDs, myocardial infarction (MI) is a severe manifestation of ischemic heart disease (IHD) and results from prolonged ischemia and hypoxia, leading to irreversible myocardial cell death [4,5]. Current clinical interventions for MI primarily focus on revascularization strategies, including percutaneous coronary intervention, thrombolysis, and coronary artery bypass grafting [6,7,8]. However, these approaches do not fully address post-MI cardiac remodeling, mitochondrial dysfunction, and cardiomyocyte death, necessitating the exploration of novel therapeutic strategies.

MI leads to structural cardiac remodeling, including scar tissue formation, cardiomyocyte hypertrophy, and microvascular dysfunction, all of which contribute to progressive heart failure [9]. The underlying pathophysiology of MI is driven by an imbalance in oxygen supply and demand, which triggers a cascade of cellular dysfunction, including oxidative stress, inflammation, and mitochondrial impairment [5]. Recent studies have identified ferroptosis, an iron-dependent form of non-apoptotic cell death, as a major contributor to cardiomyocyte loss in MI [5,10]. Unlike apoptosis or necrosis, ferroptosis is characterized by mitochondrial shrinkage, depletion of glutathione, excessive lipid peroxidation, and accumulation of reactive oxygen species (ROS) [5,11,12]. These events exacerbate mitochondrial dysfunction and impair cardiac function, further worsening MI outcomes [13,14].

*VEGFB*, a key member of the VEGF family, plays a pivotal role in cardiomyocyte survival, vascular homeostasis, and mitochondrial function [15,16,17]. Unlike, *VEGFA* which primarily promotes potent but non-selective angiogenesis, and often is accompanied by adverse effects such as vascular leakage and limited cardiomyocyte specificity [18]. On the other hand, *VEGFB* primarily engages in metabolic and cytoprotective signaling with minimal angiogenic activity, including mitochondrial protection, metabolic regulation, and endothelial cell function [19] and survival [20]. These distinctions underscore the rationale for our focus on VEGFB/NRP1 signaling as a potentially safer and more targeted approach to improving cardiomyocyte survival and function following MI [15]. Although VEGFB’s potential therapeutic role in heart failure remains promising, current evidence is limited to preclinical investigations, and further research is needed to fully understand its mechanisms of action, such that VEGFB-mediated targeted interventions can be developed.

*VEGFB* binds to VEGFR-1 and its co-receptor *NRP1*, leading to activation of downstream tyrosine kinase receptors such as p38 MAPK, ERK/MAPK, AKT, and PI3K [19,21]. *NRP1*, as a co-receptor for *VEGFB*, forms a signaling complex with *VEGFR-1* to activate pro-survival pathways that counteract ferroptosis and oxidative damage [19,21]. Beyond its role in *VEGFB* signaling, *NRP1* interacts with other molecular pathways that regulate cellular stress responses, endothelial barrier integrity, and remodeling of the extracellular matrix [22,23]. Recent evidence suggests that *NRP1* can enhance endothelial cell migration, promote vascular stability, and modulate immune cell infiltration in ischemic tissues, thereby contributing to improved cardiac repair post-MI [24,25]. Furthermore, *NRP1* has been linked to mitochondrial fission and fusion dynamics, suggesting its involvement in preserving mitochondrial integrity during hypoxic stress [23]. A previous study by Jenssen et al. has shown that silencing either *VEGFB* or *NRP1* in zebrafish embryos results in nearly the same lethal phenotype characterized by impaired brain development and vasculature, indicating an important role for *NRP1* in mediating physiological functions of VEGFB [26]. Here, we further examined the molecular signaling pathways of the *VEGFB* and *NRP1* signaling axis in cell survival and mitochondrial function in CMs in response to hypoxia/ischemic injury and validated the VEGFB/NRP1-mediated protective responses against ischemic injury in vivo using an inducible transgenic zebrafish model expressing VEGFB in the heart. Overall, our studies reveal a novel molecular insight; that is, the VEGFB/NRP1 axis plays a protective role in MI, suggesting a new direction for future therapeutics.

## 2. Results

### 2.1. VEGFB Ameliorates the Effect of Hypoxia-Induced Stress in Zebrafish Hearts

**Figure 1:**
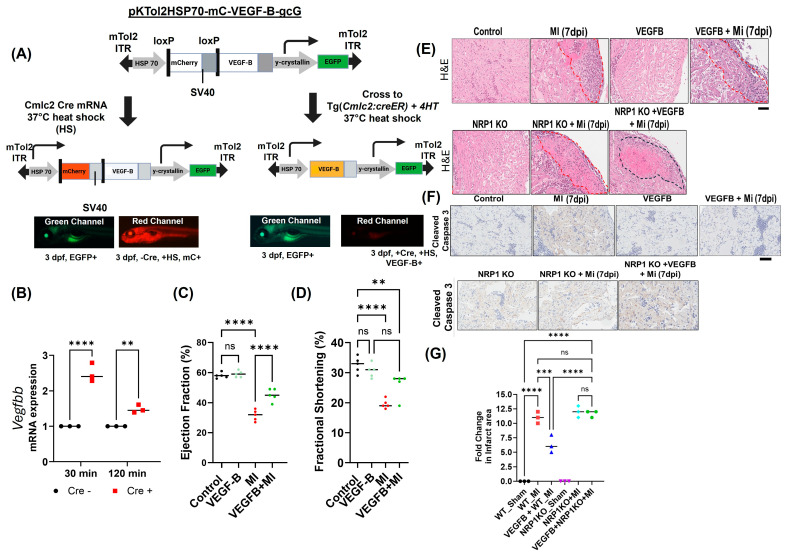
**VEGFB mitigates mitochondrial function and apoptosis in ischemic hearts in zebrafish**. (**A**) The experimental outline shows the schematics of the generation of the heat shock-inducible VEGFB transgenic model (*pKTol2H70-mC-VEGF-B-gcG*) composed of a heat-inducible HSP70 promoter driving a floxed *mCherry* gene and zebrafish *VEGF-B* (*vegf-bb*) and a *γ-crystallin* promoter driving *EGFP*. mTol2 ITR, mini-Tol2 plasmid inverted terminal repeat; BGH(A), bovine growth hormone polyadenylation signal; SV40(A), simian virus 40 polyadenylation signal; rβG(A), rabbit *β-globin* polyadenylation signal. The HSP70 promoter drives transcription of the *mCherry* gene, producing a red fluorescent protein in transgenic zebrafish. The lens-specific *γ-crystallin* promoter drives *EGFP* in the eyes. mC, mCherry; HS, heat shock. Crossing with *Tg(cmlc2:creER)* results in the excision of the floxed *mCherry* gene, and subsequent heat shock induction of the HSP70 promoter produces VEGFB(Vegfbb). Non-crossed transgenic zebrafish were heat-shocked at 37 °C and 3 dpf to monitor the temporal expression of mCherry and VEGFB. (**B**) mRNA expression of VEGFB over time after heat shock. (**C**,**D**) Heart function analysis of zebrafish (ejection fraction and fractional shortening (**D**) after MI injury and *VEGFB* overexpression (*n* = 5)). (**E**) Histological analysis shows an H&E-stained heart section. (**F**) *VEGFB*-mediated enhancement of apoptotic heart as indicated by cleaved caspase-stained heart section after MI induction and *VEGFB* overexpression. (**G**) Quantification showing fold change in the infarcted area. The analysis was performed 7 days post-MI induction. *n* = 3 different sections were analyzed per group. Scatter plots display individual data points from independent experiments. Horizontal bars represent the mean ± SD from at least three independent replicates **, *p* < 0.01. ***, *p* < 0.001. ****, *p* < 0.0001. ns: not significant. Colors indicate different experimental groups: black: control, red: treatment (**B**). black: control, light green: *VEGFB* treatment, red: MI, dark green: VEGFB plus MI (**C**,**D**). black: control, light green, red: MI, blue: VEGFB plus MI, purple: *NRP1* KO, blue: *NRP1* KO plus MI, dark green: VEGFB plus MI in *NRP1* KO (**G**). Different shapes represent groups, and each point represents a replicate. Dashed sections in (**E**) represent the infarcted area.

Their transparent nature and ability to regenerate cardiac tissue after injury, along with the capabilities of live imaging of cardiac function and vascular remodeling, make zebrafish an ideal model system for studying MI and cardiac disease [27,28,29,30]. To further investigate the therapeutic potential of VEGFB in cardiomyocyte survival and cardiac functions under hypoxic stress, we developed a novel inducible transgenic zebrafish model with inducible overexpression of *VEGFB* (*Vegfbb*) under the control of a heat shock promoter, *Tg(pKTol2H70-LP2mc-zfvegfbb-gcG)*, as described in a previous study by our lab [18] (Figure 1A). We then crossed the *Tg(pKTol2H70-LP2mc-zfvegfbb-gcG)* zebrafish line with *Tg(Cmlc2:CreER)* (both of which have a cardiac-specific promoter, *Myosin Light Chain 7 (myl7)*/*Cardiac Myosin Light Chain 2 (cmlc2)* [31]), to generate double-transgenic fish, in which VEGFB is exclusively expressed in the zebrafish heart in an inducible manner. To confirm the expression of *VEGFB*, 6–8-month-old adult zebrafish were exposed to a heat shock at 37 °C for 30 min and 120 min, followed by tissue analysis. *VEGFB* (*Vegfbb*) mRNA expression in the heart tissue isolated from the control (-Cre) and the *VEGFB*-induced (+Cre) samples indicated strong VEGFB expression in the heart tissues of the +Cre samples after both 30 min and 120 min heat shock exposures (Figure 1B). We observed a time-dependent increase in *VEGFB* mRNA with high expression at 30 min and reduced expression at 120 min. This is likely due to transient mRNA turnover after the initial transcriptional surge, a phenomenon commonly observed for rapidly induced genes. Such dynamics are consistent with transient promoter activation and HIF-1α-mediated transcription, where mRNA is rapidly synthesized and subsequently degraded to allow for tight temporal control of gene expression [18]. We then used ultrasound analysis to assess heart function by measuring ejection fraction (EF) and fractional shortening (FS) from *n* = 8 adult zebrafish, as described before [32], in the different groups following MI. The analyses were performed in a blind manner, where the group information was not available to the person performing the analysis. Both the control zebrafish and sham *VEGFB*-overexpressing zebrafish exhibited a normal ejection fraction (Figure 1C) and fractional shortening (Figure 1D), indicating healthy heart function. In contrast, MI in control (-Cre) fish showed significantly reduced EF and FS compared to zebrafish without MI (sham) (50% reduction) (Figure 1C,D), consistent with impaired cardiac function due to myocardial injury. However, in the transgenic *VEGFB*-overexpressing zebrafish plus MI group, the ejection fraction was significantly improved after MI at 3 days post-MI compared to the control (-Cre) group, indicating the protective effect of VEGFB on cardiac function (Figure 1C,D). We then perform histological analysis using hematoxylin and eosin (H&E) staining and cleaved caspase-3 immunostaining to further analyze the beneficial effects of *VEGFB* overexpression in zebrafish heart tissue sections. In the control group, H&E staining showed normal heart architecture with well-defined myocardial layers and no evidence of tissue damage (Figure 1E). In contrast, the MI group exhibited significant structural damage, including disorganized tissue structure and extensive areas of necrosis and fibrosis (Figure 1E). Cleaved caspase-3 staining in MI hearts also revealed increased immunoreactivity in the cardiomyocytes, indicating widespread apoptosis (Figure 1F). We then examined the effects of the transgenic *VEGFB*-overexpressing zebrafish post-MI. H&E staining showed partial restoration of myocardial architecture, with less evidence of fibrosis and necrosis compared to the control zebrafish MI group (Figure 1E,G). Cleaved caspase-3 staining was also markedly reduced, and the number of apoptotic cells in the myocardial tissue was also reduced in the transgenic *VEGFB*-overexpressing zebrafish hearts after MI, compared to the control zebrafish MI group (Figure 1F). These results demonstrate that *VEGFB* overexpression in our novel zebrafish models effectively attenuated MI-induced cardiomyocyte apoptosis, necrosis, and fibrosis and, importantly, promoted tissue preservation. Next, to examine the role of NRP1 signaling in these VEGFB-mediated protective effects in the cardiomyocytes, we crossed a previously characterized *NRP1* KO zebrafish line (*Tg(hsp70;nrp1b;cmlc2-cas9-EGFP* [32]) with the inducible *VEGFB*-overexpression line *(Tg.HS70:VEGFB-EGFP;Tg(Cmlc2:CreER*);*Tg.H70:VEGFB-EGFP*;*Tg(Cmlc2:CreER)*) to generate a triple-transgenic fish, in which VEGFB is overexpressed in the heart and heat shock induces *nrp1b* knockout specifically in cardiomyocytes. Using this triple-transgenic zebrafish model, we show that *nrp1b* ablation abolishes the cardioprotective effects of VEGFB, as measured by ejection and fractional shortening, indicating impaired diastolic function in the *nrp1* KO zebrafish heart (Figure 1C,D). Histological analysis showed a larger necrotic area post-injury in *nrp1b*-deficient hearts compared to VEGFB-overexpressing hearts with intact *nrp1b* (Figure 1E,G), further confirming that NRP1 is essential for VEGFB-mediated protection in this model. These results collectively demonstrate VEGFB signaling via Nrp1, and its overexpression mitigates structural damage, reduces apoptosis, and improves cardiac function in our novel transgenic zebrafish model overexpressing VEGFB after MI.

### 2.2. VEGFB Promotes Cardiomyocyte Survival by Enhancing Proliferation in Response to Hypoxic Stress via NRP1

**Figure 2:**
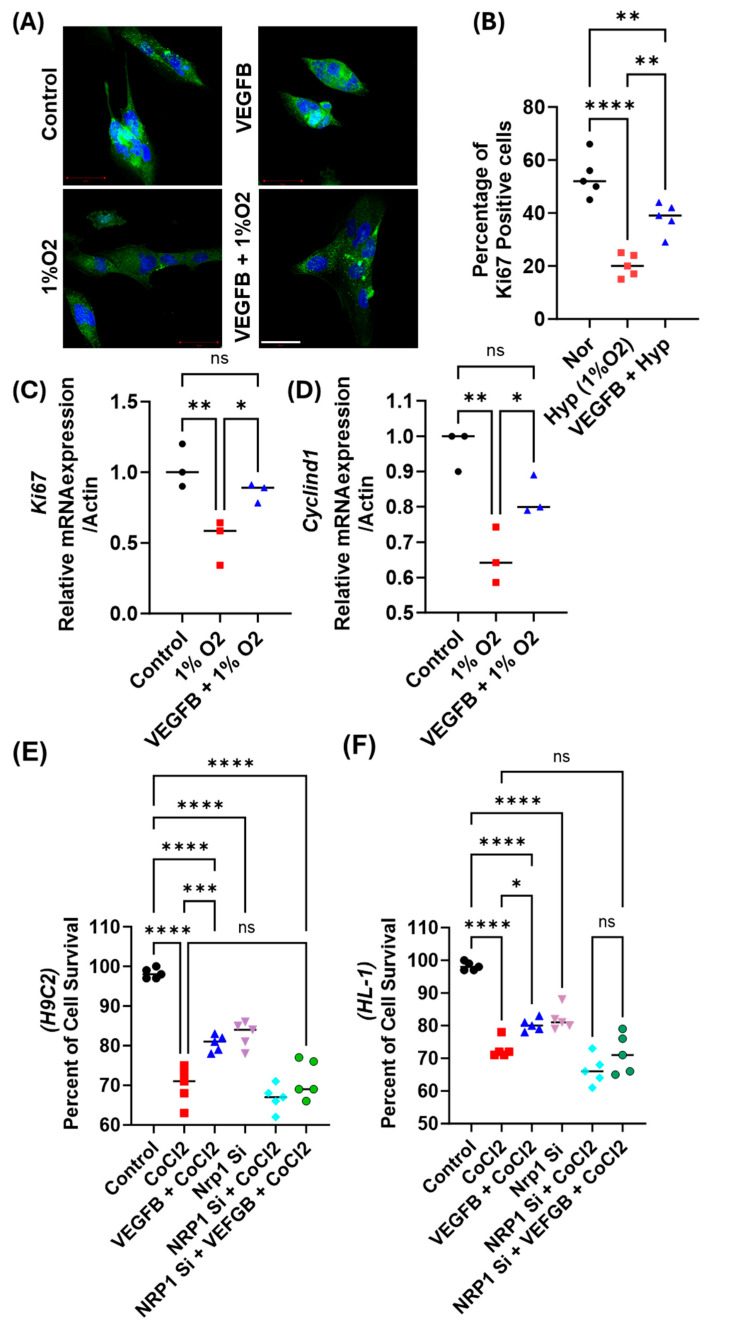
**VEGFB promotes cardiomyocyte survival by enhancing proliferation**. (**A**) Immunofluorescence images showing Ki67 staining in cardiomyocytes (CMs) treated under normoxic (21% O_2_) and hypoxic (1% O_2_) conditions. (**B**) Quantification of Ki67-positive cells. (**C**) mRNA expression analysis of *Ki67* in H9C2 cardiomyocytes. (**D**) mRNA expression analysis of *cyclin D1* in H9C2 cardiomyocytes. (**E**) MTS assay showing VEGFB-mediated prevention of H9C2 CM death. (**F**) MTS assay showing VEGFB-mediated prevention of HL-1 CM death. Scatter plots display individual data points from independent experiments. Statistical significance: * *p* < 0.05, ** *p* < 0.01, *** *p* < 0.001, and **** *p* < 0.0001, ns: not significant. Scale bar = 100 µm. Colors indicate different experimental groups. Different shapes represent groups, and each point represents the replicate.

We then performed a series of experiments to investigate mechanisms of cell death activation in the CMs under hypoxic stress and the VEGFB-mediated protective effects. To do this, we first analyzed the effect of VEGFB treatment on cell proliferation, viability, and mitochondrial functions in response to hypoxic stress conditions. To do this, we treated both H9c2 and HL-1 cells with hypoxia (1%O_2_) for 72 h to mimic ischemic injury. Normoxia-treated cells were used as controls. Ki67 immunostaining revealed that 72 h hypoxia treatment significantly reduced Ki67 expression in H9c2 cells (~50% reduction compared to normoxic controls) (Figure 2A,B). Similar results were observed for the HL-1 cells treated with hypoxia for 72 h (for this experiment, we used 200 μM CoCl_2_ to mimic hypoxia conditions, as previously described [33], which also showed reduced Ki67 expression (~35%, Supplementary Figure S1A,B). RT-qPCR analysis confirmed a decrease in *Ki67* and *cyclin d1* mRNA levels (~50% for *ki67* and 30–40% for *cyclind1* in H9c2 cells treated with hypoxia (1%O_2_) for 24 h (Figure 2C,D) and in HL-1 cells treated with 200 μM CoCl_2_ for 24 h (Supplementary Figure S1C,D) compared to the normoxic control. We then examined the effect of VEGFB treatment on hypoxia-induced Ki67 expression in both the H9c2 and HL-1 cells by treating these cells with recombinant VEGFB (20 ng/mL) and hypoxia (1%O_2_) for 72 h. VEGFB treatment increased Ki67 staining in the H9c2 cells treated with hypoxia compared to those treated with hypoxia alone (Figure 2A,B). We also observed an increased mRNA expression of *Ki67* and *cyclin D1* in the cells treated with VEGFB and hypoxia compared to the hypoxia treatment alone (Figure 2C,D). Similar results of increased *Ki67* expression were observed in the hypoxic HL-1 cells treated with VEGFB (Supplementary Figure S1A–D). We then performed MTS assays to assess cell survival in both H9c2 and HL-1 cells treated with hypoxia (CoCl_2_), VEGFB, and a combination of VEGFB and hypoxia. Previous studies have shown that CoCl_2_ treatment leads to increased generation of reactive oxygen species (ROS) and lipid peroxidation, along with depletion of antioxidant defenses such as glutathione and catalase [34]. Moreover, CoCl_2_ exposure upregulates pro-apoptotic genes including *Bax*, *p53*, *caspase-9*, and *caspase-3*, while suppressing the anti-apoptotic gene *Bcl-2*, thereby recapitulating the molecular and mitochondrial stress responses characteristic of true hypoxia [33,35]. Thus, CoCl_2_ treatment provides a well-established and reproducible in vitro model for studying ischemia- or hypoxia-induced injury mechanisms [36,37,38]. In all subsequent experiments, we used 200 µM CoCl_2_ to mimic hypoxia. CoCl_2_ treatment for 72 h resulted in a significant decrease in cell viability (~40% reduction compared to normoxia, Figure 2E,F), whereas VEGFB treatment in the hypoxic CMs showed partial recovery in cell viability both in H9c2 cells (Figure 2E) and HL-1 cells (Figure 2F), indicating that VEGFB treatment partially reduced hypoxia-induced cell death in the CMs.

To confirm the role of NRP1 in VEGFB-mediated protection, we then performed NRP1 knockdown in both H9c2 and HL-1 cells by using *NRP1* siRNA (Hs_NRP1_8 FlexiTube siRNA) [39] treatment (for 24 h) before treating the CMs with either hypoxia (CoCl_2_) alone or combined with VEGFB. Interestingly, *NRP1* knockdown by itself reduced cell viability (~10% reduction compared to untreated controls, Figure 2E,F) but, importantly, we show that *NRP1* knockdown significantly diminished the pro-survival effects of VEGFB, with cell viability in *NRP1*-knockdown hypoxic H9c2 cells treated with VEGFB remaining unaffected (~68% vs. ~70% in hypoxia-only-treated cells, Figure 2E; similar results were also seen in the HL-1 cells, Figure 2F). To further validate the specificity of *NRP1* depletion, we adopted shRNA-mediated *NRP1* knockdown as described before [40] and analyzed cell viability following hypoxia treatment. We observed similar results on cell viability using MTS as compared to our siRNA-based *NRP1*-knockdown studies in the H9c2 and HL-1 cells (Figure S2A,B).

### 2.3. VEGFB Activates Pro-Survival Signaling Pathways Under Hypoxic Stress via NRP1

**Figure 3:**
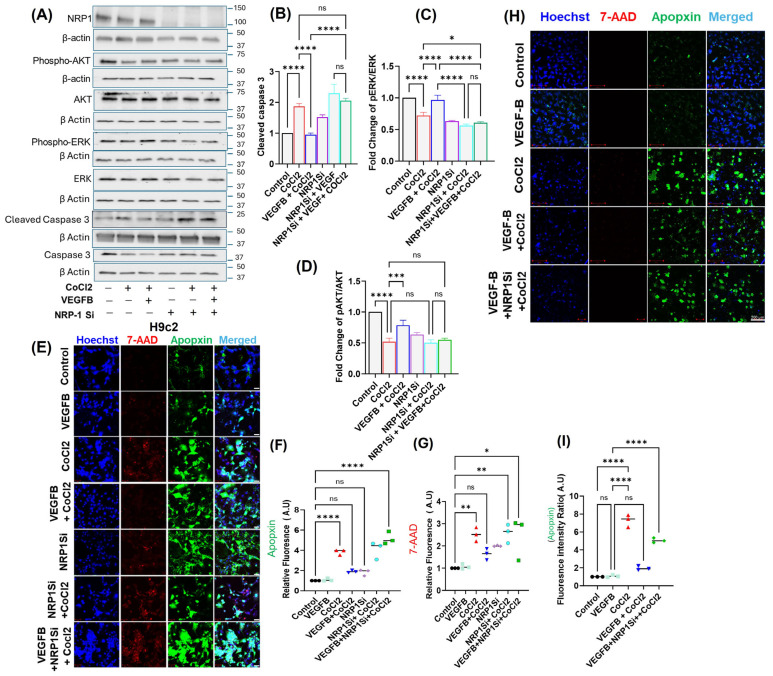
**VEGF-B activates pro-survival signaling pathways under hypoxic stress via NRP1**. (**A**) Western blot analysis showing protein expression of cell survival and apoptotic pathway proteins in lysates from H9C2 cardiomyocytes (CMs) treated with various experimental substances. (**B**–**D**) Quantification of the protein levels shown in (**A**). (**E**) Representative immunofluorescence images of H9C2 cardiomyocytes (CMs) stained with Annexin V in untreated/control H9C2 cells, H9C2 cells treated with VEGF-B, H9C2 cells exposed to hypoxia through treatment with 200 µM CoCl_2_ for 72 h, H9C2 cells treated with VEGF-B plus CoCl_2_, H9C2 cells with *NRP1* siRNA-mediated knockdown, H9C2 CMs with *NRP1* knockdown plus CoCl_2_, and H9C2 CMs with *NRP1* knockdown plus CoCl_2_ and VEGF-B treatment. (**F**,**G**) Quantification of Apopxin and 7-AAD. (**H**) Representative immunofluorescence images of HL-1 cardiomyocytes (CMs) stained with Annexin V in untreated/Control, HL-1 cells treated with VEGF-B, H9C2 cells exposed to hypoxia by treatment with 200 µM CoCl_2_ for 72 h, HL-1 cells treated with VEGF-B plus CoCl_2_, HL-1 cells with *NRP1* siRNA-mediated knockdown plus CoCl_2_, and VEGF-B treatment. (**I**) Quantification of Apopxin and 7-AAD in (**H**). Each experiment was repeated at least three times. Each bar represents the average of three independent samples ± SD. Scatter plots display individual data points from independent experiments. Statistical significance is indicated as * *p* < 0.05, ** *p* < 0.01, *** *p* < 0.001, and **** *p* < 0.00001, ns: not significant. The images were captured using an LSM 800 confocal microscope with 488 nm and 594 nm lasers. Scale bar = 100 µm. Colors indicate different experimental groups. Different shapes represent groups, and each point represents the replicate.

We next examined the protective effects of VEGFB on cell survival signaling as well as the activation of cell death pathways in both H9c2 cells and HL-1 cells treated with hypoxia (200 μM CoCl_2_) and VEGFB. We first assessed protein levels of AKT, phosphorylated AKT (pAKT), ERK, and phosphorylated ERK (pERK), all of which play critical roles in cell survival signaling [41]. Upon hypoxia treatment, there was a significant decrease in the expression of pAKT and pERK protein levels in whole cell lysates of H9c2 cells (as shown by quantification of Western blots in pAKT/AKT and pERK/ERK ratios (Figure 3A–C)), suggesting significant downregulation of cell survival signaling in response to hypoxia. We then measured protein levels of caspase-3 and cleaved caspase-3 to determine the effect of hypoxia on the activation of cell death pathways [42]. We observed significant upregulation of cleaved caspase-3 protein levels in the hypoxia-treated cells (Figure 3A,D), again indicating hypoxia-induced activation of apoptotic cell death pathways. Similar results were observed in HL-1 cells, which showed a significant increase in cleaved caspase-3 levels (Supplementary Figure S2C,D).

We then examined the effect of VEGFB treatment on cell survival and cell death signaling pathways in the CMs under hypoxia stress. Treatment with VEGFB under hypoxia conditions in H9c2 cells increased pAKT and pERK protein level expression, suggesting activation of cell survival signaling pathways in the VEGFB-treated cells (Figure 3A–C). And cleaved caspase-3 protein levels were significantly reduced in both the H9c2 cells and HL-1 cells treated with VEGFB, suggesting that VEGFB protected the CMs from hypoxia-induced cell death (Figure 3A,D and Figure S3). We then induced *NRP1* KO in the CMs by treating the cells with *NRP1* siRNA for 24 h. In *NRP1*-knockdown cells treated with VEGFB and hypoxia, the pAKT and pERK levels remained unchanged when compared to hypoxia-only-treated cells, confirming that the protective effects of VEGFB were lost when *NRP1* was knocked down (Figure 3A–C). Interestingly, cleaved caspase-3 expressions were increased in all groups of cells treated with *NRP1* siRNA, indicating that *NRP1* knockdown by itself activates pro-apoptotic pathways. And importantly, VEGFB treatment in *NRP1* knockout cells did not prevent cleaved caspase activation after hypoxia treatment, again suggesting that *NRP1* knockdown abrogates the anti-apoptotic effects of VEGFB in H9c2 cells (Figure 3A–D). Collectively, these results demonstrate that VEGFB treatment activates survival signaling through AKT and ERK and prevents apoptosis via NRP1 signaling pathways.

We then further investigated the activation of apoptosis pathways in the CMs upon hypoxia stress using additional methods as follows: following treatments, cells were stained with Hoechst 33342 (blue, nuclear stain), Apopxin Green Indicator (early apoptosis marker), and 7-AAD (red, necrosis/late apoptosis marker) and then analyzed by fluorescence microscopy (Figure 3E). The control groups showed minimal Apopxin-positive cells, and strong Hoechst staining was observed with minimal Apopxin Green or 7-AAD fluorescence, indicating a predominantly viable cell population and a low baseline level of apoptosis or necrosis (Figure 3E). In contrast, the hypoxia (CoCl_2_)-treated group showed a significant increase in Apopxin-positive and 7-AAD-positive cells (Figure 3E–G), indicating elevated apoptosis or necrosis under hypoxic stress. This increase was further exacerbated in the *NRP1*-knockdown H9c2 group subjected to hypoxia (Figure 3E–G), where Apopxin staining showed markedly higher apoptotic cell counts. Interestingly, in the *NRP1*-knockdown cells treated with both hypoxia and VEGFB, Apopxin positivity remained unchanged, with levels comparable to those of NRP1-knockdown cells treated with hypoxia alone (Figure 3E,G). Similar results were obtained in HL-1 cells (Figure 3H,I). These results again confirm that VEGFB’s protective effect against apoptosis is compromised in the absence of NRP1, reinforcing the essential role of VEGFB/NRP1-mediated signaling in enhancing cell survival.

### 2.4. VEGFB Suppresses Ferroptosis in Hypoxic H9c2 Cardiomyocytes

**Figure 4:**
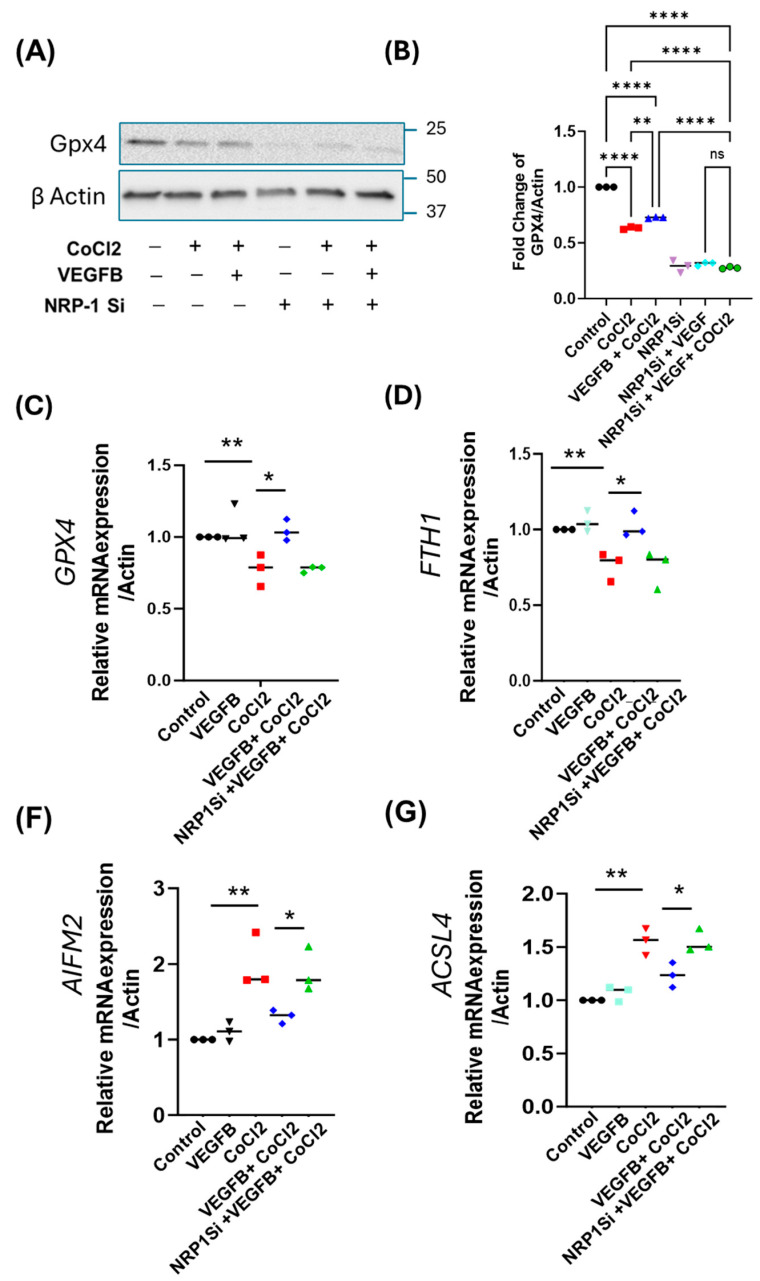
**Ferroptosis pathway activation in hypoxia-induced cardiomyocyte death and its modulation by VEGF-B and NRP1**. (**A**) Western blot analysis showing the expression of apoptosis markers and autophagy proteins in H9C2 CMs treated with CoCl_2_ and *NRP1*siRNA with and without VEGFB. (**B**,**C**) Quantification of the Western blot analysis shown in (**A**), displaying fold changes in protein expression of the indicated markers. (**D**–**G**) RT PCR analysis of ferroptosis marker genes. Scatter plots display individual data points from independent experiments. Statistical significance is indicated as * *p* < 0.05, and ** *p* < 0.01, **** *p* < 0.001. ns: not significant. Experiments in each group were repeated at least three times. Colors indicate different experimental groups. Different shapes represent groups, and each point represents the replicates.

Recent studies have identified ferroptosis, a regulated form of cell death characterized by iron-dependent lipid peroxidation, as a major contributor to cardiomyocyte loss in MI [43,44]. To investigate the role of ferroptosis in hypoxia-induced cardiomyocyte death and proliferation, we first measured levels of GPX4 (glutathione peroxidase 4), a mitochondrial antioxidant enzyme that prevents lipid peroxidation and serves as a central suppressor of ferroptosis in the protein lysates isolated from H9c2 cardiomyocytes. As shown in Figure 4A,B, we observed decreased expression of GPX4 under hypoxia conditions, suggesting that ferroptosis activation may contribute to hypoxia-induced cardiomyocyte injury. In the H9c2 CMs treated with VEGFB under hypoxic conditions, we observed a significant increase in GPX4 expression compared to the control group (Figure 4A,B). We next analyzed the effects of *NRP1* knockdown on GPX4 protein levels in the CMs. Interestingly, we observed that in *NRP1*-depleted cells, the protein levels of GPX4 decreased (Figure 4A,B), and VEGFB treatment did not alter the protein levels of GPX4 in the *Nrp1* KO background, again confirming the essential role of VEGFB-/NRP1-mediated signaling in modulating GPX4 protein levels.

We then further analyzed ferroptosis activation by measuring the mRNA expression of several key ferroptosis-related genes in H9c2 cardiomyocytes exposed to hypoxia (CoCl_2_) and VEGFB. qRT-PCR analysis revealed that the expression of *GPX4* and *FTH1*, both inhibitors of ferroptosis [45,46], was significantly downregulated under hypoxic conditions (Figure 4D,E). On the other hand, hypoxia markedly upregulated the expression of other ferroptosis markers, including *Apoptosis-Inducing Factor Mitochondria-Associated 2 (AIFM2)* [47] (Figure 4F) and *Acyl-CoA Synthetase Long-Chain Family Member 4 (ACSL4*) [48] (Figure 4G), indicating the activation of ferroptosis-dependent cell death pathways in the hypoxic CMs. And importantly, we show that treatment with VEGFB in the hypoxic CMs partially suppressed ferroptosis activation, as evidenced by decreased expression of *ACSL4* and *AIFM2* and increased levels of *GPX4* and *FTH1* compared to the hypoxia-alone control group (Figure 4D–G). And the knockdown of *NRP1* in VEGFB-treated hypoxic cells abolished these protective effects (Figure 4G), again indicating the significant role of VEGFB-NRP1-mediated signaling in ferroptosis prevention in CMs.

### 2.5. VEGFB-NRP1 Signaling Mitigates Mitochondrial Dysfunction and Oxidative Stress Under Hypoxia

**Figure 5:**
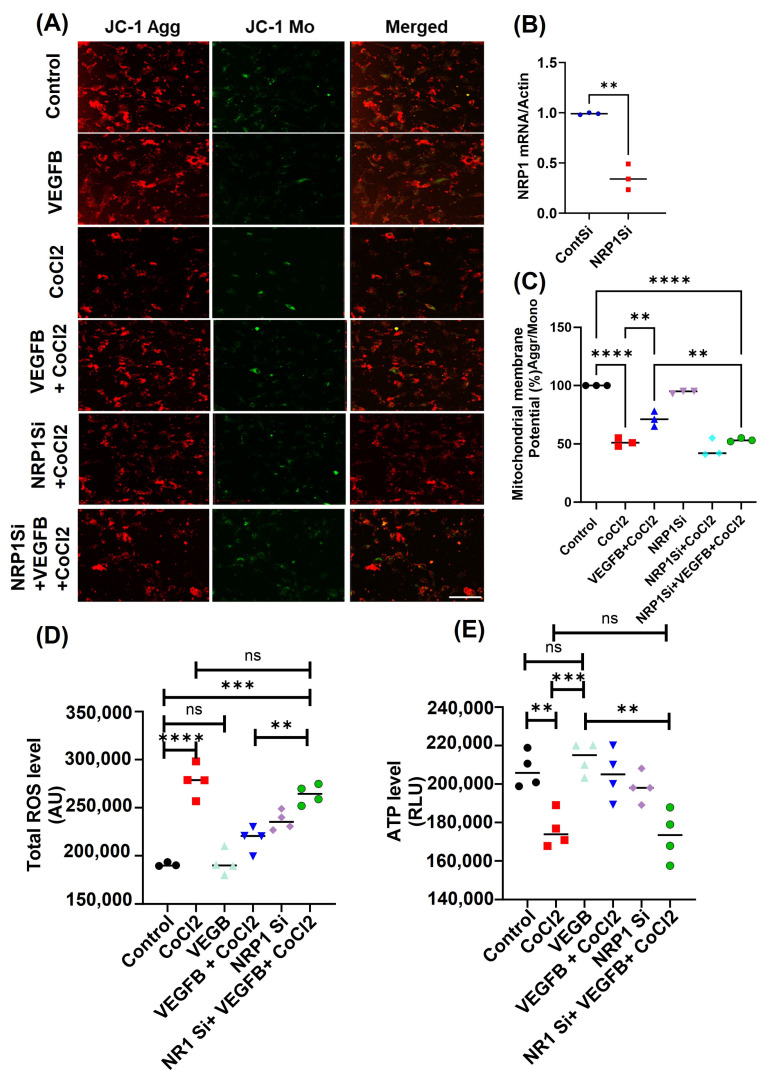
**VEGF-B mitigates the mitochondrial function in ischemic CMs through NRP1**. (**A**) Representative confocal image showing H9c2 CMs stained with JC-1 dye after different exposures. (**B**) mRNA expression showing *NRP1* knockdown in the CMs. (**C**) Quantification of the mitochondrial membrane potential based on JC-1 staining (ratio of aggregate/monomer). (**D**) Total ROS and (**E**) ATP levels were measured in the CMs after the treatments. Scatter plots display individual data points from independent experiments. Horizontal bars represent the mean ± SD from at least three independent replicates. ** *p* < 0.01, *** *p* < 0.001, **** *p* < 0.0001. ns: not significant Scale bar = 100 μm. Colors indicate different experimental groups. Different shapes represent groups, and each point represents the replicates.

We next examined ROS accumulation and the effect of hypoxia on mitochondrial functions in the CMs. To assess mitochondrial function in H9c2 and HL-1 cells following hypoxia and VEGFB treatment, we employed JC-1 staining to evaluate mitochondrial membrane potential (ΔΨm), where red fluorescence indicates JC-1 aggregates in healthy mitochondria and green fluorescence indicates monomeric JC-1 in depolarized mitochondria. In control cells, robust red fluorescence was observed, indicating intact ΔΨm, with minimal green fluorescence (Figure 5A). Knockdown of *NRP1* was confirmed by RT-PCR analysis (Figure 5B). VEGFB treatment alone did not alter red fluorescence intensity, suggesting no significant effect on mitochondrial membrane potential under normoxic conditions (Figure 5A). In contrast, hypoxia treatment led to a marked reduction in red fluorescence, indicative of mitochondrial depolarization and ΔΨm loss (Figure 5A,C). Notably, VEGFB co-treatment under hypoxic conditions partially restored red fluorescence, suggesting a protective effect on mitochondrial membrane integrity (Figure 5A,C). We then examined the effects of *NRP1* knockdown on mitochondrial membrane potential in H9c2 cells. siRNA-mediated *NRP1* depletion alone reduced red fluorescence, indicating impaired ΔΨm (Figure 5A). And importantly, VEGFB failed to restore JC-1 red fluorescence in the *NRP1*-silenced cells under hypoxia, confirming that the protective effects of VEGFB on mitochondrial membrane potential are NRP1-dependent (Figure 5A,C). To further explore the impact of VEGFB and NRP1 on mitochondrial function, we also measured mitochondrial ROS levels using the DCFH-DA fluorescence probe. Control H9c2 cells exhibited low DCF fluorescence, consistent with minimal ROS production (Figure 5D). Hypoxia significantly increased DCF fluorescence, indicating elevated mitochondrial ROS levels (Figure 5D), whereas VEGFB treatment in the hypoxic CMs reduced DCF fluorescence (Figure 5D), suggesting that VEGFB attenuated hypoxia-induced oxidative stress ROS levels. And again, this effect was abolished in *NRP1*-knockdown cells, where VEGFB failed to reduce mitochondrial ROS levels in the hypoxia-treated CMs compared to the non-treated hypoxia-alone group (Figure 5D), again suggesting the important role of NRP1 in VEGFB-mediated signaling. In addition, we also measured total intracellular ROS levels using the CellROX Green reagent. Hypoxia treatment led to a marked increase in CellROX fluorescence intensity compared to the control (Supplementary Figure S4A,B), with quantitative analysis confirming a significant increase in mean fluorescence intensity (Supplementary Figure S4B). We further confirmed *NRP1* specificity by using shRNA-mediated knockdown of *NRP1* as an alternative method, as shown in Figure S3C,D. Consistent with earlier observations, VEGFB failed to reduce ROS levels in ShRNA-mediated *NRP1*-deficient cells, again reinforcing the importance of NRP1 in mediating VEGFB’s antioxidant effects.

### 2.6. Transcriptomic Profiling Reveals That VEGFB Attenuates Hypoxia-Induced Ferroptosis and Apoptotic Pathways in H9c2 Cells

**Figure 6:**
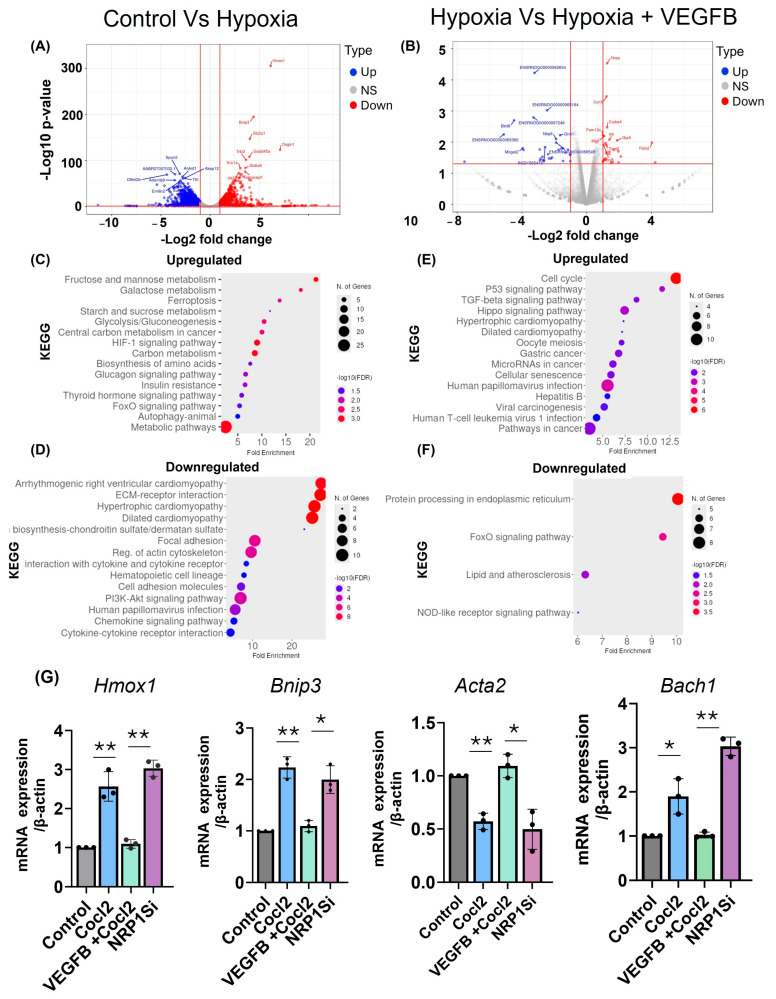
**VEGFB modulates hypoxia-induced transcriptomic changes in H9c2 cardiomyocytes**. (**A**) Volcano plot showing differentially expressed genes (DEGs) between hypoxia-treated and normoxic H9c2 cells (adjusted *p* < 0.05, |log2FC| > 1), highlighting significant upregulation of stress- and apoptosis-related genes such as Hif1a, Vegfa, Bnip3, and Pdk1. (**B**) Volcano plot of DEGs between VEGFB-treated hypoxic cells and hypoxia-only controls, showing downregulation of pro-apoptotic genes (*Casp3, Ddit3, Nos2*) and upregulation of cytoprotective genes (*Nfe2l2, Akt1, Sod2*). (**C**–**E**) KEGG pathway analysis highlighting activation of ferroptosis, HIF-1 signaling, and apoptosis under hypoxia (**D,F**) and enrichment of FoxO signaling and antioxidant pathways with VEGFB treatment (**E**). (**G**) Validation of selected DEGs by qRT-PCR confirmed consistency with RNA-seq data. Scatter plots display individual data points from independent experiments. Horizontal bars represent the mean ± SD from at least three independent replicates. * *p* < 0.05, ** *p* < 0.01.

To investigate the transcriptional profile of VEGFB treatment on cardiomyocytes under hypoxic stress, we performed bulk RNA sequencing on H9c2 cells treated with hypoxia alone or hypoxia plus VEGFB. Differential expression analysis using DESeq2 revealed substantial transcriptomic changes between normoxic controls and hypoxia-treated cells. Hypoxia induced 3238 differentially expressed genes (DEGs), including 1467 upregulated and 1771 downregulated genes (adjusted *p* < 0.05, |log2 fold change| > 1) (Figure 6A). These changes were characterized by the upregulation of genes involved in cellular stress, metabolic adaptation, and apoptosis, such as *Hif1a, Vegfa*, *Bnip3*, and *Pdk1*. Pre-treatment with VEGFB before hypoxic exposure led to 488 DEGs compared to the hypoxia-only group (Figure 6B). Notably, VEGFB downregulated key pro-apoptotic and pro-inflammatory genes, including *Casp3*, *Ddit3 (CHOP)*, and *Nos2*, while upregulating cytoprotective and antioxidant genes such as *Nfe2l2 (Nrf2)*, *Akt1*, and *Sod2*. These findings suggest that VEGFB counteracts hypoxia-induced damage by modulating stress-response pathways. Functional enrichment analysis using clusterProfiler further confirmed these effects. In the hypoxia vs. normoxia comparison, Gene Ontology (GO) terms related to hypoxic response, oxidative stress, and apoptotic signaling were enriched, and KEGG pathway analysis highlighted the activation of “HIF-1 signaling,” “glycolysis/gluconeogenesis,” “ferroptosis,” and “apoptosis” pathways (Figure 6C,D). In contrast, VEGFB-treated hypoxic cells showed enrichment of GO terms such as “negative regulation of cell death,” “angiogenesis,” and “response to oxidative stress,” along with KEGG pathways including “FoxO signaling” and “cell survival” (Figure 6E,F), supporting a VEGFB-driven cytoprotective transcriptional program.

And lastly, to investigate the impact of VEGFB on cellular energy metabolism under hypoxic stress in the CMs, we also measured ATP levels using the RealTime-Glo™ Extracellular ATP Assay (Promega Corporation, Madison, WI, USA). Control H9c2 cells maintained stable ATP levels, indicating normal mitochondrial function, whereas hypoxia significantly reduced ATP levels (Figure 5E), consistent with impaired energy metabolism. And VEGFB treatment partially restored ATP levels in the hypoxic CMs (Figure 5E) under hypoxic conditions, again indicating the beneficial role of VEGFB in maintaining energy homeostasis. Collectively, our results demonstrate that hypoxia induces significant mitochondrial depolarization, increases ROS production, and impairs ATP generation in the cardiomyocytes and suggest that VEGFB-NRP1 signaling modulates mitochondrial integrity and redox balance to counteract ferroptosis under hypoxic conditions.

Principal component analysis (PCA) revealed distinct clustering among normoxia-, hypoxia-, and VEGFB-treated groups, with VEGFB-treated samples forming a unique cluster that partially overlapped with both the hypoxia and control groups (Supplementary Figure S4), indicating a distinct VEGFB-mediated transcriptomic signature. Differential expressions of selected genes were validated by qRT-PCR, confirming the RNA-seq findings (Figure 6G). Together, these results demonstrate that VEGFB attenuates hypoxia-induced apoptotic and inflammatory gene expression in cardiomyocytes by activating cytoprotective, antioxidant, and pro-survival pathways.

### 2.7. NRP1 and VEGFB Co-Expression Networks Reveal Conserved Pathways and Adaptive Roles in Ischemic Heart Disease

To explore the regulatory roles of NRP1 and VEGFB in myocardial infarction (MI), co-expression and network analyses were conducted across five independent MI-related transcriptomic datasets. Differential expression analysis followed by stringent correlation filtering (R^2^ > 0.8) identified 327 co-expressed genes per dataset. A set of 245 conserved genes emerged, forming the basis for integrative functional enrichment and network modeling. Ingenuity Pathway Analysis (IPA) of the GSE115031 dataset revealed that *NRP1* and *VEGFB* function as central hubs within MI-associated networks. Gene Ontology (GO), Disease Ontology (DO), and KEGG enrichment of the conserved gene set reinforced the relevance of these networks to MI pathophysiology (Supplementary Figures S5 and S6). Key enriched terms included miRNA metabolic processes, extrinsic apoptosis, immune cell migration, and angiogenic signaling, all integral to post-MI remodeling. DO analysis revealed enrichment in myocardial and cerebral ischemia (Figure S5A), while KEGG pathways pointed to shared mechanisms in diabetic cardiomyopathy, myocarditis, and vascular dysfunction (Supplementary Figure S5B).

Further stratification of co-expression networks in controls vs. MI (GSE115031) revealed context-dependent functional reprogramming of *NRP1* and *VEGFB* (Figure S7). In healthy tissue, *NRP1* was associated with transcriptional and neurodevelopmental roles, whereas in MI, it shifted to muscle contraction and relaxation, indicating a role in maintaining cardiac function under stress (Figure S7A,B). *VEGFB*, conversely, transitioned from synaptic signaling to proteostasis regulation, notably inhibiting proteasomal catabolism, indicating cytoprotective remodeling (Figure S7C,D). Together, these findings underscore the adaptive, condition-specific roles of *NRP1* and *VEGFB* in MI and highlight their therapeutic implications in ischemic and cardiometabolic disorders.

### 2.8. Ferroptosis Pathway Activation in Hypoxia-Induced Cardiomyocyte Death and Its Modulation by VEGFB

Recent studies have identified ferroptosis, an iron-dependent form of non-apoptotic cell death, as a major contributor to cardiomyocyte loss in MI [5,10]. To elucidate the molecular impact of hypoxia and the protective role of VEGFB, we performed bulk RNA sequencing on H9c2 cardiomyocytes under three conditions: normoxia, hypoxia, and VEGFB pre-treatment followed by hypoxia. Compared to normoxic controls, hypoxia induced a robust ferroptosis gene signature, characterized by significant upregulation of ferroptosis drivers, including *ACSL4*, *TP53*, *HMOX1*, *IREB2*, *ALOX12*, *KEAP1*, *ATM*, *BACH1*, *SIRT3*, *LONP1*, *DNAJB6*, and *FTH1.* In contrast, the key ferroptosis suppressors *GPX4*, *AIFM2 (FSP1)*, and *FXN1* were markedly downregulated, consistent with increased ferroptosis vulnerability under hypoxic stress (Tables S1 and S2). Remarkably, pre-treatment with VEGFB significantly reversed many of these transcriptomic changes. VEGFB restored the expression of *GPX4*, *AIFM2*, and *FXN1* and suppressed the hypoxia-induced upregulation of *ACSL4*, *ALOX12*, *IREB2*, and stress regulators, including *TP53*, *KEAP1*, *SIRT3*, and *ATM*. This transcriptional reversal demonstrates the cytoprotective effect of VEGFB, likely through suppression of oxidative stress, iron dysregulation, and mitochondrial dysfunction.

### 2.9. Ferroptosis Pathway Mapping Highlights VEGFB-Driven Cytoprotective Reprogramming Under Hypoxia

To assess ferroptosis-related gene dynamics (KEGG rno04216) in *Rattus norvegicus*, we used the Pathview R package (4.4.2) for pathway-level visualization, based on RNA-seq data from control, hypoxia-treated, and VEGFB-treated hypoxic cardiomyocytes. Differential expression analysis was performed for control vs. hypoxia and hypoxia vs. VEGFB + hypoxia comparisons. Fold changes were computed from normalized counts, and Ensembl IDs were mapped to Entrez Gene IDs via the org.Rn.eg.db annotation package (Version:3.21); unmapped genes were excluded. Under hypoxia, several ferroptosis-associated genes exhibited pro-ferroptotic expression changes (Supplementary Figure S8A). *ALOX15*, a lipoxygenase driving phospholipid peroxidation, was markedly upregulated, consistent with increased lipid ROS [43]. Conversely, *ACSL4*, which sensitizes membranes to peroxidation, and *GPX4*, a key lipid peroxide-detoxifying enzyme, were downregulated, indicating compromised antioxidant defense [49]. Upregulation of *STEAP3* and *TFRC* shows increased intracellular iron and iron-mediated oxidative stress [44]. Moderate induction of *TP53* and increased *ATG5* expression implicated in ferritinophagy further supported a ferroptosis-prone state under hypoxia [50,51]. VEGFB treatment reversed many of these alterations (Supplementary Figure S8B). *ALOX15*, *STEAP3*, and *TFRC* expression decreased, while *GPX4* was restored, indicating suppression of lipid peroxidation and reinforcement of antioxidant capacity. Upregulation of *ACSL4* indicated a shift toward lipid metabolic rebalancing, and *TP53* downregulation aligned with ferroptosis inhibition. Together, the data reveals that hypoxia promotes ferroptosis via iron accumulation, lipid peroxidation, and impaired antioxidant defense effects that are attenuated by VEGFB, supporting its role in cytoprotection via ferroptosis suppression. Further, we conducted a comparative analysis between our experimental transcriptomic dataset and a previously published hypoxia-induced microarray dataset [52]. A rank-based transformation enabled scale-independent visualization of mitochondrial gene expression, which revealed strong concordance in directional shifts between datasets (Supplementary Figure S9A). For instance, *BNIP3* and *PDK1* ranked among the most significantly upregulated in our transcriptomic dataset, with compelling statistical significance [*p* = 1.93 × 10^−196^, *p* = 1.078 × 10^−24^]. VEGFB treatment reversed the hypoxia-induced upregulation of these genes (Supplementary Figure S9B,C). Recurring evidence implicates both *BNIP3* and *PDK1* in ferroptotic signaling, supported by their transcriptional responsiveness to ferroptotic triggers and involvement in pathways governing redox balance, metabolic flux, and iron homeostasis [51,53,54,55,56]. Similarly, additional ferroptosis genes, including *MAOA*, *BID*, and *CAV1*, were modulated by VEGFB treatment (Supplementary Figure S9E–G).

### 2.10. Correlation Analysis of NRP1 and VEGFB Expression in Human Tissues

To investigate the relationship between *NRP1* and *VEGFB* expression across human tissues, a Pearson correlation analysis was performed using RNA-seq data from the GTEx database. Expression levels (TPM, transcripts per million) of *NRP1* and *VEGFB* were extracted for the normal human tissue types. The analysis revealed significant co-expression patterns between *VEGFB* and *NRP1* and suggested a positive correlation between *NRP1* and *VEGFB* expression across the dataset, as shown in the scatter plot (Supplementary Figure S10A, Pearson correlation coefficient r = 0.64 (*p* < 0.0001)). To assess the disease-contextual relationship between *NRP1* and *VEGFB* expression in the human heart, we performed co-expression analysis using transcriptomic data from the iLINCS portal [57] https://www.ilincs.org/ilincs/ (accessed on 12 June 2025). Co-expression analysis was conducted independently for the ischemic cardiomyopathy (ICM) and nonischemic cardiomyopathy (NICM) groups following data normalization using the platform’s integrated preprocessing pipeline. In both groups, *NRP1* and *VEGFB* transcripts exhibited a positive correlation (Supplementary Figure S10B). However, the strength of this correlation was notably higher in the ICM cohort, implying a potentially enhanced functional relationship under ischemic conditions. Differential correlation analysis confirmed a statistically significant increase in *NRP1–VEGFB* co-expression in ICM compared to NICM myocardial tissue. These findings indicate that ischemic remodeling may involve coordinated upregulation or coregulation of *NRP1* and *VEGFB* expression, rather than direct functional interaction, and further experimentation is required to further validate these findings.

## 3. Discussion

VEGFB, through its receptor eVEGFR1 and co-receptor NRP1, initiates signaling cascades essential for angiogenesis, tissue protection, and metabolic regulation [6,58,59]. Previous studies have demonstrated that VEGFB plays a pivotal role in cardiovascular health, particularly in modulating mitochondrial function and cellular survival under stress conditions such as ischemia [15,16,17,20,60,61]. To confirm and further validate the protective role of VEGFB in mitigating hypoxia-induced myocardial injury, we developed a *VEGFB*-overexpressing transgenic zebrafish model with inducible, cardiomyocyte-specific *VEGFB* expression using an inducible Cre-loxP system, wherein heat shock induction activates *VEGFB* expression, enabling controlled temporal studies. Using a zebrafish MI model, we showed that VEGFB overexpression in the zebrafish heart significantly improved cardiac functions, as evidenced by increased ejection fraction and fractional shortening on ultrasound post-MI. Similarly, using histological analysis, we show that VEGFB overexpression in post-MI zebrafish significantly reduced apoptosis in cardiomyocytes and further revealed reduced myocardial thinning and fibrosis, with improved tissue organization overall. These results highlight and further validate VEGFB signaling via its co-receptor NRP1 as a therapeutic strategy for preserving cardiac structure and function during ischemic injury in vivo. Mechanistically, our findings demonstrate that under hypoxic conditions (using both 1%O_2_ and 200 µM CoCl_2_ to mimic hypoxia), there was enhanced cellular injury in the CMs, as measured by increased ROS production and mitochondrial depolarization and mitochondrial membrane, ΔΨm, loss, leading to the activation of apoptosis pathways in the CMs. Significantly, through all the parameters measured we showed that VEGFB treatment via NRP1 signaling mitigated cellular injury by reducing ROS, increasing ATP production, decreasing apoptosis, and enhancing mitochondrial functions, thus preserving cellular energy homeostasis under hypoxic stress. In addition, multiple studies have shown that both the AKT and ERK pathways are key regulators of cellular survival, growth, and metabolism [62]. A previous study showed that the activation of AKT and ERK signaling by VEGFB is associated with improved cellular resilience to stress, particularly in the context of cardiovascular injury [15]. Our results showed that hypoxia conditions significantly reduced the protein expression levels of both AKT/pAKT and ERK/pERK, whereas VEGFB treatment increased the expression levels of both AKT/pAKT and ERK/pERK, demonstrating the activation of pro-survival cell signaling and anti-apoptotic pathways in the CMs, again highlighting the dual role of VEGFB in both endothelial functions and myocardial cell survival under ischemic conditions. Our data show that *NRP1* knockdown alone leads to reduced cell viability and increased cleaved caspase-3 levels, indicating intrinsic pro-apoptotic effects even in the absence of additional stressors. This observation is biologically consistent with the known multifunctional role of NRP1 as a survival and homeostatic regulator in multiple cell types.

NRP1 is a multifunctional co-receptor that interacts with VEGF family members (particularly VEGF-A, VEGF-B, and PlGF) and other ligands such as semaphorins and TGF-β. Through these interactions, NRP1 modulates several key signaling pathways that promote cell survival and inhibit apoptosis. Notably, NRP1 enhances PI3K-AKT and MAPK/ERK signaling downstream of VEGFR2 or NRP1–integrin complexes, pathways well known for maintaining mitochondrial integrity, reducing ROS generation, and suppressing caspase activation. Therefore, loss of NRP1 disrupts these pro-survival signals, leading to enhanced susceptibility to intrinsic apoptotic mechanisms [26,63,64].

In heart failure (HF), ferroptosis leads to substantial impairments in CMs, making targeted therapies against oxidative stress and ferroptosis an important option in HF research [65]. In this regard, VEGFB’s involvement in lipid metabolism and its regulation of mitochondrial dynamics provide new insights into its therapeutic potential in addressing metabolic dysfunctions, in particular ferroptosis induction in MI, that often accompany cardiovascular diseases [61]. To investigate the role of ferroptosis in hypoxia-induced cardiomyocyte death and proliferation, we measured the levels of multiple markers of ferroptosis, including the ferroptosis inhibitors *GPX4* and *FTH1* and ferroptosis activators *AIFM2* and *ACSL4.* Under hypoxic conditions, we showed that both *GPX4* and *FTH1* levels were reduced, whereas levels of *AIFM2* and *ACSL4* were significantly increased, indicating the activation of ferroptosis. And again, these data confirm that VEGFB treatment via NRP1 also effectively attenuated hypoxia-induced ferroptosis pathways in the cardiomyocytes, demonstrating that VEGFB/NRP1 signaling presents an additional therapeutic avenue as ferroptosis is increasingly recognized as a key contributor to cardiomyocyte loss in HF.

We further confirmed our findings using RNA-seq analysis and showed that hypoxia robustly upregulated genes associated with cellular stress responses, metabolic reprogramming, and apoptosis in H9c2 cardiomyocytes. These included key hypoxia-inducible factors (*Hif1a*, *Vegfa*), metabolic regulators (*Pdk1*), and pro-apoptotic mediators (*Bnip3*), highlighting the activation of classical hypoxic injury pathways.

*BNIP3* (BCL2/adenovirus E1B 19 kDa-interacting protein 3) is a hypoxia-inducible pro-apoptotic protein regulated by HIF-1α that localizes to the mitochondrial outer membrane. It promotes mitochondrial dysfunction by disrupting the membrane potential and inducing a permeability transition [66], leading to cytochrome c release and the activation of intrinsic apoptotic and mitophagy pathways. In cardiomyocytes, sustained *BNIP3* activation under ischemic or hypoxic stress contributes to mitochondrial depolarization, ROS accumulation, and cell death [67]. *PDK1* (Pyruvate Dehydrogenase Kinase 1) is a key metabolic enzyme and transcriptional target of HIF-1α that inhibits the pyruvate dehydrogenase complex, thereby shifting glucose metabolism from oxidative phosphorylation toward glycolysis under low-oxygen conditions. This adaptation reduces mitochondrial oxygen consumption and ROS production, enhancing cell survival during hypoxia. In cardiac tissue, appropriate regulation of *PDK1* is crucial for maintaining metabolic flexibility and minimizing ischemia-induced injury [68]. In contrast, VEGFB pre-treatment prior to hypoxic exposure significantly altered the transcriptional response, resulting in a distinct set of differentially expressed genes (DEGs) when compared to the hypoxia-alone group. Notably, VEGFB suppressed the expression of pro-apoptotic and pro-inflammatory genes such as *Casp3*, *Ddit3 (CHOP)*, and *Nos2* while upregulating genes involved in cytoprotection and redox homeostasis, including *Nfe2l2* (Nrf2), *Akt1*, and *Sod2*. These data demonstrate that VEGFB modulates hypoxia-induced injury by activating survival signaling and antioxidant defense pathways. Further, the VEGFB-mediated regulation of *BNIP3* and *PDK1* under hypoxic stress highlights additional ferroptosis-associated gene targets such as *MAOA*, *BID*, and *CAV1*. VEGFB-induced transcriptional repression of *MAOA* likely contributes to cytoprotection via attenuation of mitochondrial ROS generation, as *MAOA* depletion promotes the accumulation of serotonin (5-HT), a potent radical-trapping antioxidant capable of mitigating lipid peroxidation and thereby counteracting ferroptosis [69,70]. Moreover, VEGFB-mediated downregulation of BID further reinforces cellular resilience against ferroptosis damage. As a BH3-only pro-apoptotic protein, BID interferes with ferroptosis through mitochondrial apoptosis pathways; thus, its repression under hypoxia limits mitochondrial outer-membrane permeabilization, preserving mitochondrial integrity and functionality crucial for cell survival under oxidative conditions [71,72]. Concurrently, reduced expression of *CAV1*, a critical regulator of iron-dependent lipid peroxidation through the modulation of membrane-associated iron transport and redox-sensitive signaling, significantly decreases ferroptosis susceptibility by diminishing intracellular iron accumulation and subsequent oxidative stress cascades [73]. Collectively, this data illuminates a previously unrecognized, sophisticated transcriptional type of reprogramming orchestrated by VEGFB, which mitigates hypoxia-induced ferroptotic damage via multi-nodal modulation of mitochondrial redox balance and integrity. These molecular mechanistic insights into the VEGFB regulatory network highlight its therapeutic potential in mitochondrial dysfunction.

Functional enrichment analysis using Gene Ontology (GO) confirmed these transcriptomic shifts. In the hypoxia vs. normoxia comparison, enriched GO terms included “response to hypoxia,” “cellular response to oxidative stress,” and “regulation of apoptotic signaling,” consistent with a cellular damage and death program. Corresponding KEGG pathway analysis indicated the activation of pathways such as “HIF-1 signaling,” “glycolysis/gluconeogenesis,” “ferroptosis,” and “apoptosis,” further supporting the deleterious effects of sustained hypoxia. In contrast, VEGFB-treated hypoxic cells exhibited enrichment of GO terms such as “negative regulation of cell death,” “angiogenesis,” and “response to oxidative stress,” along with KEGG pathway activation of “FoxO signaling,” “PI3K-Akt signaling,” and “antioxidant defense,” pointing to VEGFB’s role in promoting adaptive, protective gene networks.

These transcriptomic findings align with our functional data demonstrating that VEGFB preserves mitochondrial membrane potential, reduces ROS production, and maintains ATP levels under hypoxic stress, hallmarks of protection against ferroptosis and apoptosis. Moreover, principal component analysis (PCA) showed that VEGFB-treated samples formed a distinct cluster from both normoxic and hypoxic groups, indicating a unique transcriptomic state that reflects VEGFB’s modulatory effects. Taken together, these results highlight that VEGFB not only attenuates the transcriptional programs associated with hypoxia-induced injury but actively reprograms cardiomyocytes toward survival by engaging NRP1-dependent cytoprotective, anti-apoptotic, and antioxidant pathways. This positions VEGFB as a possible therapeutic agent for mitigating hypoxia-driven myocardial damage, in part through transcriptional suppression of ferroptosis and apoptotic signaling. And lastly, by integrating transcriptomic data from GTEx, the Human Protein Atlas, and GEO databases, we also confirmed enriched expression of VEGFB and NRP1 specifically in endothelial cells and cardiomyocytes, underscoring their physiological relevance in maintaining cardiac homeostasis. In summary, our study identifies VEGFB as a critical regulator of mitochondrial function, redox balance, and survival signaling in cardiomyocytes exposed to ischemic stress. Through activation of the AKT and ERK pathways and by attenuating both apoptosis and ferroptosis, VEGFB confers robust cardioprotection. These findings highlight that the VEGFB/NRP1 signaling axis plays an important role in myocardial infarction and heart failure. A previous study in rats demonstrated that the heart-specific VEGFB transgene induced impressive growth of the epicardial coronary vessels and their branches. This leads to increased arterial supply in ischemic heart disease [74]. We propose that VEGFB’s role may be context-dependent, influenced by metabolic state, NRP1/VEGFR1 expression balance, and cell-type specificity, consistent with the protective effects observed here under acute ischemic stress. Importantly, VEGFB is predominantly expressed in cardiomyocytes and endothelial cells, where it acts through VEGFR1 and NRP1 to regulate metabolic homeostasis, mitochondrial function, and stress resistance. In the intact heart, endothelial-derived VEGFB can influence adjacent cardiomyocytes through paracrine signaling, while cardiomyocyte-derived VEGFB may, in turn, modulate endothelial survival and angiogenesis, establishing a bidirectional protective circuit. Additionally, fibroblasts and pericytes can serve as secondary VEGFB sources that reinforce myocardial remodeling responses. Therefore, the cardioprotective effects observed in our zebrafish and in vitro models likely reflect the combined action of both autocrine cardiomyocyte and paracrine endothelial/fibroblast VEGFB–NRP1 signaling. The absence of these paracrine interactions in isolated cardiomyocyte cultures may explain the relatively modest effects observed in vitro compared with in vivo settings. Further studies are warranted to elucidate the detailed molecular mechanisms involved and to evaluate translational strategies aimed at clinical application.

## 4. Conclusions

Our study reveals that VEGFB, through its receptor NRP1, provides critical protection to cardiomyocytes against ischemic stress by preserving mitochondrial function, reducing oxidative damage, and activating pro-survival signaling pathways. Importantly, VEGFB mitigates both apoptosis and ferroptosis, two key modes of cell death implicated in myocardial injury, thereby enhancing cardiomyocyte survival under hypoxic conditions. The enriched expression of VEGFB and NRP1 in cardiomyocytes and endothelial cells further highlights their physiological relevance in cardiac homeostasis. These findings position the VEGFB/NRP1 axis as a compelling therapeutic target to limit ferroptosis and apoptotic damage in myocardial infarction and heart failure, offering promising avenues for future translational research.

Limitations: Our study highlight’s the role of the VEGFB–NRP1 axis in cardiomyocyte protection in response to ischemic injury; however, there are certain limitations in the current study: (1) In this study, zebrafish and cell-based models were employed to investigate the molecular and cellular mechanisms underlying ischemic injury. These models successfully recapitulate several key aspects of myocardial injury, including contractile dysfunction, apoptosis, oxidative stress, and ferroptosis. However, we acknowledge that they do not fully reproduce the complex pathophysiology of human myocardial infarction (MI), which involves additional processes such as ischemia–reperfusion dynamics, immune cell infiltration, and long-term structural remodeling. (2) While we have demonstrated that the protective effects of VEGFB are NRP1-dependent, we have not evaluated the role of the VEGFB co-receptor, VEGFR1, in this paradigm, which we plan to pursue in future follow-up studies. (3) While our gene expression and biochemical data strongly suggest ferroptosis involvement, we acknowledge that direct rescue experiments using ferroptosis inhibitors such as Ferrostatin-1 or Liproxstatin-1 would further strengthen this conclusion.

## 5. Materials and Methods

### 5.1. Sex as a Biological Variable

Sex as a biological variable was not considered in the design of this study. The experiments were conducted using mixed sex, and sex-specific effects were not evaluated.

### 5.2. Cell Cultures

H9c2 embryonic rat heart-derived myoblast cells from ATCC and kindly donated by Dr. Nadine Norton at Mayo Clinic, Jacksonville, were cultured in Dulbecco’s modified Eagle medium (DMEM), supplemented with 10% fetal bovine serum (FBS) under 95% air/5% CO_2_, and subcultured when at 50–60% confluence. HL-1 cells from ATCC and kindly gifted by Dr. Delisa Fairweather and Dr. Norton Nadine were grown in fibronectin–gelatine-coated flasks containing Claycomb medium (Sigma, Burlington, MA, USA) and supplemented with 10% FBS, 100 U/mL penicillin, 100 μg/mL streptomycin, 2 mM L-glutamine, and 0.1 mM norepinephrine. Cells were cultured at 37 °C and 5% CO_2_. Experiments were performed using cells between passages 8 and 12.

### 5.3. Hypoxia Induction

H9c2 and HL-1 cells were subjected to hypoxia (1% O_2_) for different periods of time (3 h, 6 h, 12 h, 24 h, 72 h) in a controlled hypoxic plastic chamber (Modular Incubator Chamber/Hypoxia Chamber (MIC-101), Embrient Inc, San Diego, CA, UAS) and incubated at 37 °C for specific periods of time. Additionally, to mimic hypoxia, we also used 200 µM cobalt chloride (CoCl_2_) and incubated cells at 37 °C for 24 to 72 h as described before [75].

### 5.4. Cell Viability Assay 

The MTS assay was performed to assess cell viability in H9c2 and HL-1 cells following treatment. Cells were seeded on 96-well plates at a density of 5 × 10^4^ cells/well (H9c2) or 8 × 10^4^ cells/well (HL-1) and allowed to adhere for 24 h in DMEM (H9c2) or Claycomb medium (HL-1) supplemented with 10% FBS and 1% penicillin–streptomycin at 37 °C in a 5% CO_2_ incubator. Following treatment, 10 µL of MTS solution (from Promega Inc., Madison, WI, USA) (5 mg/mL) was added to each well, and cells were incubated for 3–4 h to allow for formazan crystal formation. The medium was then carefully removed, and 150 µL of DMSO was added to dissolve the formazan crystals. The absorbance was measured at 570 nm using a microplate reader, with a reference wavelength of 630 nm. Cell viability was calculated as a percentage of control cells using the relevant formula. All experiments were performed in triplicate, and appropriate blank wells containing medium and MTS without cells were included to account for background absorbance.

### 5.5. ROS Response in Response to Hypoxia 

To perform a reactive oxygen species (ROS) assay, the H9c2 and HL-1 cardiomyocytes were first cultured in their appropriate growth medium until they reached 70–80% confluency. Serum starvation was achieved by replacing the culture medium with serum-free medium 2–4 h before the assay to minimize background fluorescence. For ROS detection, the fluorescent dye DCFH-DA (10 µM) (Abcam #ab113851, Cambridge, UK) and CellROX green (5 µM) (Thermo Fisher Scientific^TM^, Waltham, MA, USA) for mitochondrial ROS were prepared in a serum-free medium. For CellROX assay, on the day of the experiment, the cells were washed with warm phosphate-buffered saline (PBS) and incubated with the dye at 37 °C for 30–45 min in the dark. After incubation, the cells were washed twice with PBS to remove excess dye. Different experimental conditions were set up to assess ROS levels. Control cells were maintained in serum-free medium, while positive control cells were treated with 100–500 µM hydrogen peroxide (H_2_O_2_) for 30–60 min to induce reactive oxygen species (ROS) production. Fluorescence was then measured using a fluorescence microscope at Ex/Em 485/535 nm. Intracellular reactive oxygen species (ROS) levels were quantified using the CellROX™ Green Reagent (Thermo Fisher Scientific, Cat# C10444), following the manufacturer’s instructions, with minor modifications to optimize for cardiomyocytes (CM). For the DCFH-DA/H2DCFDA–cellular ROS assay, the reconstituted lyophilized DCFDA in DMSO was diluted in the provided 1× buffer to achieve a final concentration of 10–25 µM. Cells were seeded on a black 96-well plate with a clear bottom at a density of approximately 1 × 10^4^ to 5 × 10^4^ cells per well and incubated overnight to allow for adherence. Once the cells were ready, they were gently washed with PBS or 1× buffer, and 100 µL of the DCFDA working solution was added to each well, followed by incubation at 37 °C for 30 to 45 min in the dark. After incubation, the cells were washed with PBS to remove excess dye and replaced with serum-free media. The cells were then treated with the desired treatment for the desired time. Using a fluorescence plate reader, ROS generation was detected by detecting fluorescence at excitation/emission wavelengths of 485/535 nm. Untreated cells (negative control), TBHP-treated cells (positive control), and blank wells containing media and dye without cells were tested. The experiment was performed in the dark to prevent oxidation, and phenol red-free media were used to reduce background fluorescence.

Briefly, CMs were seeded on 4-well chamber slides at a density of [5 × 10^4^] cells/well and allowed to adhere overnight under standard culture conditions. Following experimental treatments, the cells were incubated with 5 μM CellROX Green reagent diluted in complete culture medium for 30 min at 37 °C in a humidified incubator with 5% CO_2_. After incubation, the cells were washed twice with warm phosphate-buffered saline (PBS) to remove excess probe. For visualization, fluorescent images were captured immediately using a fluorescence microscope (excitation/emission: 485/520 nm) under identical exposure settings across all samples. The fluorescence intensity was measured using a microplate reader (excitation/emission: 485/520 nm) for quantitative analysis. Background fluorescence from wells incubated with probe-free medium was subtracted from each reading. ROS levels were expressed as relative fluorescence units (RFUs) normalized to the total cell number determined by DAPI staining. All experiments were performed in at least three independent replicates.

### 5.6. Assessment of Apoptosis and Necrosis by Triple Staining (Blue/Green/Red) Assay

Apoptosis and necrosis were evaluated using the Apoptosis/Necrosis Detection Kit (Blue/Green/Red) (Abcam, Cambridge, UK, Cat# ab176749) according to the manufacturer’s protocol, with minor optimizations for cardiomyocytes (CMs). CMs were seeded on a 2-well chamber slide at a density of 8 × 10^3^ cells/well and allowed to adhere overnight under standard culture conditions. After the experimental treatments, cells were washed once with phosphate-buffered saline (PBS) and incubated with the staining solution, prepared according to the kit instructions: Hoechst 33,342 (blue nuclear stain), Apopxin Green Indicator (detecting externalized phosphatidylserine during early apoptosis), and 7-Aminoactinomycin D (7-AAD; red, membrane-impermeant viability dye) diluted in binding buffer. Cells were incubated with the staining solution at room temperature for 30 min in the dark. Following incubation, cells were gently washed with PBS and immediately imaged using a fluorescence microscope equipped with DAPI (Hoechst, Ex/Em: ~350/461 nm), FITC (Green, Ex/Em: ~490/525 nm), and Texas Red (7-AAD, Ex/Em: ~550/650 nm) filter sets. Identical acquisition settings were maintained across all conditions. Quantitative analysis was performed by manually counting cells in at least five randomly selected fields per well or by automated image analysis using ImageJ. Apoptotic cells were identified as Hoechst-positive and Apopxin Green-positive but 7-AAD-negative; necrotic or late apoptotic cells were positive for both Apopxin Green and 7-AAD. Live cells were only positive for Hoechst staining. Data is presented as a percentage of the total cell population. All experiments were independently repeated at least three times.

### 5.7. Mitochondria Extraction and Protein Collection

From the cultured cells, mitochondria were extracted using the Mitochondrial Isolation Kit for cultured cells using the manufacturer’s instructions (Thermo Scientific) (#89874) for reagent-based methods. For analysis by Western blot, mitochondria were lysed with 2% CHAPS in Tris-buffered saline (TBS) applied to the pellet and vortexed for 1 min. Mitochondria were centrifuged at a high speed for 2 min. The supernatant contained a soluble mitochondrial protein that can be analyzed using the BCA protein assay.

### 5.8. Western Blotting 

The cells were homogenized on ice in NP-40 lysis buffer (RPI) containing protease inhibitors and a Halt proteinase inhibitor cocktail (Calbiochem, Darmstadt, Germany). Insoluble material was removed by centrifugation at 4 °C, and the protein concentration was determined in the supernatant using the BCA assay (Pierce, Fort Pierce, FL, USA) with bovine serum albumin used as the standard. Equal amounts of proteins were mixed with bromophenol blue at pH 6.8 and boiled at 95 °C for 5 min. Cell extracts were separated on a 10% sodium dodecyl sulfate-polyacrylamide gel and transferred to a nitrocellulose membrane. The membrane was blocked for 1 h in TBS supplemented with 0.05% Tween 20 (*vol/vol*) and 5% (*wt/vol*) non-fat dry milk (Sigma-Aldrich, Burlington, MA, USA). Incubation with the primary antibody (HIF1α (CST #37165s), HIF2α (CST #7096S), ERK (CST #9102S), pERK (CST #9101S), AKT (CST #9272S), pAKT (CST #9271S), BAX (CST #2772S), BCL2 (CST #15071S), GPX4 (CST #52455S), NRP-1 (CST #3725S), cleaved caspase-3 (CST #9661S), caspase-3 (CST #9662), PGC1α (CST #2178S), β-actin (Sigma)) was carried out at 4 °C overnight. After three washes (10 min each) with TBS containing 0.05% Tween 20 (wt/vol), the blot was incubated with the appropriate (anti-rabbit) horseradish peroxidase-linked (rb-HRP) secondary antibody (Sigma-Aldrich) and diluted to 1:3000 in TBS supplemented with 0.05% Tween 20 (*vol*/*vol*) and 1.6% (*wt*/*vol*) dry milk powder for 45 min at room temperature. The bands were visualized after three washes using the Clarity Western ECL Substrate, (Bio-RAD, Hercules, CA, USA).

### 5.9. Assessment of Mitochondrial Membrane Potential (ΔΨm) by JC-1 Staining

Mitochondrial membrane potential (ΔΨm) was evaluated using the JC-1(5,5′,6,6′-tetrachloro-1,1′,3,3′-tetraethylbenzimidazolocarbo-cyanine iodide) Mitochondrial Membrane Potential Assay Kit (Abcam, Cat# ab113850), following the manufacturer’s protocol with slight modifications to optimize for cardiomyocytes (CMs). H9c2 and HL-1 cardiomyocytes were seeded either on 96-well plates (for quantitative analysis) or onto glass coverslips placed on 24-well plates (for imaging) and allowed to adhere overnight. After experimental treatments, cells were incubated with JC-1 working solution (5 μM final concentration) for 15–30 min at 37 °C in a humidified 5% CO_2_ incubator, protected from light. Following incubation, cells were gently washed three times with warm phosphate-buffered saline (PBS) to remove excess dye. Mitochondrial membrane potential was assessed by fluorescence microscopy. Red fluorescence (aggregated JC-1, Ex/Em: ~585/590 nm) indicates polarized (healthy) mitochondria, whereas green fluorescence (monomeric JC-1, Ex/Em: ~510/527 nm) indicates depolarized (dysfunctional) mitochondria. Identical microscope settings were maintained across all samples to allow for comparison. Representative images were acquired, and the red-to-green fluorescence ratio was quantified using ImageJ. All experiments were independently performed at least three times.

### 5.10. Immunofluorescence Analysis 

Cells were placed on chambered cover slides for 24 h (5–10 × 10^4^ cells per chamber), fixed with 4% PFA for 15 min at room temperature, washed 3 times with PBS, and permeabilized with 0.1% Triton X-100 for 10 min at 4 °C. Cells were washed once with PBS and blocked with 1% BSA in PBS for 1 h at room temperature. Immunostaining using primary antibodies against *ki67* (Abcam #ab16667) was performed overnight at 4 °C. After three washes with PBS, secondary anti-rabbit IgG Alexa Fluor 568 or goat anti-rabbit IgG FITC; 1:200 dilution) was added and incubated for 1 h at room temperature. Nuclei were counterstained with DAPI (1 µg/mL, 5 min at room temperature), and coverslips were mounted onto slides using a fluorescence mounting medium.

### 5.11. Imaging 

Immunofluorescence imaging was performed using confocal microscopy to assess protein localization and expression in H9c2 and HL-1 cells. Imaging was performed using a confocal laser scanning microscope (Zeiss, Oberkochen, Germany, LSM 880) with appropriate excitation and emission filters. Z-stack images were acquired where necessary, and fluorescence intensity was quantified using ImageJ software (1.54g). Negative controls, including samples without primary antibodies, were included to confirm specificity.

### 5.12. Reverse Transcription Quantitative Real-Time Polymerase Chain Reaction (RT-qPCR)

Total RNA was extracted from the collected cells and the zebrafish hearts using Trizol Universal reagent (Thermo Fisher Scientific^TM^). Reverse transcription was then carried out using total RNA. cDNA was synthesized via reverse transcription using an iScriptTM RT Reagent Kit (RR037) according to the manufacturer’s protocols (BioRad, Hercules, CA, USA). Then, quantitative real-time PCR was performed using USB Veriquest^TM^ SYBR Green Master Mix affymetrix, Cleveland, OH, USA) according to the manufacturer’s protocols. The primers used to amplify genes in the reactions are presented in Supplementary Table S3. qPCR was performed using an Applied Biosystems QuantStudio 5 Real-Time System (Applied Biosystems, Foster City, CA, USA). The relative expression levels of the mRNAs were normalized to those of the reference gene *β-actin* and calculated using the 2−NNCT method. All reactions were repeated in triplicate.

### 5.13. H&E Staining 

The tissue was paraffin-embedded and cut into sections (5 μm). The paraffin sections were deparaffinized, and then hematoxylin was applied to stain the sections for 5 min. Afterwards, sections were dehydrated for 4 min. Eosin dye solution was applied for staining the sections for 5 min. Neutral gum was applied to mount the sections. A light microscope (Leica Microsystems, Buffalo Grove, IL, USA) was used to examine the slides after drying.

### 5.14. ATP Assay

The ATP levels in H9c2 cardiomyocytes were measured using the RealTime-Glo™ Extracellular ATP Assay (Promega, Madison, WI, USA, #GA5010) according to the manufacturer’s instructions. Cells were seeded on white opaque 96-well plates at a density of 1 × 10^4^ cells per well and treated as indicated. Following treatment, an equal volume of 2× RealTime-Glo™ (Madison, WI, USA) reagent mixture was added directly to each well, resulting in a final 1× concentration. Plates were incubated at 37 °C for 15–30 min to allow for signal stabilization, and luminescence was recorded using a microplate reader. Background luminescence was subtracted using blank wells containing media and assay reagent without cells. Luminescence intensity was directly proportional to extracellular ATP concentration, providing a sensitive, real-time measure of cellular ATP release under different experimental conditions.

### 5.15. Zebrafish Husbandry

Wild-type adult zebrafish (*Danio rerio*) were kept at 28.5 °C in a light–dark cycle (14 h of light and 10 h of darkness). Zebrafish embryos were maintained in a 100 mm Petri dish with E3 water at 28.5 °C until reaching 7 dpf, and then they were maintained in a recycling water system at the Mayo Clinic, Jacksonville, Florida. Animal density was kept constant after 28 dpf at 15–20 fish per 3 L as described ^1^. The *Tg(cmlc2:creER)* zebrafish lines were kindly donated by Dr. Xiaoli Xu’s lab at the Mayo Clinic, Rochester.

### 5.16. Generating a Cardiac-Specific VEGFB Expression Model in Zebrafish Using Heat Shock Induction 

To generate a transgenic zebrafish model with cardiac-specific VEGFB expression under heat shock induction, we employed a strategy adapted from Hoeppner et al. (2012) [18]. A heat shock-inducible construct was generated using the *hsp70* promoter, which enables temporal control of gene expression upon heat shock treatment. The coding sequence of zebrafish *VEGFB* was cloned downstream of the *hsp70* promoter in a Tol2 transposon vector to facilitate genomic integration. To ensure cardiac-specific visualization and facilitate screening, the construct also included a fluorescent reporter “green fluorescence protein (GFP)” under the control of an eye-specific *y-crystelin* promoter. Briefly, a cassette containing the *mCherry*, Danio rerio beta *actin* 3′UTR, SV40, loxP, zebrafish *vegfbb*, and rabbit *beta globin* polyA genes was synthesized as a clonal gene in the pTwist Amp High Copy (Twist Biosciences, San Francisco, CA, USA). The cassette was digested with SbfI and SpeI restriction enzymes and cloned in the *pKTol2H70-LP2mc-hVEGF-gcG* vector backbone linearized with SbfI and Spe 1 to generate *pKTol2H70-LP2mc-zfvegfbb-gcG*. The construction was verified through sequencing before microinjection.

### 5.17. Microinjection and Transgenesis

Fertilized AB zebrafish embryos at the one-cell stage were microinjected with the *pkTol2-h70-mC-hVEGFB-gcG* plasmid (12 ng/μL) along with Tol2 transposase mRNA (12.5 ng/μL) to facilitate genomic integration, as described previously. Embryos were screened for fluorescence expression at 48 h post-fertilization (hpf) to identify successfully injected individuals and founders (F0). Positive embryos were raised to adulthood, and stable F1 transgenic lines were established by outcrossing founders with wild-type zebrafish. F2- and F3-generation transgenic zebrafish were used for experiments.

### 5.18. Heat Shock Induction of VEGFB Expression

For the induction of VEGFB expression, 5–7 days post-fertilization (dpf) zebrafish larvae or adult zebrafish were transferred to pre-warmed system water maintained at 37 °C for 30 or 120 min. Following heat shock treatment, zebrafish were returned to standard conditions at 28.5 °C. Tissue samples were collected 6 h post-heat shock to confirm transgene activation.

### 5.19. Verification of Transgene Expression

To confirm the successful induction of VEGFB expression, total RNA was extracted from heart tissues using TRIzol reagent (ThermoFisher Scientific, Waltham, MA, USA) followed by cDNA synthesis. Quantitative PCR (qPCR) was performed using primers specific to *VEGFB* to assess transcript levels. Expression levels were normalized to *β-actin*. Immunofluorescence staining for VEGFB protein expression was also conducted on heart sections to confirm tissue-specific localization.

### 5.20. VEGFB Overexpression in the Heart

*Tg(cmlc2:CreER)* fish kindly donated by Dr. Xiaolei Xu from the Mayo Clinic, Rochester, were crossed with the cardiac-specific VEGFB overexpression line *Tg(pKTol2H70-LP2mc-zfvegfbb-gcG)* harboring a floxed stop cassette upstream of VEGFF-B and EGFP downstream to generate *the Tg(pKTol2H70-LP2mc-zfvegfbb-gcG);Tg(Cmlc2:CreER*) line. Progeny carrying both transgenes were identified via fluorescent screening for DsRed expression in the eye at 48–72 h post-fertilization (hpf). To activate a tissue-specific excision of the *VEGFB*, double-transgenic adult zebrafish were incubated in 400 mL of system water containing 1 µmol/L 4HT in a liter mini tank for approximately 24 h, as described earlier, leading to simultaneous VEGFB overexpression in cardiomyocytes.

### 5.21. Cryoinjury Model

To evaluate VEGFB’s protective role, zebrafish were subjected to a myocardial infarction (MI) model, as described previously [76]. Adult zebrafish were subjected to ventricular cryoinjury, as previously described [76]. Briefly, fish were anesthetized in 0.032% tricaine and placed ventral side up on a moist sponge. A small incision was made to expose the beating heart, and a pre-cooled cryoprobe (immersed in liquid nitrogen) was applied to the ventricular surface for 10–15 s to induce localized myocardial injury. Fish were then returned to fresh system water for recovery and maintained under standard conditions for subsequent analysis. Cardiac function was recorded at each time point as described before [77] using a Vevo 3100 ultrasound machine. Echocardiographic data were analyzed using Vevo LAB 5.11.0 software (VisualSonics, Brookvale, Australia). M-mode images from the parasternal long-axis view were used to measure fractional shortening. End-diastolic and end-systolic LV areas were determined to calculate the ejection fraction (EF). All measurements were averaged over three consecutive cardiac cycles to ensure consistency, and analyses were performed while blinded to the experimental groups. A total of *n* = 8 animals were used for analysis for each group.

### 5.22. Functional Analysis in Myocardial Infarction Model

Following heat shock induction of VEGFB expression, cardiac function was assessed via ultrasound imaging for ejection fraction and fractional shortening measurements in the *Tg(pKTol2H70-LP2mc-zfvegfbb-gcG);Tg(cmlc2:CreER)* line with and without MI [77]. Histological assessments were performed using hematoxylin (Gills R series 6765010, Epredia,Portsmouth, New Hampshire USA) and eosin (Epredia, Eosin Y Alcoholic, 71211, Portsmouth, NH, USA) (H&E) staining and cleaved caspase-3 immunostaining to evaluate tissue integrity and apoptosis levels. We assessed the ultrasound recording from *n* = 8 adult zebrafish in each group for heart function analysis. The histological analysis was performed for *n* = 3 animals in each group. Experimental groups were not randomized. Only healthy animals were used, and any animals displaying signs of illness or distress that may interfere with the analysis were excluded from the study.

### 5.23. Data Retrieval and Preprocessing

The human heart tissue’s single-cell RNA sequencing (scRNA-seq) data were obtained from the Human Protein Atlas (HPA) database, which compiles data from published studies based on healthy human tissues. This dataset comprises gene expression profiles for various tissues and cell types, with a primary focus on normal human heart tissue. To examine the expression of *VEGFB* and *NRP1*, we first retrieved UMAP (Uniform Manifold Approximation and Projection) plots directly from the HPA database by investigating the expression of these three genes in the context of healthy human heart tissue. These plots were used to visualize the overall expression distribution of *VEGFB* and *NRP1* across different cells in the heart.

### 5.24. Pathway Analysis

Enrichment analysis was performed using the ShinyGO 0.82 platform https://bioinformatics.sdstate.edu/ (accessed on 23 June 2025). The top 50 enriched pathways were plotted using a minimum false discovery rate (FDR) cutoff 0.05.

### 5.25. Gene Expression Correlation Analysis Across Human Tissues

To investigate the correlation between *NRP1* and *VEGFB* transcript expression across human tissues, we utilized publicly available RNA-sequencing data from the Genotype-Tissue Expression (GTEx) project (https://gtexportal.org/home/) (accessed on 12 June 2025). Transcript per million (TPM) values for *NRP1* and *VEGFB* were retrieved for all tissue types. Expression values were log-transformed prior to correlation analysis. Pearson correlation coefficients were calculated to assess the linear relationship between *NRP1* and *VEGFB* expression levels across all tissue types, and scatter plots were generated to visualize the expression patterns.

### 5.26. Cardiac Co-Expression Analysis in Ischemic and Nonischemic Heart Tissue

To evaluate co-expression patterns of *NRP1* and *VEGFB* in diseased versus healthy cardiac tissue, we used microarray data available in the iLINCS portal (Integrative LINCS genomics data portal; https://ilincs.org) (accessed on 12 June 2025). Specifically, we analyzed data from 37 samples obtained using the Affymetrix Human Genome U133A Array (HG-U133A). The dataset included myocardial tissue samples from patients with ischemic cardiomyopathy (ICM) and nonischemic cardiomyopathy (NICM), two primary etiologies of dilated cardiomyopathy (DCM). Co-expression analysis was conducted separately for NICM and ICM groups to determine the relationship between *NRP1* and *VEGFB* in each disease context. Expression normalization and differential correlation analysis were performed using the built-in statistical tools available within the iLINCS platform.

### 5.27. RNA-Seq Data Retrieval and Comparative Analysis

Publicly available RNA sequencing data were retrieved from the Gene Expression Omnibus (GEO) using the GREIN (Gene Expression Interactive Analysis) platform (https://www.ilincs.org/apps/grein/) (accessed on 12 June 2025), an interactive tool for accessing and analyzing uniformly processed RNA-seq data from the GEO. Specifically, the dataset GSE116250 was selected, which includes transcriptional profiles from human left ventricular tissue samples. This dataset contains 14 non-failing (control) heart and 13 ischemic heart failure (IHF) samples. Using GREIN’s built-in differential expression analysis tools (based on edgeR), we compared gene expressions between the non-heart failure and IHF groups to identify differentially expressed genes (DEGs) associated with ischemic heart failure. Genes with an adjusted *p*-value < 0.05 and a |log2 fold change| > 1 were considered significantly differentially expressed. To explore conserved ferroptotic signatures, we compared the DEGs identified in the IHF dataset with those obtained from our bulk RNA-seq analysis of H9c2 cardiomyocytes under hypoxia, VEGFB treatment, and *NRP1*-knockdown conditions. Overlapping gene expression patterns were assessed to identify shared ferroptosis-related transcripts and regulatory pathways altered during ischemic stress both in vivo (human heart tissue) and in vitro (H9c2 cell model).

### 5.28. RNA Sequencing and Data Analysis 

H9c2 rat cardiomyoblasts were cultured under standard conditions and subjected to three experimental treatments: normoxic control, hypoxia (200 µM CoCl_2__2_ for 24 h), and hypoxia following pre-treatment with recombinant VEGFB (50 ng/mL for 2 h). Total RNA was extracted using TRIzol™ (Invitrogen, Carlsbad, CA, USA), and RNA quality was confirmed with a NanoDrop™ spectrophotometer (Thermo Scientific, Hampton, NH, USA) and Agilent 2100 Bioanalyzer, Santa Clara, CA 95051, United States, ensuring RNA integrity numbers (RIN) ≥ 8.0. RNA sequencing and bioinformatic analysis were performed by MedGenome. Library preparation was conducted using the NEBNext^®^ Ultra™ II Directional RNA Library Prep Kit, Ipswich, MA, USA, and sequencing was performed on an Illumina NovaSeq 6000 platform to generate paired-end 150 bp reads with a depth of ~40 million reads per sample. Quality control, alignment to the *Rattus norvegicus* (Rnor_6.0) reference genome, and quantification of gene-level counts were performed using a standardized pipeline involving FastQC, Trim Galore, STAR, and feature Counts. Differential gene expression analysis was conducted using DESeq2 (http://bioconductor.org/packages/release/bioc/html/DESeq2.html (accessed on 12 June 2025)) (v1.10.1), with significant genes defined by an adjusted *p*-value < 0.05 and |log2 fold change| > 1. Functional enrichment of differentially expressed genes was assessed using clusterProfiler for Gene Ontology (GO) terms and KEGG pathway analysis, enabling identification of key biological processes and pathways altered by hypoxia and modulated by VEGFB treatment.

### 5.29. Ferroptosis-Related Genes

Ferroptosis-related genes were downloaded from the well-documented list of human ferroptosis-related genes; 259 ferroptosis-related genes were downloaded from the FerrDb V2 database (http://www.zhounan.org/ferrdb/current/) (accessed on 23 June 2025).

### 5.30. Co-Expression Network Analysis of NRP1 and VEGFB in Myocardial Injury

Differential gene expression data were obtained from the GREIN platform for five MI-relevant RNA-seq datasets (GSE173983, GSE156869, GSE127853, GSE115031 (human); GSE147929 (mouse)). Each dataset was analyzed independently to preserve biological context and avoid batch effects. Differential expression analysis compared MI samples to controls using species-specific pipelines. Co-expression analysis of *NRP1* and *VEGFB* was performed separately within each dataset. Genes with a Pearson correlation coefficient of R^2^ > 0.8 were identified as co-expressed.

### 5.31. Pathway Enrichment and Network Analysis

A core set of 245 genes, conserved across five MI-related RNA-seq datasets, was subjected to over-representation analysis using Gene Ontology (GO), Disease Ontology (DO), and KEGG databases. Functional enrichment was performed using clusterProfiler in R(4.4.2), with *p*-adjusted < 0.05 as the significance cutoff. Enriched GO terms highlighted key processes in MI, including apoptosis, angiogenesis, inflammation, and metabolic adaptation. DO terms confirmed cardiac and cerebral ischemia relevance, while KEGG pathways included cardiomyopathy, myocarditis, and vascular remodeling. To assess MI-specific network rewiring, we analyzed the top 200 genes most positively or negatively correlated with *NRP1* and *VEGFB* in GSE115031. Gene correlation was computed using Pearson coefficients, and separate GO enrichment analyses were conducted for the control and MI groups. This enabled identification of context-dependent shifts in *NRP1/VEGFB* regulatory networks during myocardial injury.

### 5.32. Statistical Analysis

All experimental data were analyzed using GraphPad Prism (Version 9) and presented as mean ± standard error of the mean (SEM). Each experiment was performed in at least three independent biological replicates to ensure reproducibility. For comparisons between two groups, an unpaired two-tailed Student’s *t*-test was used. For multiple-group comparisons, a one-way analysis of variance (ANOVA) followed by Tukey’s post hoc test was applied to determine statistical significance. For gene expression analysis via RT-qPCR, the ΔΔCt method was employed to calculate relative fold changes in mRNA levels, with target genes normalized to *β-actin* as the internal control. A *p*-value of less than 0.05 was considered statistically significant, with significance levels denoted as follows: * *p* < 0.05; ** *p* < 0.01; ** *p* < 0.001; and **** *p* < 0.0001. Data were visually represented using bar graphs or scatter plots, with error bars indicating the mean ± standard deviation (SD).

## Data Availability

The data supporting this study’s findings are available from the corresponding author on reasonable request. Please see the Major Resources Table in the Supplementary Materials. The online Supplementary Data presents an expanded version of the Methods section.

## Supplementary Materials

The following supporting information can be downloaded at https://www.mdpi.com/article/10.3390/cells14201642/s1, Figures S1–S10: Supplementary Figures; Table S1: Table showing list of differentially expressed genes between Control and Hypoxia, Table S2: Table showing list of differentially expressed genes between Hypoxia and Hypoxia + VEGFB, Table S3: qRT-PCR Primer Sequences.
