# Supplementary material for: Vascular Endothelial Growth Factor B Modulates Cardiac Functions via Ferroptosis Pathways in Post-Myocardial Infarction
Source: Cells. 2025 Oct 21;14(20):1642. doi: 10.3390/cells14201642 (PMC12562414; doi:10.3390/cells14201642)
Supplement: Supplementary file 1 [file cells-14-01642-s001.zip › cells-3908695-supplementary.pdf]

VEGF-B/NRP-1 Signaling modulates Mitochondrial Homeostasis and Cardiac Function After Myocardial Infarction

Sai Manasa Varanasi[1], Ankit Sabharwal[2,3,4], Shreyartha Mukherjee[3], Huzaifa Muhammad[1],Riya Kar[1], Carter Magnano[1], Ananya Dorairaj[1], Enfeng Wang[1], Shamit Dutta[1], Pritam das[1], Stephen C Ekker[2,3,4,7], Ying Wang[1,2,4], Debabrata Mukhopadhyay[1]*, Ramcharan Singh Angom[1]*

[1]Department of Biochemistry and Molecular Biology, Mayo Clinic College of Medicine and Sciences, Jacksonville, FL 32224, USA; varanasi.saimanasa@mayo.edu (S.M.V.); muhammad.huzaifa@mayo.edu (H.M.); kar.riya@mayo.edu (R.K.); magnano.carter@mayo.edu (C.M.); dorairaj.anya@mayo.edu (A.D.); wang.enfeng@mayo.edu (E.W.); dutta.shamit@mayo.edu (S.D.); Das.pritam@mayo.edu (P.D.); wang.ying@mayo.edu (Y.W.)

[2] Department of Biochemistry and Molecular Biology, Mayo Clinic College of Medicine and Science, Rochester, MN 55905, USA; ankit.sabharwal@austin.utexas.edu (A.S.); stephen.ekker@austin.utexas.edu (S.C.E.)

[3] Department of Pediatrics, Dell Pediatric Research Institute, Dell Medical School, The University of Texas at Austin, Austin, TX 78712, USA

[4] Center for Rare Disease, Dell Medical School, The University of Texas at Austin, Austin, TX 78712, USA

[5] Department of Quantitative Health Sciences, Mayo Clinic, Jacksonville, FL 32224, USA; mukherjee.shreyartha@mayo.edu

[6] College of Medicine, Alfaisal University, Riyadh 11533, Saudi Arabia

[7] Department of Molecular Biosciences, The University of Texas at Austin, Austin, TX 78712, USA

[8] Department of Cardiovascular Medicine, Mayo Clinic, Rochester, MN 55905, USA; wang.ying@mayo.edu (Y.W.)

* Correspondence: mukhopadhyay.debabrata@mayo.edu (D.M.); angom.ramcharan@mayo.edu (R.S.A.);
Tel.: +1-904-953-6177 (D.M.); +1-904-953-0615 (R.S.A.)

Correspondence:

1. Ramcharan Singh Angom. Assistant Professor, Department of Biochemistry and Molecular. Biology, Mayo Clinic, Jacksonville, Florida, 32224, USA. E-mail: angom.ramcharan@mayo.edu

2. Debabrata Mukhopadhyay. Professor, Department of Biochemistry and Molecular. Biology, Mayo Clinic, Jacksonville, Florida, 32224, USA. E-mail: mukhopadhyay.debabrata@mayo.edu

Supplementary Figures

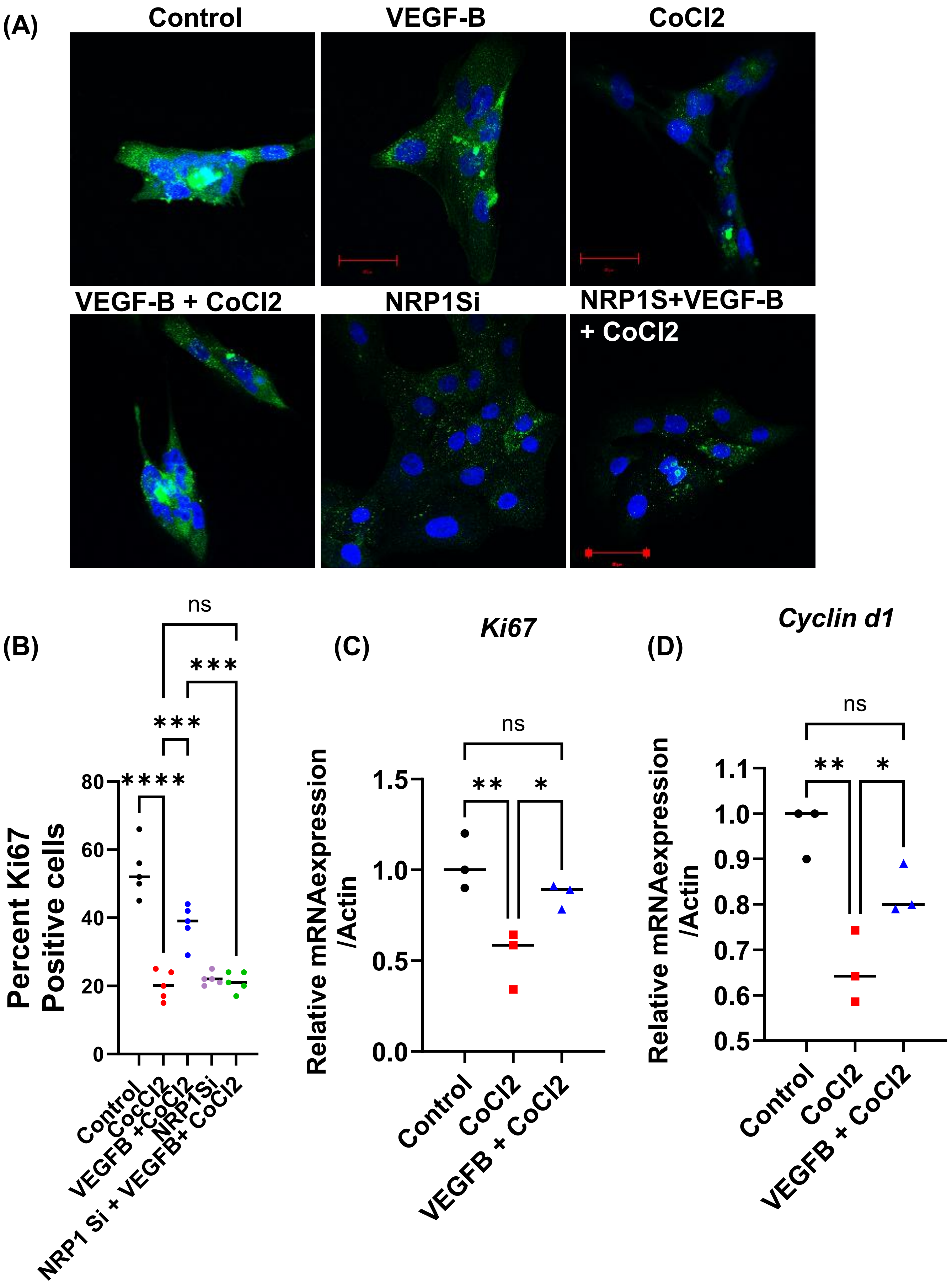
(A)
Control
VEGF-B
CoCl2
VEGF-B + CoCl2
NRP1Si
NRP1S+VEGF-B
+ CoCl2
(B)
Percent Ki67
Positive cells
ns
***
***
****
80
60
40
20
0
Control
CocCl2
VEGFB +CoCl2
NRP1Si
NRP1 Si + VEGFB+ CoCl2
(C)
Ki67
Relative mRNAexpression
/Actin
ns
**
*
1.5
1.0
0.5
0.0
Control
CoCl2
VEGFB + CoCl2
(D)
Cyclin d1
Relative mRNAexpression
/Actin
ns
**
*
1.1
1.0
0.9
0.8
0.7
0.6
0.5
Control
CoCl2
VEGFB + CoCl2

Figure S1. **VEGFB Enhances Cardiomyocyte Proliferation Following Hypoxia Treatment. (A) Confocal Image showing representative HL-1 cells stained with Ki67. (B)** Quantification of HL-1 cell proliferation following treatment with hypoxia ($CoCl_2$), VEGFB, NRP1 siRNA (NRP1Si), and their combinations. (C-D) RT-qPCR analysis of Ki67 and cyclin expression in HL-1 cardiomyocytes treated with hypoxia ($CoCl_2$) alone or with VEGFB + $CoCl_2$ and Combinations. Scatter plots display individual data points from independent experiments. Horizontal bars represent the mean ± SD from at least three independent replicates. Statistical significance is indicated as *, $p < 0.05$, **$p < 0.01$, ***$p < 0.001$, and ****$p < 0.0001$.Scale bar = 50μm.

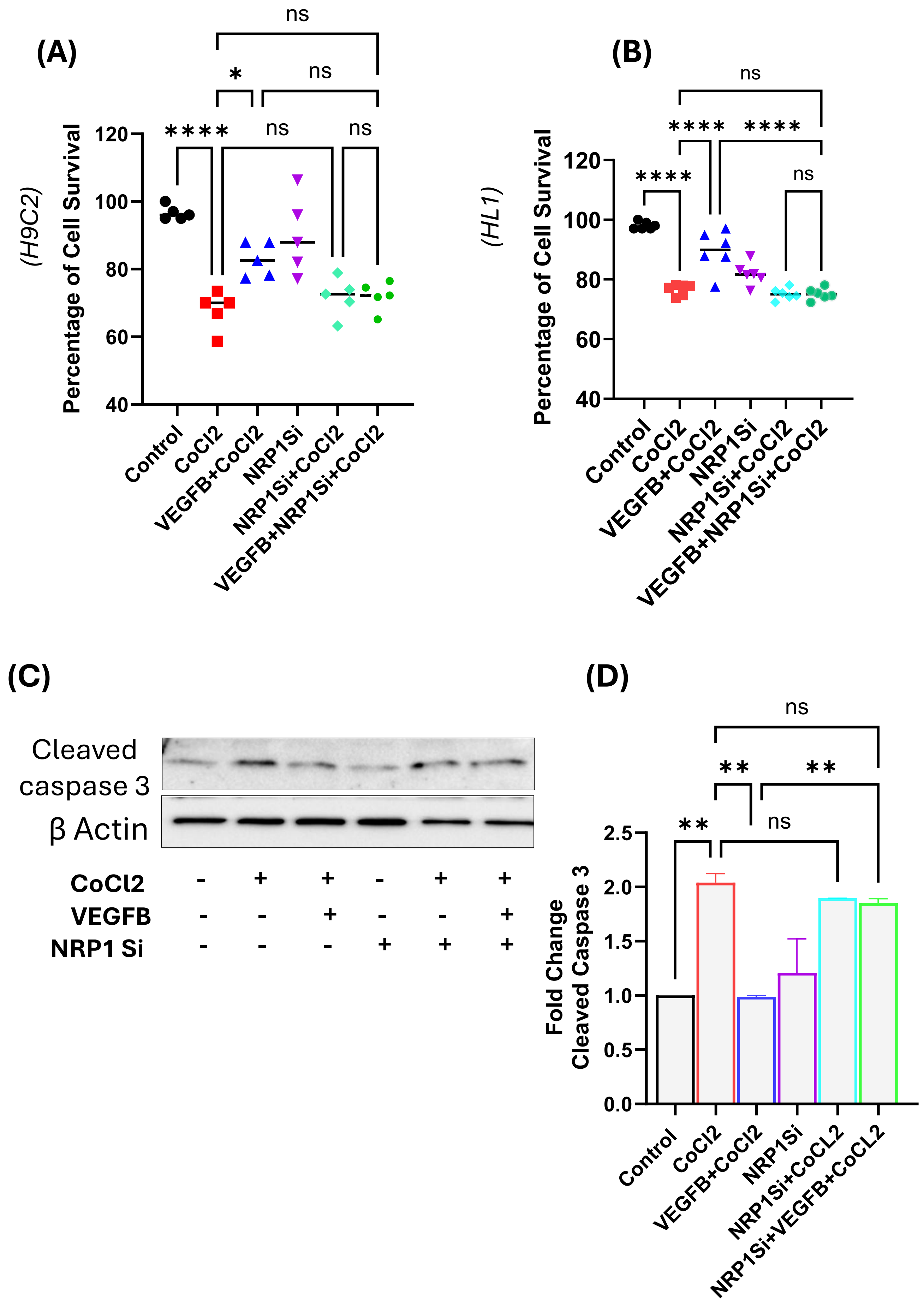

(A)
(H9C2)
Percentage of Cell Survival
120
100
80
60
40
ns
*
ns
****
ns
ns
Control
CoCl2
VEGFB+CoCl2
NRP1Si
NRP1Si+CoCl2
VEGFB+NRP1Si+CoCl2
(B)
(HL1)
Percentage of Cell Survival
120
100
80
60
40
ns
****
****
****
ns
Control
CoCl2
VEGFB+CoCl2
NRP1Si
NRP1Si+CoCl2
VEGFB+NRP1Si+CoCl2
(C)
Cleaved caspase 3
β Actin
CoCl2 - + + - + +
VEGFB - - + - - +
NRP1 Si - - - + + +
(D)
Fold Change Cleaved Caspase 3
2.5
2.0
1.5
1.0
0.5
0.0
ns
**
**
**
ns
Control
CoCl2
VEGFB+CoCl2
NRP1Si
NRP1Si+CoCL2
NRP1Si+VEGFB+CoCL2

Figure S2. VEGFB Modulates Protein Expression of Cell Proliferation and Apoptosis Pathways in HL-1 Cardiomyocytes. (A) MTS assay showing VEGFB-mediated prevention of H9C2 CM death. **(B)** MTS assay showing VEGFB-mediated prevention of HL-1 CM death. **(C)** Western blot analysis showing protein expression levels of Cleaved caspase 3 apoptotic markers in HL-1 cardiomyocytes. **(D)** Quantification of the Western blot bands shown in (C). Scatter plots display individual data points (n=5). Horizontal bars represent the mean ± SD from at least two independent replicates. Statistical significance is indicated as *, $p < 0.05$, **$p < 0.01$, ***$p < 0.001$, and ****$p < 0.0001$.

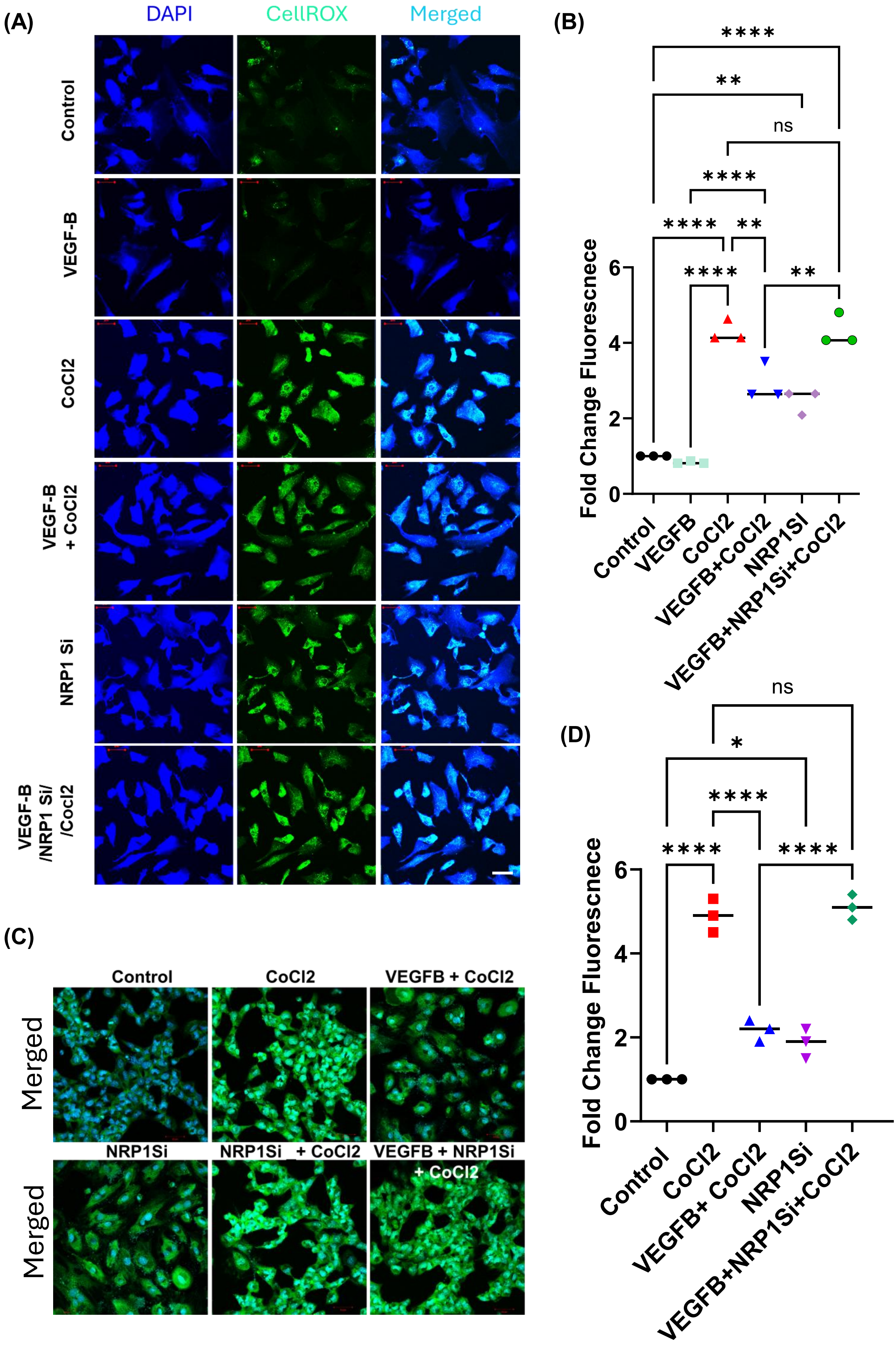
(A)
DAPI
CellROX
Merged
Control
VEGF-B
CoCl2
VEGF-B + CoCl2
NRP1 Si
VEGF-B /NRP1 Si/ /Cocl2
(B)
Fold Change Fluorescnece
0
2
4
6
****
**
ns
****
****
**
****
**
Control
VEGFB
CoCl2
VEGFB+CoCl2
NRP1SI
VEGFB+NRP1Si+CoCl2
(C)
Control
CoCl2
VEGFB + CoCl2
Merged
NRP1Si
NRP1Si + CoCl2
VEGFB + NRP1Si + CoCl2
Merged
(D)
ns
*
****
****
****
Fold Change Fluorescnece
0
2
4
6
Control
CoCl2
VEGFB+ CoCl2
NRP1Si
VEGFB+NRP1Si+CoCl2

Figure S3. VEGFB Reduces $CoCl_2$-Induced ROS Production in Cardiomyocytes via NRP1-Dependent Mechanism. **(A)** Intracellular ROS levels were measured in H9c2 cardiomyocytes (CMs) using a ROS-sensitive fluorescent probe following treatments with $CoCl_2$ (200 μM), VEGF-B (20 ng/mL), NRP1 siRNA, and their combinations (Control, VEGF-B treated, $CoCl_2$ treatment significantly increased ROS production compared to untreated controls, VEGF-B reduced ROS levels in $CoCl_2$-treated cells, indicating a protective effect against hypoxia-induced oxidative stress. However, NRP1 knockdown, reversed the antioxidant effect of VEGFB, resulting in ROS levels similar to or higher than those observed with $CoCl_2$ alone. **(B)** Quantification. **(C)** Confocal image showing the cellRox assay to analyse the effect of CoCl2 and shRNA-mediated NRP1 depletion on H9c2 cells. **(D)** Quantification of C.Scatter plots display individual data points from independent experiments. Horizontal bars represent the mean ± SD from at least three independent replicates. Statistical significance was determined by one-way ANOVA followed by Tukey's post-hoc test (*p < 0.05, **, p < 0.01, ***, p<0.001 and ****, p<0.0001). Scale bar = 50μm.

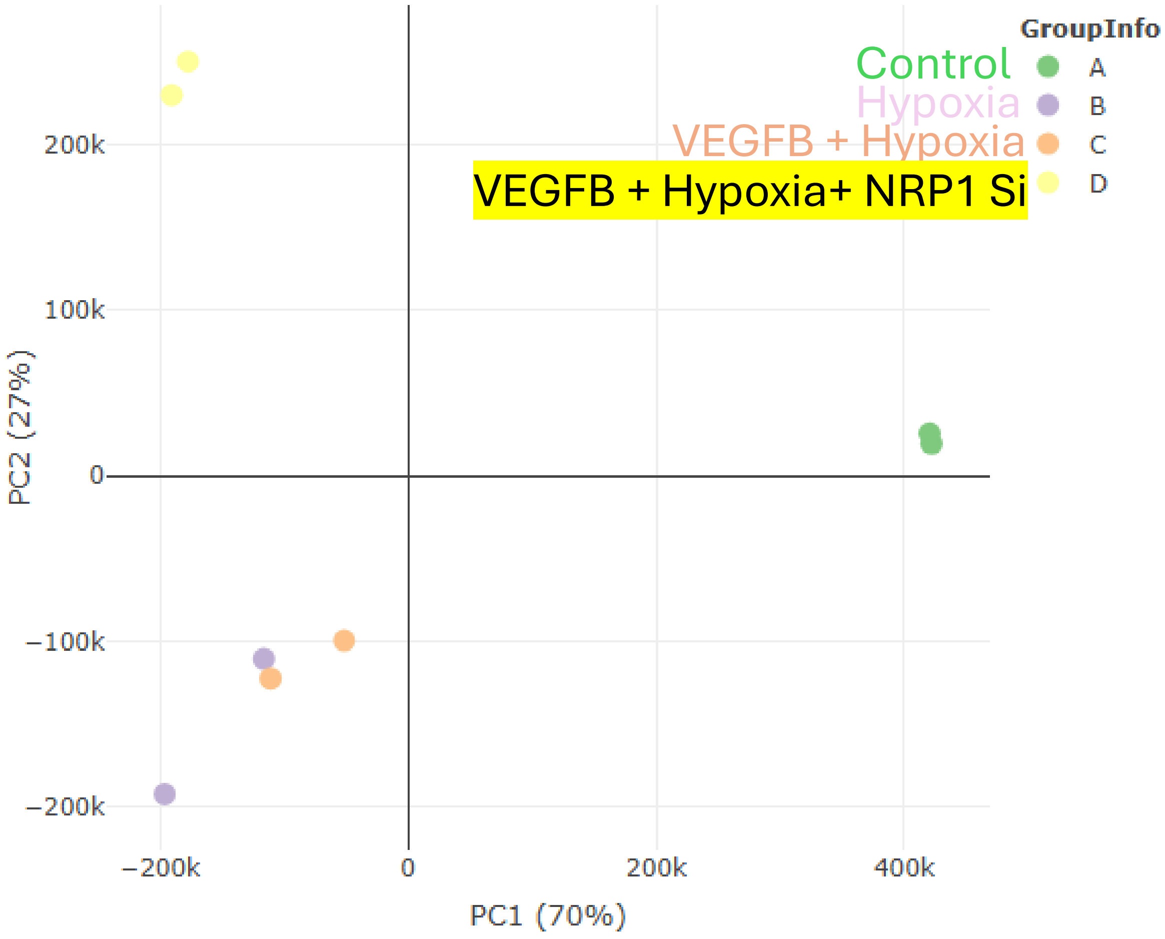

Figure S4. Principal component analysis (PCA) showing distinct clustering of normoxia, hypoxia, and VEGFB + Hypoxia treated samples.

(A)

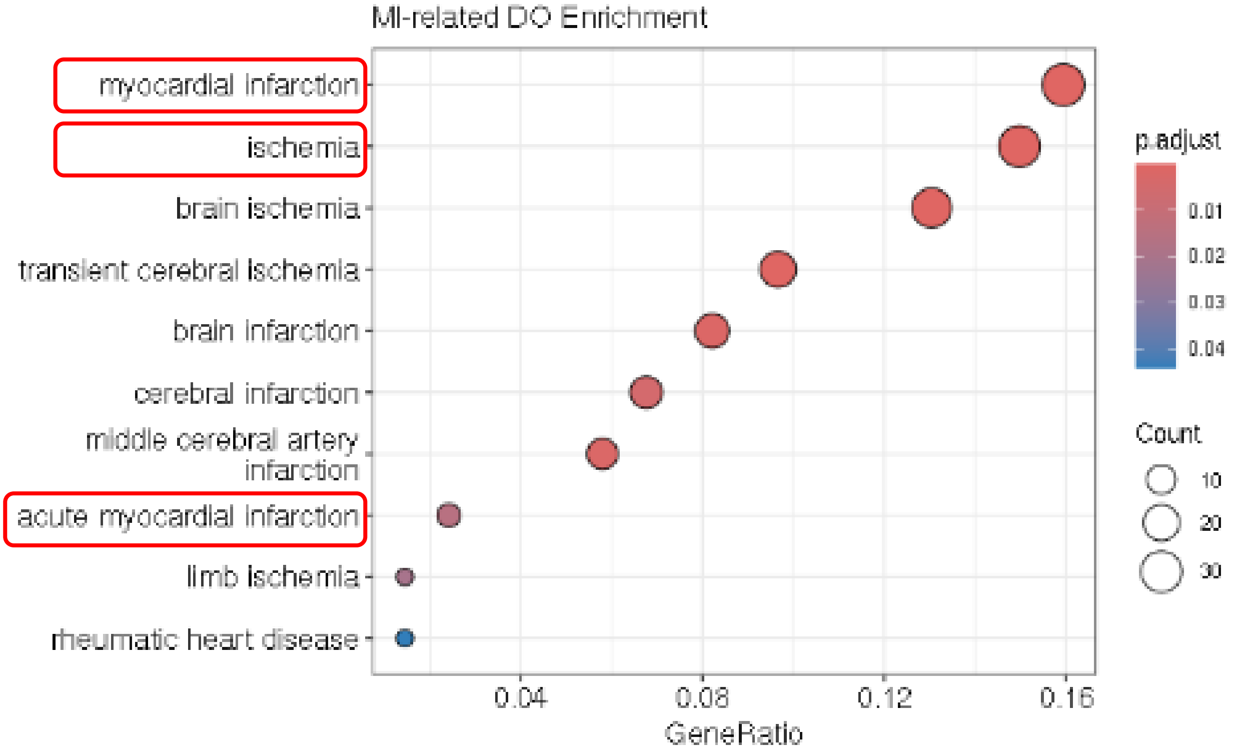

MI-related DO Enrichment
myocardial infarction
ischemia
brain ischemia
transient cerebral ischemia
brain infarction
cerebral infarction
middle cerebral artery infarction
acute myocardial infarction
limb ischemia
rheumatic heart disease
0.04
0.08
0.12
0.16
GeneRatio
p.adjust
0.01
0.02
0.03
0.04
Count
10
20
30

(B)

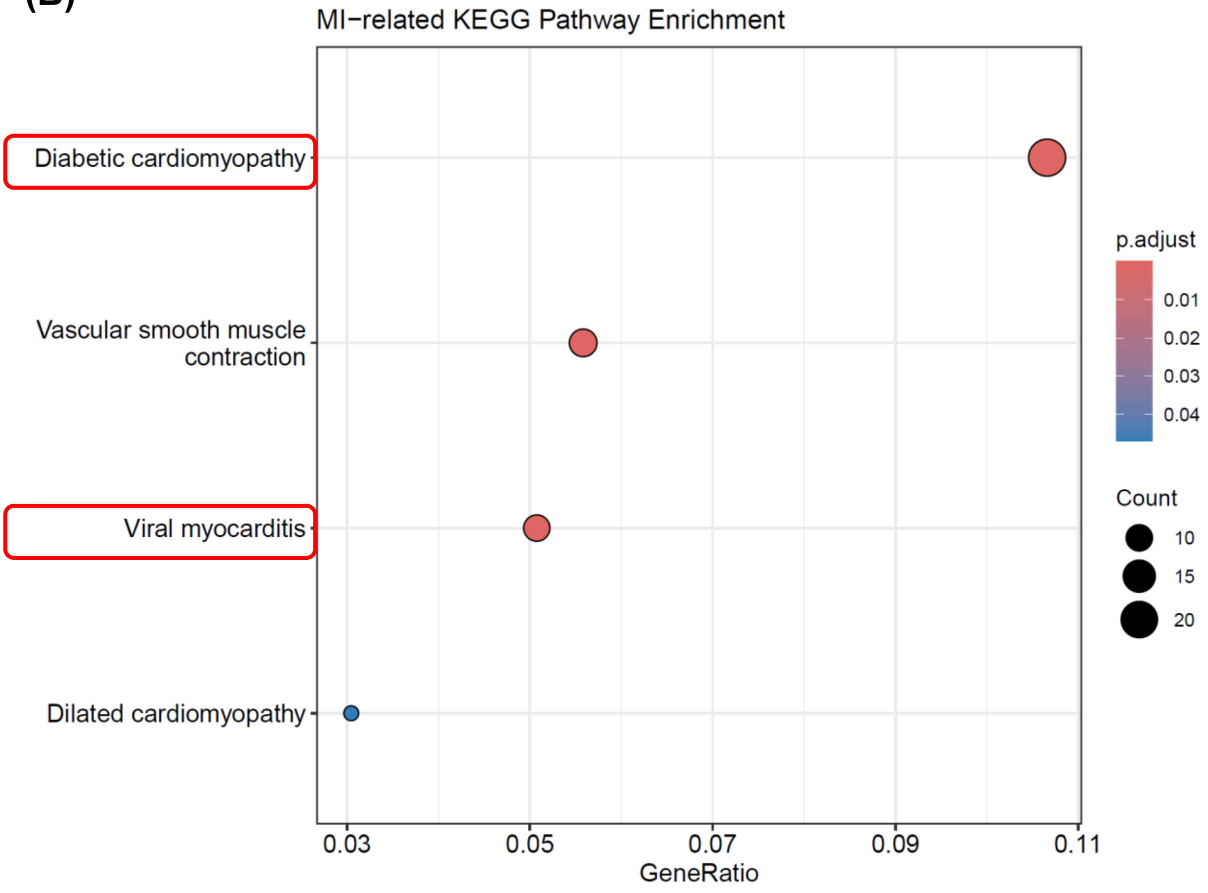

MI−related KEGG Pathway Enrichment
Diabetic cardiomyopathy
Vascular smooth muscle contraction
Viral myocarditis
Dilated cardiomyopathy
0.03
0.05
0.07
0.09
0.11
GeneRatio
p.adjust
0.01
0.02
0.03
0.04
Count
10
15
20

Figure S5. Enrichment Analysis of MI-Associated Gene Set Highlights Shared Pathways in Ischemic and Cardiometabolic Disorders. (A) Disease Ontology (DO) enrichment mapping of the MI-associated gene set revealed significant associations with myocardial infarction, cardiac and cerebral ischemia, and transient cerebral ischemia, underscoring the clinical relevance and cross-organ impact of the gene network. **(B)** KEGG pathway analysis further identified enrichment in related conditions, including diabetic cardiomyopathy, viral myocarditis, vascular smooth muscle contraction, and dilated cardiomyopathy.

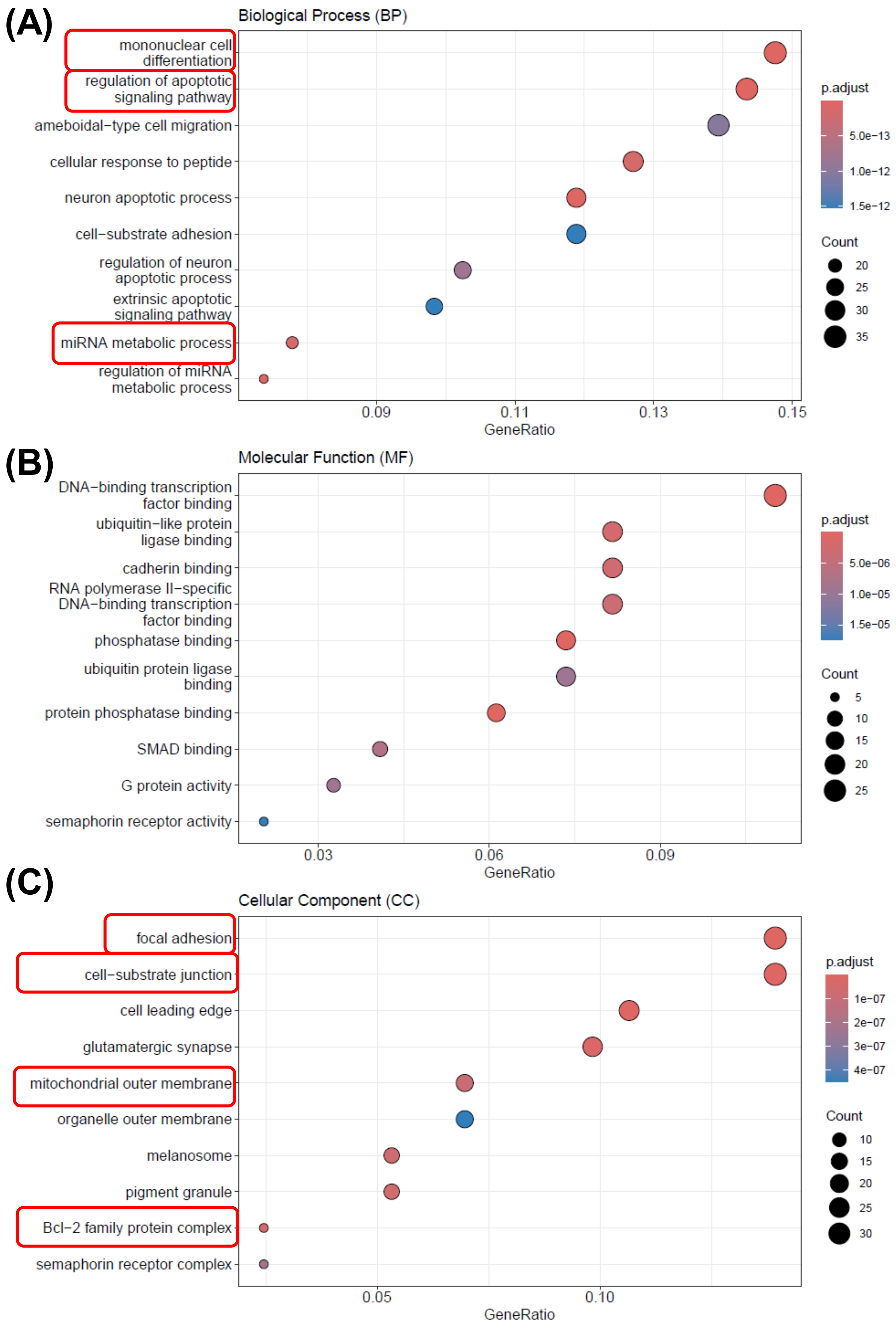
(A)
Biological Process (BP)
mononuclear cell differentiation
regulation of apoptotic signaling pathway
ameboidal−type cell migration
cellular response to peptide
neuron apoptotic process
cell−substrate adhesion
regulation of neuron apoptotic process
extrinsic apoptotic signaling pathway
miRNA metabolic process
regulation of miRNA metabolic process
0.09
0.11
0.13
0.15
GeneRatio
p.adjust
5.0e−13
1.0e−12
1.5e−12
Count
20
25
30
35
(B)
Molecular Function (MF)
DNA−binding transcription factor binding
ubiquitin−like protein ligase binding
cadherin binding
RNA polymerase II−specific DNA−binding transcription factor binding
phosphatase binding
ubiquitin protein ligase binding
protein phosphatase binding
SMAD binding
G protein activity
semaphorin receptor activity
0.03
0.06
0.09
GeneRatio
p.adjust
5.0e−06
1.0e−05
1.5e−05
Count
5
10
15
20
25
(C)
Cellular Component (CC)
focal adhesion
cell−substrate junction
cell leading edge
glutamatergic synapse
mitochondrial outer membrane
organelle outer membrane
melanosome
pigment granule
Bcl−2 family protein complex
semaphorin receptor complex
0.05
0.10
GeneRatio
p.adjust
1e−07
2e−07
3e−07
4e−07
Count
10
15
20
25
30

Figure S6. **Gene Ontology (GO) Enrichment Analysis Reveals Key Biological Processes Underlying Myocardial Infarction Pathophysiology**. **(A-C)** GO enrichment of the MI-associated gene set highlights critical processes involved in cardiac injury, repair, and remodeling.

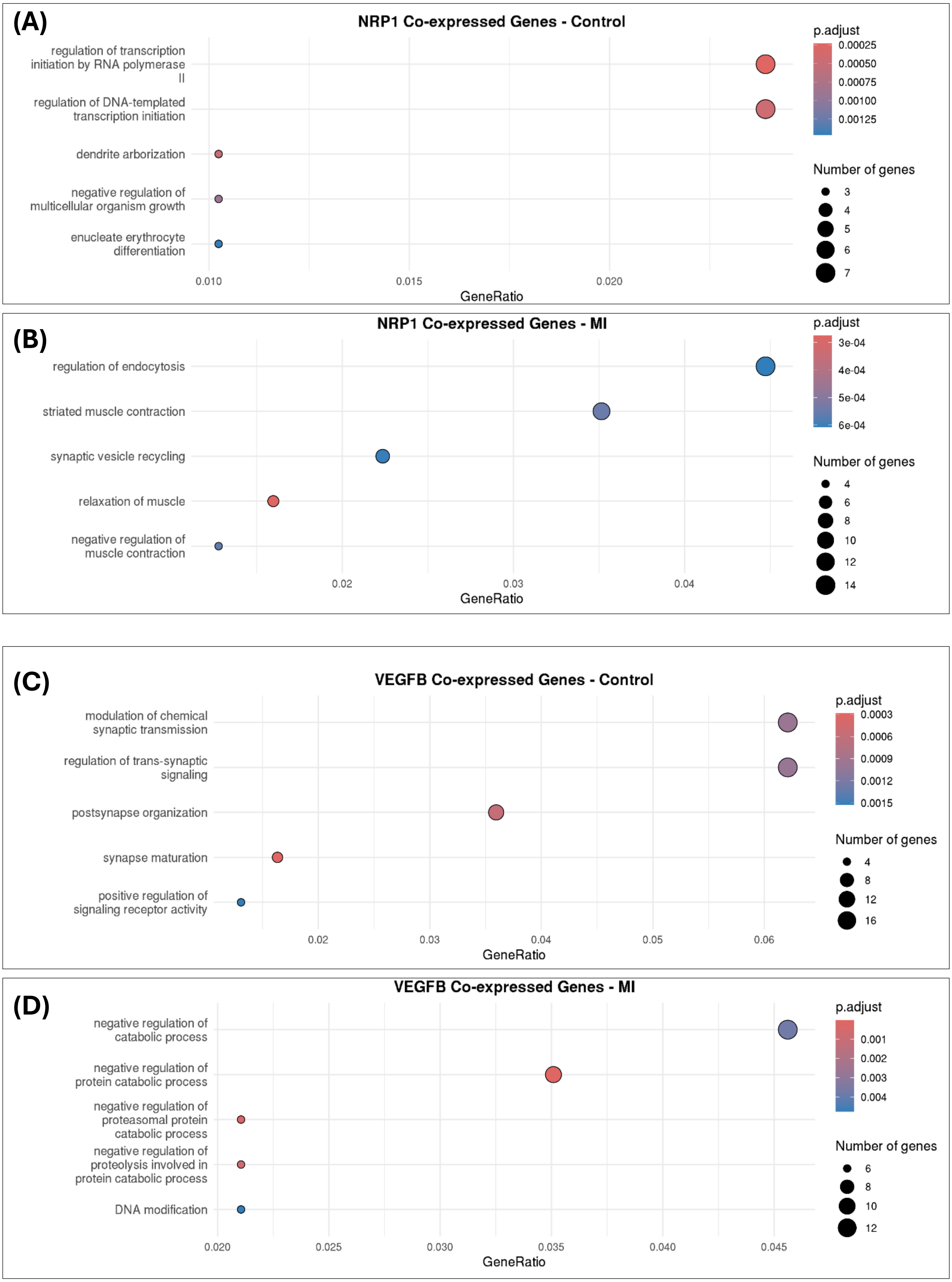
(A)
NRP1 Co-expressed Genes - Control
regulation of transcription initiation by RNA polymerase II
regulation of DNA-templated transcription initiation
dendrite arborization
negative regulation of multicellular organism growth
enucleate erythrocyte differentiation
0.010
0.015
0.020
GeneRatio
p.adjust
0.00025
0.00050
0.00075
0.00100
0.00125
Number of genes
3
4
5
6
7
(B)
NRP1 Co-expressed Genes - MI
regulation of endocytosis
striated muscle contraction
synaptic vesicle recycling
relaxation of muscle
negative regulation of muscle contraction
0.02
0.03
0.04
GeneRatio
p.adjust
3e-04
4e-04
5e-04
6e-04
Number of genes
4
6
8
10
12
14
(C)
VEGFB Co-expressed Genes - Control
modulation of chemical synaptic transmission
regulation of trans-synaptic signaling
postsynapse organization
synapse maturation
positive regulation of signaling receptor activity
0.02
0.03
0.04
0.05
0.06
GeneRatio
p.adjust
0.0003
0.0006
0.0009
0.0012
0.0015
Number of genes
4
8
12
16
(D)
VEGFB Co-expressed Genes - MI
negative regulation of catabolic process
negative regulation of protein catabolic process
negative regulation of proteasomal protein catabolic process
negative regulation of proteolysis involved in protein catabolic process
DNA modification
0.020
0.025
0.030
0.035
0.040
0.045
GeneRatio
p.adjust
0.001
0.002
0.003
0.004
Number of genes
6
8
10
12

Figure S7. GO:BP Overrepresentation Analysis of Genes Strongly Correlated with NRP1 and VEGFB Expression in Myocardial Infarction (MI). (A-B) Top five enriched Gene Ontology Biological Process (GO:BP) categories for genes most strongly co-expressed with NRP1 reveal context-specific functional reprogramming during myocardial infarction. **(A)** In control conditions, NRP1 is associated with transcriptional regulation and neuronal development (e.g., "regulation of transcription initiation"), while under MI, its network shifts toward muscle-specific processes such as "striated muscle contraction" and "relaxation of muscle," suggesting a role in maintaining cardiac contractility under ischemic stress **(B)**. In contrast, VEGFB transitions from synaptic development in healthy tissue **(C)** to regulation of protein homeostasis during MI **(D)**, notably enriching "negative regulation of proteasomal protein catabolism" (p = 1.32e-05), indicative of a protective mechanism against excessive protein degradation.

(A)

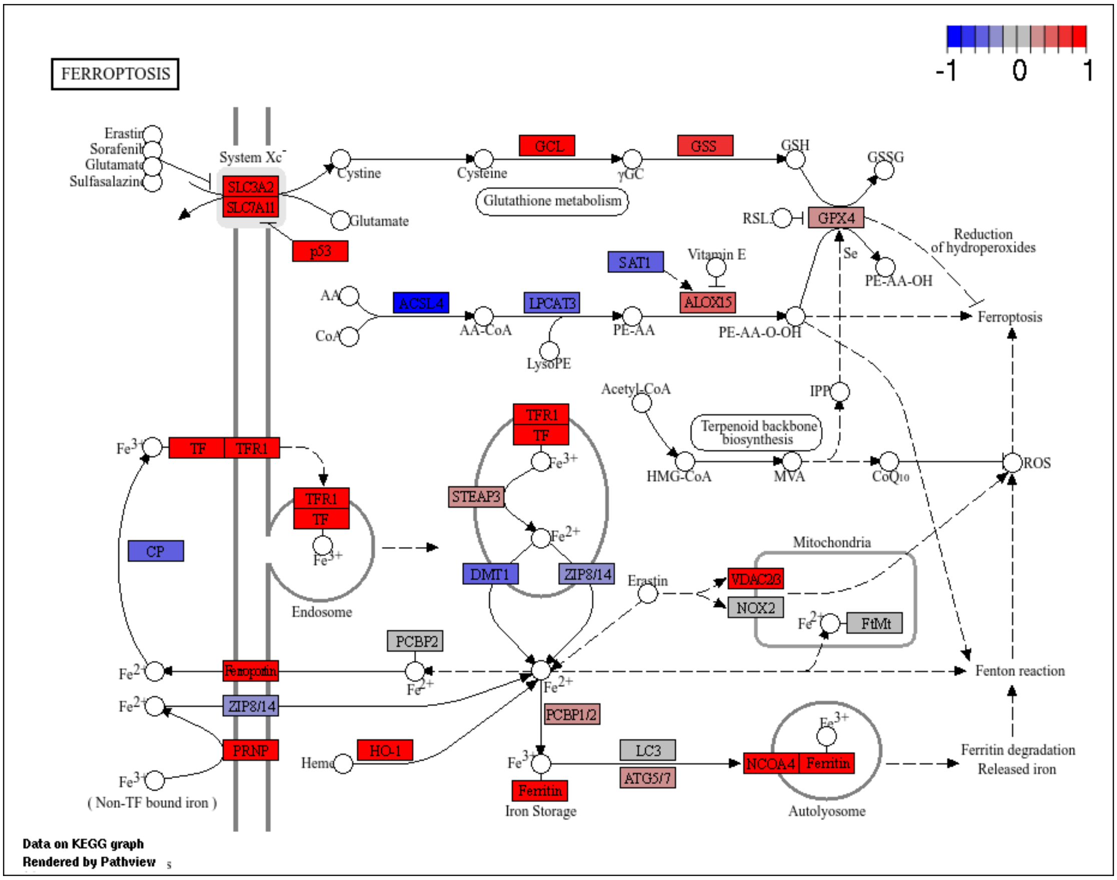

FERROPTOSIS
-1
0
1
Erastin
Sorafenib
Glutamate
Sulfasalazine
System Xc-
SLC3A2
SLC7A11
Cystine
Glutamate
p53
Cysteine
GCL
γGC
GSS
GSH
GSSG
Glutathione metabolism
RSL3
GPX4
Se
PE-AA-OH
Reduction of hydroperoxides
SAT1
Vitamin E
ALOX15
AA
CoA
ACSL4
AA-CoA
LPCAT3
LysoPE
PE-AA
PE-AA-O-OH
Ferroptosis
Acetyl-CoA
IPP
Terpenoid backbone biosynthesis
HMG-CoA
MVA
CoQ10
ROS
TF
TFR1
STEAP3
Endosome
CP
DMT1
ZIP8/14
Erastin
Mitochondria
VDAC2/3
NOX2
FtMt
PCBP2
Ferroportin
Fenton reaction
ZIP8/14
PCBP1/2
PRNP
Heme
HO-1
LC3
ATG5/7
NCOA4
Ferritin
Ferritin degradation
Released iron
Iron Storage
Autolyosome
(Non-TF bound iron)
Data on KEGG graph
Rendered by Pathview

(B)

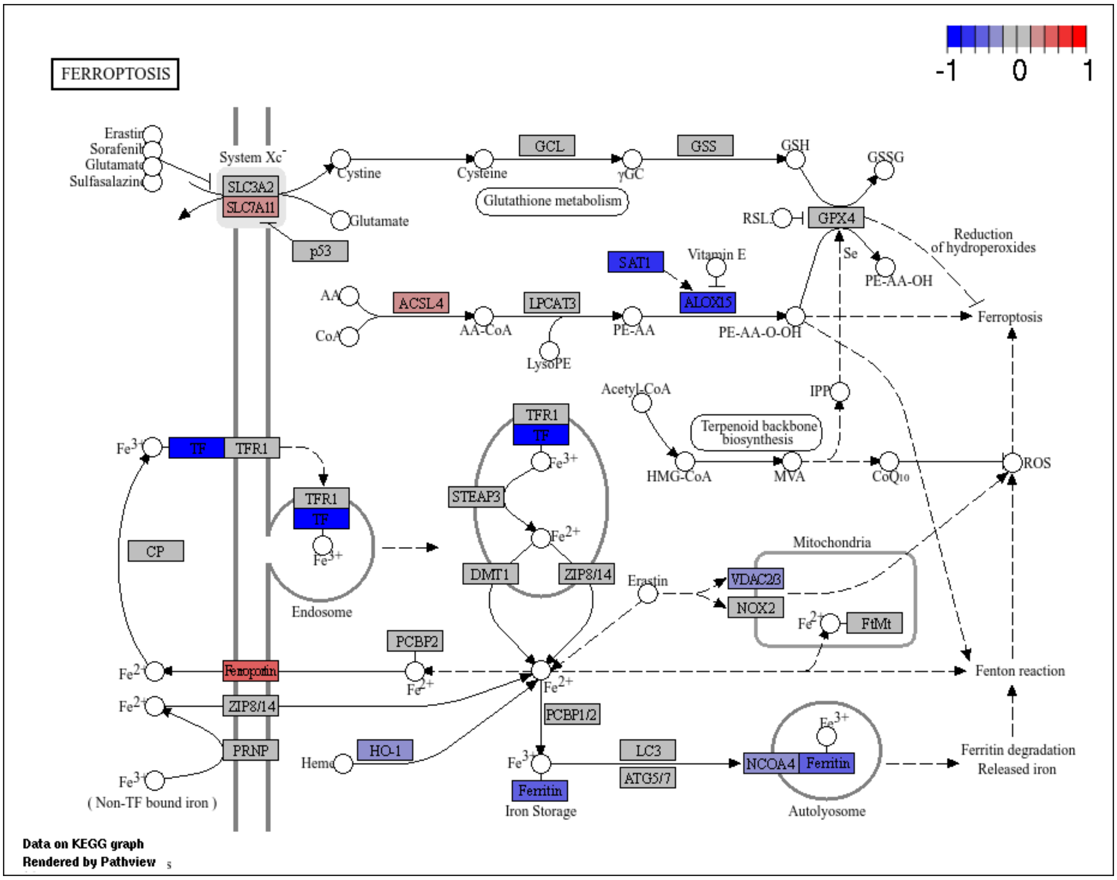

FERROPTOSIS
-1
0
1
Erastin
Sorafenib
Glutamate
Sulfasalazine
System Xc-
SLC3A2
SLC7A11
Cystine
Glutamate
p53
Cysteine
GCL
γGC
GSS
GSH
GSSG
Glutathione metabolism
RSL3
GPX4
Se
PE-AA-OH
Reduction of hydroperoxides
SAT1
Vitamin E
ALOX15
AA
CoA
ACSL4
AA-CoA
LPCAT3
LysoPE
PE-AA
PE-AA-O-OH
Ferroptosis
Acetyl-CoA
IPP
Terpenoid backbone biosynthesis
HMG-CoA
MVA
CoQ10
ROS
TF
TFR1
STEAP3
Endosome
CP
DMT1
ZIP8/14
Erastin
Mitochondria
VDAC2/3
NOX2
FtMt
PCBP2
Ferroportin
Fenton reaction
ZIP8/14
PCBP1/2
PRNP
Heme
HO-1
LC3
ATG5/7
NCOA4
Ferritin
Ferritin degradation
Released iron
Iron Storage
Autolyosome
(Non-TF bound iron)
Data on KEGG graph
Rendered by Pathview

Figure S8. VEGF-B Modulates Hypoxia-Induced Ferroptosis Pathway Gene Expression in Cardiomyocytes. KEGG pathway-based visualization of the ferroptosis pathway (rno04216) in Rattus norvegicus using the Pathview R package. Gene expression changes were mapped for two comparisons: **(A)** Control vs. Hypoxia and **(B)** Hypoxia vs. VEGFB + Hypoxia, based on RNA-seq data from primary rat cardiomyocytes. Genes are color-coded by log2 fold change (red = upregulated; green = downregulated).

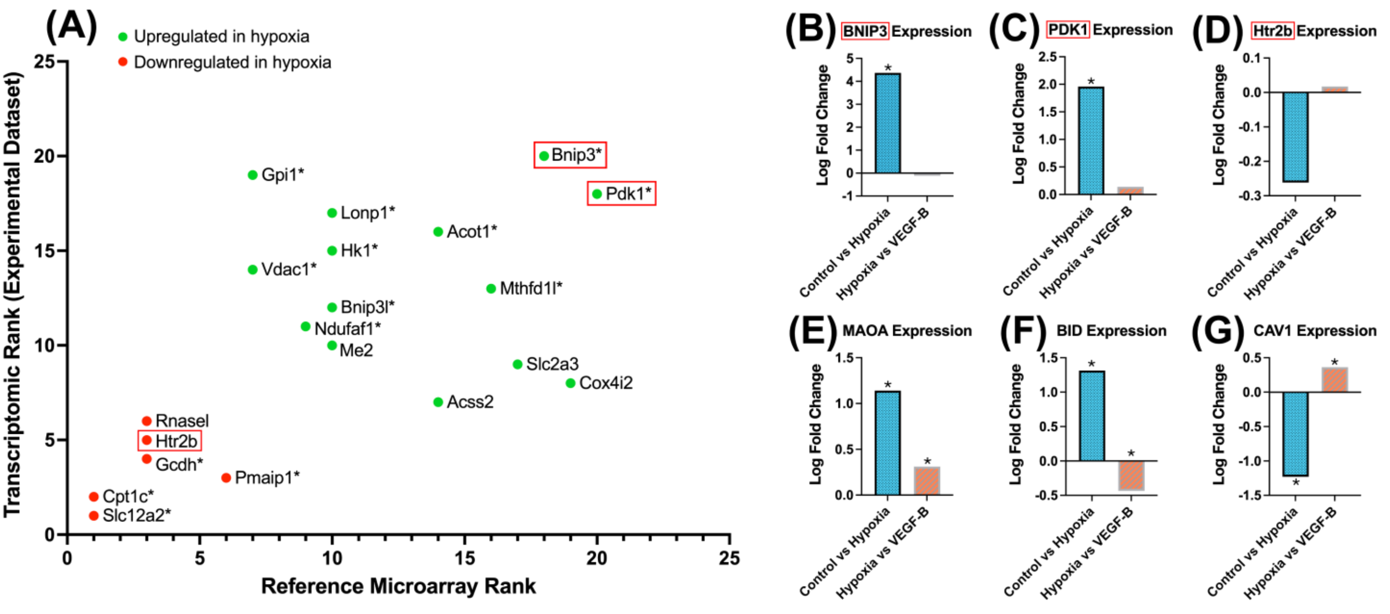

- **Figure S9: Rank-based analysis and expression profiling of hypoxia-responsive mitochondrial genes reveal VEGF-B–sensitive targets**. **(A)** A rank-based comparison of mitochondrial gene expression under hypoxia, among our transcriptomic dataset (H9C2 rat cardiomyocytes) with previously reported patterns (C57BL/6 mice cardiomyocytes). Genes were independently ranked in ascending order within each dataset to standardize scale and preserve relative expression trends. This approach enabled visualization of concordant directionality between datasets, highlighting shared regulatory shifts. **(B–D)** Bar plots showing **$\log_2$ fold** changes in expression of BNIP3, PDK1, and Htr2b under hypoxia (vs. control) and following VEGF-B treatment (vs. hypoxia). BNIP3 and PDK1 were significantly upregulated under hypoxia ($P < 0.05$) and showed marked attenuation with VEGF-B. Htr2b was modestly downregulated under hypoxia and exhibited a mild reciprocal increase with VEGF-B. (**E–G**) **Log_2 fold** change expression of MAOA, BID, and CAV1 under hypoxia and following VEGF-B treatment. All three genes were significantly modulated by hypoxia ($P < 0.05$) and exhibited statistically significant reversal with VEGF-B ($P < 0.05$), reinforcing its protective effect. BID and CAV1, in particular, showed clear bidirectional regulation, reversing direction relative to their hypoxic response; highlighting VEGF-B's potential to mitigate ferroptosis-associated mitochondrial dysfunction. *, $p<0.05$

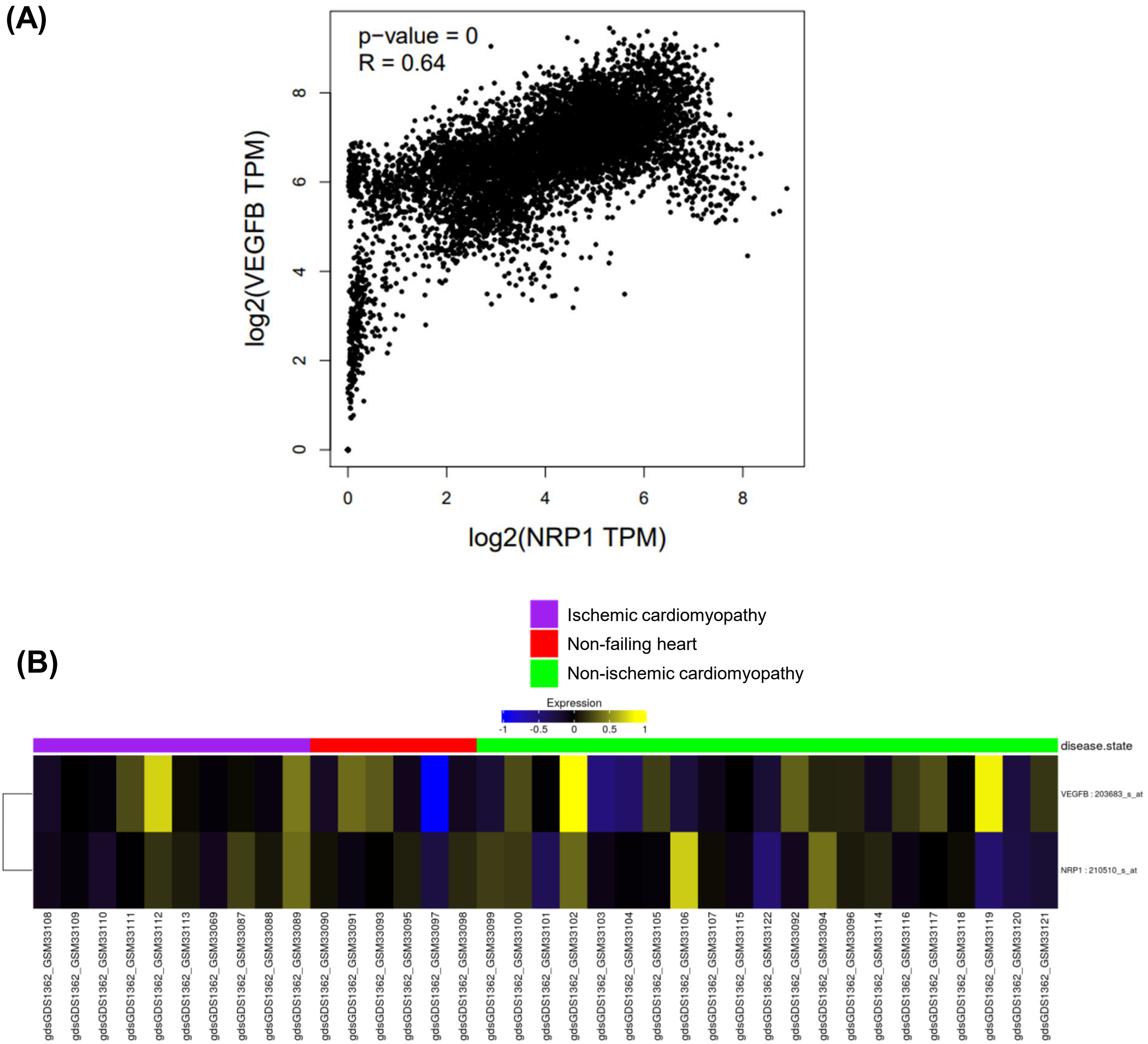

Figure S10. Correlation of NRP1 and VEGF-B. (A) Expression Across Human Tissues from the GTEx Database showing Scatter plot showing the Pearson correlation between NRP1 and VEGFB transcript expression (TPM, transcripts per million reads) across all human tissues, based on data from the GTEx database (https://gtexportal.org/home/). The analysis demonstrates a positive correlation between NRP1 and VEGFB expression, with an R value of 0.64. **(B)** Coexpression analysis in the nonfailing heart and the ischemic heart using Human array data (37 [HG-U133A] Affymetrix Human Genome U133A Array arrays. Ischemic and nonischemic cardiomyopathy comparison. Analysis of myocardial tissues from nonischemic (NICM) and ischemic cardiomyopathy (ICM) patients. NICM and ICM, major forms of dilated cardiomyopathy leading to congestive heart failure, have similar presentations but differ in pathophysiology, prognosis, and response to therapy). The analysis was performed in ILINCs DCIC integrative LINCS genomics data portal | iLINCS.

Table S1. Table showing list of differentially expressed genes between Control and Hypoxia									
Gene_ID	Gene Name	UT1_Normalized.Read.Count	UT2_Normalized.Read.Count	Hyp1_Normalized.Read.Count	Hyp2_Normalized.Read.Count	baseMean	log2FoldChange	pvalue	padj
ENSRNOG00000023116	Agmo	103.19205	87.2287	0	0	47.61	-11.39804	0.00048	0.00175
ENSRNOG00000000905	Tex26	10.319205	17.6534	0	0	6.993	-8.630808	0.01282	0.03176
ENSRNOG00000067279	ENSRNOG0000006727	9.2872841	15.5766	0	0	6.216	-8.460844	0.01549	0.03729
ENSRNOG00000064001	Olr1627	13.414966	9.34593	0	0	5.69	-8.333533	0.01777	0.04212
ENSRNOG00000069204	ENSRNOG0000006920	13.414966	8.3075	0	0	5.431	-8.266186	0.0192	0.045
ENSRNOG00000052026	Gm25777	11.351125	9.34593	0	0	5.174	-8.196367	0.02062	0.04792
ENSRNOG00000032240	Gbp5	12.383045	7.26906	0	0	4.913	-8.121693	0.02253	0.05183
ENSRNOG00000048389	Fgf18	16.510727	3.11531	0	0	4.907	-8.119927	0.02384	0.05438
ENSRNOG00000057077	U2	16.510727	2.07687	0	0	4.647	-8.041523	0.0266	0.05957
ENSRNOG00000042394	4933403O08Rik	7.2234432	10.3844	0	0	4.402	-7.96304	0.02667	0.05972
ENSRNOG00000022640	Siglec8	11.351125	4.15375	0	0	3.876	-7.779811	0.03311	0.07155
ENSRNOG00000007628	Ptp4a3	9.2872841	5.19219	0	0	3.62	-7.681027	0.03625	0.07727
ENSRNOG00000048209	Eps8l3	5.1596023	7.26906	0	0	3.107	-7.460496	0.04545	0.09335
ENSRNOG00000009291	Dnase1l3	9.2872841	3.11531	0	0	3.101	-7.457745	0.04652	0.09516
ENSRNOG00000004778	Cnga1	11.351125	1.03844	0	0	3.097	-7.456332	0.04963	0.1004
ENSRNOG00000004717	Epyc	27.861852	44.6528	1.00388	0	18.38	-6.233136	0.00015	0.00063
ENSRNOG00000014503	Celf4	34.053375	23.8841	0	0.916	14.71	-5.927974	0.00047	0.00172
ENSRNOG00000017918	Iglon5	27.861852	21.8072	0	0.916	12.65	-5.706938	0.00097	0.00324
ENSRNOG00000069561	ENSRNOG0000006956	17.542648	26.9994	1.00388	0	11.39	-5.526015	0.00161	0.00511

ENSRNOG00000049334	Atp2c2	75.330193	56.0756	2.00777	0.916	33.58	-5.510018	7.3E-08	5.6E-07
ENSRNOG00000017484	Gja5	591.29042	541.026	10.0388	16.489	289.7	-5.404234	1.8E-48	5.9E-46
ENSRNOG00000054204	Gria2	17.542648	18.6919	0	0.916	9.288	-5.25469	0.0038	0.01103
ENSRNOG00000053232	Ror2	20.638409	15.5766	0	0.916	9.283	-5.254066	0.00385	0.01116
ENSRNOG00000015972	Ano5	54.691784	47.7681	0	2.7481	26.3	-5.171123	1.7E-06	9.9E-06
ENSRNOG00000004517	Igf1	56.755625	43.6144	1.00388	1.8321	25.8	-5.130695	1.6E-06	9.6E-06
ENSRNOG00000032261	Cyp2d4	12.383045	17.6534	0	0.916	7.738	-4.98603	0.00806	0.02119
ENSRNOG00000048703	Alkal1	284.81005	271.032	1.00388	16.489	143.3	-4.983774	0.00055	0.00197
ENSRNOG00000020980	AABR07006278.1	10.319205	18.6919	0	0.916	7.482	-4.936446	0.0094	0.02421
ENSRNOG00000009892	Adamts15	194.00105	193.149	8.03107	7.3284	100.6	-4.65775	4.7E-20	1.7E-18
ENSRNOG00000051372	Mycn	553.10936	538.949	19.0738	24.733	284	-4.634547	2.9E-46	8.8E-44
ENSRNOG00000015318	Heyl	27.861852	18.6919	1.00388	0.916	12.12	-4.601983	0.00098	0.00327
ENSRNOG00000059225	AABR07020987.1	23.73417	42.5759	1.00388	1.8321	17.29	-4.534028	0.00011	0.00045
ENSRNOG00000013683	S1pr1	42.308739	43.6144	2.00777	1.8321	22.44	-4.485988	8.5E-06	4.4E-05
ENSRNOG00000009726	Grip2	9.2872841	11.4228	0	0.916	5.407	-4.453913	0.02827	0.0627
ENSRNOG00000058340	Krt79	22.70225	17.6534	0	1.8321	10.55	-4.417659	0.00259	0.00782
ENSRNOG00000018864	Klk4	11.351125	9.34593	1.00388	0	5.425	-4.411562	0.02867	0.06342
ENSRNOG00000003018	Olfml2b	1900.7975	1828.69	61.2369	116.34	976.8	-4.386109	2.9E-70	2.9E-67
ENSRNOG00000055886	AABR07051399.1	13.414966	6.23062	0	0.916	5.14	-4.379042	0.03432	0.07375
ENSRNOG00000034038	AABR07005844.1	141.3731	130.843	8.03107	5.4963	71.44	-4.338778	2E-14	4.1E-13
ENSRNOG00000019107	ENSRNOG0000001910	11.351125	8.3075	1.00388	0	5.166	-4.336573	0.03365	0.07257
ENSRNOG00000033844	Bex1	13.414966	5.19219	0	0.916	4.881	-4.301605	0.04078	0.08517

ENSRNOG00000003494	Ppfia4	108.35165	113.19	4.01553	7.3284	58.22	-4.275377	5.4E-12	8E-11
ENSRNOG00000027739	Cndp1	10.319205	8.3075	1.00388	0	4.908	-4.25806	0.03927	0.08256
ENSRNOG00000054458	Kcnmb4	278.61852	192.111	7.02718	17.405	123.8	-4.25276	8E-20	2.9E-18
ENSRNOG00000064728	ENSRNOG0000006472	12.383045	22.8456	0	1.8321	9.265	-4.223647	0.00567	0.01566
ENSRNOG00000070391	ENSRNOG0000007039	22.70225	12.4612	0	1.8321	9.249	-4.221227	0.0057	0.01573
ENSRNOG00000031834	Nkain4	511.83255	510.911	20.0777	35.726	269.6	-4.186427	2.2E-38	3.7E-36
ENSRNOG00000018003	F2rl1	34.053375	34.2684	1.00388	2.7481	18.02	-4.167089	0.0001	0.00043
ENSRNOG00000068036	ENSRNOG0000006803	25.798011	25.9609	2.00777	0.916	13.67	-4.161878	0.00065	0.00227
ENSRNOG00000021433	Arhgef39	569.62009	611.639	18.0699	48.551	312	-4.135664	2.5E-34	3.2E-32
ENSRNOG00000069809	ENSRNOG0000006980	15.478807	18.6919	2.00777	0	9.045	-4.131072	0.00648	0.01756
ENSRNOG00000005998	Smoc1	259.01203	236.764	14.0544	15.573	131.4	-4.062756	6.8E-23	3.4E-21
ENSRNOG00000007957	Tspoap1	17.542648	30.1147	1.00388	1.8321	12.62	-4.058667	0.00126	0.00411
ENSRNOG00000003762	Smarca1	361.17216	407.067	24.0932	23.817	204	-4.003327	6.4E-32	7.1E-30
ENSRNOG00000062881	ENSRNOG0000006288	43.340659	45.6912	1.00388	4.5802	23.65	-3.969992	1.8E-05	8.7E-05
ENSRNOG00000007367	Septin4	21.67033	22.8456	1.00388	1.8321	11.84	-3.960509	0.00179	0.00565
ENSRNOG00000020197	Cd248	171.2988	184.842	12.0466	10.993	94.79	-3.95201	3.8E-17	1E-15
ENSRNOG00000021098	Rasgrp2	33.021455	55.0372	3.01165	2.7481	23.45	-3.936062	1.8E-05	8.9E-05
ENSRNOG00000004327	Ddc	12.383045	16.615	1.00388	0.916	7.729	-3.918614	0.01148	0.02883
ENSRNOG00000067170	ENSRNOG0000006717	314.73574	301.147	4.01553	36.642	164.1	-3.917917	0.00202	0.00629
ENSRNOG00000008591	Colec10	13.414966	14.5381	0	1.8321	7.446	-3.892693	0.01549	0.03729
ENSRNOG00000020407	Atcay	27.861852	43.6144	4.01553	0.916	19.1	-3.881428	0.00014	0.00059
ENSRNOG00000015878	Pif1	481.90685	506.757	17.066	50.383	264	-3.861442	7.3E-27	5.4E-25

ENSRNOG00000004392	Aatk	130.02198	104.882	5.01942	10.993	62.73	-3.861306	2E-11	2.7E-10
ENSRNOG00000016325	F2	17.542648	23.8841	2.00777	0.916	11.09	-3.839363	0.00298	0.00886
ENSRNOG00000023122	ENSRNOG0000002312	17.542648	22.8456	3.01165	0	10.85	-3.782692	0.00484	0.01366
ENSRNOG00000013928	Dsp	133.11774	112.151	9.03495	9.1605	65.87	-3.752496	4.5E-12	6.7E-11
ENSRNOG00000005412	Olr813	58.819466	56.0756	2.00777	6.4123	30.83	-3.751236	2.1E-06	1.2E-05
ENSRNOG00000018674	Ntrk3	43.340659	59.1909	3.01165	4.5802	27.53	-3.747652	5.4E-06	2.9E-05
ENSRNOG00000013589	Cxcl12	3175.2192	3003.16	141.548	325.2	1661	-3.723653	5.1E-43	1.2E-40
ENSRNOG00000056915	Ptprq	607.80115	629.293	42.1631	51.299	332.6	-3.723524	4.6E-42	9.5E-40
ENSRNOG00000014505	Pmfbp1	35.085295	40.499	3.01165	2.7481	20.34	-3.715702	7.9E-05	0.00034
ENSRNOG00000033234	Ccrl2	27.861852	34.2684	2.00777	2.7481	16.72	-3.701552	0.00035	0.0013
ENSRNOG00000008529	Foxs1	127.95814	151.612	6.0233	15.573	75.29	-3.679663	3.1E-12	4.8E-11
ENSRNOG00000005968	Hoxa2	56.755625	64.3831	4.01553	5.4963	32.66	-3.665026	8.9E-07	5.6E-06
ENSRNOG00000007338	Fbln2	9.2872841	14.5381	0	1.8321	6.414	-3.664537	0.02985	0.06557
ENSRNOG00000002794	Selp	583.03506	600.217	47.1825	47.635	319.5	-3.64132	1.5E-40	2.8E-38
ENSRNOG00000037688	Ak9	17.542648	29.0762	0	3.6642	12.57	-3.635061	0.00346	0.01012
ENSRNOG00000028814	Oasl2	11.351125	12.4612	1.00388	0.916	6.433	-3.634298	0.02546	0.05735
ENSRNOG00000028992	Acan	6.1915227	17.6534	2.00777	0	6.463	-3.605393	0.03423	0.07361
ENSRNOG00000016710	ENSRNOG0000001671	5.1596023	17.6534	0	1.8321	6.161	-3.60348	0.03895	0.08202
ENSRNOG00000069091	Paqr4	118.67085	121.497	3.01165	16.489	64.92	-3.601791	1.2E-09	1.2E-08
ENSRNOG00000003442	Adora1	29.925693	39.4606	3.01165	2.7481	18.79	-3.592173	0.0002	0.00079
ENSRNOG00000023257	Adamts9	5996.4898	5828.75	355.375	628.41	3202	-3.585943	3.6E-58	2E-55
ENSRNOG00000026415	Col14a1	18.574568	26.9994	2.00777	1.8321	12.35	-3.570672	0.0025	0.00758

ENSRNOG00000046333	AABR07007032.1	1653.1366	1633.46	147.571	131	891.3	-3.56172	4.4E-71	5E-68
ENSRNOG00000002002	Tmprss11b	307.5123	348.915	14.0544	41.222	177.9	-3.557673	2.8E-19	9.8E-18
ENSRNOG00000022309	Frem1	922.53689	1075.82	64.2485	105.35	542	-3.553757	1.8E-43	4.3E-41
ENSRNOG00000069642	ENSRNOG0000006964	57.787545	41.5375	1.00388	7.3284	26.91	-3.550708	3.4E-05	0.00016
ENSRNOG00000024975	Atp8b3	244.56515	370.722	28.1087	24.733	167	-3.54328	2E-21	8.7E-20
ENSRNOG00000032666	AABR07042915.1	12.383045	9.34593	0	1.8321	5.89	-3.53298	0.04089	0.08535
ENSRNOG00000054438	ENSRNOG0000005443	206.38409	223.264	17.066	21.069	116.9	-3.490539	8.5E-18	2.5E-16
ENSRNOG00000065528	St8sia2	82.553636	109.036	5.01942	11.909	52.13	-3.487279	9.1E-09	8.1E-08
ENSRNOG00000066098	ENSRNOG0000006609	34.053375	39.4606	3.01165	3.6642	20.05	-3.4575	0.00017	0.00067
ENSRNOG00000037645	ENSRNOG0000003764	14.446886	16.615	1.00388	1.8321	8.474	-3.442742	0.0135	0.03314
ENSRNOG00000067650	ENSRNOG0000006765	53.659864	59.1909	3.01165	7.3284	30.8	-3.434043	6E-06	3.2E-05
ENSRNOG00000006142	Mymk	88.745159	52.9603	1.00388	11.909	38.65	-3.432834	7.3E-06	3.8E-05
ENSRNOG00000025725	Ly6k	25.798011	15.5766	2.00777	1.8321	11.3	-3.43134	0.00479	0.01351
ENSRNOG00000067780	ENSRNOG0000006778	14.446886	6.23062	1.00388	0.916	5.649	-3.430743	0.04462	0.09182
ENSRNOG00000018644	Slc6a7	21.67033	31.1531	5.01942	0	14.46	-3.427337	0.00307	0.0091
ENSRNOG00000030238	Fndc5	139.30926	153.689	13.0505	14.657	80.18	-3.400633	7.8E-13	1.3E-11
ENSRNOG00000055293	Ptprb	176.4584	136.035	14.0544	15.573	85.53	-3.397248	3.5E-13	6.1E-12
ENSRNOG00000042269	Gpr21	11.351125	28.0378	1.00388	2.7481	10.79	-3.376415	0.0078	0.02062
ENSRNOG00000008040	Pimreg	1349.752	1367.62	100.388	163.06	745.2	-3.362847	5.6E-46	1.6E-43
ENSRNOG00000013024	Csgalnact1	909.12192	898.248	71.2757	104.43	495.8	-3.358663	1.5E-42	3.4E-40
ENSRNOG00000029911	Cilp	357.04448	482.873	20.0777	61.375	230.3	-3.356728	4.6E-18	1.4E-16
ENSRNOG00000056697	Kcnab1	19.606489	28.0378	0	4.5802	13.06	-3.347914	0.00508	0.01424

ENSRNOG00000053895	B3gnt9	105.25589	99.69	12.0466	8.2444	56.31	-3.342475	2E-09	2E-08
ENSRNOG00000001422	Col26a1	54.691784	50.8834	3.01165	7.3284	28.98	-3.3383	1.6E-05	7.7E-05
ENSRNOG00000028865	Kprp	73.266352	70.6137	6.0233	8.2444	39.54	-3.328871	3.7E-07	2.5E-06
ENSRNOG00000008480	Kcnab3	16.510727	21.8072	2.00777	1.8321	10.54	-3.320399	0.00717	0.01921
ENSRNOG00000033734	Tnnt2	386.97017	414.336	39.1515	41.222	220.4	-3.316817	2.1E-27	1.6E-25
ENSRNOG00000024178	Cenpt	1274.4218	1288.7	55.2136	204.28	705.7	-3.303342	0.00011	0.00048
ENSRNOG00000002470	Ifi47	43.340659	31.1531	4.01553	3.6642	20.54	-3.279591	0.00024	0.00094
ENSRNOG00000004124	Grpr	79.457875	78.9212	9.03495	7.3284	43.69	-3.278261	1.2E-07	9.2E-07
ENSRNOG00000010666	Ccn5	1474.6143	1442.39	113.439	187.79	804.6	-3.272056	9.6E-44	2.4E-41
ENSRNOG00000016752	Crispld2	111.44741	147.458	15.0583	11.909	71.47	-3.266717	7.3E-11	9.2E-10
ENSRNOG00000003585	ENSRNOG0000000358	19.606489	7.26906	0	2.7481	7.406	-3.259311	0.0355	0.07584
ENSRNOG00000058243	Rfx8	41.276818	22.8456	4.01553	2.7481	17.72	-3.251176	0.00081	0.00275
ENSRNOG00000026548	Dhrs7c	31.989534	31.1531	2.00777	4.5802	17.43	-3.247903	0.0008	0.00273
ENSRNOG00000033479	Pcdhb21	49.532182	49.845	4.01553	6.4123	27.45	-3.244954	2.8E-05	0.00013
ENSRNOG00000037299	Smpd5	13.414966	22.8456	2.00777	1.8321	10.03	-3.240675	0.01023	0.02605
ENSRNOG00000015137	Spata6l	13.414966	13.4997	1.00388	1.8321	7.438	-3.236582	0.02554	0.05752
ENSRNOG00000017206	Igfbp5	2320.7891	2447.6	130.505	375.58	1319	-3.235528	3.4E-06	1.9E-05
ENSRNOG00000006426	Syt1	27.861852	16.615	1.00388	3.6642	12.29	-3.234038	0.00554	0.01535
ENSRNOG00000000956	Rasl11a	439.59811	422.644	39.1515	53.131	238.6	-3.220027	7.9E-27	5.8E-25
ENSRNOG00000069593	ENSRNOG0000006959	29.925693	32.1916	3.01165	3.6642	17.2	-3.214711	0.00083	0.00281
ENSRNOG00000020539	Muc1	34.053375	36.3453	4.01553	3.6642	19.52	-3.197908	0.00039	0.00146
ENSRNOG00000023657	Gprin3	443.7258	415.375	48.1864	45.802	238.3	-3.192954	1.5E-27	1.2E-25

ENSRNOG00000039582	Cspg4b	311.63998	331.261	31.1204	39.39	178.4	-3.18544	8.3E-22	3.7E-20
ENSRNOG00000026951	Susd5	32017.396	31265.3	2610.1	4394.3	17572	-3.175263	9.8E-52	3.8E-49
ENSRNOG00000002141	Cd200	1787.2862	1733.15	136.528	255.58	978.1	-3.163361	3.4E-37	5.5E-35
ENSRNOG00000012344	Slc7a9	56.755625	76.8443	3.01165	11.909	37.13	-3.145739	8.8E-06	4.6E-05
ENSRNOG00000032002	Hapln1	15.478807	9.34593	0	2.7481	6.893	-3.145064	0.04431	0.0913
ENSRNOG00000016717	Gas2	16.510727	8.3075	0	2.7481	6.892	-3.144939	0.04532	0.09313
ENSRNOG00000020970	Rundc3a	23.73417	34.2684	2.00777	4.5802	16.15	-3.125895	0.00177	0.00558
ENSRNOG00000014837	Emilin2	1708.8603	1765.34	177.687	223.52	968.9	-3.112567	3.4E-55	1.5E-52
ENSRNOG00000028041	Tnnt1	29.925693	43.6144	2.00777	6.4123	20.49	-3.11081	0.00059	0.00209
ENSRNOG00000030743	Myog	146.5327	86.1903	2.00777	24.733	64.87	-3.105309	3.1E-06	1.7E-05
ENSRNOG00000058609	Palmd	609.86499	575.294	56.2175	82.444	331	-3.091311	7E-30	6.9E-28
ENSRNOG00000008915	Prima1	765.68498	748.713	59.2291	118.17	422.9	-3.088343	5.3E-27	4E-25
ENSRNOG00000006033	Spon2	3872.7975	3597.15	407.577	470.85	2087	-3.087422	3.6E-72	4.9E-69
ENSRNOG00000024428	Kif20a	5203.9749	5141.3	451.748	767.65	2891	-3.083586	1.6E-45	4.4E-43
ENSRNOG00000016456	Il33	18.574568	21.8072	2.00777	2.7481	11.28	-3.080873	0.00814	0.02137
ENSRNOG00000048369	ENSRNOG0000004836	12.383045	36.3453	4.01553	1.8321	13.64	-3.06963	0.00598	0.01642
ENSRNOG00000047143	Fkbp1b	79.457875	79.9597	9.03495	10.077	44.63	-3.058628	3.4E-07	2.3E-06
ENSRNOG00000047653	Crybb1	13.414966	35.3069	5.01942	0.916	13.66	-3.056688	0.00714	0.01913
ENSRNOG00000062707	ENSRNOG0000006270	13.414966	10.3844	2.00777	0.916	6.681	-3.037014	0.04296	0.08909
ENSRNOG00000012879	Fabp3	51.596023	34.2684	5.01942	5.4963	24.1	-3.028225	0.00019	0.00076
ENSRNOG00000039336	Hrct1	410.70434	396.683	45.1748	54.047	226.7	-3.022295	2.6E-24	1.5E-22
ENSRNOG00000007882	Ablim2	405.54474	381.106	34.132	62.291	220.8	-3.021873	1.7E-20	6.5E-19

ENSRNOG00000065502	U1	17.542648	20.7687	1.00388	3.6642	10.74	-3.019491	0.01237	0.03079
ENSRNOG00000049383	Krtap3-3	71.202511	92.4209	12.0466	8.2444	45.98	-3.016914	4.8E-07	3.1E-06
ENSRNOG00000015567	Slc9a2	71.202511	59.1909	6.0233	10.077	36.62	-3.010343	6E-06	3.2E-05
ENSRNOG00000004861	Itga4	1808.9566	1863.99	214.831	241.84	1032	-3.006851	2.9E-57	1.5E-54
ENSRNOG00000046598	AABR07029742.2	37.149136	38.4222	3.01165	6.4123	21.25	-2.992593	0.00051	0.00182
ENSRNOG00000001876	Ccdc74a	17.542648	26.9994	0	5.4963	12.51	-2.991906	0.01276	0.03166
ENSRNOG00000034190	Ighm	101.1282	95.5362	9.03495	15.573	55.32	-2.990964	5.3E-08	4.2E-07
ENSRNOG00000054548	Srpk3	26.829932	17.6534	0	5.4963	12.49	-2.990168	0.01281	0.03176
ENSRNOG00000005277	Ptprv	265.20356	271.032	25.0971	42.138	150.9	-2.989275	1.6E-16	4E-15
ENSRNOG00000002092	Hunk	22.70225	37.3837	2.00777	5.4963	16.9	-2.987579	0.00226	0.00694
ENSRNOG00000007014	ENSRNOG0000000701	203.28833	266.878	31.1204	28.398	132.4	-2.98292	1.9E-15	4.3E-14
ENSRNOG00000039593	Ecscr	37.149136	45.6912	4.01553	6.4123	23.32	-2.982919	0.00027	0.00102
ENSRNOG00000046280	Tceal5	54.691784	49.845	3.01165	10.077	29.41	-2.982537	8.5E-05	0.00037
ENSRNOG00000011346	Ehd2	7468.0083	7565.01	650.517	1251.3	4234	-2.981897	2.1E-36	3E-34
ENSRNOG00000065101	ENSRNOG0000006510	17.542648	29.0762	6.0233	0	13.16	-2.978552	0.01228	0.03058
ENSRNOG00000039544	ENSRNOG0000003954	103.19205	129.805	14.0544	15.573	65.66	-2.9738	3.3E-09	3.2E-08
ENSRNOG00000020981	Prss46	56.755625	42.5759	10.0388	2.7481	28.03	-2.973511	0.00015	0.00062
ENSRNOG00000021200	Hjv	212.57561	242.994	11.0427	46.719	128.3	-2.96841	3E-10	3.4E-09
ENSRNOG00000027894	Iqgap3	2643.7802	2641.78	186.722	489.17	1490	-2.966768	3.9E-06	2.2E-05
ENSRNOG00000049614	AABR07028488.1	1439.529	1391.51	121.47	240	798.1	-2.966117	1.1E-29	1E-27
ENSRNOG00000023126	Rxfp3	91.84092	91.3825	5.01942	18.321	51.64	-2.958289	1E-06	6.5E-06
ENSRNOG00000061764	AABR07021812.1	70.170591	69.5753	7.02718	10.993	39.44	-2.948732	3.4E-06	1.9E-05

ENSRNOG00000010841	Col8a2	1144.3998	1031.17	113.439	168.55	614.4	-2.944707	5.8E-36	7.8E-34
ENSRNOG00000008758	Tspan18	533.50288	552.449	64.2485	76.948	306.8	-2.941141	1.3E-28	1.2E-26
ENSRNOG00000013463	Kcnj8	602.64155	559.718	62.2408	88.857	328.4	-2.939867	1.1E-27	8.8E-26
ENSRNOG00000006653	Slc38a4	1499.3804	1427.85	191.742	190.54	827.4	-2.936891	3.2E-51	1.2E-48
ENSRNOG00000033064	Edil3	595.4181	584.64	57.2214	97.101	333.6	-2.930003	3.3E-25	2.1E-23
ENSRNOG00000068914	Nrep	2842.9409	3005.24	268.037	499.25	1654	-2.928416	2E-34	2.6E-32
ENSRNOG00000018815	Plk1	5469.1784	5367.68	545.109	889.48	3068	-2.916267	1.5E-43	3.7E-41
ENSRNOG00000007202	Sema3d	5555.8597	5420.64	512.985	945.36	3109	-2.911031	2.4E-36	3.3E-34
ENSRNOG00000010716	Atoh8	953.4945	802.712	40.1553	194.2	497.6	-2.904947	0.00392	0.01134
ENSRNOG00000021410	Negr1	552.07744	494.296	67.2602	73.284	296.7	-2.895357	2.1E-27	1.7E-25
ENSRNOG00000002272	Lnx1	97.000523	125.651	4.01553	25.649	63.08	-2.89315	2.3E-06	1.3E-05
ENSRNOG00000004610	Lum	746.07849	779.866	110.427	96.185	433.1	-2.886086	1.1E-34	1.5E-32
ENSRNOG00000012645	Mecom	426.18315	388.375	43.167	66.872	231.1	-2.883338	5.8E-21	2.4E-19
ENSRNOG00000010302	Rasl10b	44.37258	73.729	3.01165	12.825	33.48	-2.883243	0.00011	0.00045
ENSRNOG00000012920	Col9a1	126.92622	134.997	20.0777	15.573	74.39	-2.880632	8.3E-10	8.8E-09
ENSRNOG00000067579	ENSRNOG0000006757	25.798011	29.0762	0	7.3284	15.55	-2.879903	0.00844	0.02206
ENSRNOG00000025448	Limd2	267.2674	293.878	29.1126	47.635	159.5	-2.864537	4.4E-16	1.1E-14
ENSRNOG00000005772	Hacd4	286.87389	292.839	12.0466	67.788	164.9	-2.858257	0.00725	0.01937
ENSRNOG00000021644	Slc15a3	10.319205	23.8841	1.00388	3.6642	9.718	-2.857081	0.02492	0.05639
ENSRNOG00000032596	RT1-T24-1	846.17477	876.441	70.2718	167.64	490.1	-2.851653	1.4E-20	5.6E-19
ENSRNOG00000057501	Fam81a	438.56619	428.875	51.1981	68.704	246.8	-2.851646	6.6E-23	3.3E-21
ENSRNOG00000006410	Akap5	71.202511	52.9603	8.03107	9.1605	35.34	-2.850661	1.8E-05	8.7E-05

ENSRNOG00000047977	Tcim	5.1596023	22.8456	3.01165	0.916	7.983	-2.848276	0.04671	0.09543
ENSRNOG00000063216	Ccnb2	1447.7844	1510.93	161.625	250.08	842.6	-2.842779	2E-36	2.9E-34
ENSRNOG00000037082	Mybphl	98.032443	78.9212	8.03107	16.489	50.37	-2.842271	8.9E-07	5.6E-06
ENSRNOG00000004351	Slc25a29	19.606489	29.0762	5.01942	1.8321	13.88	-2.842165	0.00699	0.01877
ENSRNOG00000043199	Bves	296.16117	311.531	34.132	50.383	173.1	-2.84153	2E-17	5.7E-16
ENSRNOG00000016177	Scara3	684.16326	691.599	87.3379	104.43	391.9	-2.840997	1.4E-31	1.5E-29
ENSRNOG00000016149	Hoxc10	13.414966	20.7687	2.00777	2.7481	9.735	-2.840795	0.02077	0.0482
ENSRNOG00000058539	Ccnb1	4046.1601	3800.68	400.55	696.2	2236	-2.837639	1.6E-36	2.3E-34
ENSRNOG00000014686	Kcnd3	177.49032	227.418	17.066	39.39	115.3	-2.833777	3.8E-11	4.9E-10
ENSRNOG00000025781	Sec31b	14.446886	12.4612	1.00388	2.7481	7.665	-2.82868	0.04084	0.08526
ENSRNOG00000066444	ENSRNOG0000006644	45.4045	68.5369	4.01553	11.909	32.47	-2.826109	9.9E-05	0.00042
ENSRNOG00000004865	Pkd1l1	92.872841	105.921	19.0738	9.1605	56.76	-2.824912	2.5E-07	1.7E-06
ENSRNOG00000062986	Ccbe1	201.22449	200.418	33.1282	23.817	114.6	-2.822451	4.2E-13	7.3E-12
ENSRNOG00000023337	Sema3a	1252.7514	1258.59	84.3262	271.15	716.7	-2.819944	0.00018	0.00073
ENSRNOG00000013515	Ptpru	36.117216	23.8841	2.00777	6.4123	17.11	-2.818827	0.0034	0.00999
ENSRNOG00000028526	Mansc1	63.979068	75.8059	5.01942	14.657	39.87	-2.816071	1.7E-05	8.6E-05
ENSRNOG00000070523	Mxd3	426.18315	426.798	40.1553	81.528	243.7	-2.803266	7.3E-18	2.2E-16
ENSRNOG00000018598	Ankrd1	53077.861	52008	6654.74	8484.4	30056	-2.79513	6.5E-66	5.4E-63
ENSRNOG00000018874	Phf19	259.01203	223.264	20.0777	49.467	138	-2.785476	7.1E-12	1E-10
ENSRNOG00000001963	Mx2	20.638409	19.7303	5.01942	0.916	11.58	-2.784973	0.01642	0.03923
ENSRNOG00000004699	Fibin	347.75719	274.147	22.0854	67.788	177.9	-2.782774	9.4E-12	1.3E-10
ENSRNOG00000058654	Mex3a	289.96965	279.34	33.1282	49.467	163	-2.780504	3.8E-16	9.3E-15

ENSRNOG0000 0002396	Serpinb8	36.117216	30.1147	6.0233	3.6642	18.98	-2.780321	0.00171	0.00541
ENSRNOG0000 0033528	Tll1	5307.1669	5434.14	709.746	856.51	3077	-2.777236	4.3E-62	2.8E-59
ENSRNOG0000 0009198	Rab6b	1075.2611	1144.36	103.4	219.85	635.7	-2.776097	5.1E-23	2.6E-21
ENSRNOG0000 0067027	ENSRNOG0 000006702	20.638409	18.6919	3.01165	2.7481	11.27	-2.772907	0.01409	0.03436
ENSRNOG0000 0018101	Klc3	80.489795	89.3056	14.0544	10.993	48.71	-2.764416	9.5E-07	5.9E-06
ENSRNOG0000 0070452	ENSRNOG0 000007045	50.564102	38.4222	2.00777	10.993	25.5	-2.758122	0.00086	0.0029
ENSRNOG0000 0043151	Cntln	636.69492	694.714	45.1748	152.06	382.2	-2.75382	0.00059	0.00209
ENSRNOG0000 0015691	Inka2	43.340659	39.4606	5.01942	7.3284	23.79	-2.740189	0.00053	0.00188
ENSRNOG0000 0020948	Pth1r	126.92622	145.381	14.0544	26.565	78.23	-2.737432	6.6E-09	6.1E-08
ENSRNOG0000 0010350	Rcan2	768.78074	693.676	85.3301	133.74	420.4	-2.735394	6.8E-26	4.6E-24
ENSRNOG0000 0013552	Scd	357.04448	510.911	52.2019	77.864	249.5	-2.734751	3E-18	9.6E-17
ENSRNOG0000 0015333	AABR07070 225.1	23.73417	20.7687	3.01165	3.6642	12.79	-2.734104	0.00991	0.02535
ENSRNOG0000 0010183	Gask1b	9546.2961	9278.44	989.829	1841.3	5414	-2.732678	1.6E-32	1.9E-30
ENSRNOG0000 0015131	Ube2c	2294.9911	2301.18	320.239	377.41	1323	-2.718974	6.7E-51	2.4E-48
ENSRNOG0000 0001827	Masp1	3602.4343	3439.3	409.584	660.47	2028	-2.717022	4.1E-37	6.5E-35
ENSRNOG0000 0010744	Nrp1	1298.1559	1300.12	143.555	251.91	748.4	-2.713116	3.6E-28	3.1E-26
ENSRNOG0000 0069437	ENSRNOG0 000006943	171.2988	179.65	14.0544	39.39	101.1	-2.705504	3.4E-09	3.2E-08
ENSRNOG0000 0020875	Celf3	121.76661	127.728	19.0738	19.237	71.95	-2.703059	6.9E-09	6.3E-08
ENSRNOG0000 0016997	Bnip5	38.181057	23.8841	4.01553	5.4963	17.89	-2.701826	0.00306	0.00907
ENSRNOG0000 0009656	Rspo1	2645.844	2502.63	271.049	521.23	1485	-2.698395	7.1E-28	6.1E-26
ENSRNOG0000 0014243	Pear1	167.17111	141.227	18.0699	29.314	88.95	-2.696627	8.7E-10	9.2E-09
ENSRNOG0000 0020355	Twist2	53.659864	55.0372	3.01165	13.741	31.36	-2.682679	0.00032	0.0012

ENSRNOG00000052424	RGD1306556	65.010989	63.3447	9.03495	10.993	37.1	-2.677447	2.4E-05	0.00012
ENSRNOG00000011718	C1rl	121.76661	202.495	26.101	24.733	93.77	-2.673803	1.5E-09	1.5E-08
ENSRNOG00000001514	Cdca7	376.65097	339.569	37.1437	75.116	207.1	-2.667535	4.8E-15	1E-13
ENSRNOG00000005291	Slc38a1	275.52276	242.994	31.1204	50.383	150	-2.664346	8.9E-14	1.7E-12
ENSRNOG00000009331	Hck	54.691784	41.5375	5.01942	10.077	27.83	-2.66362	0.00034	0.00128
ENSRNOG00000014080	Kif23	4343.3532	4301.21	540.089	827.19	2503	-2.659523	2E-39	3.6E-37
ENSRNOG00000032951	Jsrp1	566.52433	549.333	67.2602	109.01	323	-2.658245	1.5E-21	6.5E-20
ENSRNOG00000042912	Mycbpap	220.83098	264.801	30.1165	46.719	140.6	-2.655234	3.7E-13	6.3E-12
ENSRNOG00000006508	Thnsl2	40.244898	61.2678	5.01942	10.993	29.38	-2.654894	0.00031	0.00116
ENSRNOG00000018858	Myct1	13.414966	16.615	2.00777	2.7481	8.696	-2.654166	0.03671	0.07805
ENSRNOG00000060087	Adra1b	85.649398	56.0756	4.01553	18.321	41.02	-2.65194	0.00011	0.00045
ENSRNOG00000008949	Synpo2l	1207.3469	1173.43	109.423	269.32	689.9	-2.649078	1.7E-18	5.4E-17
ENSRNOG00000011729	Nxf2	160.97959	166.15	24.0932	28.398	94.91	-2.637675	1.5E-10	1.8E-09
ENSRNOG00000014835	Il1rl1	1825.4673	1965.76	289.118	321.53	1100	-2.633615	3.9E-46	1.2E-43
ENSRNOG00000042445	AC130970.1	128.99006	181.726	25.0971	25.649	90.37	-2.613891	1.4E-09	1.5E-08
ENSRNOG00000004118	Frmpd4	1693.3815	1902.42	235.913	351.76	1046	-2.611437	1.8E-33	2.3E-31
ENSRNOG00000016627	Eya4	1844.0419	1946.03	206.8	415.89	1103	-2.60361	9E-24	4.9E-22
ENSRNOG00000008869	Ppp1r9a	17.542648	17.6534	4.01553	1.8321	10.26	-2.599688	0.02715	0.06064
ENSRNOG00000009334	Knstrn	1799.6693	1688.5	219.851	358.18	1017	-2.591277	5.8E-30	5.8E-28
ENSRNOG00000025584	Agap2	29.925693	15.5766	2.00777	5.4963	13.25	-2.588469	0.01548	0.03728
ENSRNOG00000027125	Mboat1	1102.091	1063.36	152.59	207.03	631.3	-2.58806	1.1E-31	1.2E-29
ENSRNOG00000014293	Nkd1	37.149136	25.9609	5.01942	5.4963	18.41	-2.584198	0.0035	0.01023

ENSRNOG00000047940	Mmp28	1005.0905	987.554	124.482	207.94	581.3	-2.580617	3.5E-25	2.2E-23
ENSRNOG00000057284	Cenpb	810.05756	757.021	82.3185	179.55	457.2	-2.57727	5.1E-18	1.5E-16
ENSRNOG00000003345	AABR07038690.1	118.67085	78.9212	11.0427	21.985	57.65	-2.57332	2.6E-06	1.5E-05
ENSRNOG00000022411	RGD1561958	463.33228	451.72	61.2369	92.521	267.2	-2.569453	3.2E-19	1.1E-17
ENSRNOG00000007896	Klhl38	72.234432	105.921	9.03495	21.069	52.06	-2.556063	9.5E-06	4.9E-05
ENSRNOG00000014722	Raph1	4847.9623	5325.11	646.501	1085.5	2976	-2.553427	8.6E-32	9.4E-30
ENSRNOG00000027259	Faxdc2	125.8943	110.074	21.0816	19.237	69.07	-2.550245	6.6E-08	5.1E-07
ENSRNOG00000062276	AABR07044631.2	99.064364	100.728	11.0427	22.901	58.43	-2.549114	1.6E-06	9.5E-06
ENSRNOG00000017307	Prss23	4061.6389	3979.29	534.066	840.93	2354	-2.546967	1.4E-34	1.8E-32
ENSRNOG00000000897	Rxfp2	91.84092	68.5369	8.03107	19.237	46.91	-2.546817	2.1E-05	0.0001
ENSRNOG00000027109	Odf3l1	105.25589	115.267	10.0388	27.481	64.51	-2.545222	1.7E-06	9.9E-06
ENSRNOG00000015357	Bmper	4874.7922	4690.62	627.427	1010.4	2801	-2.545207	7.4E-34	9.3E-32
ENSRNOG00000015125	Spsb2	633.59916	610.601	104.404	109.01	364.4	-2.543094	5.7E-26	3.9E-24
ENSRNOG00000052498	Grb14	4898.5264	4865.08	583.256	1091	2859	-2.543029	2.4E-27	1.8E-25
ENSRNOG00000007707	Zfp467	183.68184	142.266	19.0738	36.642	95.42	-2.541851	7.1E-09	6.5E-08
ENSRNOG00000071012	ENSRNOG0000007101	171.2988	187.957	24.0932	37.558	105.2	-2.53787	3.1E-10	3.5E-09
ENSRNOG00000066109	Plekha2	138.27734	166.15	23.0893	29.314	89.21	-2.535533	2.6E-09	2.6E-08
ENSRNOG00000019549	Akap12	9139.7195	9445.62	1661.43	1548.1	5449	-2.533845	4E-63	2.8E-60
ENSRNOG00000020519	Olfm2	1827.5311	1763.27	289.118	331.61	1053	-2.531493	3.9E-42	8E-40
ENSRNOG00000019609	B4galt2	791.48299	698.868	102.396	155.73	437.1	-2.526565	3.2E-23	1.6E-21
ENSRNOG00000006956	Adamtsl1	26.829932	28.0378	4.01553	5.4963	16.09	-2.524031	0.00693	0.01863
ENSRNOG00000017959	Cfap100	234.24594	299.07	20.0777	72.368	156.4	-2.520765	1.2E-08	1E-07

ENSRNOG00000062702	ENSRNOG0000006270	15.478807	22.8456	3.01165	3.6642	11.25	-2.518545	0.02384	0.05438
ENSRNOG00000007377	Slit3	89.77708	122.536	15.0583	21.985	62.34	-2.514435	7.3E-07	4.7E-06
ENSRNOG00000059894	Hmmr	1924.5316	2002.11	277.072	409.47	1153	-2.514275	5.8E-33	6.9E-31
ENSRNOG00000004613	Gpm6b	2033.9152	2105.95	343.328	381.08	1216	-2.514162	1.3E-44	3.4E-42
ENSRNOG00000061714	ENSRNOG0000006171	36.117216	29.0762	5.01942	6.4123	19.16	-2.508553	0.00356	0.01038
ENSRNOG00000014021	Matn4	501.51334	458.989	94.3651	75.116	282.5	-2.50483	1.6E-20	6.3E-19
ENSRNOG00000063664	Timm17b	799.73835	726.906	105.408	163.06	448.8	-2.50458	5.3E-23	2.6E-21
ENSRNOG00000018606	Olr59	104.22397	141.227	24.0932	19.237	72.2	-2.504559	1.1E-07	8.1E-07
ENSRNOG00000053047	Top2a	10166.48	10269.1	932.608	2689.5	6014	-2.496097	0.00036	0.00133
ENSRNOG00000070326	ENSRNOG0000007032	73.266352	55.0372	8.03107	14.657	37.75	-2.492598	9.6E-05	0.00041
ENSRNOG00000017149	Fam131b	324.02302	273.109	24.0932	81.528	175.7	-2.492164	3.3E-09	3.2E-08
ENSRNOG00000010079	Ca3	35957.268	35484.4	5071.62	7661.8	21044	-2.488027	7.8E-39	1.4E-36
ENSRNOG00000020676	Ppp1r14a	21.67033	25.9609	2.00777	6.4123	14.01	-2.486915	0.01522	0.03672
ENSRNOG00000064828	ENSRNOG0000006482	460.23652	463.143	66.2563	99.849	272.4	-2.471278	3.2E-18	1E-16
ENSRNOG00000021781	Camk1	1535.4976	1505.73	205.796	343.52	897.6	-2.466871	5.8E-26	3.9E-24
ENSRNOG00000032410	Mtus2	2536.4605	2768.47	315.219	643.98	1566	-2.466036	8.4E-22	3.7E-20
ENSRNOG00000028415	Cdc20	3034.8781	2995.89	343.328	748.41	1781	-2.464466	1.6E-20	6.2E-19
ENSRNOG00000020164	Ifitm10	45.4045	62.3062	16.0621	3.6642	31.86	-2.462353	0.00091	0.00307
ENSRNOG00000049052	Sgk3	836.88749	822.442	87.3379	213.44	490	-2.460275	3.4E-15	7.5E-14
ENSRNOG00000054218	Il18rap	1480.8059	1463.16	225.874	309.62	869.9	-2.457149	4.1E-32	4.7E-30
ENSRNOG00000048924	Islr	146.5327	146.42	22.0854	31.146	86.55	-2.456404	1E-08	9.3E-08
ENSRNOG00000038480	Ppp1r36	121.76661	115.267	9.03495	33.894	69.99	-2.454512	6.4E-06	3.4E-05

ENSRNOG00000001193	Hsf2bp	38.181057	69.5753	3.01165	16.489	31.81	-2.452473	0.00149	0.00477
ENSRNOG00000049695	Myh4	4473.3752	4653.24	567.194	1101.1	2699	-2.450862	4E-24	2.2E-22
ENSRNOG00000037211	Kif14	2139.1711	2060.26	176.684	593.6	1242	-2.446424	0.00169	0.00536
ENSRNOG00000015353	Prss12	100.09628	87.2287	16.0621	18.321	55.43	-2.444206	2.4E-06	1.4E-05
ENSRNOG00000008034	Tmeff1	449.91732	433.028	70.2718	92.521	261.4	-2.436749	1.6E-18	5.2E-17
ENSRNOG00000003388	Cenpf	4902.6541	5102.88	493.911	1353.9	2963	-2.436742	0.00028	0.00108
ENSRNOG00000060687	Slc24a3	854.43014	804.789	111.431	196.03	491.7	-2.42887	3.5E-20	1.3E-18
ENSRNOG00000017328	Pter	104.22397	114.228	11.0427	29.314	64.7	-2.427179	4E-06	2.2E-05
ENSRNOG00000068207	ENSRNOG0000006820	1750.1371	1671.88	239.928	395.73	1014	-2.426681	4E-26	2.8E-24
ENSRNOG00000008609	Capn3	47.468341	50.8834	11.0427	7.3284	29.18	-2.425521	0.00055	0.00197
ENSRNOG00000045560	Gvin1	301.32077	331.261	43.167	74.2	187.5	-2.425446	3E-13	5.2E-12
ENSRNOG00000057794	Adamts5	1016.4416	997.938	120.466	253.75	597.1	-2.425431	8.8E-18	2.6E-16
ENSRNOG00000002004	Prdm8	897.7708	869.172	100.388	228.1	523.9	-2.424056	5.1E-16	1.2E-14
ENSRNOG00000003533	Clcn4	74.298273	64.3831	13.0505	12.825	41.14	-2.422381	4.4E-05	0.0002
ENSRNOG00000024365	Ect2	3603.4662	3672.95	450.744	911.47	2160	-2.416254	5.5E-22	2.5E-20
ENSRNOG00000033527	Pappa1	93.904761	97.6131	11.0427	24.733	56.82	-2.412062	7.8E-06	4.1E-05
ENSRNOG00000024677	Arhgap33	1212.5065	1310.51	163.633	310.54	749.3	-2.409231	1.9E-20	7.3E-19
ENSRNOG00000066309	ENSRNOG0000006630	40.244898	25.9609	7.02718	5.4963	19.68	-2.405456	0.00473	0.01336
ENSRNOG00000002548	Tnn	307.5123	358.261	81.3146	44.886	198	-2.404157	6.4E-13	1.1E-11
ENSRNOG00000019100	Kif2c	2142.2669	2023.91	291.126	495.58	1238	-2.403246	1.7E-25	1.1E-23
ENSRNOG00000004921	Nusap1	2040.1067	1997.95	276.068	487.34	1200	-2.401492	2.9E-24	1.6E-22
ENSRNOG00000000858	Sapcd1	240.43747	225.341	36.1398	52.215	138.5	-2.394518	1.5E-11	2.1E-10

ENSRNOG00000020009	Npas4	275.52276	322.954	44.1709	69.62	178.1	-2.390744	6.7E-13	1.1E-11
ENSRNOG00000020028	Pcdhb22	23.73417	20.7687	2.00777	6.4123	13.23	-2.389381	0.02228	0.05131
ENSRNOG00000010691	Cmtm3	1172.2616	1142.28	187.726	253.75	689	-2.388557	1.7E-28	1.5E-26
ENSRNOG00000045829	Thbs1	295955.82	294821	41674.2	72496	2E+05	-2.37141	2E-28	1.8E-26
ENSRNOG00000013774	Lmnb1	3097.8252	3129.85	397.538	806.12	1858	-2.370124	6.9E-21	2.8E-19
ENSRNOG00000001750	Chrd	153.75615	118.382	26.101	26.565	81.2	-2.369242	7E-08	5.4E-07
ENSRNOG00000022537	E2f8	1053.5908	1263.78	93.3612	356.34	691.8	-2.364996	0.00753	0.02002
ENSRNOG00000013925	Nox4	490.16222	550.372	79.3068	122.75	310.6	-2.361177	1.8E-17	5.3E-16
ENSRNOG00000062541	ENSRNOG0000006254	1608.764	1456.93	201.781	394.82	915.6	-2.359357	1.2E-19	4.3E-18
ENSRNOG00000046428	Lrrc75b	1204.2512	1014.55	141.548	290.39	662.7	-2.358333	3E-17	8.3E-16
ENSRNOG00000017550	Fbln7	54.691784	72.6906	12.0466	12.825	38.06	-2.355797	0.00014	0.00056
ENSRNOG00000039902	Lbh	1963.7446	1914.88	309.196	449.78	1159	-2.351996	9.5E-30	9.3E-28
ENSRNOG00000003170	Nlrp3	412.76818	443.413	84.3262	85.193	256.4	-2.33636	5.1E-18	1.6E-16
ENSRNOG00000005798	Cav3	239.40555	262.725	51.1981	48.551	150.5	-2.332229	2.2E-12	3.4E-11
ENSRNOG00000009503	Depdc1	1023.6651	1119.44	165.641	261.99	642.7	-2.322972	2.4E-22	1.1E-20
ENSRNOG00000015410	Aspn	139.30926	149.535	33.1282	24.733	86.68	-2.322802	4.4E-08	3.5E-07
ENSRNOG00000028344	Mmp11	594.38618	510.911	58.2252	162.14	331.4	-2.322202	4.7E-11	6E-10
ENSRNOG00000003794	Nmral1	605.73731	525.449	79.3068	146.57	339.3	-2.320527	2.5E-15	5.4E-14
ENSRNOG00000031743	Gbp2	718.21664	844.249	162.629	151.15	469.1	-2.316533	2.1E-24	1.2E-22
ENSRNOG00000065666	ENSRNOG0000006566	39.212977	41.5375	7.02718	9.1605	24.23	-2.315364	0.00223	0.00687
ENSRNOG00000032443	Lmod3	1098.9953	1058.17	151.586	282.14	647.7	-2.311723	8.1E-19	2.7E-17
ENSRNOG00000006731	Spc25	1639.7216	1530.66	203.788	434.21	952.1	-2.311096	2.2E-17	6.3E-16

ENSRNOG00000066242	Rtp4	80.489795	64.3831	7.02718	21.985	43.47	-2.309744	0.00024	0.00092
ENSRNOG00000049949	Hapln4	271.39508	272.071	46.1786	63.207	163.2	-2.309698	2.1E-12	3.2E-11
ENSRNOG00000001469	Eln	185.74568	238.841	37.1437	48.551	127.6	-2.306077	3.7E-10	4.1E-09
ENSRNOG00000027035	Sgo2	2245.4589	2317.79	366.418	557.87	1372	-2.302359	8.3E-28	6.9E-26
ENSRNOG00000000547	Tspyl4	134.14966	114.228	7.02718	43.054	74.61	-2.300247	9.1E-05	0.00039
ENSRNOG00000002161	Tlr6	391.09785	424.721	72.2796	93.437	245.4	-2.297233	3.6E-16	8.7E-15
ENSRNOG00000064360	U2	33.021455	28.0378	6.0233	6.4123	18.37	-2.295005	0.00778	0.02057
ENSRNOG00000024751	Macroh2a2	737.82313	720.675	98.3806	198.78	438.9	-2.291785	2.1E-15	4.7E-14
ENSRNOG00000015406	Pgm5	8111.9267	8196.38	1451.62	1879.7	4910	-2.291086	5.8E-41	1.1E-38
ENSRNOG00000015423	Ccna2	4629.1952	4639.74	595.303	1298	2791	-2.290719	2E-18	6.3E-17
ENSRNOG00000070856	ENSRNOG0000007085	24.766091	12.4612	3.01165	4.5802	11.2	-2.289066	0.04022	0.08423
ENSRNOG00000008543	Pdlim2	601.60963	591.909	89.3456	154.81	359.4	-2.285888	2.1E-16	5.1E-15
ENSRNOG00000030880	Hs6st2	30.957614	19.7303	3.01165	7.3284	15.26	-2.283857	0.0187	0.04404
ENSRNOG00000037225	Tyms	2151.5541	1955.38	280.084	566.12	1238	-2.277468	1.7E-18	5.4E-17
ENSRNOG00000029535	Nrbp2	1829.595	2153.72	297.15	523.98	1201	-2.276798	3E-21	1.3E-19
ENSRNOG00000003538	Adamts4	774.97226	667.715	108.419	189.62	435.2	-2.272157	7.1E-17	1.9E-15
ENSRNOG00000012149	Gpsm2	1170.1978	1151.63	173.672	307.79	700.8	-2.267455	1.5E-19	5.5E-18
ENSRNOG00000006970	Ntn3	170.26688	146.42	17.066	48.551	95.58	-2.263067	1.3E-06	8.2E-06
ENSRNOG00000014034	Olfml2a	324.02302	341.646	74.2874	65.04	201.2	-2.257596	2.3E-14	4.6E-13
ENSRNOG00000005465	Kcnmb1	101.1282	83.075	8.03107	30.23	55.62	-2.25718	0.00013	0.00053
ENSRNOG00000049942	RGD1564899	586.13082	549.333	69.268	167.64	343.1	-2.25688	6.3E-12	9.1E-11
ENSRNOG00000028781	Abcc6	88.745159	103.844	9.03495	31.146	58.19	-2.251105	8E-05	0.00035

ENSRNOG00000002381	Bmp3	327.11878	291.801	36.1398	93.437	187.1	-2.250235	3.2E-09	3.1E-08
ENSRNOG00000051615	Hmgn2	3000.8247	2815.2	425.647	798.8	1760	-2.246808	1.2E-20	4.8E-19
ENSRNOG00000018384	Adam12	23089.22	22798.9	4536.55	5144.5	13892	-2.244804	7.6E-51	2.7E-48
ENSRNOG00000032178	Cenpa	2692.2805	2734.2	519.008	627.49	1643	-2.242111	6.8E-37	1.1E-34
ENSRNOG00000050312	Ism2	218.76714	206.649	29.1126	60.459	128.7	-2.241785	1.1E-08	9.9E-08
ENSRNOG00000049033	Racgap1	7119.2192	7039.57	973.767	2021.7	4289	-2.240347	3.8E-19	1.3E-17
ENSRNOG00000066647	ENSRNOG0000006664	316.79958	489.104	48.1864	122.75	244.2	-2.232652	3E-09	2.9E-08
ENSRNOG00000001229	Col18a1	989.61172	1017.67	211.819	215.27	608.6	-2.232512	8.1E-28	6.7E-26
ENSRNOG00000010473	Cand2	490.16222	473.527	66.2563	138.32	292.1	-2.231701	1.7E-12	2.7E-11
ENSRNOG00000030522	ENSRNOG0000003052	57.787545	28.0378	11.0427	7.3284	26.05	-2.228492	0.00352	0.01027
ENSRNOG00000014398	Scara5	146.5327	121.497	24.0932	32.978	81.28	-2.228332	3.7E-07	2.5E-06
ENSRNOG00000019869	Lrfn1	105.25589	103.844	17.066	27.481	63.41	-2.225781	5.9E-06	3.2E-05
ENSRNOG00000002555	Serpinb7	165.10727	119.42	38.1476	22.901	86.39	-2.225448	5.8E-07	3.7E-06
ENSRNOG00000003802	Pttg1	906.02616	827.634	191.742	179.55	526.2	-2.223688	4.6E-25	2.9E-23
ENSRNOG00000011631	Fst	9655.6797	9989.77	1901.36	2304.8	5963	-2.223398	2.8E-43	6.5E-41
ENSRNOG00000016208	Setbp1	1064.9419	1108.01	146.567	318.79	659.6	-2.220788	6.8E-15	1.4E-13
ENSRNOG00000005711	Ptprd	3131.8786	3261.73	566.19	807.04	1942	-2.218196	5.6E-30	5.6E-28
ENSRNOG00000003064	Bst1	201.22449	149.535	24.0932	51.299	106.5	-2.211928	2.6E-07	1.8E-06
ENSRNOG00000006916	Sardh	1321.8901	1225.36	231.897	318.79	774.5	-2.208095	4.9E-25	3E-23
ENSRNOG00000067723	Pclaf	1663.4558	1425.77	222.862	445.2	939.3	-2.207394	8.6E-17	2.3E-15
ENSRNOG00000029141	Trabd2b	281.71428	271.032	27.1049	92.521	168.1	-2.201926	2.4E-07	1.7E-06
ENSRNOG00000010947	Mmp14	13130.156	13153.9	2433.41	3281.3	8000	-2.201213	1.6E-36	2.4E-34

ENSRNOG00000029510	Plxnb1	1261.0068	1301.16	238.924	317.87	779.7	-2.200698	5E-26	3.4E-24
ENSRNOG00000067638	ENSRNOG0000006763	469.52381	551.41	69.268	152.98	310.8	-2.195639	1.2E-11	1.6E-10
ENSRNOG00000002751	Zdhhc15	317.8315	299.07	62.2408	72.368	187.9	-2.194857	4.7E-13	8E-12
ENSRNOG00000027936	Ccdc8	1715.0518	1646.96	223.866	511.16	1024	-2.191757	8.4E-15	1.8E-13
ENSRNOG00000018943	Tnnc1	1031.9205	1045.71	187.726	266.57	633	-2.191353	1.4E-22	6.9E-21
ENSRNOG00000016812	Adamts16	42.308739	71.6522	15.0583	10.077	34.77	-2.184893	0.0009	0.00302
ENSRNOG00000039471	Pcdhb16l	147.56463	128.766	23.0893	37.558	84.24	-2.183146	6.2E-07	4E-06
ENSRNOG00000019211	Olfml3	3998.6918	3573.26	723.8	943.53	2310	-2.182523	7.8E-33	9.1E-31
ENSRNOG00000015308	Pbk	898.80272	778.828	119.462	250.08	511.8	-2.179779	6.4E-14	1.2E-12
ENSRNOG00000053875	Nacad	346.72527	325.031	45.1748	102.6	204.9	-2.179494	7.8E-10	8.4E-09
ENSRNOG00000021261	Rassf2	45.4045	45.6912	10.0388	10.077	27.8	-2.179037	0.00188	0.00588
ENSRNOG00000026324	Stard6	58.819466	64.3831	5.01942	21.985	37.55	-2.17826	0.00153	0.00489
ENSRNOG00000063244	Mpeg1	39.212977	57.114	14.0544	7.3284	29.43	-2.178218	0.0021	0.00651
ENSRNOG00000039107	Mfrp	79.457875	67.4984	15.0583	17.405	44.85	-2.176937	0.00011	0.00046
ENSRNOG00000028636	ENSRNOG0000002863	357.04448	330.223	46.1786	105.35	209.7	-2.176318	7.3E-10	7.9E-09
ENSRNOG00000020013	Psrc1	263.13972	251.302	40.1553	73.284	157	-2.176249	8.8E-10	9.3E-09
ENSRNOG00000058329	Prrx2	901.89848	861.903	160.621	229.93	538.6	-2.173057	1.1E-20	4.2E-19
ENSRNOG00000001584	Map3k7cl	1129.9529	1155.78	275.064	232.68	698.4	-2.171478	7.8E-27	5.7E-25
ENSRNOG00000068084	ENSRNOG0000006808	326.08686	261.686	57.2214	73.284	179.6	-2.169006	1.1E-11	1.6E-10
ENSRNOG00000066110	ENSRNOG0000006611	43.340659	55.0372	9.03495	12.825	30.06	-2.166123	0.00154	0.00492
ENSRNOG00000007261	Gli2	815.21716	879.556	171.664	206.11	518.1	-2.16427	7.8E-23	3.8E-21
ENSRNOG00000048166	Arhgap19	874.03663	935.632	173.672	231.76	553.8	-2.156468	8.1E-22	3.6E-20

ENSRNOG00000020342	Samd11	197.09681	171.342	31.1204	51.299	112.7	-2.155856	3.6E-08	3E-07
ENSRNOG00000006787	Dhcr24	1613.9236	1718.61	345.336	402.15	1020	-2.155784	1.3E-30	1.3E-28
ENSRNOG00000002110	Fam114a1l1	21.67033	20.7687	4.01553	5.4963	12.99	-2.153962	0.03306	0.07145
ENSRNOG00000030266	Plekhg2	2195.9267	2249.25	379.468	619.25	1361	-2.152842	2.6E-22	1.2E-20
ENSRNOG00000060587	AABR07002627.1	98.032443	10.3844	8.03107	16.489	33.23	-2.140243	0.01376	0.03366
ENSRNOG00000005871	Il1rn	431.34275	426.798	73.2835	120.92	263.1	-2.140134	3.4E-13	5.9E-12
ENSRNOG00000012802	Tenm3	7779.6483	7908.74	1349.22	2209.5	4812	-2.139856	1.3E-24	7.7E-23
ENSRNOG00000064142	ENSRNOG0000006414	3014.2396	2793.4	439.701	878.49	1781	-2.138362	2.1E-17	6E-16
ENSRNOG00000014739	Wfdc2	225.99058	232.61	50.1942	54.047	140.7	-2.136577	2.3E-10	2.7E-09
ENSRNOG00000010802	Ube3d	253.85243	180.688	40.1553	58.627	133.3	-2.133982	9.5E-09	8.5E-08
ENSRNOG00000012110	Col17a1	38.181057	53.9987	10.0388	10.993	28.3	-2.130807	0.00239	0.00728
ENSRNOG00000046276	Myh3	77.394034	40.499	15.0583	11.909	36.21	-2.130752	0.00104	0.00347
ENSRNOG00000028619	Hoxc8	55.723705	64.3831	8.03107	19.237	36.84	-2.130641	0.00096	0.00323
ENSRNOG00000013017	Arnt2	919.44113	946.016	183.711	241.84	572.8	-2.130515	4.7E-22	2.2E-20
ENSRNOG00000026252	E2f7	1061.8461	1146.43	112.435	392.99	678.4	-2.126944	0.01032	0.02625
ENSRNOG00000015049	Scn5a	630.5034	532.718	109.423	156.64	357.3	-2.125917	4.6E-16	1.1E-14
ENSRNOG00000060703	Troap	2105.1177	2473.56	425.647	625.66	1407	-2.121692	6.3E-24	3.5E-22
ENSRNOG00000001407	Tfr2	73.266352	64.3831	17.066	14.657	42.34	-2.119109	0.00023	0.0009
ENSRNOG00000012051	Ncaph	2969.8671	3136.08	456.767	949.94	1878	-2.116916	2.2E-16	5.4E-15
ENSRNOG00000002579	Parm1	10588.536	10915	2173.41	2794	6618	-2.113792	3.6E-36	5E-34
ENSRNOG00000015991	Npr2	1416.8268	1465.23	242.94	423.21	887.1	-2.111405	2.3E-18	7.5E-17
ENSRNOG00000010743	Fdxacb1	80.489795	88.2672	17.066	21.985	51.95	-2.108781	5.9E-05	0.00026

ENSRNOG00000005574	Adamts8	72.234432	106.959	13.0505	28.398	55.16	-2.105186	0.00014	0.00056
ENSRNOG00000012835	Espl1	3786.1161	3823.53	606.346	1162.5	2345	-2.104253	2.7E-18	8.5E-17
ENSRNOG00000019184	Npr3	2309.438	2315.71	367.421	708.11	1425	-2.103225	2.4E-17	6.7E-16
ENSRNOG00000011777	Spag5	2995.6651	2859.86	630.439	731.92	1804	-2.103216	1.2E-34	1.6E-32
ENSRNOG00000003616	Grem2	16670.675	16919.3	4151.06	3669.7	10353	-2.10274	2.1E-44	5.5E-42
ENSRNOG00000028856	Pknox2	54.691784	70.6137	7.02718	21.985	38.58	-2.101018	0.00136	0.0044
ENSRNOG00000015529	Cdca3	2947.1648	2902.43	497.926	864.75	1803	-2.100908	2.5E-20	9.6E-19
ENSRNOG00000005608	Tead4	402.44898	450.682	63.2447	135.58	263	-2.097134	1.8E-10	2.1E-09
ENSRNOG00000010646	Tmem229b	21.67033	26.9994	4.01553	7.3284	15	-2.09452	0.02749	0.06127
ENSRNOG00000017932	St3gal2	4701.4296	4506.82	774.998	1380.5	2841	-2.094258	3E-20	1.1E-18
ENSRNOG00000049862	Rnf157	1039.1439	970.939	122.474	348.1	620.2	-2.094202	0.00238	0.00728
ENSRNOG00000021314	Fdft1	1624.2428	1676.04	364.41	409.47	1019	-2.091843	6.1E-30	6.1E-28
ENSRNOG00000007906	Bub1b	2925.4945	2698.9	556.151	763.07	1736	-2.091194	1.7E-27	1.4E-25
ENSRNOG00000063920	ENSRNOG0000006392	487.06645	462.105	54.2097	168.55	293	-2.087064	2.7E-08	2.3E-07
ENSRNOG00000065770	Rmi2	298.22501	272.071	29.1126	105.35	176.2	-2.078965	1.5E-06	9E-06
ENSRNOG00000008270	Cntrob	716.1528	799.597	133.517	225.35	468.7	-2.075933	2.4E-15	5.3E-14
ENSRNOG00000027606	Neurl1b	374.58713	394.606	33.1282	149.32	237.9	-2.074901	0.03122	0.06818
ENSRNOG00000019022	Fam89a	52.627943	12.4612	10.0388	5.4963	20.16	-2.072425	0.02426	0.05513
ENSRNOG00000067409	Cfap299	61.915227	70.6137	15.0583	16.489	41.02	-2.069709	0.00037	0.00138
ENSRNOG00000067849	ENSRNOG0000006784	291.00157	245.071	42.1631	85.193	165.9	-2.068739	1.2E-08	1.1E-07
ENSRNOG00000022391	ENSRNOG0000002239	44.37258	43.6144	9.03495	11.909	27.23	-2.06776	0.00342	0.01003
ENSRNOG00000019622	Ackr3	6927.282	6576.42	1457.64	1766.1	4182	-2.066245	2.6E-36	3.6E-34

ENSRNOG00000038035	Kif4a	3070.9953	3245.12	530.051	978.34	1956	-2.065117	3.1E-18	9.8E-17
ENSRNOG00000005655	Cpa6	292.03349	261.686	70.2718	62.291	171.6	-2.063596	4.2E-11	5.5E-10
ENSRNOG00000052173	Ranbp3l	66.042909	61.2678	12.0466	18.321	39.42	-2.063387	0.00058	0.00204
ENSRNOG00000014693	Btbd6	470.55573	495.335	74.2874	156.64	299.2	-2.060582	7.6E-11	9.5E-10
ENSRNOG00000016538	Itga8	4589.9822	4433.09	999.868	1168	2798	-2.057026	5E-36	6.9E-34
ENSRNOG00000015461	Serpine2	1642.8174	1736.27	366.418	445.2	1048	-2.056919	1.8E-27	1.4E-25
ENSRNOG00000048576	RGD1562844	24.766091	26.9994	6.0233	6.4123	16.05	-2.056776	0.02315	0.05298
ENSRNOG00000005185	ENSRNOG0000000518	34.053375	45.6912	10.0388	9.1605	24.74	-2.055256	0.00559	0.01548
ENSRNOG00000063732	H1f0	10970.346	10370.9	1757.8	3394.9	6623	-2.049959	4.6E-18	1.4E-16
ENSRNOG00000023296	Wtip	912.21768	884.748	179.695	253.75	557.6	-2.049803	3.6E-19	1.2E-17
ENSRNOG00000012952	Lrig1	3770.6373	3712.41	902.491	908.72	2324	-2.046652	5.5E-38	9.1E-36
ENSRNOG00000020505	Map4k1	27.861852	49.845	4.01553	14.657	24.09	-2.046381	0.01241	0.03089
ENSRNOG00000005659	Aurkb	2258.8739	2105.95	330.278	726.43	1355	-2.045113	5.5E-14	1.1E-12
ENSRNOG00000064037	Hpse2	56.755625	89.3056	17.066	18.321	45.36	-2.044454	0.00034	0.00126
ENSRNOG00000020140	Pigq	1807.9246	1679.15	333.289	512.99	1083	-2.041484	9E-21	3.6E-19
ENSRNOG00000026490	Dnm3	233.21402	234.687	53.2058	60.459	145.4	-2.040211	7.9E-10	8.4E-09
ENSRNOG00000015465	Cep72	586.13082	554.525	84.3262	192.37	354.3	-2.04002	1.5E-10	1.8E-09
ENSRNOG00000002139	Cracd	1237.2726	1288.7	269.041	346.27	785.3	-2.036241	2.4E-23	1.2E-21
ENSRNOG00000013547	Slc6a12	329.18263	316.723	59.2291	98.017	200.8	-2.034577	1.6E-10	1.9E-09
ENSRNOG00000033119	Plcb4	3047.2611	3264.85	557.155	982.92	1963	-2.034238	8.6E-19	2.9E-17
ENSRNOG00000003956	Arhgap6	12.383045	31.1531	7.02718	3.6642	13.56	-2.032061	0.04862	0.09867
ENSRNOG00000020716	Axl	15577.871	15319	2987.56	4572.9	9614	-2.030721	1.8E-25	1.2E-23

ENSRNOG00000027511	AC119015.1	158.91575	163.035	30.1165	48.551	100.2	-2.028697	5.1E-07	3.3E-06
ENSRNOG00000000889	AABR07011057.1	77.394034	66.46	15.0583	20.153	44.77	-2.027506	0.00031	0.00116
ENSRNOG00000000420	Nelfe	1007.1544	1067.51	197.765	310.54	645.7	-2.027157	1.1E-17	3.3E-16
ENSRNOG00000007437	Irf5	134.14966	180.688	34.132	43.054	98.01	-2.025973	5.6E-07	3.7E-06
ENSRNOG00000005573	Ntn4	1447.7844	1370.74	237.92	454.36	877.7	-2.023767	3.4E-15	7.5E-14
ENSRNOG00000012181	Lpl	1052.5589	1082.05	214.831	309.62	664.8	-2.023361	1.5E-19	5.5E-18
ENSRNOG00000062420	ENSRNOG0000006242	87.713239	105.921	21.0816	26.565	60.32	-2.020488	3.8E-05	0.00018
ENSRNOG00000045779	AABR07039210.2	749.17425	707.176	208.808	152.06	454.3	-2.014711	2.1E-17	6E-16
ENSRNOG00000020695	Tead2	572.71585	556.602	101.392	177.71	352.1	-2.013508	7.5E-13	1.2E-11
ENSRNOG00000000906	Medag	1015.4097	1012.48	207.804	294.05	632.4	-2.012925	2.1E-19	7.2E-18
ENSRNOG00000016724	Epb41l3	3987.3406	4047.83	791.06	1200	2507	-2.012113	1.5E-23	7.9E-22
ENSRNOG00000010308	Nr2f2	8276.002	8393.69	1522.89	2608.9	5200	-2.012029	1.4E-20	5.6E-19
ENSRNOG00000049537	ENSRNOG0000004953	6531.0246	6872.38	1367.29	1964	4184	-2.008047	1.6E-26	1.2E-24
ENSRNOG00000054603	Pacsin1	33.021455	13.4997	7.02718	4.5802	14.53	-2.007392	0.04251	0.08829
ENSRNOG00000020202	Asrgl1	510.80063	510.911	95.3689	158.48	318.9	-2.005911	8.2E-13	1.4E-11
ENSRNOG00000064807	ENSRNOG0000006480	181.618	174.457	38.1476	50.383	111.2	-2.005343	9.4E-08	7.1E-07
ENSRNOG00000057691	AC112557.1	37.149136	19.7303	4.01553	10.077	17.74	-2.004556	0.02717	0.06067
ENSRNOG00000028627	Hmcn1	7026.3464	6884.84	1300.03	2171	4346	-2.002378	5.3E-21	2.2E-19
ENSRNOG00000046231	Cacna1s	42.308739	44.6528	6.0233	15.573	27.14	-2.000804	0.00657	0.01777
ENSRNOG00000025551	Rgs22	20.638409	29.0762	6.0233	6.4123	15.54	-1.99841	0.03029	0.06641
ENSRNOG00000053456	ENSRNOG0000005345	782.1957	751.828	163.633	219.85	479.4	-1.998334	6.4E-18	1.9E-16
ENSRNOG00000012659	ENSRNOG0000001265	175.42648	102.805	19.0738	50.383	86.92	-1.995842	6E-05	0.00027

ENSRNOG00000060753	Esyt1	13326.221	12795.6	2967.48	3586.3	8169	-1.994702	5.3E-36	7.2E-34
ENSRNOG00000002487	ENSRNOG0000000248	714.08895	716.522	138.536	219.85	447.2	-1.994611	4.2E-15	9.1E-14
ENSRNOG00000008057	ENSRNOG0000000805	2505.5029	2509.9	584.26	677.88	1569	-1.990001	1.7E-30	1.7E-28
ENSRNOG00000068162	Npas3	60.883307	41.5375	12.0466	13.741	32.05	-1.988502	0.00257	0.00778
ENSRNOG00000016103	Nkd2	1437.4652	1558.69	223.866	530.39	937.6	-1.988347	9E-12	1.3E-10
ENSRNOG00000011927	Sdc3	50.564102	72.6906	8.03107	22.901	38.55	-1.985767	0.00232	0.00709
ENSRNOG00000004210	Osr1	3552.9021	3625.18	686.656	1125.8	2248	-1.984885	2.9E-20	1.1E-18
ENSRNOG00000051548	Lmod1	8794.0261	8955.48	1540.96	2942.4	5558	-1.984811	3E-17	8.4E-16
ENSRNOG00000014508	Mgll	536.59864	550.372	77.299	196.95	340.3	-1.983241	4.2E-09	4E-08
ENSRNOG00000005450	Lsm11	548.98168	629.293	108.419	189.62	369.1	-1.980199	1.7E-12	2.7E-11
ENSRNOG00000022957	Ctxn3	248.69283	236.764	32.1243	90.689	152.1	-1.977247	1.5E-06	9E-06
ENSRNOG00000016099	Id4	691.3867	727.944	126.489	233.59	444.9	-1.976096	7.8E-13	1.3E-11
ENSRNOG00000004155	Samd14	168.20303	154.727	39.1515	43.054	101.3	-1.973012	3E-07	2E-06
ENSRNOG00000053945	Daam2	1068.0377	1105.94	179.695	375.58	682.3	-1.96697	1.6E-12	2.6E-11
ENSRNOG00000007668	Mafa	74.298273	60.2294	16.0621	18.321	42.23	-1.966825	0.0006	0.00213
ENSRNOG00000012950	Efr3b	21.67033	22.8456	4.01553	7.3284	13.96	-1.966112	0.04351	0.09005
ENSRNOG00000001823	St6gal1	1717.1156	1598.15	359.39	489.17	1041	-1.964929	2.8E-22	1.3E-20
ENSRNOG00000060949	Anxa8	1254.8153	1124.63	274.06	335.27	747.2	-1.964344	6.8E-22	3.1E-20
ENSRNOG00000067771	ENSRNOG0000006777	38.181057	47.7681	11.0427	10.993	27	-1.963654	0.00539	0.015
ENSRNOG00000027770	Trpm3	83.585557	80.9981	22.0854	20.153	51.71	-1.96314	0.00015	0.00062
ENSRNOG00000012991	Adgra2	10596.791	10500.7	2253.72	3165	6629	-1.960813	3.6E-27	2.8E-25
ENSRNOG00000014973	Tekt1	30.957614	17.6534	7.02718	5.4963	15.28	-1.959375	0.037	0.07859

ENSRNOG00000013215	Dctd	544.854	504.68	83.3223	185.96	329.7	-1.95912	9.1E-10	9.5E-09
ENSRNOG00000004346	Notch3	316.79958	470.412	68.2641	133.74	247.3	-1.958856	8E-09	7.3E-08
ENSRNOG00000029993	Kynu	111.44741	109.036	7.02718	49.467	69.24	-1.955959	0.00154	0.00492
ENSRNOG00000014182	Tns1	16730.526	16689.8	3502.55	5127.1	10512	-1.953181	1.4E-25	9.3E-24
ENSRNOG00000042374	Tgif2	47.468341	64.3831	17.066	11.909	35.21	-1.952261	0.00201	0.00626
ENSRNOG00000021079	Fxyd1	12.383045	35.3069	5.01942	7.3284	15.01	-1.945525	0.04805	0.09766
ENSRNOG00000014786	Ccne1	1819.2758	1700.96	317.227	596.35	1108	-1.944699	6.5E-15	1.4E-13
ENSRNOG00000027722	H1f10	651.14181	673.946	126.489	217.1	417.2	-1.944663	4.4E-13	7.5E-12
ENSRNOG00000062746	ENSRNOG0000006274	38.181057	31.1531	6.0233	11.909	21.82	-1.944366	0.01458	0.03541
ENSRNOG00000000142	Plxdc2	2695.3762	2628.28	493.911	888.57	1677	-1.944191	1.1E-16	3E-15
ENSRNOG00000059961	Rapgef3	31.989534	45.6912	12.0466	8.2444	24.49	-1.940495	0.00952	0.02448
ENSRNOG00000003069	Cd38	45.4045	21.8072	13.0505	4.5802	21.21	-1.939722	0.02307	0.05283
ENSRNOG00000063755	Gas2l3	2009.1491	1962.65	477.849	558.79	1252	-1.937291	4.5E-27	3.4E-25
ENSRNOG00000017448	Exoc3l2	244.56515	284.532	58.2252	79.696	166.8	-1.937097	2.5E-09	2.5E-08
ENSRNOG00000017854	Ucp2	1521.0508	1604.39	410.588	405.81	985.5	-1.936761	1.7E-26	1.2E-24
ENSRNOG00000026143	Ckap2l	1319.8263	1259.62	226.878	447.95	813.6	-1.932705	2.7E-13	4.7E-12
ENSRNOG00000018111	Slc12a5	14.446886	36.3453	5.01942	8.2444	16.01	-1.932124	0.0418	0.08699
ENSRNOG00000019174	Chtf18	919.44113	871.249	154.598	314.2	564.9	-1.931128	5.9E-12	8.6E-11
ENSRNOG00000025994	Krt80	417.92778	424.721	107.416	113.59	265.9	-1.930399	1.5E-13	2.7E-12
ENSRNOG00000065786	ENSRNOG0000006578	232.1821	261.686	42.1631	87.025	155.8	-1.929871	1.8E-07	1.3E-06
ENSRNOG00000028384	Npepl1	1734.6583	1653.19	327.266	562.45	1069	-1.927575	2.6E-16	6.4E-15
ENSRNOG00000066437	Ntm	492.22606	546.218	114.443	158.48	327.8	-1.925732	1.4E-13	2.6E-12

ENSRNOG00000025384	Slc2a10	500.48142	489.104	115.447	144.74	312.4	-1.925681	3.7E-14	7.2E-13
ENSRNOG00000018992	Dpysl3	13153.89	13522.5	2965.47	4061.8	8426	-1.924349	1.4E-27	1.1E-25
ENSRNOG00000008919	Arpp21	36.117216	30.1147	12.0466	5.4963	20.94	-1.924265	0.01822	0.04307
ENSRNOG00000014029	Klhl13	4372.247	4460.09	1076.16	1252.2	2790	-1.92314	5.5E-32	6.2E-30
ENSRNOG00000013328	Rbpms	993.7394	1035.32	236.917	297.72	640.9	-1.922995	6.4E-20	2.3E-18
ENSRNOG00000025100	Ikbke	613.99267	752.867	106.412	253.75	431.8	-1.921358	2.1E-09	2.1E-08
ENSRNOG00000001588	Hoxd13	733.69544	697.83	135.524	242.75	452.5	-1.917481	1.4E-12	2.2E-11
ENSRNOG00000024557	Cep112	58.819466	64.3831	17.066	15.573	38.96	-1.917288	0.00116	0.00382
ENSRNOG00000014816	Slc1a1	37.149136	34.2684	6.0233	12.825	22.57	-1.914634	0.01469	0.03563
ENSRNOG00000025408	Dux4	39.212977	68.5369	12.0466	16.489	34.07	-1.913727	0.00347	0.01015
ENSRNOG00000000479	Kifc1	2477.641	2546.25	452.751	881.24	1589	-1.912046	1.5E-14	3E-13
ENSRNOG00000006726	Zfp9	295.12925	310.493	76.2952	85.193	191.8	-1.90605	1.2E-10	1.5E-09
ENSRNOG00000029912	Septin5	9418.338	9515.2	2534.81	2522.8	5998	-1.904423	3.1E-38	5.2E-36
ENSRNOG00000019662	Tm6sf1	637.72684	623.062	138.536	197.87	399.3	-1.903973	1.7E-14	3.5E-13
ENSRNOG00000013437	ENSRNOG0000001343	59.851386	71.6522	14.0544	21.069	41.66	-1.900606	0.00109	0.00361
ENSRNOG00000065861	Samd1	1241.4003	1145.4	214.831	424.13	756.4	-1.899443	1.1E-12	1.8E-11
ENSRNOG00000052129	Nwd1	361.17216	507.796	60.233	172.22	275.4	-1.898706	5.3E-07	3.4E-06
ENSRNOG00000002449	Maged2	1636.6258	1515.08	360.394	484.59	999.2	-1.898065	6.1E-21	2.5E-19
ENSRNOG00000009872	Kcnh2	104.22397	113.19	29.1126	29.314	68.96	-1.895666	2.9E-05	0.00014
ENSRNOG00000000632	Cdk1	5006.878	4679.2	910.522	1698.4	3074	-1.891937	8.3E-16	1.9E-14
ENSRNOG00000025948	Inava	34.053375	29.0762	5.01942	11.909	20.01	-1.890871	0.02344	0.05357
ENSRNOG00000008053	Atp8a2	487.06645	478.72	108.419	152.06	306.6	-1.888267	9.3E-13	1.5E-11

ENSRNOG00000013791	Enpp3	75.330193	88.2672	24.0932	20.153	51.96	-1.888244	0.00027	0.00105
ENSRNOG00000007164	Cln6	1943.1062	1878.53	353.367	679.71	1214	-1.885997	6E-14	1.1E-12
ENSRNOG00000047124	AABR07005775.1	219.79906	165.112	51.1981	53.131	122.3	-1.883123	2.2E-07	1.6E-06
ENSRNOG00000066004	ENSRNOG0000006600	57.787545	46.7297	8.03107	20.153	33.18	-1.882923	0.00524	0.01462
ENSRNOG00000011453	Kif6	46.43642	44.6528	9.03495	15.573	28.92	-1.882863	0.00635	0.01728
ENSRNOG00000065997	ENSRNOG0000006599	170.26688	185.88	23.0893	73.284	113.1	-1.879474	4.3E-05	0.00019
ENSRNOG00000021579	Mpp6	1387.933	1520.27	274.06	515.74	924.5	-1.879038	1.5E-13	2.7E-12
ENSRNOG00000058039	Acta2	229687.95	227344	50516.4	73851	1E+05	-1.877675	2.1E-24	1.2E-22
ENSRNOG00000068431	Eppk1	112.47933	75.8059	19.0738	32.062	59.86	-1.876129	0.00026	0.001
ENSRNOG00000059997	Brip1	660.42909	645.908	125.485	229.93	415.4	-1.875278	1.5E-11	2E-10
ENSRNOG00000024077	Fbxo5	1477.7101	1380.08	201.781	578.94	909.6	-1.871679	0.00718	0.01922
ENSRNOG00000062673	Fendrr	576.84353	668.754	137.532	202.45	396.4	-1.871177	2.6E-13	4.6E-12
ENSRNOG00000018254	Sncaip	47.468341	40.499	15.0583	9.1605	28.05	-1.86576	0.00758	0.02014
ENSRNOG00000070404	ENSRNOG0000007040	158.91575	125.651	32.1243	45.802	90.62	-1.865538	7.6E-06	4E-05
ENSRNOG00000011367	Cyp2r1	205.35217	220.149	57.2214	59.543	135.6	-1.865188	3.5E-08	2.9E-07
ENSRNOG00000045997	Chst14	704.80167	806.866	173.672	241.84	481.8	-1.861442	5.3E-15	1.1E-13
ENSRNOG00000026504	Fam114a1	5712.7116	5739.44	1451.62	1701.1	3651	-1.860698	5.4E-31	5.8E-29
ENSRNOG00000015036	Ccn2	63798.482	62168.1	14540.2	20207	40178	-1.858025	5.8E-26	3.9E-24
ENSRNOG00000015275	Ska1	799.73835	778.828	137.532	297.72	503.5	-1.856239	3.1E-10	3.5E-09
ENSRNOG00000069555	ENSRNOG0000006955	3629.2642	3477.73	657.544	1306.3	2268	-1.854857	7.7E-14	1.4E-12
ENSRNOG00000048431	Nynrin	560.33281	580.486	111.431	203.36	363.9	-1.854729	7.1E-11	8.9E-10
ENSRNOG00000008336	Tnfrsf11b	4481.6305	4472.55	1093.23	1382.3	2857	-1.854403	5.6E-27	4.1E-25

ENSRNOG00000023453	Lrba	1411.6672	1605.42	272.052	561.54	962.7	-1.854267	6.2E-12	9E-11
ENSRNOG00000071108	Hoxd8	1070.1015	1168.24	233.905	384.74	714.2	-1.853515	1.3E-14	2.8E-13
ENSRNOG00000042980	Adam19	7651.6902	7396.79	1681.5	2482.5	4803	-1.853248	4.2E-22	1.9E-20
ENSRNOG00000015329	Kpna2	5775.6588	5630.41	1281.96	1877.9	3641	-1.851448	5.8E-22	2.6E-20
ENSRNOG00000017414	Irf7	289.96965	210.803	69.268	69.62	159.9	-1.850257	2.2E-08	1.9E-07
ENSRNOG00000060356	Kif15	1310.539	1345.81	213.827	522.15	848.1	-1.850069	5.7E-10	6.2E-09
ENSRNOG00000017259	Tacc3	3825.3291	3694.76	852.297	1233	2401	-1.849896	1.7E-21	7.5E-20
ENSRNOG00000037886	Hs6st3	170.26688	145.381	36.1398	51.299	100.8	-1.848985	2.9E-06	1.7E-05
ENSRNOG00000019615	Colq	54.691784	59.1909	16.0621	15.573	36.38	-1.848246	0.00232	0.00711
ENSRNOG00000000700	Tmem119	2223.7886	2056.11	530.051	658.64	1367	-1.847529	1.5E-23	8.1E-22
ENSRNOG00000003722	Dusp27	2702.5997	2582.59	696.695	773.15	1689	-1.845982	8E-28	6.7E-26
ENSRNOG00000004554	Dcn	184.71376	193.149	49.1903	55.879	120.7	-1.845369	2.1E-07	1.5E-06
ENSRNOG00000055994	AABR07029741.3	249.72475	261.686	66.2563	76.032	163.4	-1.844487	4.7E-09	4.4E-08
ENSRNOG00000063089	Tmem139	27.861852	23.8841	8.03107	6.4123	16.55	-1.843404	0.03722	0.07902
ENSRNOG00000033772	Serpinb9	16212.502	15621.2	3521.62	5352.5	10177	-1.84272	1.8E-21	7.7E-20
ENSRNOG00000003875	Ocrl	1064.9419	1208.74	214.831	419.55	727	-1.83979	6.6E-12	9.6E-11
ENSRNOG00000011714	Sat2	196.06489	185.88	33.1282	73.284	122.1	-1.838455	5.6E-06	3E-05
ENSRNOG00000020638	Hmx2	220.83098	190.034	49.1903	65.956	131.5	-1.832795	2E-07	1.4E-06
ENSRNOG00000007159	Ccl2	134.14966	146.42	56.2175	22.901	89.92	-1.832417	6.7E-05	0.0003
ENSRNOG00000001516	Rapgef4	1671.7111	1869.19	455.763	538.64	1134	-1.831598	1E-22	5E-21
ENSRNOG00000013884	Psd3	497.38566	577.371	135.524	166.72	344.3	-1.828849	2.3E-13	4.1E-12
ENSRNOG00000070963	ENSRNOG0000007096	243.53323	263.763	35.1359	107.18	162.4	-1.828656	1.1E-05	5.8E-05

ENSRNOG00000011136	Osr2	379.74673	290.762	87.3379	101.68	214.9	-1.825657	7E-10	7.5E-09
ENSRNOG00000016163	Slc1a3	587.16274	641.754	114.443	231.76	393.8	-1.824887	7.5E-10	8.1E-09
ENSRNOG00000014079	Stat4	18021.459	17706.4	3579.85	6518.6	11457	-1.822764	4.8E-16	1.1E-14
ENSRNOG00000042460	Hmga2	319.89534	325.031	82.3185	99.849	206.8	-1.822342	3.1E-10	3.5E-09
ENSRNOG00000064049	ENSRNOG0000006404	55.723705	68.5369	13.0505	21.985	39.82	-1.821611	0.00232	0.00711
ENSRNOG00000032759	Cdx2	25.798011	24.9225	7.02718	7.3284	16.27	-1.820529	0.04042	0.08457
ENSRNOG00000008243	Slc43a1	209.47985	214.956	52.2019	67.788	136.1	-1.820429	1.1E-07	8.4E-07
ENSRNOG00000008775	Lrguk	51.596023	50.8834	19.0738	10.077	32.91	-1.81966	0.0054	0.01501
ENSRNOG00000010721	Dlgap5	3245.3898	3139.2	832.219	980.17	2049	-1.816296	8.1E-27	5.9E-25
ENSRNOG00000020991	Ms4a6a	36.117216	38.4222	12.0466	9.1605	23.94	-1.816195	0.01432	0.03483
ENSRNOG00000059326	Abca9	2598.3757	2688.51	489.895	1012.2	1697	-1.814505	2.1E-12	3.2E-11
ENSRNOG00000042522	Pheta2	79.457875	66.46	11.0427	30.23	46.8	-1.814288	0.00221	0.00681
ENSRNOG00000047931	ENSRNOG0000004793	84612.318	78181.9	21520.3	24809	52281	-1.813025	5E-33	5.9E-31
ENSRNOG00000028677	ENSRNOG0000002867	1375.55	1325.05	335.297	433.29	867.3	-1.81196	1.8E-19	6.3E-18
ENSRNOG00000017369	Mustn1	107.31973	106.959	27.1049	33.894	68.82	-1.810547	6.9E-05	0.0003
ENSRNOG00000060775	Lmo7	3513.6891	3551.45	839.247	1177.1	2270	-1.808363	1.8E-21	7.7E-20
ENSRNOG00000016207	Galnt1	5888.1381	5699.98	1423.51	1884.3	3724	-1.808342	1.2E-24	6.9E-23
ENSRNOG00000018931	Dis3l2	470.55573	464.181	91.3534	174.97	300.3	-1.808211	2.4E-09	2.4E-08
ENSRNOG00000011781	Oplah	1292.9963	1327.12	340.317	407.64	842	-1.807799	1.4E-20	5.4E-19
ENSRNOG00000004110	Trib2	3049.3249	3009.39	780.018	950.86	1947	-1.807023	2.2E-25	1.4E-23
ENSRNOG00000015155	Tnnc2	346.72527	278.301	86.334	92.521	201	-1.804637	1.2E-09	1.2E-08
ENSRNOG00000018500	Frmd4a	1972	2065.45	539.085	618.33	1299	-1.802053	3.1E-24	1.8E-22

ENSRNOG00000007286	Mdm1	1476.6782	1402.93	278.076	548.71	926.6	-1.798793	5.9E-12	8.7E-11
ENSRNOG00000015520	ENSRNOG0000001552	1302.2836	1345.81	250.971	510.24	852.3	-1.796989	2.1E-11	2.8E-10
ENSRNOG00000015541	Gnb3	95.968602	106.959	16.0621	42.138	65.28	-1.794969	0.00055	0.00196
ENSRNOG00000002052	Ccdc80	38558.74	38815.7	9066.07	13233	24918	-1.794793	2.7E-22	1.3E-20
ENSRNOG00000042944	Cenpw	929.76033	957.439	222.862	320.62	607.7	-1.794334	2.7E-15	5.9E-14
ENSRNOG00000010701	Depdc1b	459.2046	490.142	113.439	160.31	305.8	-1.791902	1.5E-11	2.1E-10
ENSRNOG00000009192	Rnaseh2b	561.36473	537.91	141.548	175.88	354.2	-1.790644	2E-13	3.5E-12
ENSRNOG00000013257	Hecw2	597.48194	628.254	132.513	221.68	395	-1.788553	2.6E-11	3.4E-10
ENSRNOG00000012730	Lrrk1	2607.663	2618.94	673.606	842.77	1686	-1.784662	2.2E-23	1.2E-21
ENSRNOG00000025074	Fgg	102.16013	120.459	39.1515	25.649	71.85	-1.78414	9.7E-05	0.00041
ENSRNOG00000039668	Col8a1	9845.5531	9889.04	2288.85	3440.7	6366	-1.783989	2.5E-20	9.5E-19
ENSRNOG00000020700	ENSRNOG0000002070	386.97017	370.722	82.3185	137.41	244.4	-1.78281	3E-09	3E-08
ENSRNOG00000038970	Fgd1	1197.0277	1169.28	295.142	392.07	763.4	-1.782623	1.1E-17	3.3E-16
ENSRNOG00000023628	Tmem106a	272.427	250.263	59.2291	92.521	168.6	-1.781059	7E-08	5.4E-07
ENSRNOG00000016316	Mcm2	5640.4772	5828.75	1428.53	1910.9	3702	-1.779748	1.5E-23	8.1E-22
ENSRNOG00000051487	Kremen1	3696.3391	3630.38	1028.98	1105.7	2365	-1.778997	8.8E-29	8.1E-27
ENSRNOG00000007839	Slc16a7	816.24908	789.212	216.839	251	518.3	-1.778077	1.2E-16	3E-15
ENSRNOG00000066557	RGD1565779	182.64992	204.572	57.2214	55.879	125.1	-1.775737	3.7E-07	2.5E-06
ENSRNOG00000056651	ENSRNOG0000005665	1278.5494	1112.17	323.251	374.66	772.2	-1.775673	7.6E-19	2.5E-17
ENSRNOG00000015812	Tm4sf1	337.43799	374.876	82.3185	125.5	230	-1.774417	2.5E-09	2.5E-08
ENSRNOG00000008843	Eci1	293.06541	303.224	73.2835	100.77	192.6	-1.774138	5.6E-09	5.2E-08
ENSRNOG00000020525	Col5a3	8142.8843	8279.46	1988.69	2813.2	5306	-1.773712	5.4E-22	2.5E-20

ENSRNOG00000004158	Hdac9	356.01256	399.798	60.233	160.31	244.1	-1.77311	1.3E-06	7.9E-06
ENSRNOG00000007206	Cfap20dc	230.11826	229.495	46.1786	87.941	148.4	-1.772628	1.1E-06	6.9E-06
ENSRNOG00000021206	Plaat3	212.57561	217.033	56.2175	69.62	138.9	-1.769674	1.6E-07	1.1E-06
ENSRNOG00000008245	AABR07054614.1	1193.932	1249.24	362.402	354.51	790	-1.768987	1E-20	4.2E-19
ENSRNOG00000027410	Ccdc125	44.37258	34.2684	13.0505	10.077	25.44	-1.768296	0.01443	0.03507
ENSRNOG00000005700	Nsg1	14617.153	13748.9	4073.76	4254.1	9173	-1.768112	2.6E-33	3.1E-31
ENSRNOG00000006776	Smyd1	69.13867	55.0372	6.0233	30.23	40.11	-1.766812	0.01012	0.02582
ENSRNOG00000011789	Cdon	1418.8906	1467.31	360.394	487.34	933.5	-1.766407	4.1E-18	1.3E-16
ENSRNOG00000020129	Cdh3	1008.1863	881.633	252.979	302.3	611.3	-1.766102	8.4E-17	2.2E-15
ENSRNOG00000013223	Fah	107.31973	160.958	44.1709	34.81	86.81	-1.766031	3.9E-05	0.00018
ENSRNOG00000005115	Asf1b	1518.9869	1393.58	324.254	531.31	942	-1.766006	2.6E-14	5.2E-13
ENSRNOG00000017118	P4ha3	4532.1946	4581.58	1055.08	1626	2949	-1.764735	2.6E-18	8.4E-17
ENSRNOG00000008678	Antxr1	5550.7001	5628.33	1399.41	1895.3	3618	-1.762199	1.1E-22	5.4E-21
ENSRNOG00000013148	Rad54l	477.77917	509.873	105.408	185.96	319.8	-1.75832	1.1E-09	1.1E-08
ENSRNOG00000018394	Chrdl2	79.457875	96.5747	31.1204	21.069	57.06	-1.757452	0.00048	0.00175
ENSRNOG00000005691	Rnf32	43.340659	45.6912	6.0233	20.153	28.8	-1.756885	0.01647	0.03935
ENSRNOG00000015794	Fam83d	1997.798	1837	587.272	547.8	1242	-1.756626	2.2E-23	1.1E-21
ENSRNOG00000018110	Svil	4611.6525	4733.2	965.736	1799.1	3027	-1.756447	6.8E-14	1.3E-12
ENSRNOG00000047314	Tk1	4313.4275	3914.91	929.596	1506.9	2666	-1.755238	1.9E-16	4.9E-15
ENSRNOG00000008834	Dach1	854.43014	874.364	210.816	301.38	560.2	-1.753356	2.3E-14	4.6E-13
ENSRNOG00000010081	Tmem144	108.35165	120.459	32.1243	35.726	74.17	-1.752725	6.2E-05	0.00027
ENSRNOG00000013167	Hmgb2l1	2406.4385	2374.91	528.043	890.4	1550	-1.752189	6.9E-15	1.4E-13

ENSRNOG00000024798	Bora	723.37624	711.329	182.707	242.75	465	-1.752119	3.5E-14	6.8E-13
ENSRNOG00000056069	Kif11	5006.878	5140.26	1160.49	1854.1	3290	-1.750584	3.2E-17	9E-16
ENSRNOG00000010635	Igfbp4	124.86238	139.151	38.1476	40.306	85.62	-1.750169	2E-05	9.7E-05
ENSRNOG00000028036	Adamts7	1746.0094	1564.92	410.588	573.45	1074	-1.749455	2.2E-17	6.3E-16
ENSRNOG00000051792	Hspb2	643.91836	669.792	161.625	229.01	426.1	-1.747882	6.6E-13	1.1E-11
ENSRNOG00000028335	Fat4	1245.528	1327.12	279.08	486.42	834.5	-1.747256	8.5E-13	1.4E-11
ENSRNOG00000009339	Cenpe	3744.8393	4074.83	1003.88	1325.5	2537	-1.746675	6.8E-22	3.1E-20
ENSRNOG00000000165	ENSRNOG0000000016	230.11826	213.918	47.1825	85.193	144.1	-1.742029	1.5E-06	9.1E-06
ENSRNOG00000008115	Arhgap11a	5364.9544	5640.79	1246.82	2044.6	3574	-1.741033	1.9E-16	4.8E-15
ENSRNOG00000013541	Sh2d4a	62.947148	70.6137	16.0621	23.817	43.36	-1.740131	0.0021	0.00651
ENSRNOG00000002436	Mmd	4855.1857	4775.77	1415.48	1467.5	3128	-1.740048	1.6E-29	1.5E-27
ENSRNOG00000060100	Knl1	4411.4599	4576.39	901.487	1793.6	2921	-1.7371	1.7E-12	2.7E-11
ENSRNOG00000006998	Fhl4	105.25589	106.959	24.0932	39.39	68.92	-1.7369	0.0002	0.00078
ENSRNOG00000007302	Fbn1	17053.517	18146.7	4604.81	5972.6	11444	-1.734477	6.8E-25	4.2E-23
ENSRNOG00000014320	Inhba	1880.1591	1789.23	566.19	536.8	1193	-1.734316	4.6E-23	2.4E-21
ENSRNOG00000025710	Ces5a	248.69283	264.801	79.3068	75.116	167	-1.733892	2.1E-08	1.8E-07
ENSRNOG00000006472	Hspa2	127.95814	133.958	32.1243	46.719	85.19	-1.728861	3.9E-05	0.00018
ENSRNOG00000003606	AABR07014550.1	56.755625	56.0756	11.0427	22.901	36.69	-1.726711	0.0059	0.01623
ENSRNOG00000014549	Arhgef26	163.04343	175.496	36.1398	65.956	110.2	-1.725008	1.4E-05	7.2E-05
ENSRNOG00000033658	Kntc1	3016.3035	3084.16	653.528	1192.7	1987	-1.723595	2.2E-13	4E-12
ENSRNOG00000067072	ENSRNOG0000006707	126.92622	106.959	36.1398	34.81	76.21	-1.721312	7E-05	0.00031
ENSRNOG00000053969	AABR07008681.1	44.37258	28.0378	10.0388	11.909	23.59	-1.720638	0.02259	0.05194

ENSRNOG00000039924	Triqk	300.28885	292.839	75.2913	104.43	193.2	-1.720218	1.6E-08	1.4E-07
ENSRNOG00000030633	Lmf2	5336.0607	5414.41	1390.38	1872.4	3503	-1.719857	8.4E-22	3.7E-20
ENSRNOG00000007491	Hoxb13	38.181057	23.8841	5.01942	13.741	20.21	-1.717979	0.04218	0.0877
ENSRNOG00000007462	Septin8	6969.5908	7125.76	1699.57	2590.6	4596	-1.715788	3.3E-18	1E-16
ENSRNOG00000015810	Trip13	918.4092	797.52	186.722	335.27	559.5	-1.714914	9.3E-11	1.1E-09
ENSRNOG00000007431	Shroom1	1861.5845	1991.72	499.934	674.21	1257	-1.713638	2.3E-18	7.3E-17
ENSRNOG00000054286	Rrm2	15543.818	15620.2	3225.48	6276.8	10167	-1.71338	4.3E-13	7.3E-12
ENSRNOG00000012422	Tnik	66.042909	57.114	22.0854	15.573	40.2	-1.712725	0.00334	0.00982
ENSRNOG00000053269	Tmem106c	1156.7828	1034.28	222.862	445.2	714.8	-1.711858	3E-10	3.5E-09
ENSRNOG00000029465	Slc26a10	338.46991	367.607	70.2718	144.74	230.3	-1.711811	2.7E-07	1.9E-06
ENSRNOG00000032778	Bub1	3917.17	4030.17	1037.01	1388.7	2593	-1.711576	4.4E-21	1.9E-19
ENSRNOG00000005174	Tmem121	181.618	150.573	36.1398	65.04	108.3	-1.7109	2.1E-05	0.0001
ENSRNOG00000031420	Hyal2	1051.5269	1067.51	278.076	369.17	691.6	-1.709808	6.9E-16	1.6E-14
ENSRNOG00000008445	Dact1	2227.9163	2149.56	555.148	783.22	1429	-1.708797	7.9E-18	2.3E-16
ENSRNOG00000018382	Inpp4b	115.57509	136.035	32.1243	44.886	82.16	-1.705189	6.5E-05	0.00029
ENSRNOG00000019178	Taf10	772.90842	662.523	201.781	238.17	468.8	-1.705171	5.1E-14	9.8E-13
ENSRNOG00000013647	Polm	1753.2329	1757.04	309.196	767.65	1147	-1.70449	0.00536	0.01491
ENSRNOG00000005874	Tle2	400.38514	485.989	86.334	185.04	289.4	-1.704439	1.8E-07	1.3E-06
ENSRNOG00000020039	Slc5a2	59.851386	42.5759	13.0505	18.321	33.45	-1.704058	0.00787	0.02076
ENSRNOG00000005628	Hoxa1	141.3731	121.497	30.1165	50.383	85.84	-1.70327	7.6E-05	0.00033
ENSRNOG00000062503	ENSRNOG0000006250	5753.9885	6273.2	1548.99	2144.5	3930	-1.702915	2.5E-20	9.6E-19
ENSRNOG00000012980	Hyls1	462.30036	489.104	135.524	156.64	310.9	-1.702269	8.5E-12	1.2E-10

ENSRNOG00000049385	Adamtsl4	1575.7425	1726.92	431.67	584.44	1080	-1.699635	3E-17	8.4E-16
ENSRNOG00000001072	Cers4	701.70591	781.943	193.75	263.82	485.3	-1.695533	3.9E-13	6.8E-12
ENSRNOG00000010204	Tmem9	2125.7561	1897.22	532.058	710.85	1316	-1.693773	5.1E-18	1.6E-16
ENSRNOG00000000572	Chst3	1015.4097	1019.75	251.975	377.41	666.1	-1.691581	1.3E-13	2.3E-12
ENSRNOG00000008300	Zbtb8a	413.8001	370.722	60.233	182.29	256.8	-1.690087	1E-05	5.2E-05
ENSRNOG00000000275	Fam13c	406.57666	415.375	83.3223	171.3	269.1	-1.68738	1.4E-07	1E-06
ENSRNOG00000009225	Copz2	1372.4542	1371.78	333.289	518.48	899	-1.686559	5E-14	9.6E-13
ENSRNOG00000015566	Atp6v1e2	35.085295	38.4222	9.03495	13.741	24.07	-1.686438	0.0227	0.05215
ENSRNOG00000065345	Lrrc75a	305.44845	327.108	55.2136	141.07	207.2	-1.684193	5.8E-06	3.1E-05
ENSRNOG00000020704	Tkfc	698.61015	611.639	175.68	231.76	429.4	-1.683722	1.7E-12	2.7E-11
ENSRNOG00000016075	Dmrt1	339.50183	279.34	74.2874	118.17	202.8	-1.682162	1.1E-07	8.5E-07
ENSRNOG00000009694	Bmp4	1809.9885	1800.65	457.771	666.88	1184	-1.681783	4.6E-16	1.1E-14
ENSRNOG00000016706	Fanca	1127.8891	1108.01	222.862	473.6	733.1	-1.681047	1.8E-09	1.8E-08
ENSRNOG00000004682	Parpbp	986.51595	1028.05	222.862	404.89	660.6	-1.680417	7E-11	8.8E-10
ENSRNOG00000062645	ENSRNOG0000006264	3029.7185	3256.54	649.513	1310.9	2062	-1.680362	2.8E-11	3.8E-10
ENSRNOG00000010911	Tmem108	1991.6065	1961.61	481.864	751.16	1297	-1.679843	6.5E-15	1.4E-13
ENSRNOG00000004752	Nalcn	793.54683	771.559	174.676	313.29	513.3	-1.679315	2.4E-10	2.8E-09
ENSRNOG00000020300	Lsp1	2901.7603	2867.12	727.816	1072.7	1892	-1.679201	5E-17	1.3E-15
ENSRNOG00000070046	Cdc45	1068.0377	1058.17	243.944	419.55	697.4	-1.678459	1.1E-11	1.5E-10
ENSRNOG00000042458	AABR07046707.1	766.7169	796.481	160.621	327.03	512.7	-1.678382	3.9E-09	3.7E-08
ENSRNOG00000033010	Akr1c12	423.08739	382.145	91.3534	160.31	264.2	-1.674959	2.9E-08	2.4E-07
ENSRNOG00000067311	ENSRNOG0000006731	34.053375	51.9219	15.0583	11.909	28.24	-1.674783	0.01564	0.0376

ENSRNOG00000025338	Msi2	1226.9534	1193.16	269.041	488.25	794.4	-1.674605	2.9E-11	3.8E-10
ENSRNOG00000024494	AABR07056633.1	34.053375	45.6912	14.0544	10.993	26.2	-1.67298	0.01864	0.04391
ENSRNOG00000059538	Clec2g	1506.6039	1511.96	355.375	592.68	991.7	-1.669558	6.4E-13	1.1E-11
ENSRNOG00000007461	Klhl41	3713.8817	3337.54	902.491	1314.5	2317	-1.66874	3.7E-17	1E-15
ENSRNOG00000017645	Mylpf	41.276818	38.4222	15.0583	10.077	26.21	-1.668642	0.01877	0.04417
ENSRNOG00000020138	Slc4a3	811.08948	919.017	179.695	364.59	568.6	-1.666424	3.6E-09	3.5E-08
ENSRNOG00000005464	Lgalsl	2836.7493	2854.66	706.734	1086.4	1871	-1.665562	8.7E-16	2E-14
ENSRNOG00000003013	Rpgr	6959.2715	6576.42	1863.21	2403.7	4451	-1.66524	3.4E-22	1.6E-20
ENSRNOG00000039470	AC131360.1	280.68236	265.84	73.2835	98.933	179.7	-1.663873	8.1E-08	6.2E-07
ENSRNOG00000045949	Faah	28.893773	37.3837	8.03107	12.825	21.78	-1.663692	0.03292	0.07119
ENSRNOG00000018865	Adamts12	10370.801	10238	2445.46	4059.9	6779	-1.663322	2.3E-15	5.2E-14
ENSRNOG00000029038	Gemin8	817.281	817.25	205.796	309.62	537.5	-1.663314	2.7E-12	4.2E-11
ENSRNOG00000013867	Fgf1	2527.1732	2769.51	659.552	1013.2	1742	-1.662161	2.3E-15	5.1E-14
ENSRNOG00000007433	Cyb561	42.308739	40.499	4.01553	21.985	27.2	-1.660951	0.03565	0.07615
ENSRNOG00000065098	ENSRNOG0000006509	508.73678	586.717	123.478	222.6	360.4	-1.659808	6.9E-09	6.3E-08
ENSRNOG00000066894	ENSRNOG0000006689	326.08686	481.835	93.3612	162.14	265.9	-1.658249	2.1E-07	1.5E-06
ENSRNOG00000009681	Flot2	1510.7315	1438.24	339.313	594.52	970.7	-1.657687	6.1E-12	8.9E-11
ENSRNOG00000011841	Map2	443.7258	502.604	93.3612	207.03	311.7	-1.652476	3.5E-07	2.3E-06
ENSRNOG00000048949	Elovl6	521.11983	564.91	162.629	183.21	358	-1.650125	3.8E-12	5.8E-11
ENSRNOG00000008309	Rpa3	695.51439	656.292	225.874	205.2	445.7	-1.649473	8.6E-14	1.6E-12
ENSRNOG00000009514	Mme	11626.648	11304.4	3091.96	4218.4	7560	-1.649111	1E-20	4E-19
ENSRNOG00000056174	ENSRNOG0000005617	470.55573	416.413	118.458	163.97	292.4	-1.649047	8.2E-10	8.7E-09

ENSRNOG00000020339	Neurl1	192.96913	199.38	57.2214	67.788	129.3	-1.648716	1.7E-06	9.9E-06
ENSRNOG00000022356	Zc2hc1a	1954.4573	1866.07	433.678	784.14	1260	-1.648422	7.4E-12	1.1E-10
ENSRNOG00000062314	Foxq1	209.47985	223.264	34.132	103.51	142.6	-1.647582	0.00012	0.00051
ENSRNOG00000028137	Mki67	16339.428	17430.2	3623.02	7174.5	11142	-1.644886	7E-12	1E-10
ENSRNOG00000061100	Tmem150a	340.53375	357.222	85.3301	137.41	230.1	-1.644517	4.9E-08	3.9E-07
ENSRNOG00000002660	Magee1	254.88435	186.919	64.2485	76.948	145.7	-1.644432	1.5E-06	9.3E-06
ENSRNOG00000006569	Itgb8	191.9372	198.341	67.2602	57.711	128.8	-1.644152	1.8E-06	1E-05
ENSRNOG00000006281	Hoxa3	104.22397	107.997	33.1282	34.81	70.04	-1.642827	0.00022	0.00087
ENSRNOG00000060448	Hand2	1216.6342	1203.55	300.161	474.51	798.7	-1.642051	9.1E-13	1.5E-11
ENSRNOG00000008055	Ccne2	918.4092	976.131	182.707	424.13	625.3	-1.640582	4.3E-08	3.4E-07
ENSRNOG00000011263	Plac9	71.202511	50.8834	18.0699	21.069	40.31	-1.639922	0.00493	0.01388
ENSRNOG00000039284	Haus4	2141.2349	2048.84	481.864	862	1383	-1.639617	4.7E-12	7E-11
ENSRNOG00000014476	Evl	1097.9634	1138.13	286.107	431.46	738.4	-1.638384	4E-13	6.8E-12
ENSRNOG00000008956	Cdkn2c	1235.2088	1339.58	339.313	487.34	850.4	-1.637911	2.9E-14	5.8E-13
ENSRNOG00000015713	Parva	13770.978	14333.5	4064.72	4966.8	9284	-1.637644	1.4E-24	8.1E-23
ENSRNOG00000050258	Ccnd3	7248.2093	7140.29	1964.6	2662	4754	-1.636611	3.5E-20	1.3E-18
ENSRNOG00000033335	Cenpi	1204.2512	1290.78	275.064	526.73	824.2	-1.636262	3.1E-10	3.5E-09
ENSRNOG00000020480	Fads1	4576.5672	4891.04	1222.73	1822	3128	-1.636239	1E-16	2.7E-15
ENSRNOG00000011521	Filip1	123.83045	187.957	60.233	40.306	103.1	-1.635604	6.6E-05	0.00029
ENSRNOG00000023410	Apol9a	77.394034	75.8059	19.0738	30.23	50.63	-1.631717	0.00202	0.00628
ENSRNOG00000006198	Prr11	626.37572	666.677	170.66	246.42	427.5	-1.630573	3E-11	3.9E-10
ENSRNOG00000008911	Draxin	182.64992	188.996	51.1981	68.704	122.9	-1.629749	5E-06	2.7E-05

ENSRNOG00000020084	Pcdhb5	725.44008	717.56	206.8	261.07	477.7	-1.623652	4.7E-13	8E-12
ENSRNOG00000009173	Smad6	679.00366	673.946	161.625	278.48	448.3	-1.618026	1.1E-09	1.1E-08
ENSRNOG00000022932	Serhl2	192.96913	164.073	41.1592	75.116	118.3	-1.614538	3.5E-05	0.00016
ENSRNOG00000007728	Gsdmd	2478.6729	2379.06	648.509	937.12	1611	-1.61449	7E-16	1.7E-14
ENSRNOG00000015904	Wfdc1	4179.2778	3860.91	1283.97	1342.9	2667	-1.613786	3E-24	1.7E-22
ENSRNOG00000014290	Grm1	48.500261	63.3447	21.0816	15.573	37.12	-1.61207	0.00773	0.02047
ENSRNOG00000070027	ENSRNOG0000007002	356.01256	379.03	84.3262	155.73	243.8	-1.611314	2.8E-07	1.9E-06
ENSRNOG00000071032	Mmp25	121.76661	150.573	21.0816	67.788	90.3	-1.609624	0.00112	0.0037
ENSRNOG00000006756	Maged1	15043.336	14989.8	4700.18	5141.8	9969	-1.609483	1E-27	8.6E-26
ENSRNOG00000022911	Hjurp	2289.8315	2519.25	499.934	1075.4	1596	-1.609215	1.7E-09	1.7E-08
ENSRNOG00000002418	Tgfb2	16766.644	16951.4	4462.26	6589.1	11192	-1.609163	1.2E-17	3.6E-16
ENSRNOG00000030776	Sytl2	4473.3752	4508.89	1156.47	1787.2	2981	-1.608993	2E-15	4.6E-14
ENSRNOG00000052506	Ak3	2251.6504	2453.83	576.229	965.52	1562	-1.608936	9.1E-13	1.5E-11
ENSRNOG00000025981	Ska2	248.69283	241.956	79.3068	81.528	162.9	-1.608896	2.2E-07	1.5E-06
ENSRNOG00000067207	ENSRNOG0000006720	52.627943	38.4222	4.01553	25.649	30.18	-1.608152	0.03968	0.08327
ENSRNOG00000037199	Spink8	59.851386	58.1525	11.0427	27.481	39.13	-1.608115	0.00981	0.02512
ENSRNOG00000027444	Nubpl	263.13972	259.609	72.2796	98.933	173.5	-1.608077	3.1E-07	2.1E-06
ENSRNOG00000011321	Rftn1	83.585557	112.151	19.0738	44.886	64.92	-1.607814	0.0018	0.00568
ENSRNOG00000048706	Nox1	263.13972	268.955	77.299	97.101	176.6	-1.607588	1.6E-07	1.2E-06
ENSRNOG00000024874	Prtfdc1	978.26059	956.401	259.002	375.58	642.3	-1.606762	1E-12	1.7E-11
ENSRNOG00000010284	St3gal5	1006.1224	1067.51	340.317	341.69	688.9	-1.604285	2.1E-16	5.3E-15
ENSRNOG00000019257	Kif27	33.021455	37.3837	15.0583	8.2444	23.43	-1.600466	0.03409	0.07334

ENSRNOG00000050675	Myl4	138.27734	125.651	28.1087	58.627	87.67	-1.600458	0.00032	0.00121
ENSRNOG00000013340	Twsg1	10393.503	10424.9	3144.16	3727.4	6922	-1.599015	2.4E-24	1.4E-22
ENSRNOG00000068717	Ccdc167	181.618	176.534	51.1981	66.872	119.1	-1.598831	8.8E-06	4.6E-05
ENSRNOG00000004160	ENSRNOG0000000416	1405.4757	1266.89	362.402	519.4	888.5	-1.598483	9.8E-14	1.8E-12
ENSRNOG00000014783	Pgghg	603.67347	645.908	143.555	269.32	415.6	-1.595321	1.6E-08	1.4E-07
ENSRNOG00000049906	Foxf1	1583.9979	1793.38	502.946	615.59	1124	-1.593654	5.9E-17	1.6E-15
ENSRNOG00000003927	Cd55	899.83464	973.016	287.111	333.44	623.4	-1.592885	9.6E-15	2E-13
ENSRNOG00000012946	Mov10	194.00105	224.302	66.2563	72.368	139.2	-1.592644	1.9E-06	1.1E-05
ENSRNOG00000006921	Rbl1	1951.3616	2098.68	486.884	855.59	1348	-1.592082	1.3E-11	1.8E-10
ENSRNOG00000062598	ENSRNOG0000006259	367.36368	389.414	113.439	137.41	251.9	-1.591759	3.2E-09	3.1E-08
ENSRNOG00000047699	Snai2	1336.337	1471.47	359.39	571.61	934.7	-1.591365	3E-12	4.5E-11
ENSRNOG00000000614	Bicc1	1403.4118	1330.24	396.534	510.24	910.1	-1.591128	1.2E-15	2.7E-14
ENSRNOG00000012196	Asah2	437.53427	444.451	117.454	174.97	293.6	-1.590464	5.5E-09	5.1E-08
ENSRNOG00000057713	Cav2	1953.4254	2030.14	593.295	731.92	1327	-1.587225	4.2E-18	1.3E-16
ENSRNOG00000006557	Cyfip2	78.425955	75.8059	20.0777	31.146	51.36	-1.586526	0.00243	0.00741
ENSRNOG00000020441	Vps25	3125.6871	3297.04	861.332	1278.8	2141	-1.58489	2.5E-15	5.5E-14
ENSRNOG00000012292	Alg8	446.82156	468.335	137.532	167.64	305.1	-1.583149	3.7E-10	4.1E-09
ENSRNOG00000032446	Recql4	1227.9853	1291.82	332.285	508.41	840.1	-1.582385	1.6E-12	2.5E-11
ENSRNOG00000009354	Nrarp	104.22397	140.189	31.1204	50.383	81.48	-1.580727	0.00036	0.00133
ENSRNOG00000025302	Cdca2	1689.2538	1778.84	484.876	675.13	1157	-1.579119	5E-15	1.1E-13
ENSRNOG00000006966	Nfia	551.04552	616.832	128.497	261.99	389.6	-1.578049	1.3E-07	9.9E-07
ENSRNOG00000006384	Ddx58	318.86342	281.416	88.3418	112.67	200.3	-1.576666	1E-07	7.7E-07

ENSRNOG00000065898	ENSRNOG0000006589	37.149136	34.2684	12.0466	11.909	23.84	-1.576066	0.03138	0.06849
ENSRNOG00000022499	Sgo1	1380.7096	1372.81	418.619	504.74	919.2	-1.575607	1.6E-16	4E-15
ENSRNOG00000029598	Robo2	4136.9691	4211.9	1119.33	1683.7	2788	-1.57412	2.5E-15	5.5E-14
ENSRNOG00000042729	Efhc1	71.202511	38.4222	11.0427	25.649	36.58	-1.573119	0.01719	0.04089
ENSRNOG00000023334	Parp14	721.3124	740.406	235.913	255.58	488.3	-1.571982	2E-13	3.6E-12
ENSRNOG00000043044	Cnn2	22311.152	22054.3	6855.52	8076.8	14824	-1.570937	1.3E-24	7.9E-23
ENSRNOG00000046764	Tmprss11a	99.064364	99.69	31.1204	35.726	66.4	-1.570866	0.00057	0.00201
ENSRNOG00000043128	Armcx4	2837.7813	3102.85	636.462	1363.1	1985	-1.570259	2.5E-09	2.5E-08
ENSRNOG00000013994	Enpp1	1919.372	1880.61	569.202	709.94	1270	-1.570176	2.1E-17	6E-16
ENSRNOG00000014443	Pde5a	322.9911	268.955	67.2602	131.91	197.8	-1.568025	4.8E-06	2.6E-05
ENSRNOG00000016408	Kirrel1	4771.6002	4840.16	1344.2	1897.1	3213	-1.567818	5.6E-17	1.5E-15
ENSRNOG00000000561	Pald1	373.5552	357.222	90.3495	155.73	244.2	-1.56742	2.6E-07	1.8E-06
ENSRNOG00000012318	Aspm	4310.3317	4314.71	1141.42	1768.9	2884	-1.566892	1.3E-14	2.7E-13
ENSRNOG00000052634	Lsmem2	46.43642	67.4984	22.0854	16.489	38.13	-1.564915	0.00932	0.02402
ENSRNOG00000003815	Slc25a11	1303.3155	1403.97	327.266	587.19	905.4	-1.564549	2.5E-10	2.8E-09
ENSRNOG00000007687	Sema7a	45.4045	70.6137	19.0738	20.153	38.81	-1.563847	0.00879	0.02286
ENSRNOG00000018358	Nt5dc2	1876.0314	1859.84	473.833	792.38	1251	-1.559931	7.2E-12	1E-10
ENSRNOG00000012427	Veph1	213.60753	176.534	56.2175	76.032	130.6	-1.558557	1E-05	5.3E-05
ENSRNOG00000011585	Fat3	149.62847	138.112	36.1398	61.375	96.31	-1.557239	0.00015	0.0006
ENSRNOG00000002180	Tbc1d1	5669.371	5766.44	1923.44	1971.3	3833	-1.553909	9.7E-25	5.9E-23
ENSRNOG00000004281	Cobl	2920.3349	2999.01	949.674	1066.3	1984	-1.553701	5.9E-21	2.4E-19
ENSRNOG00000018044	Phyh	496.35374	506.757	121.47	219.85	336.1	-1.552714	7.1E-08	5.5E-07

ENSRNOG00000064429	Snx21	1000.9628	917.978	245.951	407.64	643.1	-1.552252	2.6E-10	3E-09
ENSRNOG00000021151	Ppp1r14b	3734.5201	3464.23	996.856	1458.4	2413	-1.551391	3.5E-15	7.7E-14
ENSRNOG00000039183	Cip2a	1380.7096	1285.59	422.635	487.34	894.1	-1.550397	3.1E-16	7.6E-15
ENSRNOG00000020601	Hmg20b	8904.4416	9278.44	2507.7	3702.7	6098	-1.549604	4.4E-16	1.1E-14
ENSRNOG00000015442	Sfxn3	3298.0178	3395.69	849.285	1440	2246	-1.547288	2.7E-12	4.1E-11
ENSRNOG00000010105	S100a11	24469.93	23341	8010.99	8348	16042	-1.547243	7.3E-27	5.4E-25
ENSRNOG00000005041	Crip2	156.85191	150.573	37.1437	67.788	103.1	-1.546664	0.00014	0.00057
ENSRNOG00000051528	Ddx11	1148.5275	1254.43	328.27	493.75	806.2	-1.546309	4.9E-12	7.3E-11
ENSRNOG00000063590	Fzd4	1014.3778	993.784	203.788	483.67	673.9	-1.544833	2.8E-07	1.9E-06
ENSRNOG00000009660	Enpp6	78.425955	76.8443	35.1359	18.321	52.18	-1.543401	0.0038	0.01104
ENSRNOG00000020782	Pspc1	875.06855	889.941	223.866	381.08	592.5	-1.543072	8.5E-10	9E-09
ENSRNOG00000018225	Tp53inp2	5987.2025	5782.02	2002.75	2037.3	3952	-1.542554	2E-24	1.2E-22
ENSRNOG00000011332	Clspn	1381.7415	1395.66	316.223	636.65	932.6	-1.54207	5.7E-09	5.3E-08
ENSRNOG00000002746	Fstl1	36827.177	36140.7	11367	13691	24507	-1.541936	3.1E-23	1.6E-21
ENSRNOG00000019778	ENSRNOG0000001977	3429.0717	3023.93	1031.99	1184.5	2167	-1.541454	1.6E-19	5.8E-18
ENSRNOG00000016117	Myof	32529.229	32686.9	8730.78	13678	21906	-1.541128	5.4E-15	1.2E-13
ENSRNOG00000031916	Timeless	2286.7357	2210.83	468.814	1077.3	1511	-1.539666	3.1E-08	2.6E-07
ENSRNOG00000019560	Pde2a	46.43642	68.5369	10.0388	29.314	38.58	-1.539524	0.01792	0.04245
ENSRNOG00000013968	Comtd1	91.84092	80.9981	31.1204	28.398	58.09	-1.538859	0.00148	0.00476
ENSRNOG00000012349	Tlnrd1	1270.2941	1220.16	335.297	521.23	836.7	-1.538572	1E-11	1.5E-10
ENSRNOG00000007561	Glb1l2	3289.7624	3141.27	870.367	1342.9	2161	-1.538266	8.7E-14	1.6E-12
ENSRNOG00000023807	Ccnyl1	4599.2695	4633.51	1580.11	1599.4	3103	-1.537927	1.3E-23	6.9E-22

ENSRNOG00000018949	Dlk2	46.43642	53.9987	19.0738	15.573	33.77	-1.537246	0.01371	0.03356
ENSRNOG00000067472	ENSRNOG0000006747	46.43642	31.1531	12.0466	14.657	26.07	-1.537208	0.03103	0.06782
ENSRNOG00000068686	ENSRNOG0000006868	63.979068	73.729	17.066	30.23	46.25	-1.537205	0.00575	0.01587
ENSRNOG00000036833	Zfp385a	1545.8168	1617.89	499.934	590.85	1064	-1.535672	1E-16	2.7E-15
ENSRNOG00000011195	Prkra	1538.5934	1560.77	443.717	624.75	1042	-1.535472	7.7E-14	1.4E-12
ENSRNOG00000029862	Spc24	548.98168	552.449	138.536	240.92	370.2	-1.535029	3E-08	2.5E-07
ENSRNOG00000019422	Egr1	1016.4416	1040.51	404.565	305.96	691.9	-1.534662	3.6E-13	6.3E-12
ENSRNOG00000039472	AC131360.2	123.83045	111.113	37.1437	43.97	79.01	-1.532912	0.00029	0.00112
ENSRNOG00000002802	Cxcl1	395.22553	403.952	155.602	120.92	268.9	-1.532716	8.4E-09	7.6E-08
ENSRNOG00000037518	Hyi	78.425955	52.9603	15.0583	30.23	44.17	-1.531493	0.00908	0.02351
ENSRNOG00000067290	ENSRNOG0000006729	45.4045	63.3447	9.03495	28.398	36.55	-1.531019	0.02183	0.0504
ENSRNOG00000014603	Sgcg	725.44008	752.867	243.944	267.49	497.4	-1.530836	7E-13	1.2E-11
ENSRNOG00000012095	Pkia	2837.7813	2725.9	757.932	1168.9	1873	-1.529161	1.9E-13	3.5E-12
ENSRNOG00000017045	Zdhhc1	268.29932	232.61	64.2485	109.01	168.5	-1.528446	6.7E-06	3.6E-05
ENSRNOG00000029401	Actg2	82184.209	80974.2	25616.1	31009	54946	-1.526737	7.1E-23	3.5E-21
ENSRNOG00000020776	Dhcr7	457.14076	463.143	127.493	191.45	309.8	-1.526595	1.5E-08	1.3E-07
ENSRNOG00000068374	Insyn1	444.75772	438.22	105.408	200.61	297.3	-1.525954	4.8E-07	3.1E-06
ENSRNOG00000020119	ENSRNOG0000002011	2115.4369	2147.49	643.489	836.35	1436	-1.525751	3.4E-16	8.4E-15
ENSRNOG00000007613	C1qtnf5	1323.9539	1254.43	364.41	530.39	868.3	-1.525694	1.6E-12	2.5E-11
ENSRNOG00000011774	Fblim1	1807.9246	1687.46	514.992	698.95	1177	-1.524947	1.5E-14	3.1E-13
ENSRNOG00000002171	Phldb2	14865.846	15032.4	4303.65	6090.8	10073	-1.524114	4.4E-17	1.2E-15
ENSRNOG00000002607	Sox9	524.21559	560.756	186.722	190.54	365.6	-1.523888	5.6E-11	7.1E-10

ENSRNOG00000033787	Adamtsl5	1445.7206	1432	376.456	623.83	969.5	-1.523333	5E-11	6.4E-10
ENSRNOG00000052025	Tle5	41621.48	41808.5	12254.4	16783	28117	-1.52261	2.2E-18	7.1E-17
ENSRNOG00000060146	Six5	812.1214	847.365	175.68	401.23	559.1	-1.52239	4.1E-07	2.7E-06
ENSRNOG00000062678	H19	46980.243	45746.3	13730.1	18574	31258	-1.521231	1E-18	3.3E-17
ENSRNOG00000070119	Pbx1	11151.964	11608.7	3296.75	4634.3	7673	-1.520789	5.5E-17	1.5E-15
ENSRNOG00000015496	Tpm4	63420.799	63291.7	19227.4	24953	42723	-1.520047	3.5E-20	1.3E-18
ENSRNOG00000013481	Cdh11	6587.7802	6512.04	1654.4	2917.6	4418	-1.518324	8.8E-12	1.3E-10
ENSRNOG00000009066	Thra	755.36577	765.328	200.777	329.78	512.8	-1.517326	1.8E-09	1.8E-08
ENSRNOG00000069334	ENSRNOG0000006933	234.24594	188.996	80.3107	67.788	142.8	-1.516215	6.4E-06	3.4E-05
ENSRNOG00000019356	Lrfn4	1819.2758	1877.49	579.241	713.6	1247	-1.515108	1.9E-16	4.9E-15
ENSRNOG00000020310	Grik5	612.96075	687.445	192.746	261.99	438.8	-1.51437	2.3E-10	2.7E-09
ENSRNOG00000059947	Sdc1	10864.059	10644	3158.22	4374.1	7260	-1.513528	2.9E-17	8.2E-16
ENSRNOG00000003121	Rtn4rl1	74.298273	84.1134	14.0544	41.222	53.42	-1.512179	0.00784	0.02072
ENSRNOG00000016645	Sfmbt1	615.02459	610.601	156.606	272.98	413.8	-1.510319	2.5E-08	2.1E-07
ENSRNOG00000016377	Cep55	2568.45	2531.71	850.289	941.7	1723	-1.50872	1.7E-19	6E-18
ENSRNOG00000011203	Farp1	4430.0345	4312.63	1228.75	1844.9	2954	-1.507674	2.7E-14	5.4E-13
ENSRNOG00000020022	Psmc3ip	253.85243	249.225	60.233	116.34	169.9	-1.506899	1.8E-05	8.7E-05
ENSRNOG00000067799	Selenow	5004.8142	4524.47	1548.99	1803.7	3220	-1.506834	5.6E-20	2.1E-18
ENSRNOG00000004854	Has2	267.2674	260.648	79.3068	106.26	178.4	-1.506342	1.1E-06	6.7E-06
ENSRNOG00000069779	ENSRNOG0000006977	75.330193	47.7681	25.0971	18.321	41.63	-1.506127	0.00991	0.02535
ENSRNOG00000058288	Tcf19	2010.181	2089.34	396.534	1047	1386	-1.505606	0.02038	0.04748
ENSRNOG00000008595	Ttc12	195.03297	155.766	63.2447	60.459	118.6	-1.504149	2.8E-05	0.00013

ENSRNOG00000028236	RGD1309104	1610.8278	1644.88	451.748	695.28	1101	-1.504059	4.4E-12	6.5E-11
ENSRNOG00000071138	ENSRNOG0000007113	10655.611	10219.3	3188.33	4174.4	7059	-1.503284	1.4E-18	4.6E-17
ENSRNOG00000000091	Smad9	619.15227	682.253	165.641	293.14	440	-1.502113	3.2E-08	2.7E-07
ENSRNOG00000004839	B4galnt1	313.70382	322.954	95.3689	129.16	215.3	-1.501625	2.3E-07	1.6E-06
ENSRNOG00000013949	Idh2	6843.6965	6822.53	1783.9	3042.2	4623	-1.501382	4E-12	5.9E-11
ENSRNOG00000040101	AABR07026805.2	156.85191	83.075	50.1942	34.81	81.23	-1.499549	0.00128	0.00416
ENSRNOG00000018214	Bok	1523.1146	1429.93	389.507	655.89	999.6	-1.497009	1.7E-10	2E-09
ENSRNOG00000027736	Cnn1	12608.004	12198.5	4115.92	4672.8	8399	-1.496918	6.2E-23	3.1E-21
ENSRNOG00000017512	Aldh3b1	165.10727	203.534	57.2214	73.284	124.8	-1.496245	2.7E-05	0.00013
ENSRNOG00000005034	Arhgef25	2217.5971	2542.09	690.672	995.75	1612	-1.496228	1.9E-13	3.5E-12
ENSRNOG00000027837	ENSRNOG0000002783	390.06593	420.567	143.555	143.82	274.5	-1.496086	4.8E-09	4.5E-08
ENSRNOG00000016592	Gnai2	15379.742	15196.5	5307.53	5538.4	10356	-1.495227	5.3E-25	3.3E-23
ENSRNOG00000038478	AABR07029613.1	41.276818	32.1916	16.0621	10.077	24.9	-1.495015	0.0402	0.08422
ENSRNOG00000008165	Tpx2	5771.5311	5864.05	1795.95	2331.3	3941	-1.495001	5.1E-18	1.6E-16
ENSRNOG00000058891	Cys1	88.745159	91.3825	28.1087	35.726	60.99	-1.49459	0.00165	0.00524
ENSRNOG00000012818	Ksr1	722.34432	694.714	188.73	314.2	480	-1.492559	6.7E-09	6.1E-08
ENSRNOG00000070694	ENSRNOG0000007069	2005.0214	1935.65	535.07	864.75	1335	-1.492309	1.5E-11	2E-10
ENSRNOG00000024650	Ckap2	2250.6185	2378.02	666.579	978.34	1568	-1.491846	2.1E-13	3.8E-12
ENSRNOG00000000579	Marcks	5801.4568	5897.28	1904.37	2258.1	3965	-1.49065	2.1E-20	8.3E-19
ENSRNOG00000018570	C1qtnf3	145.50078	145.381	27.1049	76.032	98.5	-1.49051	0.00124	0.00406
ENSRNOG00000023338	Tspan2	947.30298	925.247	259.002	406.73	634.6	-1.490489	3.2E-10	3.6E-09
ENSRNOG00000014574	Entpd1	2263.0016	2397.75	762.952	896.81	1580	-1.489171	1.5E-17	4.3E-16

ENSRNOG00000001314	Fam20c	2714.9827	2648.01	741.87	1168	1818	-1.488916	2E-12	3E-11
ENSRNOG00000068306	ENSRNOG0000006830	208.44793	199.38	55.2136	89.773	138.2	-1.48884	2.8E-05	0.00013
ENSRNOG00000007610	Gdf11	919.44113	898.248	280.084	368.25	616.5	-1.486151	5.1E-12	7.6E-11
ENSRNOG00000053086	Selenop	206.38409	155.766	51.1981	77.864	122.8	-1.485763	7.2E-05	0.00032
ENSRNOG00000019189	Acat2	1797.6054	1957.45	639.474	701.69	1274	-1.485053	3.4E-17	9.3E-16
ENSRNOG00000004278	Dlx3	90.809	83.075	29.1126	32.978	58.99	-1.484648	0.00196	0.00613
ENSRNOG00000014721	Ahrr	2235.1397	2216.02	539.085	1050.7	1510	-1.484501	2.9E-09	2.9E-08
ENSRNOG00000020918	Ccnd1	14398.386	13941	3892.06	6242.9	9619	-1.483327	2.8E-13	4.8E-12
ENSRNOG00000008016	Ckap4	22420.536	21618.2	6945.87	8805.1	14947	-1.483261	1.2E-19	4.3E-18
ENSRNOG00000018830	Aff3	1197.0277	1521.31	353.367	618.33	922.5	-1.482976	3.4E-09	3.3E-08
ENSRNOG00000001250	Lfng	2785.1533	2830.78	958.709	1050.7	1906	-1.48253	1.8E-19	6.5E-18
ENSRNOG00000017850	Dctpp1	390.06593	345.8	105.408	157.56	249.7	-1.482309	2.7E-07	1.9E-06
ENSRNOG00000020702	Cyb561a3	858.55782	926.286	264.021	374.66	605.9	-1.481265	5.8E-11	7.3E-10
ENSRNOG00000028624	Kif26b	3422.8801	3366.61	926.585	1508.7	2306	-1.478636	5E-12	7.3E-11
ENSRNOG00000000168	Gatm	953.4945	958.477	276.068	409.47	649.4	-1.478352	8.8E-11	1.1E-09
ENSRNOG00000038916	Dram1	1389.9969	1436.16	405.569	608.26	960	-1.477952	9.5E-12	1.3E-10
ENSRNOG00000025705	Armcx2	4277.3103	4233.71	1300.03	1757	2892	-1.476824	4.2E-16	1E-14
ENSRNOG00000007590	Eya1	2177.3522	2297.02	699.707	908.72	1521	-1.475431	2.4E-15	5.4E-14
ENSRNOG00000000866	ENSRNOG0000000086	1533.4338	1535.85	602.33	502.91	1044	-1.474141	2.3E-15	5.2E-14
ENSRNOG00000019243	Glb1l	1307.4432	1314.66	417.616	525.81	891.4	-1.473935	5.7E-14	1.1E-12
ENSRNOG00000015736	Dhrs3	60.883307	71.6522	21.0816	26.565	45.05	-1.473914	0.00697	0.01873
ENSRNOG00000015473	Phactr2	239.40555	234.687	76.2952	94.353	161.2	-1.472617	3.1E-06	1.7E-05

ENSRNOG00000045846	Rfx2	92.872841	90.344	32.1243	33.894	62.31	-1.472171	0.00158	0.00505
ENSRNOG00000010311	Dzip1	1142.3359	1157.86	353.367	475.43	782.2	-1.47162	1.6E-12	2.6E-11
ENSRNOG00000046660	Gpc6	3557.0298	3549.38	1078.17	1485.8	2418	-1.470259	3.2E-15	7.1E-14
ENSRNOG00000018250	Tnni3	43.340659	38.4222	12.0466	17.405	27.8	-1.469958	0.03199	0.06955
ENSRNOG00000061795	Rbm4b	715.12088	652.139	227.882	265.65	465.2	-1.469282	3.4E-11	4.4E-10
ENSRNOG00000006359	Galk1	1012.314	928.363	254.986	445.2	660.2	-1.469198	6.1E-09	5.6E-08
ENSRNOG00000026502	Dscc1	732.66352	807.904	186.722	369.17	524.1	-1.468667	1.7E-07	1.3E-06
ENSRNOG00000001795	Itgb5	20971.719	20173.7	6866.56	8001.7	14003	-1.46844	7.4E-22	3.3E-20
ENSRNOG00000015756	Nphp1	552.07744	531.68	147.571	243.67	368.7	-1.467749	5.5E-08	4.4E-07
ENSRNOG00000031700	Map3k5	477.77917	509.873	118.458	238.17	336.1	-1.466983	1.4E-06	8.6E-06
ENSRNOG00000019504	Adprhl1	2274.3527	2349.98	761.948	911.47	1574	-1.466001	7E-17	1.9E-15
ENSRNOG00000020058	Celsr2	350.85295	332.3	103.4	143.82	232.6	-1.464408	2.9E-07	2E-06
ENSRNOG00000026124	Id3	6023.3197	5688.56	1704.59	2539.3	3989	-1.464198	5.9E-14	1.1E-12
ENSRNOG00000015921	Esco2	792.51491	805.827	195.757	382.91	544.3	-1.4639	1.2E-07	9E-07
ENSRNOG00000000692	Ung	556.20513	542.064	157.61	240	374	-1.463832	1.5E-08	1.3E-07
ENSRNOG00000004805	Stac2	85.649398	69.5753	25.0971	31.146	52.87	-1.46288	0.00406	0.0117
ENSRNOG00000062882	Hey1	44.37258	32.1916	14.0544	13.741	26.09	-1.462123	0.03814	0.08067
ENSRNOG00000018232	Srf	5072.921	4880.65	1613.24	1999.7	3392	-1.461756	3.7E-18	1.2E-16
ENSRNOG00000001825	Pkp2	346.72527	298.031	111.431	122.75	219.7	-1.46051	2E-07	1.4E-06
ENSRNOG00000005935	A3galt2	2340.3956	2172.41	758.936	881.24	1538	-1.459797	6.6E-17	1.8E-15
ENSRNOG00000059622	Pigb	114.54317	104.882	30.1165	49.467	74.75	-1.459517	0.00116	0.00382
ENSRNOG00000008666	Etl4	927.69649	1062.32	306.184	417.72	678.5	-1.457773	3.1E-11	4E-10

ENSRNOG00000066220	ENSRNOG0000006622	48.500261	41.5375	6.0233	26.565	30.66	-1.457522	0.04863	0.09867
ENSRNOG00000004430	Cep131	545.88592	561.794	156.606	247.33	377.9	-1.453295	3.2E-08	2.6E-07
ENSRNOG00000008517	Cdc42ep1	1120.6656	1086.21	395.53	410.39	753.2	-1.453137	2.3E-14	4.5E-13
ENSRNOG00000013911	Nagk	938.01569	1015.59	297.15	415.89	666.7	-1.452863	4.5E-11	5.8E-10
ENSRNOG00000031824	Slc44a2	3595.2109	3783.03	1117.32	1577.4	2518	-1.452662	2.3E-14	4.7E-13
ENSRNOG00000005917	Pawr	1849.2015	1885.8	536.074	828.11	1275	-1.452191	1.3E-11	1.8E-10
ENSRNOG00000018615	Cenph	1235.2088	1203.55	331.282	559.71	832.4	-1.451376	1.3E-09	1.3E-08
ENSRNOG00000021176	Mtmr11	1083.5165	976.131	344.332	408.56	703.1	-1.451198	7.8E-13	1.3E-11
ENSRNOG00000018075	Spcs1	2394.0555	2362.44	834.227	906.89	1624	-1.449668	4.2E-18	1.3E-16
ENSRNOG00000069551	ENSRNOG0000006955	53.659864	50.8834	19.0738	19.237	35.71	-1.448216	0.01613	0.0386
ENSRNOG00000014863	Reck	1282.6771	1245.09	398.542	527.64	863.5	-1.447558	1E-12	1.7E-11
ENSRNOG00000015068	Il11ra1	913.2496	776.751	221.858	397.57	577.4	-1.446362	4.5E-08	3.6E-07
ENSRNOG00000008246	Emilin1	5839.6379	6183.89	1978.65	2433	4109	-1.446237	3.1E-18	9.7E-17
ENSRNOG00000055082	Maz	6364.8854	6545.27	1929.46	2807.7	4412	-1.446128	2.6E-14	5.2E-13
ENSRNOG00000020937	Ubtf	4838.675	4736.31	1497.79	2017.1	3272	-1.445434	9.5E-16	2.2E-14
ENSRNOG00000033348	Duox1	847.20669	873.326	286.107	345.35	588	-1.445238	5.2E-12	7.7E-11
ENSRNOG00000019143	Galnt2	8147.012	7966.89	2387.24	3533.2	5509	-1.444296	4.4E-14	8.5E-13
ENSRNOG00000008851	Slc35b4	4829.3877	4881.69	1544.98	2023.6	3320	-1.443984	2E-16	5E-15
ENSRNOG00000018836	RGD1559896	20303.035	19751.1	6305.39	8421.2	13695	-1.443435	3.8E-17	1E-15
ENSRNOG00000046889	Dbi	4098.788	3741.49	1351.23	1536.2	2682	-1.440903	1.6E-18	5.3E-17
ENSRNOG00000000130	Dnajb5	2641.7164	2713.44	852.297	1120.3	1832	-1.440282	4.8E-15	1E-13
ENSRNOG00000018106	Neu3	262.1078	250.263	96.3728	92.521	175.3	-1.439919	1.6E-06	9.7E-06

ENSRNOG00000002886	Myh10	8316.2469	8844.37	2956.44	3375.6	5873	-1.438235	2.1E-20	8E-19
ENSRNOG00000064090	Tmsb10	32585.984	30729.4	11685.2	11682	21671	-1.438049	1E-23	5.6E-22
ENSRNOG00000015895	B4galt6	1191.8681	1164.09	358.386	511.16	806.4	-1.436868	2.3E-11	3.1E-10
ENSRNOG00000019316	Sh3bp4	6020.2239	5759.17	1826.06	2524.6	4033	-1.436659	3.6E-15	7.8E-14
ENSRNOG00000002196	Ociad2	608.83307	526.488	190.738	229.01	388.8	-1.434553	1.5E-09	1.5E-08
ENSRNOG00000007234	Cyp51	4778.8236	5005.27	1688.53	1932.9	3351	-1.4337	2.9E-19	1E-17
ENSRNOG00000009045	Alg6	595.4181	592.948	177.687	261.99	407	-1.432695	9.4E-09	8.4E-08
ENSRNOG00000016055	Fkrp	560.33281	562.833	155.602	260.16	384.7	-1.431635	1.1E-07	7.9E-07
ENSRNOG00000011431	Mfhas1	1337.3689	1240.93	347.344	608.26	883.5	-1.430709	4.6E-09	4.3E-08
ENSRNOG00000026573	Ophn1	1100.0272	1002.09	336.301	443.37	720.4	-1.429913	1.2E-11	1.7E-10
ENSRNOG00000018184	Tpm1	63176.234	63637.5	20713.1	26370	43474	-1.429394	1.3E-18	4.2E-17
ENSRNOG00000028701	C8g	142.40502	179.65	33.1282	86.109	110.3	-1.428685	0.00124	0.00406
ENSRNOG00000001309	Camkk2	657.33333	565.948	174.676	279.4	419.3	-1.427913	5.2E-08	4.1E-07
ENSRNOG00000045670	Tnfsf12	269.33124	269.994	80.3107	120	184.9	-1.426401	5.4E-06	2.9E-05
ENSRNOG00000024931	Ccdc88b	48.500261	51.9219	19.0738	18.321	34.45	-1.425497	0.01982	0.04628
ENSRNOG00000014589	Arpin	1037.0801	1044.67	315.219	459.86	714.2	-1.424137	1.4E-10	1.7E-09
ENSRNOG00000016689	Fanci	2041.1387	1930.45	529.047	950.86	1363	-1.423336	2.5E-09	2.4E-08
ENSRNOG00000067628	Olr812	143.43694	128.766	50.1942	51.299	93.42	-1.423159	0.00026	0.00101
ENSRNOG00000001627	Abi3bp	741.95081	670.83	265.025	261.99	484.9	-1.422696	3.5E-11	4.5E-10
ENSRNOG00000017704	Sema3f	1132.0167	1170.32	425.647	433.29	790.3	-1.422393	3.8E-14	7.4E-13
ENSRNOG00000060914	Nudcd2	1337.3689	1232.62	366.418	591.77	882	-1.422215	8.2E-10	8.7E-09
ENSRNOG00000011417	Pde3b	1102.091	1055.05	323.251	481.84	740.6	-1.420654	2.3E-10	2.7E-09

ENSRNOG00000001726	Tmem44	150.66039	146.42	40.1553	70.536	101.9	-1.420557	0.00042	0.00156
ENSRNOG00000027430	Ikzf2	37.149136	73.729	24.0932	17.405	38.09	-1.420148	0.02289	0.05252
ENSRNOG00000019181	Myoz3	4858.2815	4893.12	1547.99	2102.3	3350	-1.417253	4.7E-15	1E-13
ENSRNOG00000048043	F2r	27689.522	29110.5	9859.14	11414	19518	-1.416821	9.7E-21	3.9E-19
ENSRNOG00000008892	Parp2	1200.1235	1207.7	356.379	545.05	827.3	-1.416257	2.5E-10	2.9E-09
ENSRNOG00000067501	ENSRNOG0000006750	187.80952	209.764	68.2641	80.612	136.6	-1.415866	2.5E-05	0.00012
ENSRNOG00000029055	Ttk	1716.0837	1756	459.779	840.93	1193	-1.415513	6.2E-09	5.7E-08
ENSRNOG00000065112	ENSRNOG0000006511	208.44793	172.381	59.2291	83.36	130.9	-1.414981	6.5E-05	0.00029
ENSRNOG00000045952	Ephb4	1246.5599	1280.39	365.414	581.69	868.5	-1.414614	6.2E-10	6.7E-09
ENSRNOG00000053406	Gramd1c	227.0225	201.457	80.3107	80.612	147.4	-1.412855	1.2E-05	6.2E-05
ENSRNOG00000003954	Il2rg	410.70434	384.222	160.621	138.32	273.5	-1.411884	4.7E-08	3.8E-07
ENSRNOG00000053026	Shcbp1	1768.7117	1625.15	530.051	744.75	1167	-1.411846	4.3E-12	6.4E-11
ENSRNOG00000004165	Prkd1	1343.5604	1563.89	357.383	734.67	999.9	-1.411573	1.7E-07	1.2E-06
ENSRNOG00000000940	ENSRNOG0000000094	715.12088	650.062	266.029	247.33	469.6	-1.411437	8.4E-11	1E-09
ENSRNOG00000062331	ENSRNOG0000006233	695.51439	691.599	240.932	280.31	477.1	-1.41129	9.2E-11	1.1E-09
ENSRNOG00000068968	ENSRNOG0000006896	102.16013	94.4978	26.101	47.635	67.6	-1.410982	0.00301	0.00896
ENSRNOG00000048838	Gjc1	846.17477	788.174	205.796	408.56	562.2	-1.409799	4.1E-07	2.8E-06
ENSRNOG00000062353	ENSRNOG0000006235	53.659864	44.6528	27.1049	10.077	33.87	-1.409678	0.03484	0.0747
ENSRNOG00000016770	Calm1	3789.2119	3541.07	1271.92	1486.7	2522	-1.409634	2.2E-17	6.2E-16
ENSRNOG00000020185	Wdr6	4300.0125	4112.21	1286.98	1882.5	2895	-1.407842	4.4E-13	7.5E-12
ENSRNOG00000010274	Smc4	6760.1109	6963.76	2243.68	2928.6	4724	-1.407586	3.3E-16	8E-15
ENSRNOG00000009360	Sh3bp1	1013.3459	1172.4	359.39	464.44	752.4	-1.406798	2.1E-11	2.9E-10

ENSRNOG00000026136	Tnfaip8	856.49398	886.825	335.297	322.45	600.3	-1.406414	2.6E-12	3.9E-11
ENSRNOG00000015796	Msh2	1700.6049	1725.88	505.957	785.97	1180	-1.406284	9.3E-11	1.1E-09
ENSRNOG00000004400	Avpr1a	10838.261	10761.3	3004.62	5146.4	7438	-1.405773	6.3E-11	8E-10
ENSRNOG00000012438	Larp6	224.95866	272.071	67.2602	120	171.1	-1.405083	4.7E-05	0.00021
ENSRNOG00000037627	Trappc1	1202.1873	1130.86	391.515	489.17	803.4	-1.404719	2.1E-12	3.2E-11
ENSRNOG00000027249	Fancl	232.1821	154.727	56.2175	89.773	133.2	-1.403449	0.0002	0.0008
ENSRNOG00000012561	Arhgef10	1795.5416	1807.92	551.132	810.7	1241	-1.40299	1.4E-11	1.9E-10
ENSRNOG00000003108	Acbd4	275.52276	219.11	75.2913	111.76	170.4	-1.400592	2.1E-05	0.0001
ENSRNOG00000045752	Rrm1	14807.027	14073.9	4361.87	6579.1	9955	-1.400251	3.7E-13	6.4E-12
ENSRNOG00000013179	Tinagl1	570.65201	497.411	170.66	233.59	368.1	-1.400151	2.4E-08	2.1E-07
ENSRNOG00000001518	Itga6	8064.4584	8151.73	2707.47	3436.1	5590	-1.400103	5.2E-17	1.4E-15
ENSRNOG00000056209	Exo1	1047.3993	1032.21	223.866	563.37	716.7	-1.399954	5.9E-06	3.2E-05
ENSRNOG00000025796	Brme1	464.3642	503.642	112.435	253.75	333.5	-1.399872	1.4E-05	7.2E-05
ENSRNOG00000021962	Fzd2	3609.6578	3537.96	1036.01	1671.8	2464	-1.399838	4E-11	5.1E-10
ENSRNOG00000048870	St6galnac4	2893.505	2861.93	985.814	1198.2	1985	-1.397562	5.5E-16	1.3E-14
ENSRNOG00000016423	Tacc1	1855.393	1873.34	671.598	744.75	1286	-1.396201	1E-15	2.3E-14
ENSRNOG00000017477	Mmp23	180.58608	178.611	75.2913	61.375	124	-1.395629	6.3E-05	0.00028
ENSRNOG00000001294	Ift81	986.51595	998.977	327.266	426.88	684.9	-1.395568	2.8E-11	3.8E-10
ENSRNOG00000000894	Fry	2305.3103	2402.94	755.924	1037	1625	-1.392288	4.4E-13	7.5E-12
ENSRNOG00000050325	Adgre4	46.43642	31.1531	13.0505	16.489	26.78	-1.39138	0.04725	0.09626
ENSRNOG00000066650	ENSRNOG0000006665	462.30036	447.566	149.579	196.95	314.1	-1.391165	4.9E-08	3.9E-07
ENSRNOG00000019681	Pold1	1696.4772	1677.08	440.705	844.6	1165	-1.391161	3.4E-08	2.8E-07

ENSRNOG00000060098	Ptms	10486.376	11024	3547.72	4652.6	7428	-1.391132	4.3E-16	1E-14
ENSRNOG00000057470	Pla2g12a	294.09733	307.377	94.3651	134.66	207.6	-1.39084	3E-06	1.7E-05
ENSRNOG00000026842	Nnt	5313.3584	5062.38	1507.83	2450.4	3584	-1.389918	3.8E-11	4.9E-10
ENSRNOG00000017707	Abi2	3536.3914	3806.91	1124.35	1678.2	2536	-1.389224	3.5E-12	5.3E-11
ENSRNOG00000017494	Acp6	1069.0696	1076.86	432.674	387.49	741.5	-1.388076	6.8E-13	1.1E-11
ENSRNOG00000038102	Elmod3	231.15018	339.569	68.2641	149.32	197.1	-1.388023	0.00018	0.00071
ENSRNOG00000050453	AABR07002848.1	72.234432	73.729	31.1204	24.733	50.45	-1.387765	0.00705	0.01891
ENSRNOG00000004292	Sptssa	1073.1973	979.246	322.247	461.69	709.1	-1.387364	3.9E-10	4.3E-09
ENSRNOG00000011271	Mcc	1404.4437	1421.62	483.872	599.1	977.3	-1.383115	4.8E-13	8.1E-12
ENSRNOG00000018929	Kif20b	4372.247	4469.43	1535.94	1856.8	3059	-1.381591	8.5E-17	2.2E-15
ENSRNOG00000016640	Dner	374.58713	396.683	123.478	172.22	266.7	-1.381252	4.4E-07	2.9E-06
ENSRNOG00000021242	Adam33	1666.5515	1908.65	517	854.67	1237	-1.381193	1.7E-09	1.7E-08
ENSRNOG00000015257	Coro2b	621.21611	649.023	176.684	310.54	439.4	-1.380463	3.1E-07	2.1E-06
ENSRNOG00000009625	Dpysl2	3695.3071	3854.68	1269.91	1629.7	2612	-1.380275	4E-15	8.7E-14
ENSRNOG00000017283	Kcnt1	33.021455	46.7297	15.0583	15.573	27.6	-1.380125	0.0443	0.0913
ENSRNOG00000066721	ENSRNOG0000006672	101.1282	100.728	25.0971	52.215	69.79	-1.379737	0.00412	0.01185
ENSRNOG00000057458	Oip5	580.97122	665.638	149.579	328.86	431.3	-1.37946	7.2E-06	3.8E-05
ENSRNOG00000004903	Ebp	751.23809	750.79	273.056	304.13	519.8	-1.379276	5.6E-11	7.1E-10
ENSRNOG00000013166	Wnt4	265.20356	277.263	100.388	108.09	187.7	-1.379086	2.4E-06	1.4E-05
ENSRNOG00000004821	Sntb1	415.86394	381.106	114.443	191.45	275.7	-1.379062	2.3E-06	1.3E-05
ENSRNOG00000038600	Dnaaf3	264.17164	287.647	99.3845	112.67	191	-1.37886	2.5E-06	1.4E-05
ENSRNOG00000023778	Gcnt2	496.35374	561.794	176.684	229.93	366.2	-1.378459	2.1E-08	1.8E-07

ENSRNOG00000020554	Slc50a1	489.1303	406.029	143.555	201.53	310.1	-1.373513	3E-07	2.1E-06
ENSRNOG00000011697	Zfp827	1398.2522	1423.7	461.786	627.49	977.8	-1.372439	1.2E-11	1.6E-10
ENSRNOG00000027839	Ptk2b	909.12192	937.709	247.959	465.35	640	-1.37087	2.1E-07	1.5E-06
ENSRNOG00000005686	Suclg2	1332.2093	1470.43	485.88	598.18	971.7	-1.369676	1.4E-12	2.3E-11
ENSRNOG00000018020	Apbb1	1840.9461	1889.96	549.124	894.06	1294	-1.369398	8E-10	8.5E-09
ENSRNOG00000001443	Rhbdd2	571.68393	574.256	195.757	248.25	397.5	-1.366666	6.2E-09	5.7E-08
ENSRNOG00000010950	Cep41	570.65201	547.256	179.695	253.75	387.8	-1.365279	3.5E-08	2.9E-07
ENSRNOG00000001070	Cdk2ap1	3102.9848	2931.51	1036.01	1308.1	2095	-1.363775	1.4E-14	2.8E-13
ENSRNOG00000009157	Fut4	78.425955	86.1903	29.1126	34.81	57.13	-1.36324	0.00503	0.01411
ENSRNOG00000014628	Pomk	960.71794	900.325	277.072	446.12	646.1	-1.362245	1.5E-08	1.3E-07
ENSRNOG00000050106	Rcc1	1231.0811	1150.59	375.452	550.55	826.9	-1.361802	4.8E-10	5.3E-09
ENSRNOG00000060773	Sertad4	4626.0994	4575.35	1450.61	2128.9	3195	-1.36173	2E-12	3.1E-11
ENSRNOG00000048812	Gpx1	3914.0743	3678.14	1429.53	1525.2	2637	-1.361374	9.1E-18	2.7E-16
ENSRNOG00000011814	Cbx3	2882.1538	2947.08	943.651	1324.6	2024	-1.361198	1.3E-12	2E-11
ENSRNOG00000003191	ENSRNOG0000000319	1530.338	1622.04	483.872	742.92	1095	-1.360611	4.1E-10	4.5E-09
ENSRNOG00000042501	Tmem250	1176.3893	1139.17	353.367	547.8	804.2	-1.360304	2.1E-09	2.1E-08
ENSRNOG00000014336	Mcm5	7364.8163	6818.38	2090.09	3437.9	4928	-1.359089	1.2E-10	1.4E-09
ENSRNOG00000024429	Peg12	672.81214	671.869	193.75	329.78	467.1	-1.35908	2.1E-07	1.5E-06
ENSRNOG00000048282	Mpnd	758.46153	800.635	229.889	377.41	541.6	-1.358588	5.7E-08	4.5E-07
ENSRNOG00000015213	Fxn	700.67399	768.443	227.882	344.43	510.4	-1.358513	2.1E-08	1.8E-07
ENSRNOG00000013657	Stmn3	69.13867	78.9212	19.0738	38.474	51.4	-1.358384	0.01191	0.02979
ENSRNOG00000016623	Tmeff2	2750.068	2758.09	1050.06	1099.3	1914	-1.357583	5E-17	1.3E-15

ENSRNOG00000009745	Ppp3cc	731.6316	793.366	235.913	359.09	530	-1.356303	1.9E-08	1.6E-07
ENSRNOG00000061733	Galr2	74.298273	77.8828	31.1204	28.398	52.92	-1.35513	0.00678	0.01829
ENSRNOG00000047967	Arhgef19	172.33072	206.649	69.268	78.78	131.8	-1.355093	7.4E-05	0.00032
ENSRNOG00000003276	Myo1d	9717.5949	9737.42	3537.69	4073.7	6767	-1.353814	1.4E-18	4.6E-17
ENSRNOG00000017690	Ccsap	257.98011	201.457	88.3418	91.605	159.8	-1.352107	2.3E-05	0.00011
ENSRNOG00000015076	Cyp26b1	228.05442	245.071	86.334	98.933	164.6	-1.351651	1.3E-05	6.4E-05
ENSRNOG00000021145	Gpr137	815.21716	828.673	204.792	438.79	571.9	-1.351186	3.1E-06	1.8E-05
ENSRNOG00000049819	Dnajc19	393.16169	325.031	126.489	154.81	249.9	-1.351104	8.8E-07	5.5E-06
ENSRNOG00000030467	Ube2l6	626.37572	636.562	208.808	285.81	439.4	-1.35098	1.1E-08	9.9E-08
ENSRNOG00000008151	Plscr4	385.93825	433.028	137.532	183.21	284.9	-1.350795	3.7E-07	2.5E-06
ENSRNOG00000020482	Nfatc4	537.63056	547.256	178.691	246.42	377.5	-1.350083	4.3E-08	3.4E-07
ENSRNOG00000017416	Ppic	6655.8869	6519.31	2337.04	2830.6	4586	-1.350066	9.1E-17	2.4E-15
ENSRNOG00000059245	Rad51b	130.02198	131.882	42.1631	60.459	91.13	-1.349064	0.00084	0.00284
ENSRNOG00000004245	Mgst3	769.81266	751.828	275.064	322.45	529.8	-1.347844	2.2E-10	2.5E-09
ENSRNOG00000008421	Klhl5	1016.4416	1033.24	328.27	476.35	713.6	-1.347836	1.1E-09	1.2E-08
ENSRNOG00000011096	Hmgb3	1687.1899	1703.04	591.287	742.92	1181	-1.344778	7.1E-13	1.2E-11
ENSRNOG00000018765	Pold4	532.47095	434.067	181.703	198.78	336.8	-1.344533	5.1E-08	4E-07
ENSRNOG00000061216	Tubb5	64547.656	62678	20648.9	29458	44333	-1.344271	1E-13	1.9E-12
ENSRNOG00000015594	Rftn2	68.10675	37.3837	26.101	15.573	36.79	-1.343791	0.03373	0.07273
ENSRNOG00000065021	Naaladl2	174.39456	144.343	51.1981	74.2	111	-1.343369	0.00038	0.00142
ENSRNOG00000007806	Arf5	3559.0936	3484.99	1289.99	1485.8	2455	-1.343252	2.3E-16	5.7E-15
ENSRNOG00000016254	Sema4c	1025.7289	1088.28	355.375	477.26	736.7	-1.343209	1.9E-10	2.3E-09

ENSRNOG00000021903	Atad5	925.63265	907.594	242.94	479.09	638.8	-1.342675	8.3E-07	5.2E-06
ENSRNOG00000033110	Svep1	1432.3056	1595.04	486.884	706.27	1055	-1.342374	2.3E-10	2.7E-09
ENSRNOG00000001088	Rfc3	1441.5929	1366.58	465.802	641.23	978.8	-1.342056	5.4E-11	6.9E-10
ENSRNOG00000007418	Ipo9	9655.6797	9476.78	3334.9	4213.8	6670	-1.341569	5.3E-16	1.3E-14
ENSRNOG00000054391	Snrpn	2325.9487	2410.21	822.181	1046.1	1651	-1.341479	1.5E-13	2.8E-12
ENSRNOG00000020189	Tinf2	464.3642	465.22	159.617	207.03	324.1	-1.340798	9.2E-08	7E-07
ENSRNOG00000002592	ENSRNOG0000000259	163.04343	158.881	46.1786	80.612	112.2	-1.340755	0.00056	0.00199
ENSRNOG00000043192	Hacd1	2013.2768	1899.3	701.715	842.77	1364	-1.340532	9.1E-14	1.7E-12
ENSRNOG00000046905	Sgce	701.70591	714.445	277.072	282.14	493.8	-1.340404	2.1E-10	2.4E-09
ENSRNOG00000038001	Slc25a1	1023.6651	1056.09	410.588	411.31	725.4	-1.339377	2.5E-12	3.8E-11
ENSRNOG00000003068	Mrnip	118.67085	104.882	22.0854	65.956	77.9	-1.338848	0.00799	0.02105
ENSRNOG00000009245	Exosc2	982.38827	1106.97	302.169	523.06	728.6	-1.338836	7.9E-08	6E-07
ENSRNOG00000011981	Slc39a13	3175.2192	3312.61	1120.33	1444.6	2263	-1.338404	6.1E-14	1.2E-12
ENSRNOG00000012457	Cyc1	3273.2517	3148.54	1258.87	1281.6	2241	-1.337874	3.7E-17	1E-15
ENSRNOG00000009867	Tgfb3	23507.148	23230.9	7250.05	11239	16307	-1.337845	8.3E-12	1.2E-10
ENSRNOG00000017857	Cul7	3053.4526	3007.31	1219.72	1180.8	2115	-1.336231	5.8E-17	1.6E-15
ENSRNOG00000052407	ENSRNOG0000005240	2021.5322	1929.42	622.408	942.61	1379	-1.335252	2.2E-10	2.5E-09
ENSRNOG00000056358	Ccdc27	77.394034	122.536	24.0932	54.963	69.75	-1.333705	0.00874	0.02277
ENSRNOG00000001260	Nudt1	144.46886	136.035	46.1786	65.04	97.93	-1.332189	0.00066	0.00231
ENSRNOG00000066106	Ubald2	1324.9859	1287.66	523.023	514.82	912.6	-1.331986	2.5E-13	4.4E-12
ENSRNOG00000018944	Pank1	797.67451	830.75	297.15	349.93	568.9	-1.330724	2E-10	2.3E-09
ENSRNOG00000007230	Kank3	88.745159	140.189	33.1282	57.711	79.94	-1.329972	0.00393	0.01136

ENSRNOG00000007733	Arhgef9	488.09838	484.95	145.563	240.92	339.9	-1.329968	1.3E-06	8.2E-06
ENSRNOG00000064210	ENSRNOG0000006421	58.819466	43.6144	14.0544	26.565	35.76	-1.329806	0.03432	0.07375
ENSRNOG00000053468	Tuba1b	61220.745	57995.7	21133.8	26296	41662	-1.329691	9E-17	2.4E-15
ENSRNOG00000016255	Podxl2	385.93825	427.836	136.528	186.87	284.3	-1.329595	6.8E-07	4.4E-06
ENSRNOG00000040205	Zcchc24	2300.1507	2366.6	607.35	1249.5	1631	-1.328839	2.7E-07	1.9E-06
ENSRNOG00000024533	Eogt	2731.4934	2864.01	846.274	1380.5	1956	-1.328722	8.9E-10	9.4E-09
ENSRNOG00000001793	Heg1	605.73731	562.833	219.851	245.5	408.5	-1.327781	4.4E-09	4.1E-08
ENSRNOG00000032297	Msmo1	1976.1277	2062.34	990.833	619.25	1412	-1.327449	1.3E-09	1.3E-08
ENSRNOG00000004402	Lpgat1	2902.7922	2941.89	1170.53	1158.8	2044	-1.327231	1.1E-16	2.7E-15
ENSRNOG00000004668	Slc25a35	50.564102	86.1903	26.101	28.398	47.81	-1.32653	0.01551	0.03732
ENSRNOG00000029212	Vcan	22545.398	23179	7605.42	10627	15989	-1.326374	1E-13	1.9E-12
ENSRNOG00000019674	Mfsd13a	187.80952	168.227	49.1903	92.521	124.4	-1.325446	0.00056	0.00199
ENSRNOG00000025209	Plxnd1	947.30298	857.749	481.864	240.92	632	-1.321969	1.8E-06	1E-05
ENSRNOG00000001578	Hoxd4	104.22397	85.1518	40.1553	35.726	66.31	-1.3204	0.00379	0.01101
ENSRNOG00000058645	Tnc	3112.2721	2987.58	1220.72	1222.9	2136	-1.319737	1.4E-16	3.5E-15
ENSRNOG00000054274	Cacnb3	3769.6054	3736.3	1228.75	1779	2628	-1.31893	8.9E-12	1.3E-10
ENSRNOG00000018788	Btbd2	3603.4662	3589.88	1123.35	1760.6	2519	-1.318127	1.8E-10	2.1E-09
ENSRNOG00000010718	Gpr153	218.76714	190.034	85.3301	78.78	143.2	-1.317328	5.7E-05	0.00026
ENSRNOG00000061373	Loxl3	5348.4437	4855.73	1860.2	2237	3575	-1.316245	2.7E-15	6E-14
ENSRNOG00000058007	Nde1	1766.6478	1849.46	656.54	795.13	1267	-1.316207	4.9E-13	8.3E-12
ENSRNOG00000015975	ENSRNOG0000001597	428.24699	440.297	105.408	242.75	304.2	-1.316197	6.9E-05	0.0003
ENSRNOG00000031814	Sort1	5962.4364	5514.1	1969.62	2641.9	4022	-1.315122	2.1E-13	3.7E-12

ENSRNOG00000020518	Fam189b	751.23809	821.404	234.909	396.65	551	-1.314591	2.4E-07	1.7E-06
ENSRNOG00000013202	Akap7	278.61852	256.494	82.3185	132.83	187.6	-1.311885	4.2E-05	0.00019
ENSRNOG00000020882	Shkbp1	1857.4568	1767.42	731.831	729.18	1271	-1.310985	2.2E-14	4.5E-13
ENSRNOG00000003357	Col3a1	116253.06	117647	42238.4	52066	82051	-1.310475	7.9E-17	2.1E-15
ENSRNOG00000065643	Sox4	2223.7886	2327.14	696.695	1138.6	1597	-1.309402	2.7E-09	2.6E-08
ENSRNOG00000030568	Rgs12	1005.0905	989.631	323.251	480.93	699.7	-1.309376	6.5E-09	5.9E-08
ENSRNOG00000021150	Plcb3	4498.1413	4611.7	1607.22	2068.4	3196	-1.309127	4.7E-14	9E-13
ENSRNOG00000008857	Adamts10	2826.4301	2857.78	911.526	1384.2	1995	-1.30748	1.6E-10	1.9E-09
ENSRNOG00000008612	Agbl5	264.17164	241.956	61.2369	142.9	177.6	-1.306393	0.00046	0.00169
ENSRNOG00000010650	Plekhh1	221.8629	275.186	87.3379	113.59	174.5	-1.305032	3.5E-05	0.00016
ENSRNOG00000016879	Ldlrad4	464.3642	473.527	181.703	197.87	329.4	-1.304562	5.1E-08	4E-07
ENSRNOG00000065264	Sox12	1660.36	1781.96	486.884	908.72	1209	-1.301568	1.5E-07	1.1E-06
ENSRNOG00000062943	Lurap1	179.55416	171.342	53.2058	88.857	123.2	-1.301345	0.00046	0.00168
ENSRNOG00000011720	L3mbtl3	578.90738	606.447	221.858	259.24	416.6	-1.300107	9.4E-09	8.4E-08
ENSRNOG00000014310	Tmtc4	1447.7844	1356.2	433.678	705.36	985.8	-1.29864	1.5E-08	1.3E-07
ENSRNOG00000046744	Ankrd29	198.12873	208.726	78.3029	87.025	143	-1.298426	6.6E-05	0.00029
ENSRNOG00000020440	Fads2	1918.3401	1763.27	648.509	848.26	1295	-1.297862	1.3E-11	1.8E-10
ENSRNOG00000029813	AABR07035218.1	9487.4767	10009.5	3188.33	4746.1	6858	-1.296882	1.4E-11	1.9E-10
ENSRNOG00000033192	Osmr	1225.9215	1233.66	402.557	598.18	865.1	-1.296296	2.9E-09	2.8E-08
ENSRNOG00000021771	Trim29	73.266352	36.3453	17.066	27.481	38.54	-1.295849	0.03877	0.08177
ENSRNOG00000031431	Cdca8	2064.8728	2043.64	824.188	849.18	1445	-1.295784	1.1E-14	2.3E-13
ENSRNOG00000007221	Dut	1698.5411	1628.27	535.07	819.86	1170	-1.295055	1.9E-09	1.9E-08

ENSRNOG00000069756	Ccdc92b	145.50078	168.227	22.0854	105.35	110.3	-1.294997	0.01316	0.03246
ENSRNOG00000069314	Lrrc3c	83.585557	89.3056	43.167	27.481	60.88	-1.294443	0.00746	0.01986
ENSRNOG00000027365	Hoxa4	98.032443	93.4593	20.0777	57.711	67.32	-1.293986	0.0133	0.03277
ENSRNOG00000033397	Adamts20	429.27891	510.911	207.804	175.88	331	-1.293881	2E-07	1.4E-06
ENSRNOG00000006604	Thy1	7252.337	7009.45	2968.48	2848.9	5020	-1.293751	4.7E-18	1.4E-16
ENSRNOG00000016708	Necab3	961.74986	955.362	303.173	478.18	674.6	-1.293564	3.9E-08	3.1E-07
ENSRNOG00000029449	Ror1	328.1507	334.377	124.482	145.65	233.2	-1.293325	2E-06	1.2E-05
ENSRNOG00000063477	ENSRNOG0000006347	522.15175	531.68	177.687	251.91	370.9	-1.292951	2.4E-07	1.7E-06
ENSRNOG00000012655	Adamts6	1323.9539	1538.96	524.027	643.98	1008	-1.292828	3.7E-11	4.9E-10
ENSRNOG00000003486	Mnda	310.60806	322.954	155.602	103.51	223.2	-1.292116	1.3E-05	6.4E-05
ENSRNOG00000049605	Zfp296	89.77708	102.805	41.1592	37.558	67.82	-1.291399	0.00397	0.01147
ENSRNOG00000051056	AABR07063279.1	409.67242	365.53	107.416	208.86	272.9	-1.290796	3.9E-05	0.00018
ENSRNOG00000058478	Mapk8ip1	1031.9205	1101.78	306.184	565.2	751.3	-1.290636	5.2E-07	3.4E-06
ENSRNOG00000013318	Ccdc150	150.66039	185.88	51.1981	86.109	118.5	-1.290182	0.00074	0.00256
ENSRNOG00000005708	Mmp16	1899.7656	1873.34	678.625	863.83	1329	-1.289939	4E-12	6E-11
ENSRNOG00000005258	Myef2	4061.6389	4096.63	1498.8	1837.6	2874	-1.289694	1.8E-14	3.6E-13
ENSRNOG00000018577	Neil1	305.44845	311.531	106.412	145.65	217.3	-1.289584	8.2E-06	4.3E-05
ENSRNOG00000056228	Atp10a	1148.5275	1023.9	442.713	446.12	765.3	-1.289315	2.1E-11	2.8E-10
ENSRNOG00000063070	Pcdhga7	3617.9131	3594.03	1380.34	1570.1	2541	-1.289238	1.7E-15	3.8E-14
ENSRNOG00000006403	Nectin1	428.24699	438.22	180.699	174.05	305.3	-1.288566	1.3E-07	9.9E-07
ENSRNOG00000052873	Npnt	13237.476	12950.3	4537.55	6185.2	9228	-1.288104	2.3E-13	4E-12
ENSRNOG00000014753	Tcf7l1	988.5798	1135.01	373.445	495.58	748.2	-1.288101	1.4E-09	1.4E-08

ENSRNOG00000018971	Mob3a	8451.4285	8422.76	3300.77	3614.7	5947	-1.286837	1.1E-17	3.2E-16
ENSRNOG00000000288	Scarf2	1865.7122	1824.53	651.52	862	1301	-1.28516	1.9E-11	2.6E-10
ENSRNOG00000020726	Sipa1	914.28152	904.479	341.32	404.89	641.2	-1.284593	2.7E-10	3.1E-09
ENSRNOG00000011167	Wdhd1	1380.7096	1423.7	416.612	734.67	988.9	-1.283387	1.1E-07	8.4E-07
ENSRNOG00000018005	Duoxa1	340.53375	321.916	100.388	171.3	233.5	-1.283285	2.9E-05	0.00014
ENSRNOG00000024139	Miip	567.55625	590.871	226.878	249.17	408.6	-1.282504	1.1E-08	9.9E-08
ENSRNOG00000020653	S1pr2	784.25955	809.981	331.282	324.28	562.5	-1.282157	3E-10	3.4E-09
ENSRNOG00000017253	Ccdc107	1083.5165	1139.17	363.406	550.55	784.2	-1.280966	1.2E-08	1.1E-07
ENSRNOG00000051399	Col10a1	153.75615	145.381	56.2175	66.872	105.6	-1.279854	0.00055	0.00197
ENSRNOG00000015225	Gramd2b	877.13239	868.133	334.293	384.74	616.1	-1.278715	3.1E-10	3.5E-09
ENSRNOG00000016257	Cotl1	1036.0481	1049.86	528.043	333.44	736.8	-1.277023	4.2E-08	3.4E-07
ENSRNOG00000002253	Wdr5b	155.81999	154.727	61.2369	66.872	109.7	-1.276799	0.00042	0.00154
ENSRNOG00000014597	Irs1	888.48351	871.249	277.072	449.78	621.6	-1.274199	1.5E-07	1.1E-06
ENSRNOG00000000001	Arsj	519.05599	506.757	200.777	223.52	362.5	-1.273055	4.7E-08	3.7E-07
ENSRNOG00000019299	Edem2	1375.55	1419.54	531.054	625.66	988	-1.272336	8.5E-12	1.2E-10
ENSRNOG00000015346	Obsl1	1347.6881	1276.24	425.647	660.47	927.5	-1.271521	1.3E-08	1.1E-07
ENSRNOG00000036960	Abcc9	1428.1779	1579.46	442.713	802.46	1063	-1.271287	2.4E-07	1.7E-06
ENSRNOG00000014041	Cep78	695.51439	725.868	195.757	392.99	502.5	-1.269773	8.1E-06	4.2E-05
ENSRNOG00000042753	Fgf13	76.362114	62.3062	16.0621	41.222	48.99	-1.26971	0.02704	0.06043
ENSRNOG00000008365	ENSRNOG0000000836	116.60701	163.035	46.1786	69.62	98.86	-1.269259	0.0018	0.00567
ENSRNOG00000037160	Bloc1s6	590.2585	634.485	200.777	306.88	433.1	-1.268898	4.5E-07	3E-06
ENSRNOG00000021063	Grin2d	248.69283	292.839	107.416	117.25	191.6	-1.268641	1.6E-05	7.9E-05

ENSRNOG00000033496	Igdcc4	91.84092	92.4209	36.1398	40.306	65.18	-1.268392	0.00525	0.01466
ENSRNOG00000061102	ENSRNOG0000006110	271.39508	299.07	108.419	128.25	201.8	-1.2682	1.1E-05	5.6E-05
ENSRNOG00000003853	Scoc	4740.6426	4657.39	1586.14	2314.9	3325	-1.268179	4.4E-11	5.6E-10
ENSRNOG00000055157	Pard6g	832.75981	676.023	286.107	340.77	533.9	-1.266444	1.3E-08	1.1E-07
ENSRNOG00000007483	Ccnf	2854.292	2823.51	1013.92	1346.6	2010	-1.265771	5.5E-12	8.1E-11
ENSRNOG00000020687	Dpf1	482.93877	478.72	210.816	189.62	340.5	-1.264538	1E-07	7.5E-07
ENSRNOG00000027907	AABR07039303.1	62.947148	45.6912	26.101	19.237	38.49	-1.263188	0.03266	0.07072
ENSRNOG00000007151	Cdk14	3601.4024	3834.95	1403.43	1697.4	2634	-1.26164	8E-14	1.5E-12
ENSRNOG00000004208	Crim1	17555.031	17223.5	5735.19	8769.3	12321	-1.261594	7.9E-11	9.9E-10
ENSRNOG00000014712	Zfp39	77.394034	75.8059	28.1087	35.726	54.26	-1.261146	0.01116	0.02817
ENSRNOG00000019692	Metrn	462.30036	419.529	160.621	207.03	312.4	-1.260857	7.5E-07	4.8E-06
ENSRNOG00000068121	ENSRNOG0000006812	49.532182	56.0756	22.0854	21.985	37.42	-1.260827	0.03154	0.06873
ENSRNOG00000011821	S100a4	8201.7038	7542.17	3143.16	3426.9	5578	-1.260732	1.8E-16	4.6E-15
ENSRNOG00000001478	Gtf2ird1	933.88801	820.365	334.293	397.57	621.5	-1.260549	1.7E-09	1.7E-08
ENSRNOG00000062478	ENSRNOG0000006247	50.564102	42.5759	14.0544	24.733	32.98	-1.259601	0.04933	0.09989
ENSRNOG00000010457	Vash1	910.15384	1011.44	308.192	493.75	680.9	-1.259425	1.4E-07	1E-06
ENSRNOG00000046548	ENSRNOG0000004654	1802.765	1737.31	624.416	853.76	1255	-1.259265	1.6E-10	1.9E-09
ENSRNOG00000005905	Pde4b	437.53427	392.529	175.68	171.3	294.3	-1.258534	4.5E-07	3E-06
ENSRNOG00000018993	Ilk	16200.119	15457.1	6102.61	7130.5	11223	-1.258317	3.1E-16	7.6E-15
ENSRNOG00000062890	Zfp618	917.37728	991.707	353.367	444.28	676.7	-1.258195	1.4E-09	1.4E-08
ENSRNOG00000006519	Tmem107	187.80952	166.15	55.2136	92.521	125.4	-1.257506	0.0007	0.00243
ENSRNOG00000002907	Plekha6	1041.2077	1031.17	374.449	491.92	734.7	-1.257306	1.4E-09	1.4E-08

ENSRNOG00000021041	Fam171a2	584.06698	597.101	201.781	292.22	418.8	-1.256067	3.2E-07	2.2E-06
ENSRNOG00000047236	AABR07008066.2	74.298273	63.3447	18.0699	39.39	48.78	-1.255215	0.02456	0.05571
ENSRNOG00000007090	Cacna1c	1236.2407	1442.39	443.717	677.88	950.1	-1.254979	2.3E-08	1.9E-07
ENSRNOG00000009184	Foxp1	4224.6823	4551.47	1514.86	2162.8	3113	-1.254456	4.7E-11	6E-10
ENSRNOG00000000795	RT1-N3	379.74673	441.336	142.551	201.53	291.3	-1.253034	3.7E-06	2.1E-05
ENSRNOG00000006996	Mms22l	1508.6677	1389.43	398.542	817.12	1028	-1.252338	2.7E-06	1.6E-05
ENSRNOG00000017022	Cerk	2824.3663	2903.47	1049.06	1355.8	2033	-1.251639	3.5E-12	5.3E-11
ENSRNOG00000017577	Bphl	110.41549	80.9981	26.101	54.047	67.89	-1.251586	0.01124	0.02834
ENSRNOG00000000506	Tead3	2891.4411	2933.58	1165.51	1281.6	2068	-1.251023	1.5E-14	3E-13
ENSRNOG00000063806	ENSRNOG0000006380	172.33072	142.266	53.2058	78.78	111.6	-1.250614	0.00096	0.0032
ENSRNOG00000017078	Selenon	11145.773	10908.8	4045.65	5222.4	7831	-1.250612	9.6E-14	1.8E-12
ENSRNOG00000004440	Fignl1	241.46939	252.34	64.2485	142.9	175.2	-1.249817	0.00066	0.00232
ENSRNOG00000012862	Spsb4	1305.3794	1345.81	511.981	602.76	941.5	-1.249399	2.9E-11	3.8E-10
ENSRNOG00000046227	Tprg1l	1843.0099	1695.77	709.746	778.64	1257	-1.249249	1.2E-12	2E-11
ENSRNOG00000056836	Cav1	18949.155	19106.2	6950.89	9059.7	13516	-1.248993	8.9E-14	1.7E-12
ENSRNOG00000035513	Mir214	61.915227	83.075	26.101	34.81	51.48	-1.248949	0.01598	0.03831
ENSRNOG00000016810	Stmn1	7933.4045	7009.45	2742.61	3545.1	5308	-1.248674	8E-13	1.3E-11
ENSRNOG00000060594	Atn1	9713.4672	10364.6	3822.79	4630.6	7133	-1.247901	8.6E-15	1.8E-13
ENSRNOG00000031950	Izumo4	350.85295	372.799	140.544	163.97	257	-1.247849	2.1E-06	1.2E-05
ENSRNOG00000023148	Col11a1	1722.2752	1724.84	626.423	824.44	1224	-1.247816	8.2E-11	1E-09
ENSRNOG00000014513	Rexo5	759.49345	776.751	279.08	367.34	545.7	-1.247767	1.6E-08	1.4E-07
ENSRNOG00000028274	Myrf	2667.5144	2915.93	1050.06	1300.8	1984	-1.24759	1.8E-12	2.8E-11

ENSRNOG00000021663	Vxn	94.936682	76.8443	18.0699	54.047	60.97	-1.246456	0.02297	0.05266
ENSRNOG00000017488	Snx24	1003.0267	966.785	357.383	472.68	700	-1.245801	3.3E-09	3.1E-08
ENSRNOG00000016012	Spats2l	2656.1633	2660.48	936.623	1305.4	1890	-1.245212	8.2E-11	1E-09
ENSRNOG00000009795	Nfib	430.31083	400.837	107.416	242.75	295.3	-1.244476	0.00017	0.00067
ENSRNOG00000024043	Orc6	803.86603	850.48	237.92	459.86	588	-1.243836	5E-06	2.7E-05
ENSRNOG00000013572	Lxn	2331.1083	2213.95	841.254	1077.3	1616	-1.243806	1.1E-11	1.6E-10
ENSRNOG00000032703	Rasgrp3	1887.3825	2060.26	634.454	1033.3	1404	-1.242324	2.4E-08	2.1E-07
ENSRNOG00000027914	Plscr3	1265.1345	1304.28	438.697	646.73	913.7	-1.242193	8.6E-09	7.7E-08
ENSRNOG00000008781	Erg28	588.19466	599.178	182.707	318.79	422.2	-1.241582	4.5E-06	2.4E-05
ENSRNOG00000012406	Pcbp4	2644.8121	2820.4	1063.11	1252.2	1945	-1.238726	4E-13	6.9E-12
ENSRNOG00000015380	Jup	4438.2899	4282.51	1790.93	1904.5	3104	-1.238644	1.4E-15	3.2E-14
ENSRNOG00000070725	ENSRNOG0000007072	230.11826	179.65	103.4	70.536	145.9	-1.238581	0.00035	0.00129
ENSRNOG00000019041	Psme1	870.94086	880.595	371.437	371	623.5	-1.238283	3.8E-10	4.2E-09
ENSRNOG00000029662	Wdfy4	248.69283	219.11	99.3845	98.933	166.5	-1.238147	5.4E-05	0.00024
ENSRNOG00000002285	ENSRNOG0000000228	245.59707	241.956	88.3418	118.17	173.5	-1.237522	6.7E-05	0.00029
ENSRNOG00000018526	Dlg4	1515.8911	1525.46	597.311	692.53	1083	-1.237072	1E-11	1.4E-10
ENSRNOG00000023065	ENSRNOG0000002306	1289.9006	1138.13	444.72	585.36	864.5	-1.23624	1.8E-09	1.8E-08
ENSRNOG00000066229	ENSRNOG0000006622	195.03297	178.611	74.2874	84.277	133.1	-1.235746	0.00023	0.00091
ENSRNOG00000011800	F3	2752.1319	2792.36	842.258	1511.5	1975	-1.235515	1.1E-07	8.1E-07
ENSRNOG00000014190	Hint3	167.17111	148.497	53.2058	80.612	112.4	-1.235474	0.00104	0.00345
ENSRNOG00000042620	Marveld1	6389.6515	6293.97	2097.11	3290.4	4518	-1.234998	1E-09	1.1E-08
ENSRNOG00000009722	Chd3	6285.4275	6841.22	2601.06	2975.3	4676	-1.234967	6.1E-15	1.3E-13

ENSRNOG00000038789	Cenpl	915.31344	926.286	370.433	412.22	656.1	-1.234066	4.7E-10	5.2E-09
ENSRNOG00000012953	Arsa	2129.8838	2073.76	797.084	991.17	1498	-1.232613	8.8E-12	1.3E-10
ENSRNOG00000045821	Slc41a3	1308.4751	1349.97	498.93	632.07	947.4	-1.232269	2.5E-10	2.9E-09
ENSRNOG00000010891	Lrrcc1	1266.1664	1344.78	471.825	640.32	930.8	-1.230382	1.7E-09	1.7E-08
ENSRNOG00000013229	Mthfs	296.16117	400.837	113.439	183.21	248.4	-1.230231	6.4E-05	0.00028
ENSRNOG00000015625	MGC116202	1457.0717	1493.27	731.831	526.73	1052	-1.229917	1.5E-09	1.5E-08
ENSRNOG00000047453	Casd1	486.03453	455.874	155.602	245.5	335.8	-1.229682	5.4E-06	2.9E-05
ENSRNOG00000018207	Dynlt1	1396.1884	1351.01	539.085	632.07	979.6	-1.229523	4E-11	5.1E-10
ENSRNOG00000001098	Pds5b	3590.0513	3578.45	1310.07	1746.9	2556	-1.229196	1.1E-11	1.5E-10
ENSRNOG00000053309	Tubd1	128.99006	200.418	68.2641	72.368	117.5	-1.227488	0.00109	0.0036
ENSRNOG00000039859	Dnmt1	4384.63	4235.79	1378.33	2305.7	3076	-1.226091	1.4E-08	1.2E-07
ENSRNOG00000016731	Tpm2	32816.102	32690	12450.2	15552	23377	-1.226062	1.9E-14	3.9E-13
ENSRNOG00000050767	Nrn1	1755.2967	1639.69	552.136	898.64	1211	-1.225736	5.1E-08	4E-07
ENSRNOG00000064015	ENSRNOG0000006401	114.54317	127.728	42.1631	61.375	86.45	-1.223832	0.0031	0.00919
ENSRNOG00000006663	Usp2	668.68445	679.138	245.951	330.69	481.1	-1.223639	9.6E-08	7.2E-07
ENSRNOG00000006197	Calu	72965.032	71223.3	30631.5	31140	51490	-1.222935	4E-18	1.3E-16
ENSRNOG00000018648	Mppe1	672.81214	704.06	201.781	387.49	491.5	-1.222617	1.1E-05	5.8E-05
ENSRNOG00000015134	Map3k12	560.33281	720.675	209.812	338.94	457.4	-1.221474	4.3E-06	2.4E-05
ENSRNOG00000013287	Sall2	531.43903	560.756	211.819	256.49	390.1	-1.220726	1.6E-07	1.2E-06
ENSRNOG00000013656	Lpar1	409.67242	317.762	106.412	205.2	259.8	-1.220639	0.00016	0.00064
ENSRNOG00000004479	Aurka	1777.9989	1584.66	806.119	637.57	1202	-1.220422	1.7E-10	2E-09
ENSRNOG00000010643	Kank2	5286.5285	5297.07	1795.95	2745.4	3781	-1.220333	8.7E-10	9.2E-09

ENSRNOG00000016488	Pltp	198.12873	178.611	79.3068	82.444	134.6	-1.219548	0.00025	0.00096
ENSRNOG00000059903	Thbs3	778.06802	844.249	387.499	309.62	579.9	-1.219451	1.2E-08	1.1E-07
ENSRNOG00000023079	Nras	2301.1826	2248.22	955.697	998.49	1626	-1.219	1.6E-13	2.9E-12
ENSRNOG00000019365	Ablim3	69.13867	58.1525	16.0621	38.474	45.46	-1.217386	0.03673	0.07808
ENSRNOG00000016143	P3h3	3619.977	3724.87	1510.84	1648	2626	-1.217205	1.6E-14	3.3E-13
ENSRNOG00000017622	Npm3	1030.8885	1056.09	438.697	458.94	746.2	-1.21703	1.5E-10	1.8E-09
ENSRNOG00000005074	Arl6ip6	1176.3893	1209.78	396.534	630.24	853.2	-1.215481	1.1E-07	8.4E-07
ENSRNOG00000020822	Atp8b2	1400.3161	1432	512.985	706.27	1013	-1.215165	2.2E-09	2.2E-08
ENSRNOG00000052062	Tut4	1272.3579	1329.2	468.814	651.31	930.4	-1.214838	4.4E-09	4.2E-08
ENSRNOG00000024435	Galnt17	2363.0978	2410.21	947.666	1109.3	1708	-1.214108	1.4E-12	2.2E-11
ENSRNOG00000019630	Hacl1	356.01256	367.607	151.586	160.31	258.9	-1.213827	2.7E-06	1.5E-05
ENSRNOG00000013057	Prc1	11779.372	11689.7	5131.85	4998	8400	-1.212168	6.7E-17	1.8E-15
ENSRNOG00000004471	Polr3h	715.12088	655.254	232.901	358.18	490.4	-1.211655	8.8E-07	5.6E-06
ENSRNOG00000004060	Calm1	23759.968	23034.6	10225.6	9994.1	16754	-1.21059	2.5E-17	7E-16
ENSRNOG00000048397	Sephs2	1054.6227	1010.4	343.328	548.71	739.3	-1.209768	2.5E-07	1.8E-06
ENSRNOG00000036921	Tmsb10	141.3731	114.228	60.233	50.383	91.55	-1.209656	0.00238	0.00727
ENSRNOG00000027008	Igtp	195.03297	203.534	74.2874	98.017	142.7	-1.208048	0.00028	0.00108
ENSRNOG00000012874	Rgma	418.9597	422.644	137.532	226.26	301.3	-1.207873	1.9E-05	9.2E-05
ENSRNOG00000004109	Zfpm2	1126.8571	1023.9	414.604	516.65	770.5	-1.206868	2.4E-09	2.4E-08
ENSRNOG00000022162	Pbx3	2054.5536	2175.53	832.219	1000.3	1516	-1.206419	1E-11	1.5E-10
ENSRNOG00000003435	ENSRNOG0000000343	213.60753	235.725	88.3418	106.26	161	-1.206025	0.00012	0.00051
ENSRNOG00000006661	Rad51c	551.04552	497.411	161.625	292.22	375.6	-1.205991	2.1E-05	0.0001

ENSRNOG00000025587	Plagl1	143.43694	155.766	83.3223	46.719	107.3	-1.20559	0.00241	0.00734
ENSRNOG00000024733	Mnd1	72.234432	66.46	27.1049	32.978	49.69	-1.205401	0.01939	0.04541
ENSRNOG00000043364	Zfp362	280.68236	274.147	109.423	131	198.8	-1.205382	3E-05	0.00014
ENSRNOG00000005289	Prkd3	3224.7514	3162.04	976.779	1792.7	2289	-1.204968	2.9E-07	2E-06
ENSRNOG00000058908	Hmgb1	273.45892	361.376	95.3689	179.55	227.4	-1.20478	0.00028	0.00106
ENSRNOG00000020567	Fibp	1622.179	1641.77	555.148	860.17	1170	-1.204652	2.8E-08	2.4E-07
ENSRNOG00000003720	Prrx1	3664.3495	3948.14	1473.7	1829.4	2729	-1.204278	2.6E-12	3.9E-11
ENSRNOG00000002207	Guf1	827.6002	887.864	294.138	449.78	614.8	-1.204071	3.1E-07	2.1E-06
ENSRNOG00000028751	Nxf3	499.4495	539.987	191.742	259.24	372.6	-1.203253	1E-06	6.3E-06
ENSRNOG00000066694	Mrtfb	1239.3365	1169.28	419.623	626.58	863.7	-1.20204	4.5E-08	3.6E-07
ENSRNOG00000008425	Nav1	3016.3035	2940.85	1140.41	1448.3	2136	-1.202023	1.1E-11	1.5E-10
ENSRNOG00000016598	Hoxc5	98.032443	87.2287	28.1087	52.215	66.4	-1.201662	0.01228	0.03058
ENSRNOG00000023679	Prdm5	401.41706	372.799	150.583	185.96	277.7	-1.200807	3.8E-06	2.1E-05
ENSRNOG00000045636	Fasn	5363.9225	5298.11	2265.77	2373.5	3825	-1.200462	2.4E-15	5.4E-14
ENSRNOG00000018378	Cacnb2	852.3663	848.403	319.235	420.47	610.1	-1.200154	2.5E-08	2.1E-07
ENSRNOG00000024420	Eme1	278.61852	266.878	104.404	132.83	195.7	-1.199824	4.5E-05	0.0002
ENSRNOG00000056944	Arhgap24	586.13082	485.989	226.878	240	384.8	-1.199089	3.2E-07	2.2E-06
ENSRNOG00000013727	ENSRNOG0000001372	4057.5112	4234.75	1510.84	2101.4	2976	-1.198519	1.2E-10	1.5E-09
ENSRNOG00000007722	Tlcd3a	1068.0377	1079.97	354.371	581.69	771	-1.197133	4.4E-07	2.9E-06
ENSRNOG00000022822	Fam169b	126.92622	152.65	39.1515	82.444	100.3	-1.197095	0.00512	0.01432
ENSRNOG00000030830	Mex3d	2381.6724	2321.95	770.983	1279.7	1689	-1.19701	6.6E-08	5.1E-07
ENSRNOG00000015117	Lrrc39	71.202511	70.6137	25.0971	36.642	50.89	-1.196966	0.02055	0.0478

ENSRNOG00000001833	Mcm4	6829.2496	6526.58	2257.73	3568.9	4796	-1.196484	4.2E-09	4E-08
ENSRNOG00000011216	Tbl1xr1	1756.3286	1846.34	696.695	874.83	1294	-1.196358	1.1E-10	1.4E-09
ENSRNOG00000001143	Cit	2897.6326	2899.32	1001.88	1529.8	2082	-1.194687	5.4E-09	5E-08
ENSRNOG00000017212	Spsb1	1735.6902	1621	781.021	686.12	1206	-1.194394	2.3E-11	3.1E-10
ENSRNOG00000001199	Pop5	216.7033	278.301	69.268	146.57	177.7	-1.194325	0.0011	0.00363
ENSRNOG00000024549	Pwwp3a	1569.551	1683.31	532.058	888.57	1168	-1.194308	2.1E-07	1.5E-06
ENSRNOG00000049507	Septin10	1447.7844	1533.77	530.051	772.23	1071	-1.194186	1.4E-08	1.2E-07
ENSRNOG00000019491	Stard10	715.12088	685.369	284.099	327.95	503.1	-1.193579	2.4E-08	2.1E-07
ENSRNOG00000006132	Fam71f1	215.67138	276.224	77.299	137.41	176.7	-1.193169	0.00053	0.00191
ENSRNOG00000018406	Wipf1	1377.6138	1607.5	507.965	797.88	1073	-1.191926	1.3E-07	9.4E-07
ENSRNOG00000033531	Cacna2d1	2654.0994	2477.71	1015.93	1231.2	1845	-1.191045	9.5E-12	1.3E-10
ENSRNOG00000064076	ENSRNOG0000006407	66.042909	44.6528	19.0738	29.314	39.77	-1.190904	0.04401	0.09092
ENSRNOG00000065301	ENSRNOG0000006530	80.489795	105.921	31.1204	50.383	66.98	-1.190266	0.01186	0.02968
ENSRNOG00000010840	Adamtsl3	1586.0617	1621	555.148	850.09	1153	-1.189599	3.4E-08	2.8E-07
ENSRNOG00000005788	Cdk5rap2	2174.2564	2291.83	853.301	1104.8	1606	-1.189087	1.2E-10	1.5E-09
ENSRNOG00000057760	Stil	1265.1345	1231.59	537.078	557.87	897.9	-1.189037	7.3E-11	9.1E-10
ENSRNOG00000008691	Cilk1	1338.4008	1346.85	521.016	656.81	965.8	-1.188261	8E-10	8.5E-09
ENSRNOG00000014650	Sema4g	288.93773	290.762	159.617	95.269	208.6	-1.188002	0.00015	0.00062
ENSRNOG00000007329	Frmd6	11820.649	11590	5485.22	4792.8	8422	-1.187681	6.2E-15	1.3E-13
ENSRNOG00000026958	Cyren	280.68236	285.57	83.3223	164.89	203.6	-1.186928	0.00044	0.0016
ENSRNOG00000004544	Ube2e3	3495.1146	3321.96	1374.32	1620.5	2453	-1.186434	1.1E-12	1.7E-11
ENSRNOG00000069321	Ttc30a	54.691784	57.114	15.0583	33.894	40.19	-1.186201	0.04949	0.10016

ENSRNOG00000061346	B3glct	1739.8179	1709.27	684.649	830.86	1241	-1.185921	7.6E-11	9.4E-10
ENSRNOG00000015078	Ifitm3	1958.585	2034.3	838.243	916.97	1437	-1.185558	3.5E-12	5.2E-11
ENSRNOG00000058560	Col2a1	9334.7524	9940.96	3482.47	4991.6	6937	-1.185504	1.8E-10	2.1E-09
ENSRNOG00000063796	Foxf2	1301.2517	1433.04	403.561	798.8	984.2	-1.184267	6.4E-06	3.4E-05
ENSRNOG00000007713	Tmcc3	428.24699	452.759	156.606	230.84	317.1	-1.183354	8.4E-06	4.4E-05
ENSRNOG00000004328	Deptor	5327.8053	5235.8	1960.58	2692.3	3804	-1.182646	7.3E-11	9.2E-10
ENSRNOG00000004216	Lrr1	79.457875	98.6515	38.1476	40.306	64.14	-1.182379	0.01029	0.02618
ENSRNOG00000022777	Six1	1235.2088	1209.78	425.647	651.31	880.5	-1.18185	1.1E-07	8E-07
ENSRNOG00000012364	Prickle2	1522.0827	1476.66	548.12	773.15	1080	-1.181643	8E-09	7.3E-08
ENSRNOG00000011910	Hnrnpr	1872.9356	2029.11	671.598	1048.9	1406	-1.180706	4.4E-08	3.6E-07
ENSRNOG00000000900	Tpst1	1577.8064	1516.12	656.54	709.94	1115	-1.178744	2.6E-11	3.5E-10
ENSRNOG00000067908	Gadl1	179.55416	193.149	68.2641	96.185	134.3	-1.178205	0.00065	0.00226
ENSRNOG00000010077	Smarcd3	2722.2062	2507.83	1039.02	1274.2	1886	-1.17654	2.5E-11	3.3E-10
ENSRNOG00000025895	Cavin2	10524.557	10435.3	3764.56	5511	7559	-1.175975	4E-10	4.4E-09
ENSRNOG00000025602	Cdk4	6060.4688	5709.33	2241.67	2967.1	4245	-1.175854	2.4E-11	3.2E-10
ENSRNOG00000018397	Dnph1	241.46939	239.879	63.2447	149.32	173.5	-1.175793	0.00179	0.00565
ENSRNOG00000011526	Pcsk6	2743.8765	2509.9	1018.94	1306.3	1895	-1.175571	1E-10	1.3E-09
ENSRNOG00000071046	ENSRNOG0000007104	142.40502	116.305	39.1515	75.116	93.24	-1.17521	0.0061	0.0167
ENSRNOG00000002983	Nfix	1580.9021	1673.96	511.981	928.87	1174	-1.174786	1.4E-06	8.3E-06
ENSRNOG00000005506	Arhgef5	8098.5117	7979.35	3384.09	3737.5	5800	-1.174728	7.6E-15	1.6E-13
ENSRNOG00000008747	Plekha5	661.46101	765.328	244.948	386.57	514.6	-1.174399	2.7E-06	1.5E-05
ENSRNOG00000001706	Kalrn	3234.0387	3276.27	1037.01	1846.8	2349	-1.174286	3E-07	2.1E-06

ENSRNOG00000054474	Mastl	1410.6353	1406.04	551.132	697.11	1016	-1.17344	1E-09	1.1E-08
ENSRNOG00000045775	Rnpepl1	1699.573	1803.77	552.136	1000.3	1264	-1.17334	1.2E-06	7.4E-06
ENSRNOG00000026040	Prss53	82.553636	87.2287	32.1243	43.054	61.24	-1.173129	0.013	0.03214
ENSRNOG00000020087	Zfp90	128.99006	151.612	36.1398	87.941	101.2	-1.172902	0.00813	0.02134
ENSRNOG00000027697	Nmnat2	515.96023	586.717	265.025	224.43	398	-1.172528	4.7E-07	3.1E-06
ENSRNOG00000057116	Pex19	4120.4584	3881.68	1582.12	1970.4	2889	-1.171261	7.8E-12	1.1E-10
ENSRNOG00000017765	Net1	589.22658	582.563	213.827	305.96	422.9	-1.171255	1.5E-06	9.1E-06
ENSRNOG00000000040	RGD1304622	1022.6332	1182.78	407.577	571.61	796.2	-1.170439	7.3E-08	5.6E-07
ENSRNOG00000032569	Lingo3	307.5123	314.646	97.3767	178.63	224.5	-1.169919	0.00025	0.00095
ENSRNOG00000006619	Dnajc9	2186.6394	2204.6	778.01	1173.5	1586	-1.169436	1.6E-08	1.4E-07
ENSRNOG00000012486	Prim2	851.33438	813.096	322.247	417.72	601.1	-1.168539	5.3E-08	4.2E-07
ENSRNOG00000002876	Eef2kmt	62.947148	62.3062	17.066	38.474	45.2	-1.168052	0.04254	0.08832
ENSRNOG00000011901	Rrad	4337.1617	4344.82	1674.48	2188.4	3136	-1.168041	3E-11	4E-10
ENSRNOG00000011647	S100a6	20654.92	18836.2	7972.84	9602.9	14267	-1.167885	2.1E-13	3.8E-12
ENSRNOG00000032878	Cxxc5	542.79016	479.758	204.792	250.08	369.4	-1.167686	1.1E-06	6.8E-06
ENSRNOG00000019838	Gmfg	536.59864	606.447	232.901	275.73	412.9	-1.167376	4E-07	2.7E-06
ENSRNOG00000017786	Acta1	399.35322	331.261	182.707	142.9	264.1	-1.167284	1.8E-05	8.7E-05
ENSRNOG00000003114	B4galt4	223.92674	235.725	87.3379	117.25	166.1	-1.165981	0.00022	0.00086
ENSRNOG00000066013	ENSRNOG0000006601	411.73626	275.186	161.625	144.74	248.3	-1.165522	6.2E-05	0.00028
ENSRNOG00000023463	Parp9	694.48247	717.56	298.153	331.61	510.5	-1.164414	3.4E-08	2.8E-07
ENSRNOG00000042029	Efs	1866.7441	1827.65	790.056	858.34	1336	-1.164057	1.2E-11	1.6E-10
ENSRNOG00000046257	Ctdspl	676.93982	684.33	273.056	334.36	492.2	-1.163317	1.1E-07	8.1E-07

ENSRNOG00000031135	Smarcc2	4733.4191	5063.42	1703.59	2673	3543	-1.16218	1.4E-08	1.2E-07
ENSRNOG00000002182	Septin11	12924.804	13046.9	5042.51	6563.5	9394	-1.161966	5.2E-12	7.6E-11
ENSRNOG00000051706	Tep1	1318.7943	1608.54	456.767	851.01	1059	-1.161538	5.3E-06	2.9E-05
ENSRNOG00000039473	Ccdc152	177.49032	170.304	83.3223	72.368	125.9	-1.160527	0.0007	0.00243
ENSRNOG00000012791	Ajuba	6374.1727	6413.39	2005.76	3714.6	4627	-1.16031	5E-07	3.3E-06
ENSRNOG00000005602	Mthfd1	3189.6661	2931.51	1090.22	1648	2215	-1.160121	1.2E-08	1.1E-07
ENSRNOG00000020416	Zfr2	648.04605	707.176	233.905	371.92	490.3	-1.160026	3.8E-06	2.1E-05
ENSRNOG00000007981	Klhl23	1227.9853	1122.55	432.674	619.25	850.6	-1.159029	7.2E-08	5.6E-07
ENSRNOG00000016847	Bace1	705.83359	718.598	296.146	341.69	515.6	-1.158503	4.8E-08	3.8E-07
ENSRNOG00000029330	Ca5b	327.11878	354.107	111.431	193.29	246.5	-1.158241	0.00015	0.0006
ENSRNOG00000016889	Fancc	210.51177	160.958	68.2641	98.017	134.4	-1.157539	0.00113	0.00372
ENSRNOG00000070673	ENSRNOG0000007067	146.5327	165.112	33.1282	106.26	112.8	-1.156347	0.013	0.03214
ENSRNOG00000002928	Guk1	1247.5918	1298.05	472.829	668.72	921.8	-1.156153	3.5E-08	2.9E-07
ENSRNOG00000019675	Pycard	66.042909	71.6522	24.0932	37.558	49.84	-1.156071	0.02779	0.06179
ENSRNOG00000019485	Kat8	3962.5745	4018.75	1509.84	2073.9	2891	-1.154808	3.6E-10	4E-09
ENSRNOG00000016271	Itm2b	7173.911	6616.92	2929.33	3265.7	4996	-1.154429	1.1E-13	2E-12
ENSRNOG00000008986	Diaph3	8698.0575	8602.41	3259.61	4513.4	6268	-1.154102	1.3E-10	1.6E-09
ENSRNOG00000014019	Tbc1d7	861.65358	878.518	335.297	446.12	630.4	-1.154065	8.2E-08	6.2E-07
ENSRNOG00000010194	Ift43	675.9079	699.907	255.99	361.84	498.4	-1.153699	7.7E-07	4.9E-06
ENSRNOG00000021384	Ankrd44	432.37467	499.488	175.68	242.75	337.6	-1.153631	7.5E-06	4E-05
ENSRNOG00000024482	Tnrc18	9093.283	8737.41	3566.8	4447.4	6461	-1.153596	2.2E-12	3.4E-11
ENSRNOG00000004487	Nek2l1	1594.3171	1468.35	568.198	807.96	1110	-1.153385	2.4E-08	2E-07

ENSRNOG00000008829	Sorbs3	1721.2433	1633.46	581.249	927.04	1216	-1.152468	1.9E-07	1.4E-06
ENSRNOG00000013668	Capg	2467.3218	2352.06	954.693	1214.7	1747	-1.151133	1.9E-10	2.3E-09
ENSRNOG00000028390	Hhipl1	1165.0382	1184.86	510.977	547.8	852.2	-1.149953	5.3E-10	5.9E-09
ENSRNOG00000058739	Snn	401.41706	440.297	152.59	226.26	305.1	-1.149883	2E-05	1E-04
ENSRNOG00000064248	Cdkn2d	955.55834	806.866	376.456	417.72	639.1	-1.149662	2.5E-08	2.1E-07
ENSRNOG00000003144	Gprc5c	1735.6902	1712.38	679.629	874.83	1251	-1.148794	1.1E-09	1.2E-08
ENSRNOG00000014928	Apba1	221.8629	184.842	74.2874	109.01	147.5	-1.1476	0.00076	0.0026
ENSRNOG00000024818	Eva1b	257.98011	272.071	92.3573	146.57	192.2	-1.147153	0.00028	0.00106
ENSRNOG00000007489	Zfp41	846.17477	779.866	282.091	451.61	589.9	-1.14674	2.6E-06	1.5E-05
ENSRNOG00000016152	Dek	3039.0057	3054.04	957.705	1794.5	2211	-1.146047	1.6E-06	9.5E-06
ENSRNOG00000019179	Ggta1	2345.5552	2398.79	880.406	1263.2	1722	-1.145591	6.8E-09	6.3E-08
ENSRNOG00000019832	Bbs1	935.95185	875.402	308.192	510.24	657.4	-1.144839	2.8E-06	1.6E-05
ENSRNOG00000046245	AABR07057423.1	297.19309	344.761	109.423	180.46	233	-1.144666	0.00018	0.00071
ENSRNOG00000030034	Sox11	1388.9649	1528.58	547.117	772.23	1059	-1.144145	3.4E-08	2.8E-07
ENSRNOG00000007726	Mcam	1188.7724	1257.55	548.12	559.71	888.5	-1.142802	4.5E-10	5E-09
ENSRNOG00000029735	Pid1	4598.2375	4481.89	1543.97	2570.4	3299	-1.141684	1.1E-07	7.9E-07
ENSRNOG00000016243	Casq2	15794.574	15736.5	7112.52	7181.8	11456	-1.141325	1.8E-15	4.1E-14
ENSRNOG00000020721	Nme6	278.61852	235.725	86.334	146.57	186.8	-1.140452	0.00053	0.00192
ENSRNOG00000056141	Matn3	67.07483	76.8443	35.1359	30.23	52.32	-1.139746	0.02338	0.05346
ENSRNOG00000010668	Anxa6	21619.765	20569.4	8891.4	10267	15337	-1.138846	6.6E-14	1.2E-12
ENSRNOG00000016374	Fgfr2	2950.2606	2908.66	1096.24	1563.7	2130	-1.138783	4E-09	3.8E-08
ENSRNOG00000009636	Scrn1	7811.6378	7716.63	2821.92	4231.2	5645	-1.138358	3.9E-09	3.7E-08

ENSRNOG00000069663	Arl4c	1743.9456	1628.27	669.59	862	1226	-1.138084	2.3E-09	2.2E-08
ENSRNOG00000027540	Fam102b	2254.7462	2255.49	964.732	1084.6	1640	-1.137786	1.5E-11	2.1E-10
ENSRNOG00000016477	Vangl1	1793.4778	1957.45	783.029	923.38	1364	-1.135891	2.3E-10	2.7E-09
ENSRNOG00000006723	Itga11	16827.527	16865.3	6421.84	8912.2	12257	-1.135609	1.6E-10	1.9E-09
ENSRNOG00000001457	Rfc2	1367.2946	1231.59	498.93	683.37	945.3	-1.135503	4E-08	3.2E-07
ENSRNOG00000013744	Akip1	1863.6483	1689.54	628.431	989.33	1293	-1.134371	2.3E-07	1.6E-06
ENSRNOG00000004585	Tmtc2	53.659864	62.3062	29.1126	23.817	42.22	-1.133025	0.04147	0.08638
ENSRNOG00000062466	ENSRNOG0000006246	663.52485	647.985	233.905	363.67	477.3	-1.132522	5E-06	2.7E-05
ENSRNOG00000003472	ENSRNOG0000000347	1270.2941	1400.85	518.004	699.86	972.3	-1.132332	2.8E-08	2.3E-07
ENSRNOG00000018683	Dock1	4110.1392	4165.17	1560.04	2215	3013	-1.131983	2E-09	2E-08
ENSRNOG00000026840	Thap3	211.54369	190.034	82.3185	100.77	146.2	-1.131906	0.00052	0.00188
ENSRNOG00000014645	Aldh7a1	3762.382	3519.26	1468.68	1854.1	2651	-1.131583	8.3E-11	1E-09
ENSRNOG00000052745	Met	2950.2606	2918.01	1382.35	1297.1	2137	-1.131106	1.7E-12	2.7E-11
ENSRNOG00000021156	Vegfb	428.24699	412.26	177.687	206.11	306.1	-1.13012	4.9E-06	2.7E-05
ENSRNOG00000034254	Actb	350843.67	351061	156350	164555	3E+05	-1.129131	1.2E-15	2.8E-14
ENSRNOG00000016274	Zfp580	199.16065	214.956	78.3029	110.84	150.8	-1.128448	0.00066	0.00229
ENSRNOG00000012683	Abhd17c	1336.337	1413.31	485.88	771.31	1002	-1.128113	5.4E-07	3.5E-06
ENSRNOG00000010214	Scrn2	231.15018	245.071	102.396	115.42	173.5	-1.127733	0.00018	0.00072
ENSRNOG00000016719	Zfp161	552.07744	452.759	161.625	297.72	366	-1.127401	0.00011	0.00047
ENSRNOG00000069968	ENSRNOG0000006996	66.042909	65.4215	28.1087	32.062	47.91	-1.126549	0.0306	0.06704
ENSRNOG00000061085	Fancd2	2094.7985	1929.42	714.765	1127.7	1467	-1.126433	2E-07	1.4E-06
ENSRNOG00000002563	Mcts1	471.58765	455.874	171.664	252.83	338	-1.125866	1.6E-05	7.9E-05

ENSRNOG00000022030	Aunip	251.78859	253.379	78.3029	152.98	184.1	-1.124121	0.00111	0.00368
ENSRNOG00000005713	Ccdc82	310.60806	396.683	124.482	199.7	257.9	-1.123439	0.00018	0.00071
ENSRNOG00000030127	Eml2	1959.6169	1998.99	794.072	1023.2	1444	-1.122673	1.5E-09	1.5E-08
ENSRNOG00000012826	Creb3l2	6072.8519	6084.2	2544.84	3037.6	4435	-1.122659	2.8E-12	4.3E-11
ENSRNOG00000064636	ENSRNOG0000006463	76.362114	94.4978	25.0971	53.131	62.27	-1.122547	0.02675	0.05985
ENSRNOG00000001492	Slc8a2	664.55677	576.333	230.893	338.94	452.7	-1.121387	5.5E-06	3E-05
ENSRNOG00000049277	Syne4	123.83045	165.112	62.2408	70.536	105.4	-1.120883	0.00303	0.009
ENSRNOG00000023465	Depp1	421.02355	399.798	189.734	187.79	299.6	-1.120568	4.9E-06	2.7E-05
ENSRNOG00000003081	ENSRNOG0000000308	85.649398	73.729	18.0699	54.963	58.1	-1.120278	0.04444	0.09154
ENSRNOG00000008907	Fggy	370.45944	319.839	142.551	174.97	252	-1.119306	3.5E-05	0.00016
ENSRNOG00000001192	Gltp	1627.3386	1615.81	566.19	926.13	1184	-1.119014	7E-07	4.4E-06
ENSRNOG00000008613	Gatad1	2170.1287	2079.99	882.414	1074.5	1552	-1.118495	3.1E-10	3.5E-09
ENSRNOG00000024757	Endod1	1380.7096	1390.47	599.318	676.96	1012	-1.118183	7.7E-10	8.2E-09
ENSRNOG00000008704	Tmem97	1235.2088	1338.55	578.237	607.34	939.8	-1.118115	9.5E-10	1E-08
ENSRNOG00000037449	Pole	2682.9932	2879.59	910.522	1651.6	2031	-1.117856	1.9E-06	1.1E-05
ENSRNOG00000001316	Anapc5	16477.706	16141.5	6585.48	8446	11913	-1.117657	1.5E-11	2E-10
ENSRNOG00000016687	Ssc5d	618.12035	708.214	260.006	350.85	484.3	-1.117383	1.6E-06	9.8E-06
ENSRNOG00000012442	Cemip	267.2674	209.764	104.404	115.42	174.2	-1.117175	0.0003	0.00113
ENSRNOG00000038330	RGD1359508	184.71376	136.035	66.2563	81.528	117.1	-1.116718	0.00234	0.00716
ENSRNOG00000031612	Gls2	625.3438	712.368	284.099	332.53	488.6	-1.11661	3.8E-07	2.6E-06
ENSRNOG00000013577	Kdelr3	5558.9555	5314.72	2476.58	2538.4	3972	-1.116505	1.6E-13	2.9E-12
ENSRNOG00000011946	Ptn	69.13867	76.8443	38.1476	29.314	53.36	-1.115571	0.02591	0.05824

ENSRNOG00000009951	Aif1l	1929.6913	1940.84	790.056	995.75	1414	-1.11546	1.1E-09	1.2E-08
ENSRNOG00000003479	Rnf150	1708.8603	1756	599.318	999.41	1266	-1.115081	9.4E-07	5.9E-06
ENSRNOG00000015386	ENSRNOG0000001538	122.79853	90.344	33.1282	65.04	77.83	-1.114714	0.01636	0.03912
ENSRNOG00000050298	Rfng	758.46153	805.827	330.278	392.07	571.7	-1.114044	1E-07	7.9E-07
ENSRNOG00000025731	Ptma	1609.7959	1475.62	717.777	709.02	1128	-1.112727	2.9E-10	3.3E-09
ENSRNOG00000006469	Cdk2	1558.1999	1632.42	586.268	889.48	1167	-1.111597	2.1E-07	1.5E-06
ENSRNOG00000010584	Tmem123	6083.1711	5926.36	2600.06	2960.7	4393	-1.110709	1.1E-12	1.7E-11
ENSRNOG00000046023	Exoc4	3479.6358	3594.03	1481.73	1793.6	2587	-1.110537	4.1E-11	5.3E-10
ENSRNOG00000018087	Vim	112226.51	110688	43897.8	59406	81555	-1.109587	9.7E-11	1.2E-09
ENSRNOG00000067317	Cmtm7	1493.1889	1505.73	637.466	752.08	1097	-1.109374	1.3E-09	1.3E-08
ENSRNOG00000026917	AABR07021573.2	88.745159	58.1525	48.1864	20.153	53.81	-1.108987	0.04699	0.09585
ENSRNOG00000029459	AABR07021591.1	168.20303	197.303	71.2757	98.017	133.7	-1.108386	0.00138	0.00447
ENSRNOG00000043143	Eml1	7572.2323	7665.74	3213.43	3856.6	5577	-1.107757	4E-12	6E-11
ENSRNOG00000014362	Nudt14	280.68236	235.725	97.3767	141.99	188.9	-1.107324	0.0004	0.00146
ENSRNOG00000009955	Plch1	68.10675	85.1518	39.1515	32.062	56.12	-1.107135	0.02418	0.055
ENSRNOG00000007062	Rin3	2087.5751	2166.18	816.157	1159.7	1557	-1.105659	2.4E-08	2E-07
ENSRNOG00000020325	Calhm2	2315.6295	2290.79	945.658	1194.5	1687	-1.105474	8E-10	8.5E-09
ENSRNOG00000004518	Cacnb1	127.95814	122.536	53.2058	63.207	91.73	-1.104329	0.00478	0.0135
ENSRNOG00000067120	Paqr8	1666.5515	1562.85	609.357	892.23	1183	-1.104033	1.3E-07	9.3E-07
ENSRNOG00000047692	Hells	2012.2449	1942.92	710.75	1128.6	1449	-1.10388	3.6E-07	2.4E-06
ENSRNOG00000019922	Ntpcr	369.42752	384.222	106.412	243.67	275.9	-1.103665	0.00105	0.00348
ENSRNOG00000019648	Col6a3	206.38409	220.149	102.396	96.185	156.3	-1.103316	0.00041	0.0015

ENSRNOG00000066480	Lsm6	1115.506	1018.71	417.616	576.2	782	-1.101759	2.3E-07	1.7E-06
ENSRNOG00000008932	Ncapd3	2322.8529	2210.83	818.165	1293.5	1661	-1.101725	2.7E-07	1.9E-06
ENSRNOG00000025843	Ccdc102a	1593.2852	1792.34	720.788	856.51	1241	-1.101544	2.1E-09	2.1E-08
ENSRNOG00000057945	Fancg	345.69335	333.338	137.532	178.63	248.8	-1.101418	5.1E-05	0.00023
ENSRNOG00000019271	Ulk4	170.26688	187.957	50.1942	116.34	131.2	-1.101328	0.00635	0.01727
ENSRNOG00000025695	Tns3	7384.4228	7311.64	3574.83	3275.8	5387	-1.101196	2.7E-13	4.7E-12
ENSRNOG00000016989	Dolk	990.64364	858.788	340.317	521.23	677.7	-1.10095	2.7E-06	1.6E-05
ENSRNOG00000000437	Agpat1	2988.4416	3130.89	1272.92	1580.2	2243	-1.100509	2.3E-10	2.7E-09
ENSRNOG00000016338	Cibar1	777.0361	809.981	334.293	405.81	581.8	-1.099755	1.6E-07	1.2E-06
ENSRNOG00000014170	Dbn1	9740.2972	9747.81	3938.24	5154.6	7145	-1.099657	1.1E-10	1.3E-09
ENSRNOG00000028585	ENSRNOG0000002858	3902.7232	4070.67	1916.41	1804.6	2924	-1.099576	1.8E-12	2.8E-11
ENSRNOG00000010640	Agtr1b	82.553636	91.3825	37.1437	43.97	63.76	-1.09928	0.01672	0.03986
ENSRNOG00000032929	Incenp	5656.9879	5517.22	2150.32	3065.1	4097	-1.099065	4.2E-09	4E-08
ENSRNOG00000042326	Smpdl3b	328.1507	386.299	156.606	176.8	262	-1.098869	3.1E-05	0.00015
ENSRNOG00000014278	Zfp710	943.1753	980.285	364.41	533.14	705.3	-1.098551	8.6E-07	5.4E-06
ENSRNOG00000007625	B3galt1	163.04343	225.341	58.2252	122.75	142.3	-1.098379	0.00509	0.01425
ENSRNOG00000019164	Uba1	9875.4788	9569.2	4216.31	4865.1	7132	-1.098294	1E-12	1.7E-11
ENSRNOG00000015544	ENSRNOG0000001554	343.62951	293.878	148.575	149.32	233.8	-1.097663	5.2E-05	0.00023
ENSRNOG00000000563	Adamts14	710.99319	685.369	299.157	353.6	512.3	-1.09636	3.1E-07	2.1E-06
ENSRNOG00000015664	Tmem8b	391.09785	408.106	176.684	196.95	293.2	-1.096335	1.1E-05	5.4E-05
ENSRNOG00000045795	Gatd1	677.97174	743.521	255.99	408.56	521.5	-1.095517	1.1E-05	5.6E-05
ENSRNOG00000020144	Rusf1	2569.4819	2548.32	900.484	1494.1	1878	-1.095206	6.4E-07	4.1E-06

ENSRNOG00000004072	Myo1c	35632.213	34735.7	14418.8	18541	25832	-1.094182	2.9E-11	3.8E-10
ENSRNOG00000015577	Lpar6	865.78126	857.749	332.285	474.51	632.6	-1.093964	1E-06	6.3E-06
ENSRNOG00000036876	Faap20	223.92674	208.726	71.2757	131	158.7	-1.093958	0.0019	0.00594
ENSRNOG00000046652	Zfp84	4071.9581	4248.25	1952.55	1946.6	3055	-1.093458	1.2E-12	2E-11
ENSRNOG00000011887	RGD1307929	6842.6645	6852.65	2763.69	3654.1	5028	-1.09334	3.1E-10	3.5E-09
ENSRNOG00000018610	Pde6d	755.36577	832.827	305.181	439.7	583.3	-1.09111	2.2E-06	1.3E-05
ENSRNOG00000070355	Tedc1	836.88749	876.441	373.445	430.54	629.3	-1.090997	6.9E-08	5.4E-07
ENSRNOG00000020751	Rdm1	385.93825	355.145	188.73	159.39	272.3	-1.090972	2.5E-05	0.00012
ENSRNOG00000001414	Serpine1	4895.4306	5084.19	2227.62	2458.7	3666	-1.090422	2.3E-12	3.5E-11
ENSRNOG00000055089	Slc44a1	2358.9702	2493.29	1058.09	1221.1	1783	-1.089851	1.4E-10	1.7E-09
ENSRNOG00000012865	Parp3	1431.2737	1293.89	559.163	720.93	1001	-1.089449	3.4E-08	2.8E-07
ENSRNOG00000015880	Dpep1	172.33072	172.381	95.3689	66.872	126.7	-1.089446	0.00201	0.00625
ENSRNOG00000024886	Ext1	6453.6305	6388.47	2987.56	3050.4	4720	-1.088717	2.6E-13	4.6E-12
ENSRNOG00000024345	Pard3b	1078.3569	1015.59	412.596	571.61	769.5	-1.088261	3.3E-07	2.2E-06
ENSRNOG00000001692	Chaf1b	1114.4741	991.707	347.344	643.07	774.1	-1.087368	2.6E-05	0.00012
ENSRNOG00000008332	Smo	3164.9	3218.12	1272.92	1730.4	2347	-1.087282	4.1E-09	3.9E-08
ENSRNOG00000026059	Paqr6	70.170591	58.1525	31.1204	29.314	47.19	-1.086848	0.03892	0.08201
ENSRNOG00000069265	Purb	10350.162	10118.5	4029.59	5606.2	7526	-1.086803	1.5E-09	1.5E-08
ENSRNOG00000011078	Srm	1833.7226	1783	707.738	994.83	1330	-1.086325	5.2E-08	4.1E-07
ENSRNOG00000020883	Dhdh	155.81999	136.035	38.1476	98.933	107.2	-1.086078	0.01429	0.03477
ENSRNOG00000004841	Akap6	2257.842	2034.3	997.86	1025.1	1579	-1.085206	1.3E-10	1.6E-09
ENSRNOG00000016494	Tmem204	264.17164	203.534	87.3379	132.83	172	-1.084904	0.00106	0.00352

ENSRNOG00000008298	Dock7	3587.9874	3235.77	1275.94	1942	2510	-1.084019	1.1E-07	8.2E-07
ENSRNOG00000024338	ENSRNOG0000002433	110.41549	143.304	46.1786	73.284	93.3	-1.083825	0.00873	0.02276
ENSRNOG00000056226	Cenpq	576.84353	607.486	223.866	334.36	435.6	-1.083667	1.2E-05	6.2E-05
ENSRNOG00000010652	Dock6	1101.0591	1213.93	447.732	643.98	851.7	-1.083491	5.4E-07	3.5E-06
ENSRNOG00000060350	Iqsec1	11084.89	11635.7	4339.79	6394.9	8364	-1.081582	1.1E-08	9.8E-08
ENSRNOG00000012236	Hddc3	356.01256	391.491	130.505	222.6	275.2	-1.079802	0.00025	0.00097
ENSRNOG00000002462	B9d1	262.1078	259.609	106.412	140.16	192.1	-1.079708	0.00028	0.00108
ENSRNOG00000022943	Dgka	935.95185	951.208	412.596	480.01	694.9	-1.079566	4.9E-08	3.9E-07
ENSRNOG00000017628	Tagln	68743.445	67084.1	30867.4	33422	50029	-1.079113	5.8E-14	1.1E-12
ENSRNOG00000067705	Klf2	492.22606	529.603	228.885	254.66	376.3	-1.078885	2.9E-06	1.7E-05
ENSRNOG00000047741	E2f2	366.33176	342.684	93.3612	241.84	261.1	-1.078184	0.00273	0.00819
ENSRNOG00000021231	Lzts3	508.73678	583.602	237.92	279.4	402.4	-1.077546	3.4E-06	1.9E-05
ENSRNOG00000011498	Psip1	2469.3856	2679.17	949.674	1489.5	1897	-1.077244	3.6E-07	2.4E-06
ENSRNOG00000000195	Vwa5a	2527.1732	2558.71	986.818	1424.5	1874	-1.076197	4.9E-08	3.9E-07
ENSRNOG00000008812	Tmem214	4145.2245	4063.4	1652.39	2240.7	3025	-1.075934	3.2E-09	3.1E-08
ENSRNOG00000019482	Gnao1	610.89691	600.217	204.792	369.17	446.3	-1.07559	8.1E-05	0.00035
ENSRNOG00000014689	Zbtb4	1367.2946	1430.97	542.097	785.05	1031	-1.075384	3.1E-07	2.1E-06
ENSRNOG00000019986	Pot1	747.11041	679.138	230.893	445.2	525.6	-1.075367	0.00011	0.00045
ENSRNOG00000001094	Zfp316	133.11774	146.42	47.1825	85.193	103	-1.075046	0.0078	0.02063
ENSRNOG00000003613	Mid1	1135.1125	1143.32	502.946	578.94	840.1	-1.074023	1.5E-08	1.3E-07
ENSRNOG00000031801	Ephb3	1684.0942	1726.92	842.258	778.64	1258	-1.073613	5.3E-10	5.8E-09
ENSRNOG00000006079	Psd4	72.234432	85.1518	39.1515	35.726	58.07	-1.072326	0.02508	0.05669

ENSRNOG00000028845	Ebf1	1040.1758	1066.47	486.884	514.82	777.1	-1.072291	1.2E-08	1.1E-07
ENSRNOG00000046949	Kcnb1	721.3124	751.828	265.025	435.12	543.3	-1.071729	1.8E-05	9E-05
ENSRNOG00000010760	E2f5	535.56672	549.333	280.084	236.34	400.3	-1.071727	2.8E-06	1.6E-05
ENSRNOG00000000638	Zfp365	859.58974	808.943	358.386	435.12	615.5	-1.071544	2.5E-07	1.7E-06
ENSRNOG00000011306	Myod1	2859.4516	2686.44	963.728	1674.5	2046	-1.071313	2.5E-06	1.4E-05
ENSRNOG00000067963	ENSRNOG0000006796	272.427	282.455	95.3689	168.55	204.7	-1.069491	0.0009	0.00304
ENSRNOG00000003508	Ergic1	4682.855	4628.31	1769.85	2666.6	3437	-1.069245	6.1E-08	4.7E-07
ENSRNOG00000024967	Uqcrb	2627.2695	2625.17	1256.86	1249.5	1940	-1.067409	2.9E-11	3.9E-10
ENSRNOG00000057880	Myh11	2290.8634	2403.98	864.344	1375.9	1734	-1.066838	6.7E-07	4.3E-06
ENSRNOG00000065213	Ccdc85c	1101.0591	1013.51	469.818	539.55	781	-1.066454	3.9E-08	3.1E-07
ENSRNOG00000005918	RGD1563941	132.08582	105.921	52.2019	61.375	87.9	-1.066291	0.00823	0.02157
ENSRNOG00000004442	Dglucy	1184.6447	1282.47	441.709	736.5	911.3	-1.065228	6.3E-06	3.4E-05
ENSRNOG00000028640	Siva1	694.48247	586.717	264.021	348.1	473.3	-1.064582	5.3E-06	2.9E-05
ENSRNOG00000014480	Dbndd2	3081.3145	3010.43	1375.32	1537.1	2251	-1.064434	5.5E-11	7E-10
ENSRNOG00000002537	ENSRNOG0000000253	1472.5505	1586.73	649.513	813.45	1131	-1.063739	2E-08	1.7E-07
ENSRNOG00000014846	Anp32a	4799.462	5049.92	2030.86	2681.3	3640	-1.063405	1.7E-09	1.7E-08
ENSRNOG00000026857	Kif7	728.53584	744.559	322.247	382.91	544.6	-1.062134	4.9E-07	3.2E-06
ENSRNOG00000017595	Rsu1	2568.45	2355.18	1098.25	1259.6	1820	-1.062016	4.7E-10	5.2E-09
ENSRNOG00000002934	Atp1b1	5897.4254	5810.06	2530.79	3079.8	4330	-1.061046	8.1E-11	1E-09
ENSRNOG00000022448	Htra2	2414.6939	2281.45	967.744	1282.5	1737	-1.060952	1.3E-08	1.1E-07
ENSRNOG00000004737	Cd48	1003.0267	888.902	444.72	462.6	699.8	-1.060038	6.7E-08	5.2E-07
ENSRNOG00000009550	Sqle	2687.1209	2694.74	1396.4	1185.4	1991	-1.060035	3.5E-10	3.9E-09

ENSRNOG00000048747	Cdk5rap3	1169.1659	1139.17	480.86	626.58	853.9	-1.05887	1.4E-07	1E-06
ENSRNOG00000003703	Mcm6	7463.8807	7354.21	2993.58	4120.4	5483	-1.058451	3.7E-09	3.5E-08
ENSRNOG00000004696	ENSRNOG0000000469	4382.5662	4475.66	1906.37	2348.8	3278	-1.057588	2.7E-10	3.1E-09
ENSRNOG00000016846	Pik3cd	1751.169	1790.27	730.827	971.93	1311	-1.055874	3.8E-08	3.1E-07
ENSRNOG00000006227	Ifih1	300.28885	337.492	131.509	174.97	236.1	-1.055783	0.00017	0.00067
ENSRNOG00000058805	Aida	6324.6405	6208.82	2725.54	3310.6	4642	-1.053926	8.9E-11	1.1E-09
ENSRNOG00000016581	Serpinb1a	68.10675	66.46	28.1087	36.642	49.83	-1.053446	0.04073	0.08508
ENSRNOG00000024411	Cbr4	212.57561	195.226	63.2447	132.83	151	-1.053231	0.00497	0.01398
ENSRNOG00000013231	Ptafr	72.234432	80.9981	25.0971	48.551	56.72	-1.052875	0.0403	0.08439
ENSRNOG00000004980	Rangrf	284.81005	235.725	103.4	147.48	192.9	-1.051158	0.00067	0.00234
ENSRNOG00000054331	Wdpcp	184.71376	181.726	83.3223	93.437	135.8	-1.05105	0.00146	0.00471
ENSRNOG00000017561	Toe1	362.20408	389.414	131.509	230.84	278.5	-1.050406	0.00041	0.00152
ENSRNOG00000070030	ENSRNOG0000007003	78.425955	61.2678	26.101	41.222	51.75	-1.050092	0.04405	0.09098
ENSRNOG00000000440	Pbx2	1150.5913	1106.97	493.911	596.35	837	-1.049512	6.3E-08	4.9E-07
ENSRNOG00000010150	Strbp	1183.6128	1232.62	437.693	729.18	895.8	-1.049114	8.4E-06	4.4E-05
ENSRNOG00000018241	Ank1	1067.0058	1054.01	449.74	575.28	786.5	-1.048353	2.1E-07	1.5E-06
ENSRNOG00000016774	Telo2	631.53532	625.139	248.963	358.18	466	-1.048183	1.1E-05	5.8E-05
ENSRNOG00000049758	Tbc1d16	929.76033	1014.55	465.802	475.43	721.4	-1.046552	5.6E-08	4.4E-07
ENSRNOG00000011669	Gstcd	611.92883	695.753	294.138	338.94	485.2	-1.045944	1.8E-06	1.1E-05
ENSRNOG00000003526	ENSRNOG0000000352	575.81161	564.91	238.924	313.29	423.2	-1.045512	7.7E-06	4E-05
ENSRNOG00000033747	Sp110	502.54526	512.988	242.94	249.17	376.9	-1.045068	4.2E-06	2.3E-05
ENSRNOG00000003848	Med12	2416.7577	2600.25	937.627	1493.2	1862	-1.044864	1.1E-06	6.7E-06

ENSRNOG00000009199	Accs	403.4809	411.221	130.505	263.82	302.3	-1.044613	0.00081	0.00276
ENSRNOG00000006324	ENSRNOG0000000632	218.76714	266.878	115.447	120	180.3	-1.044212	0.00051	0.00183
ENSRNOG00000009250	Pts	180.58608	223.264	78.3029	117.25	149.9	-1.04398	0.00226	0.00693
ENSRNOG00000006720	Rnpep	3254.6771	3169.31	1272.92	1843.1	2385	-1.043362	8.3E-08	6.3E-07
ENSRNOG00000014142	Ogfrl1	342.59759	400.837	150.583	209.78	275.9	-1.043196	0.00014	0.00056
ENSRNOG00000006864	Scaper	234.24594	237.802	79.3068	149.32	175.2	-1.043097	0.00248	0.00753
ENSRNOG00000068672	ENSRNOG0000006867	171.2988	176.534	87.3379	81.528	129.2	-1.042959	0.00192	0.00599
ENSRNOG00000022260	Senp1	2265.0654	2400.87	1022.96	1241.2	1733	-1.042798	2.9E-09	2.8E-08
ENSRNOG00000030597	Ankrd52	3438.359	3591.95	1646.37	1768	2611	-1.041869	4.3E-11	5.6E-10
ENSRNOG00000023661	Celf2	2315.6295	2343.75	906.507	1355.8	1730	-1.041823	3.3E-07	2.3E-06
ENSRNOG00000023130	Lyplal1	243.53323	210.803	78.3029	141.99	168.7	-1.041601	0.00263	0.00793
ENSRNOG00000020652	Tgfb1	778.06802	728.983	346.34	385.66	559.8	-1.041389	4.6E-07	3E-06
ENSRNOG00000016021	Lims2	1544.7849	1551.43	623.412	880.32	1150	-1.041239	3E-07	2.1E-06
ENSRNOG00000021752	Foxq1	147.56463	172.381	56.2175	98.933	118.8	-1.041076	0.00665	0.01796
ENSRNOG00000048101	Zfp397	130.02198	148.497	73.2835	62.291	103.5	-1.039727	0.00533	0.01484
ENSRNOG00000018785	Slc16a13	1024.697	1095.55	381.476	649.48	787.8	-1.039139	1.9E-05	9.4E-05
ENSRNOG00000018584	Ptma	29538.723	30042	12630.9	16372	22146	-1.038625	3.7E-10	4.2E-09
ENSRNOG00000010551	Lhx2	267.2674	284.532	115.447	152.98	205.1	-1.038053	0.00037	0.00139
ENSRNOG00000020753	Bnip1	453.01308	366.568	172.668	226.26	304.6	-1.03755	8.5E-05	0.00037
ENSRNOG00000000262	Zfp821	913.2496	831.788	360.394	489.17	648.7	-1.037508	2E-06	1.2E-05
ENSRNOG00000060063	ENSRNOG0000006006	825.53636	879.556	403.561	427.8	634.1	-1.036073	1.7E-07	1.3E-06
ENSRNOG00000053070	AABR07053749.2	274.49084	306.339	138.536	144.74	216	-1.035648	0.00018	0.00073

ENSRNOG00000053450	Myadm	17083.443	17642	7267.11	9673.5	12917	-1.035433	1.5E-09	1.5E-08
ENSRNOG00000012721	Ednra	264.17164	261.686	101.392	154.81	195.5	-1.035219	0.00081	0.00276
ENSRNOG00000011060	Unc119	632.56724	637.6	229.889	389.32	472.3	-1.034942	7.3E-05	0.00032
ENSRNOG00000015505	Mfap5	1154.719	1045.71	543.101	531.31	818.7	-1.034325	3.9E-08	3.1E-07
ENSRNOG00000010685	Tbx18	2132.9796	2326.1	1065.12	1112.1	1659	-1.03417	5.9E-10	6.5E-09
ENSRNOG00000019936	Anapc15	2006.0534	1955.38	983.806	950.86	1474	-1.034021	6.1E-10	6.6E-09
ENSRNOG00000004711	Mta1	3122.5913	3184.89	1271.92	1808.3	2347	-1.033636	7.1E-08	5.5E-07
ENSRNOG00000055300	Ncapd2	4344.3851	4323.01	2159.35	2081.3	3227	-1.031373	1.6E-11	2.2E-10
ENSRNOG00000008659	Arhgap21	4983.1439	5330.3	2374.18	2671.2	3840	-1.031364	5.9E-11	7.4E-10
ENSRNOG00000012534	Mfsd10	1759.4244	1739.38	748.897	962.77	1303	-1.030925	3.8E-08	3.1E-07
ENSRNOG00000020623	Aldh16a1	2795.4725	2721.74	1247.83	1453.8	2055	-1.029867	7.8E-10	8.3E-09
ENSRNOG00000029939	Gypc	1288.8686	1333.35	562.175	721.85	976.6	-1.02946	1.4E-07	1E-06
ENSRNOG00000007821	ENSRNOG0000000782	2305.3103	2607.52	1086.2	1320.9	1830	-1.02889	7.9E-09	7.2E-08
ENSRNOG00000006076	Steap2	1925.5636	1983.41	763.955	1151.5	1456	-1.028488	8E-07	5.1E-06
ENSRNOG00000015297	RGD1561662	649.07797	636.562	267.033	362.76	478.9	-1.028387	8.2E-06	4.3E-05
ENSRNOG00000005376	Mad2l1	1772.8393	1814.15	770.983	987.5	1336	-1.027917	3.6E-08	2.9E-07
ENSRNOG00000055564	RGD1564664	796.64259	827.634	259.002	536.8	605	-1.027884	0.00028	0.00108
ENSRNOG00000047867	Klhdc8b	860.62166	795.443	286.107	525.81	617	-1.027001	9.8E-05	0.00042
ENSRNOG00000049402	Nbl1	1651.0727	1616.85	670.594	932.54	1218	-1.026817	2.8E-07	1.9E-06
ENSRNOG00000011300	Lama3	458.17268	570.102	180.699	323.37	383.1	-1.026759	0.00034	0.00127
ENSRNOG00000006048	Ezh2	3699.4348	3822.49	1422.5	2270	2804	-1.02614	9.2E-07	5.7E-06
ENSRNOG00000012512	Nexn	7062.4636	7723.9	3259.61	4003.1	5512	-1.025542	5.4E-10	5.9E-09

ENSRNOG00000006570	Plekhg3	888.48351	861.903	389.507	469.93	652.5	-1.025534	4.8E-07	3.1E-06
ENSRNOG00000034026	Lclat1	2996.697	2937.74	1327.13	1588.4	2212	-1.025056	1.3E-09	1.3E-08
ENSRNOG00000006810	Lancl2	1379.6776	1354.12	633.451	710.85	1020	-1.023712	1.6E-08	1.4E-07
ENSRNOG00000067441	H1f2	620.18419	619.947	191.742	417.72	462.4	-1.023156	0.00069	0.0024
ENSRNOG00000015898	Fer	1230.0492	1203.55	520.012	676.96	907.6	-1.022989	2.8E-07	1.9E-06
ENSRNOG00000003707	Zmym3	887.45159	906.556	367.421	515.74	669.3	-1.021422	3.2E-06	1.8E-05
ENSRNOG00000021170	Plekho1	964.84563	883.71	439.701	470.85	689.8	-1.021348	1.8E-07	1.3E-06
ENSRNOG00000010278	Il6	212.57561	193.149	110.427	89.773	151.5	-1.020345	0.00149	0.00478
ENSRNOG00000009116	Itgb3bp	331.24647	353.069	159.617	177.71	255.4	-1.019889	9.2E-05	0.0004
ENSRNOG00000067086	Calm2	19941.863	19488.3	9561.99	9886	14720	-1.019669	1.1E-12	1.8E-11
ENSRNOG00000003563	Blmh	2129.8838	2152.68	852.297	1259.6	1599	-1.019381	5.5E-07	3.5E-06
ENSRNOG00000001215	Cfap410	462.30036	426.798	217.843	220.77	331.9	-1.019349	1.8E-05	8.8E-05
ENSRNOG00000021117	Rps6ka4	2740.7807	2730.05	1175.55	1524.3	2043	-1.018494	1.5E-08	1.3E-07
ENSRNOG00000016733	Rab13	717.18472	728.983	365.414	349.01	540.1	-1.017566	7E-07	4.5E-06
ENSRNOG00000024207	Fgfrl1	395.22553	420.567	184.715	218.02	304.6	-1.017518	4.3E-05	0.0002
ENSRNOG00000018553	Pitpnm1	2229.9801	2129.83	1013.92	1139.6	1628	-1.01735	1.9E-09	1.9E-08
ENSRNOG00000006460	Amdhd2	765.68498	685.369	307.188	409.47	541.9	-1.016737	6.1E-06	3.3E-05
ENSRNOG00000013938	Tshz3	1140.2721	1265.85	520.012	668.72	898.7	-1.016607	4E-07	2.7E-06
ENSRNOG00000068020	Smim4	227.0225	190.034	118.458	87.941	155.9	-1.016548	0.00186	0.00585
ENSRNOG00000027787	Cdc6	998.899	920.055	310.2	637.57	716.7	-1.016481	0.00024	0.00094
ENSRNOG00000008628	Ica1	451.98116	453.797	127.493	319.7	338.2	-1.016096	0.00274	0.00822
ENSRNOG00000007300	C1qtnf6	3810.8822	3592.99	1818.03	1844	2766	-1.015615	7.5E-11	9.4E-10

ENSRNOG00000069303	Sh3bgrl	12515.131	12755.1	5449.08	7054.5	9443	-1.015003	1.4E-09	1.4E-08
ENSRNOG00000003217	Lgals3bp	1850.2334	1837	799.091	1025.1	1378	-1.014802	4.8E-08	3.8E-07
ENSRNOG00000047734	Chst2	1686.158	1704.08	636.462	1040.6	1267	-1.014674	6E-06	3.2E-05
ENSRNOG00000000474	Rgl2	981.35635	1111.13	512.985	523.06	782.1	-1.014043	1.2E-07	8.9E-07
ENSRNOG00000004090	ENSRNOG0000000409	99.064364	87.2287	50.1942	42.138	69.66	-1.013915	0.02173	0.05019
ENSRNOG00000045913	Prdm16	809.02564	855.672	306.184	517.57	622.1	-1.013679	4.9E-05	0.00022
ENSRNOG00000013069	Sapcd2	1317.7624	1238.86	563.179	702.61	955.6	-1.013605	1.6E-07	1.2E-06
ENSRNOG00000014771	Wfdc8	301.32077	326.069	136.528	174.05	234.5	-1.013101	0.00025	0.00095
ENSRNOG00000017804	Septin1	100.09628	61.2678	46.1786	33.894	60.36	-1.013025	0.04137	0.08622
ENSRNOG00000000483	Syngap1	992.70748	1151.63	406.573	656.81	801.9	-1.010841	2.1E-05	0.0001
ENSRNOG00000032813	Mdc1	876.10047	829.711	279.08	567.03	638	-1.010189	0.00028	0.00108
ENSRNOG00000021669	Mybl1	2164.9691	2453.83	1051.07	1242.2	1728	-1.009824	1.1E-08	9.6E-08
ENSRNOG00000008409	Myo1f	223.92674	246.11	88.3418	144.74	175.8	-1.009503	0.00212	0.00657
ENSRNOG00000021137	Kctd15	2234.1078	2200.45	781.021	1420.8	1659	-1.009485	2.1E-05	0.0001
ENSRNOG00000069246	Abcg3l3	186.7776	186.919	77.299	108.09	139.8	-1.009304	0.00284	0.00849
ENSRNOG00000064183	Amigo3	518.02407	558.679	276.068	259.24	403	-1.008457	7E-06	3.7E-05
ENSRNOG00000015206	Alad	543.82208	495.335	190.738	325.2	388.8	-1.008421	0.00022	0.00087
ENSRNOG00000011413	Scp2	3017.3354	2869.2	1445.59	1481.3	2203	-1.008034	2.5E-10	2.8E-09
ENSRNOG00000002215	Mylk	9539.0727	9469.51	4122.95	5328.7	7115	-1.007894	2.2E-09	2.1E-08
ENSRNOG00000005126	Slc66a3	5001.7184	5154.8	2129.24	2921.3	3802	-1.007655	2.9E-08	2.4E-07
ENSRNOG00000003407	Ptpn14	3184.5065	3078.97	1244.82	1870.6	2345	-1.007134	5.4E-07	3.5E-06
ENSRNOG00000011068	Papss2	1123.7614	1207.7	477.849	682.46	872.9	-1.005861	2.5E-06	1.4E-05

ENSRNOG00000017500	Mtss2	2833.6536	3251.35	1091.22	1938.4	2279	-1.005686	1.5E-05	7.4E-05
ENSRNOG00000017737	Dgkz	2159.8095	2193.18	930.6	1236.7	1630	-1.005645	7.9E-08	6E-07
ENSRNOG00000053203	Mical3	1704.7326	1668.77	694.687	984.75	1263	-1.005611	6.3E-07	4E-06
ENSRNOG00000026109	Prmt1	4446.5452	4383.24	2036.88	2360.7	3307	-1.005512	3.5E-10	3.9E-09
ENSRNOG00000023093	Mis18bp1	843.07901	862.941	408.581	441.54	639	-1.004599	3.7E-07	2.5E-06
ENSRNOG00000021153	Fkbp2	1236.2407	1183.82	549.124	656.81	906.5	-1.004371	1.3E-07	9.8E-07
ENSRNOG00000034013	Acaca	1715.0518	1904.49	774.998	1029.6	1356	-1.003549	2.2E-07	1.5E-06
ENSRNOG00000043105	Lyrm2	682.09942	587.755	226.878	406.73	475.9	-1.001468	0.00024	0.00092
ENSRNOG00000049780	Sri	3704.5944	3682.3	1620.27	2069.4	2769	-1.001209	8.6E-09	7.7E-08
ENSRNOG00000068826	Mgat1	5796.2972	5625.21	2585	3121	4282	-1.001044	7.4E-10	8E-09
ENSRNOG00000008577	Fbf1	636.69492	863.98	271.049	478.18	562.5	-1.000829	0.00025	0.00096
ENSRNOG00000028156	Pld1	1586.0617	1631.38	642.485	964.6	1206	-1.000743	2.3E-06	1.3E-05
ENSRNOG00000063594	ENSRNOG0000006359	376.65097	424.721	165.641	234.51	300.4	-1.000371	0.00018	0.00071
ENSRNOG00000056894	St6galnac3	182.64992	174.457	68.2641	109.93	133.8	-1.000342	0.00502	0.01408
ENSRNOG00000012360	Gtf2h1	1997.798	2127.76	5036.48	3220.8	3096	1.0009214	1.2E-06	7.4E-06
ENSRNOG00000046439	Vps37c	1531.37	1525.46	3564.79	2553.9	2294	1.0009963	1.4E-07	1E-06
ENSRNOG00000024036	Fam98c	113.51125	101.767	236.917	194.2	161.6	1.00119	0.00147	0.00472
ENSRNOG00000018057	Mrpl2	430.31083	442.374	983.806	763.99	655.1	1.0015695	2.2E-06	1.3E-05
ENSRNOG00000049714	Asap3	505.64102	536.872	1110.3	977.42	782.6	1.0016545	2E-07	1.5E-06
ENSRNOG00000070895	ENSRNOG0000007089	83.585557	93.4593	201.781	152.98	133	1.0018677	0.00363	0.01057
ENSRNOG00000050657	Crlf3	933.88801	818.288	2019.81	1495	1317	1.0039916	4.6E-07	3.1E-06
ENSRNOG00000001785	Etv5	1162.9744	1086.21	2518.74	1993.3	1690	1.0041722	3.4E-08	2.8E-07

ENSRNOG00000050543	Safb	3471.3804	3487.07	8061.19	5898.4	5230	1.0043306	2E-08	1.7E-07
ENSRNOG00000018375	Eif2b3	914.28152	955.362	1806.99	1943.9	1405	1.0045514	3.6E-09	3.5E-08
ENSRNOG00000007272	Rnf103	890.54735	953.285	2016.8	1683.7	1386	1.0048218	2.4E-08	2E-07
ENSRNOG00000018090	Ppp6r1	2868.7389	2818.32	6572.43	4842.2	4275	1.0050238	2.1E-08	1.8E-07
ENSRNOG00000003635	Disp1	637.72684	573.217	1339.18	1091.9	910.5	1.0051674	2.9E-07	2E-06
ENSRNOG00000015097	Kcmf1	2992.5693	3009.39	6852.51	5197.7	4513	1.0054493	8.3E-09	7.5E-08
ENSRNOG00000027724	Plekhf1	1019.5374	905.517	2061.98	1806.4	1448	1.0066821	1.3E-08	1.1E-07
ENSRNOG00000045843	Rusc2	4867.5688	4719.7	9980.61	9284.2	7213	1.0067497	1.1E-11	1.6E-10
ENSRNOG00000067931	Harbi1	123.83045	152.65	267.033	288.56	208	1.0071611	0.00043	0.00157
ENSRNOG00000017427	Pfn2	2447.7153	2300.14	4816.63	4727.7	3573	1.0073572	4.6E-11	6E-10
ENSRNOG00000059714	Hsp90aa1	24691.793	25060.6	63070	36945	37442	1.007366	2E-06	1.2E-05
ENSRNOG00000070904	ENSRNOG0000007090	154.78807	228.456	487.887	283.06	288.5	1.0075142	0.00135	0.00439
ENSRNOG00000031827	Arih2	1368.3265	1344.78	3000.61	2456.8	2043	1.0081192	5.8E-09	5.4E-08
ENSRNOG00000045686	Nfs1	1303.3155	1305.32	2738.59	2510	1964	1.008548	6.3E-10	6.8E-09
ENSRNOG00000033663	P4ha2	4068.8624	3895.18	7947.75	8075.9	5997	1.008633	8.5E-12	1.2E-10
ENSRNOG00000020063	Nfkbib	537.63056	582.563	1426.52	829.94	844.2	1.0098036	4E-05	0.00018
ENSRNOG00000002186	Nudt9	522.15175	614.755	1292	998.49	856.8	1.0102243	1.1E-06	6.8E-06
ENSRNOG00000020703	Sipa1l3	1704.7326	1753.92	3749.51	3219.9	2607	1.0107231	7.6E-10	8.1E-09
ENSRNOG00000048195	Nudt13	50.564102	61.2678	103.4	121.83	84.27	1.0107994	0.01361	0.03336
ENSRNOG00000058588	Nfxl1	732.66352	816.212	1663.44	1458.4	1168	1.010999	2.7E-08	2.2E-07
ENSRNOG00000019435	Psd	92.872841	104.882	234.909	163.97	149.2	1.0112076	0.00274	0.00822
ENSRNOG00000023768	Rundc1	588.19466	566.987	1330.15	999.41	871.2	1.0115365	8.1E-07	5.1E-06

ENSRNOG00000047620	Golph3l	511.83255	529.603	1133.38	967.35	785.5	1.0120571	2E-07	1.4E-06
ENSRNOG00000019671	Rsbn1	473.65149	559.718	1167.52	917.88	779.7	1.0126462	1.2E-06	7.6E-06
ENSRNOG00000002705	Vps4b	3656.0942	3739.41	8603.28	6319.8	5580	1.0127425	1.3E-08	1.2E-07
ENSRNOG00000024437	Blcap	1082.4846	1155.78	2417.35	2104.2	1690	1.0142956	3.3E-09	3.1E-08
ENSRNOG00000009499	Hinfp	292.03349	251.302	658.548	439.7	410.4	1.0145099	8.3E-05	0.00036
ENSRNOG00000002278	Tec	171.2988	168.227	368.425	317.87	256.5	1.0148678	0.00011	0.00047
ENSRNOG00000002915	Trim11	507.70486	559.718	1172.54	985.67	806.4	1.0154422	2.5E-07	1.7E-06
ENSRNOG00000063476	ENSRNOG0000006347	53.659864	60.2294	119.462	110.84	86.05	1.0156829	0.01137	0.0286
ENSRNOG00000006002	Stk35	734.72736	858.788	1949.54	1273.3	1204	1.0157869	4.3E-06	2.3E-05
ENSRNOG00000055860	Arl8b	6350.4385	6146.51	14390.7	10881	9442	1.0158855	3.1E-09	3E-08
ENSRNOG00000008214	Fbxo9	615.02459	638.639	1426.52	1109.3	947.4	1.0159889	2.8E-07	2E-06
ENSRNOG00000019812	Nr1h2	1949.2977	1893.07	4349.83	3421.4	2903	1.0160167	4.9E-09	4.6E-08
ENSRNOG00000018334	Ctu1	302.35269	315.685	638.47	611.92	467.1	1.0165252	2E-06	1.2E-05
ENSRNOG00000028834	Polr2a	10032.331	10551.6	23276	18381	15560	1.0170335	5.3E-10	5.8E-09
ENSRNOG00000061782	Tspyl2	912.21768	974.054	2138.27	1681	1426	1.0175086	4.7E-08	3.8E-07
ENSRNOG00000019330	Procr	238.37363	298.031	607.35	479.09	405.7	1.0177826	2.9E-05	0.00014
ENSRNOG00000011639	Mrpl47	425.15123	409.144	932.608	757.57	631.1	1.0181529	1E-06	6.5E-06
ENSRNOG00000007083	Gpn2	374.58713	328.146	833.223	590.85	531.7	1.0183581	1.5E-05	7.6E-05
ENSRNOG00000070073	Kti12	358.0764	329.185	772.99	622	520.6	1.0208603	3.5E-06	1.9E-05
ENSRNOG00000031506	Ftl1	34.053375	30.1147	64.2485	65.956	48.59	1.0208892	0.04854	0.09853
ENSRNOG00000023152	Tmem201	468.49189	409.144	948.67	832.69	664.7	1.0210072	5.1E-07	3.4E-06
ENSRNOG00000049531	Usp19	3815.0099	3703.07	8596.25	6666.1	5695	1.0214638	1.8E-09	1.8E-08

ENSRNOG00000015679	Dok4	291.00157	241.956	624.416	458.02	403.8	1.0215149	4E-05	0.00018
ENSRNOG00000015242	Fbxl8	150.66039	130.843	239.928	331.61	213.3	1.0225262	0.00057	0.00201
ENSRNOG00000011827	ENSRNOG0000001182	47.468341	35.3069	103.4	65.04	62.8	1.0232228	0.03575	0.07633
ENSRNOG00000002080	Urb1	689.32286	739.367	1675.48	1230.3	1084	1.0238757	4.2E-07	2.8E-06
ENSRNOG00000020903	Lto1	1044.3035	1063.36	2423.37	1864.2	1599	1.0242704	3.5E-08	2.9E-07
ENSRNOG00000018369	Prx	254.88435	247.148	580.245	441.54	381	1.0246041	2.4E-05	0.00012
ENSRNOG00000028168	Ankrd16	77.394034	73.729	168.652	139.24	114.8	1.0259989	0.0042	0.01204
ENSRNOG00000014511	Alg10	1114.4741	1043.63	2544.84	1851.3	1639	1.0262246	1.3E-07	9.5E-07
ENSRNOG00000033100	AABR07054189.1	298.22501	292.839	670.594	534.06	448.9	1.0267257	6.6E-06	3.5E-05
ENSRNOG00000007875	Actr6	661.46101	722.752	1617.26	1203.7	1051	1.0267845	3.7E-07	2.5E-06
ENSRNOG00000016725	Elp1	2177.3522	2160.99	4253.45	4587.6	3295	1.0271184	3.1E-11	4.1E-10
ENSRNOG00000013865	Zmym6	447.85348	418.49	1049.06	717.27	658.2	1.0271765	7.3E-06	3.9E-05
ENSRNOG00000016330	Rbm12b	43.340659	46.7297	111.431	72.368	68.47	1.0275157	0.02607	0.05856
ENSRNOG00000014097	Psmd7	5511.4871	5531.75	12564.6	9958.4	8392	1.0281838	4E-10	4.5E-09
ENSRNOG00000023140	Pnldc1	62.947148	59.1909	149.579	99.849	92.89	1.0287244	0.01133	0.02852
ENSRNOG00000003215	Zfp287	362.20408	388.375	899.48	632.99	570.8	1.0292207	8.4E-06	4.4E-05
ENSRNOG00000010353	Dnajb6	2882.1538	2991.74	6863.55	5128	4466	1.0295354	6.3E-09	5.8E-08
ENSRNOG00000020191	Tbc1d17	970.00523	938.747	2044.91	1855.9	1452	1.0310294	1.5E-09	1.5E-08
ENSRNOG00000015340	Dnajc25	787.35531	780.905	1725.68	1481.3	1194	1.0318271	9.6E-09	8.6E-08
ENSRNOG00000014440	Josd1	1561.2956	1569.08	3511.58	2890.1	2383	1.0319851	1.3E-09	1.3E-08
ENSRNOG00000049988	Mbtd1	362.20408	359.299	897.472	578.94	549.5	1.0323558	2.4E-05	0.00012
ENSRNOG00000005749	Foxred2	75.330193	122.536	286.107	119.09	150.8	1.0326504	0.01424	0.03469

ENSRNOG00000007029	Dnaja1	6760.1109	6480.89	17895.2	9199.9	10084	1.0329649	1.3E-05	6.7E-05
ENSRNOG00000007224	Bmt2	599.54578	611.639	1407.44	1073.6	923.1	1.034175	2.7E-07	1.9E-06
ENSRNOG00000011513	ENSRNOG0000001151	1439.529	1435.12	3526.64	2362.5	2191	1.0344497	2.9E-07	2E-06
ENSRNOG00000050630	AABR07070043.1	103.19205	91.3825	246.955	152.06	148.4	1.0347542	0.00329	0.00967
ENSRNOG00000001728	Fam43a	252.82051	245.071	566.19	454.36	379.6	1.0349108	1.3E-05	6.8E-05
ENSRNOG00000024022	Ndfip2	1382.7734	1295.97	2945.39	2544.8	2042	1.0351637	8.1E-10	8.6E-09
ENSRNOG00000025554	Zfp445	1193.932	1158.9	2660.29	2168.3	1795	1.0370173	4E-09	3.8E-08
ENSRNOG00000010100	Tyw5	326.08686	331.261	803.107	546.88	501.8	1.0375619	1.7E-05	8.3E-05
ENSRNOG00000025424	Yrdc	601.60963	572.179	1452.62	959.1	896.4	1.0384252	3.3E-06	1.8E-05
ENSRNOG00000027250	Rbm27	1410.6353	1455.89	3529.65	2361.6	2189	1.0390497	2.7E-07	1.9E-06
ENSRNOG00000016591	Utp15	2062.809	1848.42	4443.19	3595.5	2987	1.0392116	1.8E-09	1.8E-08
ENSRNOG00000004515	Pes1	2548.8435	2432.02	5741.21	4499.6	3805	1.0397573	1.4E-09	1.4E-08
ENSRNOG00000001483	Rcc1l	465.39613	506.757	1102.26	896.81	742.8	1.039758	2.9E-07	2E-06
ENSRNOG00000001992	Atp6v1a	3745.8713	3640.76	8653.48	6537.8	5644	1.0401772	2.2E-09	2.2E-08
ENSRNOG00000053196	Slc48a1	4567.2799	4399.86	9042.98	9397.7	6852	1.0401875	1.6E-12	2.5E-11
ENSRNOG00000063314	Utp23	151.69231	119.42	292.13	265.65	207.2	1.0404341	0.00032	0.00119
ENSRNOG00000018367	Taldo1	5798.361	5556.68	12060.7	11299	8679	1.0406969	1.6E-12	2.5E-11
ENSRNOG00000055292	ENSRNOG0000005529	81.521716	99.69	199.773	173.13	138.5	1.0407488	0.00187	0.00586
ENSRNOG00000004526	Cox7a2l	3039.0057	2794.43	6118.67	5883.8	4459	1.0408862	8.9E-12	1.3E-10
ENSRNOG00000060701	AC130232.2	176.4584	223.264	442.713	380.16	305.6	1.0413692	5.2E-05	0.00023
ENSRNOG00000063551	ENSRNOG0000006355	79.457875	107.997	224.87	161.22	143.4	1.0415541	0.00282	0.00843
ENSRNOG00000012716	Chd2	1148.5275	1256.51	2394.26	2556.7	1839	1.0417262	3.4E-10	3.8E-09

ENSRNOG00000065424	ENSRNOG0000006542	233.21402	224.302	586.268	356.34	350	1.0419123	0.00018	0.0007
ENSRNOG00000065226	Ufm1	1273.3898	1198.36	3108.02	1982.3	1891	1.0419742	9.6E-07	6E-06
ENSRNOG00000054347	Gm26224	35.085295	51.9219	101.392	77.864	66.57	1.042063	0.02575	0.05792
ENSRNOG00000052787	Rsl24d1	1238.3045	1166.16	2653.26	2299.3	1839	1.0423101	8.6E-10	9.1E-09
ENSRNOG00000068859	Fam210a	307.5123	341.646	643.489	693.45	496.5	1.0424935	1E-06	6.5E-06
ENSRNOG00000057620	Slc6a8	1845.0738	1746.65	4145.04	3255.6	2748	1.0428339	2.6E-09	2.5E-08
ENSRNOG00000009609	ENSRNOG0000000960	240.43747	241.956	537.078	458.02	369.4	1.0442366	8.5E-06	4.4E-05
ENSRNOG00000025768	Clk1	2526.1413	2655.28	6429.87	4261.5	3968	1.0448952	1.3E-07	9.5E-07
ENSRNOG00000019506	Dnajb2	2829.5259	2797.55	6539.3	5074	4310	1.0452215	1.1E-09	1.1E-08
ENSRNOG00000018069	Ngdn	744.01465	924.209	1960.58	1484	1278	1.0457864	2.6E-07	1.8E-06
ENSRNOG00000019288	Ogfod1	777.0361	827.634	1869.23	1445.5	1230	1.0463545	5E-08	3.9E-07
ENSRNOG00000014805	Wrap73	240.43747	242.994	614.377	384.74	370.6	1.0464807	0.0001	0.00044
ENSRNOG00000000048	Gak	3172.1235	3298.08	7290.2	6078	4960	1.046855	8.3E-11	1E-09
ENSRNOG00000050500	Tob2	1867.776	2022.88	5047.53	3010.1	2987	1.0501805	1.6E-06	9.6E-06
ENSRNOG00000012472	Pyroxd1	557.23705	565.948	1348.22	978.34	862.4	1.0501844	5.7E-07	3.7E-06
ENSRNOG00000001043	Ddx55	536.59864	588.794	1245.82	1085.5	864.2	1.0505322	3.9E-08	3.1E-07
ENSRNOG00000000461	Brd2	6447.439	6475.69	16771.9	9997.8	9923	1.0505863	6.4E-07	4.1E-06
ENSRNOG00000008788	Mpp5	1216.6342	1140.2	2556.89	2325.8	1810	1.0507353	2.9E-10	3.3E-09
ENSRNOG00000023053	ENSRNOG0000002305	1868.8079	2178.64	4522.5	3865.7	3109	1.0512787	5.6E-10	6.1E-09
ENSRNOG00000066932	Pfdn2	1635.5939	1570.12	3911.13	2735.3	2463	1.051748	5.5E-08	4.3E-07
ENSRNOG00000042389	Bcl10	650.10989	639.677	1664.44	1010.4	991.2	1.0518683	6.7E-06	3.5E-05
ENSRNOG00000021119	Pdcd2l	198.12873	246.11	509.973	411.31	341.4	1.0518733	3.1E-05	0.00014

ENSRNOG00000049517	Tnfaip3	433.40659	404.99	1176.55	562.45	644.4	1.051887	0.00025	0.00097
ENSRNOG00000020027	Arhgef2	3367.1564	3537.96	6902.7	7431.9	5310	1.0537994	2.9E-12	4.4E-11
ENSRNOG00000066681	Atp13a3	2841.9089	2867.12	6465.01	5391.9	4391	1.0543285	6.9E-11	8.7E-10
ENSRNOG00000001640	Tomm70	2152.5861	2167.22	4923.05	4054.4	3324	1.0552407	2E-10	2.3E-09
ENSRNOG00000012356	Slc36a1	255.91627	229.495	563.179	446.12	373.7	1.0554929	1.3E-05	6.5E-05
ENSRNOG00000045695	NEWGENE_1559832	2411.5981	2554.56	5891.79	4430.9	3822	1.0555116	3.1E-09	3E-08
ENSRNOG00000001494	Napa	3599.3385	3464.23	8745.83	5936.9	5437	1.0555583	3.5E-08	2.9E-07
ENSRNOG00000017564	Mib2	948.3349	943.939	2126.23	1809.2	1457	1.0562181	1.8E-09	1.8E-08
ENSRNOG00000015447	Calcoco1	2177.3522	2085.18	4798.56	4066.3	3282	1.0562972	9.6E-11	1.2E-09
ENSRNOG00000006418	Samhd1	580.97122	571.14	1416.48	980.17	887.2	1.0562982	1E-06	6.3E-06
ENSRNOG00000013920	Arhgap26	52.627943	58.1525	109.423	120.92	85.28	1.0565003	0.00883	0.02295
ENSRNOG00000009779	Krt8	2672.674	2869.2	5404.91	6121	4267	1.0565024	2E-11	2.7E-10
ENSRNOG00000013456	Ighmbp2	803.86603	741.444	1844.13	1371.3	1190	1.0568042	1E-07	7.8E-07
ENSRNOG00000030126	Clk3	982.38827	996.9	2461.52	1657.1	1524	1.0568974	3.2E-07	2.2E-06
ENSRNOG00000056098	Tfpt	600.5777	586.717	1351.23	1119.4	914.5	1.0569213	3.5E-08	2.9E-07
ENSRNOG00000000306	Smpd2	340.53375	329.185	719.785	674.21	515.9	1.0574424	4.1E-07	2.8E-06
ENSRNOG00000043357	Zfp407	473.65149	516.103	1149.45	912.39	762.9	1.058441	2.3E-07	1.6E-06
ENSRNOG00000045637	Rnf14	1779.0309	2069.61	4028.58	3992.1	2967	1.0593947	6E-11	7.6E-10
ENSRNOG00000018126	Abca1	368.3956	357.222	736.851	775.89	559.6	1.0599892	2E-07	1.5E-06
ENSRNOG00000025890	Opa3	1461.1994	1503.66	3485.48	2698.7	2287	1.0604481	3.1E-09	3E-08
ENSRNOG00000061857	Mgst2	1164.0063	1093.47	2321.98	2389.1	1742	1.0613522	1.1E-10	1.3E-09
ENSRNOG00000068809	ENSRNOG0000006880	107.31973	109.036	252.979	198.78	167	1.0614035	0.00069	0.00239

ENSRNOG00000012742	Irx2	238.37363	224.302	544.105	422.3	357.3	1.0620073	1.5E-05	7.7E-05
ENSRNOG00000016688	Ston1	3966.7022	4010.44	8036.09	8620	6158	1.062133	9.3E-13	1.5E-11
ENSRNOG00000016573	Dgat2	977.22867	1039.48	2406.31	1805.5	1557	1.0622103	2.5E-08	2.1E-07
ENSRNOG00000015347	Trim45	66.042909	75.8059	129.501	166.72	109.5	1.0632557	0.00414	0.01189
ENSRNOG00000000901	Vkorc1l1	888.48351	970.939	2064.99	1822.9	1437	1.0640206	1.2E-09	1.2E-08
ENSRNOG00000021047	Lrrfip2	1457.0717	1648	3728.42	2764.6	2400	1.0641107	1.5E-08	1.3E-07
ENSRNOG00000019196	Xpnpep3	351.88488	323.992	799.091	614.67	522.4	1.0641818	2.2E-06	1.3E-05
ENSRNOG00000015385	Pink1	721.3124	671.869	1513.86	1399.7	1077	1.0642812	2.8E-09	2.7E-08
ENSRNOG00000005165	Smcr8	1103.123	1058.17	2604.07	1916.4	1670	1.064323	2.8E-08	2.4E-07
ENSRNOG00000017291	Sord	3366.1245	3408.15	6731.04	7435.6	5235	1.0643975	2.5E-12	3.8E-11
ENSRNOG00000022247	Nr2c2ap	628.43956	574.256	1365.28	1150.6	929.6	1.0644982	2.7E-08	2.2E-07
ENSRNOG00000061080	Kmt2c	1314.6667	1264.82	2620.14	2775.6	1994	1.064795	4.8E-11	6.1E-10
ENSRNOG00000043077	Zfpm1	814.18524	787.135	1847.15	1504.2	1238	1.0652109	8E-09	7.3E-08
ENSRNOG00000013423	Nup50	3125.6871	2985.51	7414.68	5381.8	4727	1.0661182	4.3E-09	4.1E-08
ENSRNOG00000017607	Faf2	1982.3192	2097.64	4934.09	3611.1	3156	1.0664124	6.7E-09	6.2E-08
ENSRNOG00000046252	Tbl2	1001.9948	1005.21	2184.45	2019.9	1553	1.0666047	2E-10	2.3E-09
ENSRNOG00000010993	Dpm1	828.63213	877.479	2136.26	1439.1	1320	1.0670606	3.9E-07	2.6E-06
ENSRNOG00000067892	ENSRNOG0000006789	1922.4678	2054.03	4606.82	3728.3	3078	1.0675983	3.5E-10	4E-09
ENSRNOG00000006499	Cops5	2094.7985	2084.14	4782.5	3977.5	3235	1.067688	9.6E-11	1.2E-09
ENSRNOG00000027145	Rora	74.298273	70.6137	129.501	174.05	112.1	1.0677324	0.00379	0.01101
ENSRNOG00000016620	Csnk1g1	281.71428	295.955	685.652	525.81	447.3	1.0679004	4.4E-06	2.4E-05
ENSRNOG00000011470	Tsc1	1243.4641	1200.43	2821.92	2303.9	1892	1.0684114	1.1E-09	1.1E-08

ENSRNOG00000003980	Ttc1	1296.0921	1244.05	3084.93	2244.3	1967	1.0688041	2.1E-08	1.8E-07
ENSRNOG00000001719	Psmd2	14054.757	13672.1	33813.8	24385	21481	1.0696896	1.4E-09	1.5E-08
ENSRNOG00000000066	Fbxo28	718.21664	777.789	1775.87	1364.9	1159	1.0697264	4E-08	3.3E-07
ENSRNOG00000022838	Cnksr1	91.84092	111.113	244.948	181.38	157.3	1.069981	0.00113	0.00374
ENSRNOG00000006025	Lamb3	57.787545	44.6528	90.3495	124.58	79.34	1.0700919	0.01321	0.03257
ENSRNOG00000000157	Atg12	2210.3736	2308.45	5581.59	3916.1	3504	1.0714987	1.5E-08	1.3E-07
ENSRNOG00000030869	Aldoart2	90.809	100.728	227.882	174.97	148.6	1.0718115	0.00115	0.00379
ENSRNOG00000006241	Marchf7	3896.5316	4091.44	9392.33	7413.6	6198	1.073004	1.4E-10	1.7E-09
ENSRNOG00000006238	Traf2	661.46101	714.445	1585.13	1312.7	1068	1.0743591	1.1E-08	1E-07
ENSRNOG00000029220	Zmym5	684.16326	700.945	1667.45	1250.4	1076	1.074581	6.5E-08	5.1E-07
ENSRNOG00000013969	Ahi1	862.6855	845.288	1846.14	1754.2	1327	1.0757925	2.8E-10	3.2E-09
ENSRNOG00000011572	Klc1	5697.2328	5988.67	13315.5	11320	9080	1.0759144	5E-12	7.4E-11
ENSRNOG00000063591	Adat3	1323.9539	1179.66	2919.29	2363.4	1947	1.0770807	1.8E-09	1.8E-08
ENSRNOG00000010038	Psmc5	3225.7833	3203.58	7559.24	6006.5	4999	1.0771438	1.1E-10	1.4E-09
ENSRNOG00000015161	ENSRNOG0000001516	113.51125	66.46	196.761	183.21	140	1.0777084	0.00259	0.00783
ENSRNOG00000019023	Tars1	7915.8618	7811.12	16477.7	16730	12234	1.0782719	5.5E-14	1.1E-12
ENSRNOG00000027906	Ankrd11	7866.3296	8524.53	18177.3	16441	12752	1.0786399	6.3E-13	1.1E-11
ENSRNOG00000039017	Naa50	2234.1078	2217.06	5248.3	4158.9	3465	1.0794622	2.4E-10	2.8E-09
ENSRNOG00000048818	Catsperd	51.596023	33.23	68.2641	110.84	65.98	1.0796001	0.02689	0.06012
ENSRNOG00000025764	AC128848.1	210.51177	204.572	523.023	355.43	323.4	1.0807143	5.4E-05	0.00024
ENSRNOG00000055028	Setd1a	3046.2292	3342.73	7982.88	5541.2	4978	1.0817821	1.2E-08	1.1E-07
ENSRNOG00000018920	Slc25a17	7246.1454	6917.03	17683.4	12297	11036	1.0818303	4.6E-09	4.3E-08

ENSRNOG00000017979	Mrto4	1453.9759	1400.85	3374.05	2672.1	2225	1.0824487	8.5E-10	9E-09
ENSRNOG00000026569	ENSRNOG0000002656	328.1507	321.916	756.928	620.17	506.8	1.0825477	7.3E-07	4.6E-06
ENSRNOG00000057056	Rab5a	1590.1894	1632.42	3849.89	2976.2	2512	1.0826832	1.1E-09	1.2E-08
ENSRNOG00000011294	Usp33	2028.7556	2029.11	4862.81	3733.8	3164	1.08292	7.5E-10	8E-09
ENSRNOG00000013474	Casz1	146.5327	149.535	367.421	260.16	230.9	1.0829762	0.00018	0.00073
ENSRNOG00000054437	Rel	170.26688	178.611	449.74	290.39	272.3	1.0840938	0.00016	0.00064
ENSRNOG00000038183	Heatr4	35.085295	34.2684	80.3107	66.872	54.13	1.0848288	0.0286	0.06328
ENSRNOG00000007576	Zfp597	132.08582	110.074	308.192	206.11	189.1	1.0855345	0.00066	0.00231
ENSRNOG00000010495	Ttc17	2080.3516	2229.52	4532.53	4616	3365	1.0859077	1.8E-12	2.9E-11
ENSRNOG00000004438	Cntn1	101.1282	157.842	274.06	275.73	202.2	1.0862772	0.00043	0.00156
ENSRNOG00000030157	Dhx16	754.33385	708.214	1725.68	1380.5	1142	1.0863693	1E-08	9.1E-08
ENSRNOG00000003581	Pou2f1	115.57509	92.4209	256.994	185.04	162.5	1.0865692	0.00096	0.00323
ENSRNOG00000067232	ENSRNOG0000006723	321.95918	317.762	732.835	626.58	499.8	1.0871249	4.6E-07	3.1E-06
ENSRNOG00000069274	ENSRNOG0000006927	3401.2098	3463.19	8034.08	6553.4	5363	1.0874589	2.6E-11	3.4E-10
ENSRNOG00000006248	Trim37	1695.4453	1631.38	3496.53	3581.8	2601	1.0892647	3.3E-12	4.9E-11
ENSRNOG00000016217	Gtpbp4	1100.0272	1008.32	2299.9	2186.6	1649	1.0894105	7.8E-11	9.7E-10
ENSRNOG00000010795	Cnot4	1105.1868	1183.82	2822.92	2053.8	1791	1.0909607	1.5E-08	1.3E-07
ENSRNOG00000061893	Leng1	223.92674	195.226	532.058	361.84	328.3	1.0917817	5E-05	0.00022
ENSRNOG00000011936	Abhd14a	40.244898	34.2684	65.2524	93.437	58.3	1.0918687	0.02603	0.05848
ENSRNOG00000003177	Mat2b	1327.0497	1336.47	3075.9	2603.4	2086	1.0922464	1E-10	1.3E-09
ENSRNOG00000052109	AABR07043389.1	126.92622	109.036	323.251	180.46	184.9	1.0926688	0.00143	0.0046
ENSRNOG00000019990	Rex1bd	300.28885	352.03	741.87	650.4	511.1	1.0935595	5.2E-07	3.4E-06

ENSRNOG00000000609	Ipmk	1476.6782	1547.27	3241.54	3216.2	2370	1.0946059	4.1E-12	6.2E-11
ENSRNOG00000061337	Mindy2	160.97959	220.149	411.592	403.06	298.9	1.0959703	2.8E-05	0.00013
ENSRNOG00000060707	Atg2a	1723.3072	1916.95	4607.83	3175.9	2856	1.0962707	2.3E-08	2E-07
ENSRNOG00000004359	Wars1	3367.1564	3502.65	6906.72	7788.3	5391	1.0970296	1.1E-12	1.7E-11
ENSRNOG00000016479	Plekhg4	122.79853	113.19	267.033	238.17	185.3	1.0977551	0.0002	0.00078
ENSRNOG00000027833	Fnip2	1084.5484	1095.55	2696.43	1970.4	1712	1.0978152	1.1E-08	9.5E-08
ENSRNOG00000062360	ENSRNOG0000006236	66.042909	63.3447	155.602	121.83	101.7	1.0995814	0.00383	0.01111
ENSRNOG00000016636	Lpin3	1377.6138	1605.42	2976.51	3417.8	2344	1.1001183	1.4E-10	1.7E-09
ENSRNOG00000011040	ENSRNOG0000001104	24.766091	36.3453	75.2913	55.879	48.07	1.1010185	0.04072	0.08506
ENSRNOG00000013728	Polg2	40.244898	52.9603	97.3767	102.6	73.29	1.1016673	0.01157	0.02903
ENSRNOG00000021250	ENSRNOG0000002125	189.87336	208.726	465.802	390.24	313.7	1.1023138	1E-05	5.2E-05
ENSRNOG00000013756	Snrnp48	626.37572	682.253	1610.23	1200	1030	1.1023176	5.2E-08	4.1E-07
ENSRNOG00000000169	Spata5l1	299.25693	326.069	778.01	565.2	492.1	1.1024447	2.7E-06	1.5E-05
ENSRNOG00000019426	Ramac	401.41706	374.876	987.821	679.71	611	1.102462	1.9E-06	1.1E-05
ENSRNOG00000067995	Rnf113a2	343.62951	310.493	838.243	567.03	514.8	1.1025616	5.5E-06	3E-05
ENSRNOG00000060129	Zfp655	1860.5526	2010.41	4864.82	3452.6	3047	1.1032951	6.1E-09	5.7E-08
ENSRNOG00000037198	Usp18	114.54317	88.2672	219.851	216.19	159.7	1.1041529	0.00051	0.00183
ENSRNOG00000000968	Rnf6	1961.6808	1977.18	4968.22	3500.2	3102	1.1041659	5.6E-09	5.2E-08
ENSRNOG00000019830	Hsd17b1	36.117216	35.3069	92.3573	61.375	56.29	1.1044412	0.02713	0.0606
ENSRNOG00000029152	Tmem69	608.83307	484.95	1172.54	1179.9	861.5	1.1047697	1.9E-08	1.7E-07
ENSRNOG00000060729	Qtrt2	326.08686	339.569	814.15	618.33	524.5	1.1051653	9.1E-07	5.7E-06
ENSRNOG00000019425	Gabarapl2	1392.0607	1409.16	3478.46	2548.4	2207	1.1051691	3.8E-09	3.6E-08

ENSRNOG00000023760	Plekhm3	491.19414	484.95	1120.33	980.17	769.2	1.105336	1.1E-08	9.6E-08
ENSRNOG00000002342	Aldh3a2	1588.1256	1411.24	3263.63	3190.6	2363	1.1055563	9.4E-12	1.3E-10
ENSRNOG00000018122	Tspan17	547.94976	489.104	1086.2	1147.8	817.8	1.107211	5.5E-09	5.1E-08
ENSRNOG00000005577	Desi1	1839.9142	1756	4275.54	3472.7	2836	1.1073916	7.5E-11	9.4E-10
ENSRNOG00000021259	Prnp	13938.15	13529.8	32211.6	26988	21667	1.1078152	7.6E-13	1.3E-11
ENSRNOG00000065102	ENSRNOG0000006510	61.915227	52.9603	152.59	95.269	90.68	1.1078853	0.0082	0.02149
ENSRNOG00000018593	Txndc9	266.23548	219.11	548.12	498.33	382.9	1.108127	2.6E-06	1.5E-05
ENSRNOG00000001254	Col6a2	76.362114	94.4978	201.781	166.72	134.8	1.1083324	0.00117	0.00384
ENSRNOG00000055908	AABR07065593.1	41.276818	35.3069	99.3845	65.956	60.48	1.108787	0.02275	0.05225
ENSRNOG00000052319	AABR07004881.1	62.947148	99.69	153.594	196.95	128.3	1.1088577	0.00262	0.00789
ENSRNOG00000009820	Bnip3l	1640.7535	1641.77	3508.57	3571.7	2591	1.1090093	1.1E-12	1.8E-11
ENSRNOG00000019390	Klhl40	131.0539	122.536	230.893	316.95	200.4	1.1121331	0.00022	0.00085
ENSRNOG00000017120	Abhd2	2863.5793	2869.2	7006.1	5391	4532	1.1125965	1.1E-10	1.3E-09
ENSRNOG00000003872	NEWGENE_620180	904.99424	996.9	1379.34	2732.6	1503	1.112703	1.5E-05	7.4E-05
ENSRNOG00000012035	Rnf181	1141.304	966.785	2501.68	2060.2	1667	1.1134992	1.3E-09	1.4E-08
ENSRNOG00000003302	Flcn	956.59026	1096.59	2580.98	1862.3	1624	1.1135455	2.1E-08	1.8E-07
ENSRNOG00000063135	Gna13	296.16117	328.146	777.006	574.36	493.9	1.1135622	1.8E-06	1E-05
ENSRNOG00000049269	ENSRNOG0000004926	114.54317	85.1518	220.854	211.61	158	1.1145087	0.00053	0.00189
ENSRNOG00000052134	ENSRNOG0000005213	201.22449	263.763	413.6	592.68	367.8	1.1145332	2.9E-05	0.00014
ENSRNOG00000006513	Borcs6	227.0225	208.726	471.825	471.77	344.8	1.1146334	2.2E-06	1.3E-05
ENSRNOG00000003424	Elac2	988.5798	988.592	2339.05	1942.9	1565	1.1146566	2.8E-10	3.2E-09
ENSRNOG00000064520	Hist3h2a	68.10675	69.5753	163.633	134.66	109	1.1147128	0.00237	0.00725

ENSRNOG0000 0022312	Lrsam1	679.00366	746.636	1435.55	1653.5	1129	1.1157444	1.1E-09	1.1E-08
ENSRNOG0000 0016987	Pstpip2	88.745159	71.6522	186.722	161.22	127.1	1.1166245	0.0013	0.00425
ENSRNOG0000 0062521	Gtpbp6	391.09785	322.954	849.285	699.86	565.8	1.1169684	3.7E-07	2.5E-06
ENSRNOG0000 0046912	Nr1d2	1124.7933	1248.2	2753.65	2394.6	1880	1.1172515	7.3E-11	9.1E-10
ENSRNOG0000 0047656	Gaa	6001.6494	5553.56	12900.9	12167	9156	1.1172792	5.1E-14	9.7E-13
ENSRNOG0000 0065892	ENSRNOG0 000006589	494.2899	565.948	1311.07	990.25	840.4	1.1177273	9.3E-08	7.1E-07
ENSRNOG0000 0011844	Ints12	814.18524	840.096	1899.35	1691	1311	1.117793	1.5E-10	1.8E-09
ENSRNOG0000 0019819	Dus2	333.31031	334.377	778.01	671.46	529.3	1.1179757	1.3E-07	9.8E-07
ENSRNOG0000 0066042	Abcb1b	342.59759	365.53	750.905	785.97	561.3	1.1180373	4.7E-08	3.7E-07
ENSRNOG0000 0050343	Jmy	1127.8891	1154.74	2659.29	2299.3	1810	1.1190936	4.2E-11	5.4E-10
ENSRNOG0000 0047198	Tatdn1	269.33124	277.263	589.28	599.1	433.7	1.1205007	2.9E-07	2E-06
ENSRNOG0000 0008618	Tex10	1013.3459	1168.24	2932.34	1818.4	1733	1.122507	4.1E-07	2.7E-06
ENSRNOG0000 0017730	Psmd1	8595.8974	8250.38	20099.8	16588	13383	1.1228331	1.1E-12	1.8E-11
ENSRNOG0000 0006378	Mga	1520.0188	1536.89	3496.53	3165.9	2430	1.1238923	2.5E-12	3.8E-11
ENSRNOG0000 0038106	ENSRNOG0 000003810	48.500261	31.1531	78.3029	95.269	63.31	1.1242055	0.01867	0.04397
ENSRNOG0000 0025679	Stk40	613.99267	690.561	1704.59	1140.5	1037	1.1245389	2.9E-07	2E-06
ENSRNOG0000 0071137	ENSRNOG0 000007113	40.244898	62.3062	128.497	95.269	81.58	1.1248696	0.00994	0.0254
ENSRNOG0000 0024964	Nufip2	1197.0277	1247.16	2958.44	2373.5	1944	1.12513	2.1E-10	2.4E-09
ENSRNOG0000 0025724	Mtf1	620.18419	613.716	1556.02	1138.6	982.1	1.12651	3.8E-08	3.1E-07
ENSRNOG0000 0008081	Ddx27	1856.4249	1866.07	4689.14	3443.4	2964	1.1272922	7.9E-10	8.4E-09
ENSRNOG0000 0014856	Etnk1	1445.7206	1688.5	4001.48	2848	2496	1.1277291	8.1E-09	7.3E-08
ENSRNOG0000 0008614	Zfyve1	659.39717	679.138	1757.8	1168.9	1066	1.1282217	2E-07	1.5E-06

ENSRNOG00000009282	Nub1	1633.5301	1662.54	4033.6	3172.3	2625	1.1282861	1.1E-10	1.3E-09
ENSRNOG00000002755	Pafah1b1	6020.2239	6362.5	14894.6	12176	9863	1.1283693	2.1E-12	3.2E-11
ENSRNOG00000020463	Eif1ad	1205.2831	1332.31	2961.46	2597.9	2024	1.1313616	2.3E-11	3.1E-10
ENSRNOG00000042603	Ttc39b	641.85452	635.524	1379.34	1419.9	1019	1.1318777	1.9E-10	2.3E-09
ENSRNOG00000019140	Banp	331.24647	292.839	870.367	498.33	498.2	1.1322222	2.7E-05	0.00013
ENSRNOG00000028521	Plekhm1	527.31135	516.103	1398.41	893.15	833.7	1.1345241	8.6E-07	5.4E-06
ENSRNOG00000019202	Pvr	4037.9047	4035.37	11176.2	6549.8	6450	1.1345572	1.4E-07	1E-06
ENSRNOG00000033338	Gimap6	123.83045	105.921	319.235	185.96	183.7	1.1354403	0.00078	0.00267
ENSRNOG00000016578	Mtpap	592.32234	696.791	1723.67	1109.3	1031	1.1355791	6E-07	3.9E-06
ENSRNOG00000025076	Ecpas	6586.7483	6377.04	14267.2	14222	10363	1.1359084	3.5E-15	7.7E-14
ENSRNOG00000012147	Nop14	2134.0115	2005.22	5044.51	4053.5	3309	1.136076	2.5E-11	3.3E-10
ENSRNOG00000014456	Coq10b	1951.3616	1827.65	5075.64	3235.5	3023	1.1368657	4.1E-08	3.3E-07
ENSRNOG00000027860	Ggnbp2	2071.0644	2202.53	5547.46	3852.9	3418	1.1371335	3E-09	2.9E-08
ENSRNOG00000029127	Parp16	576.84353	558.679	1382.35	1115.7	908.4	1.1371602	5.5E-09	5.1E-08
ENSRNOG00000004305	Abl2	1071.1334	1158.9	2852.03	2055.6	1784	1.1377447	5.1E-09	4.8E-08
ENSRNOG00000003070	Pigl	305.44845	258.571	610.361	631.16	451.4	1.1383099	2.8E-07	2E-06
ENSRNOG00000001701	Cbr3	220.83098	197.303	420.627	500.16	334.7	1.1392982	3.4E-06	1.9E-05
ENSRNOG00000016346	Prkcd	825.53636	923.171	2191.48	1663.5	1401	1.1402122	3.7E-09	3.6E-08
ENSRNOG00000013078	Zcchc7	658.36525	777.789	1723.67	1442.8	1151	1.1404752	2.1E-09	2.1E-08
ENSRNOG00000021289	Rbbp5	1975.0958	2011.45	5171	3619.3	3194	1.1406285	2.1E-09	2E-08
ENSRNOG00000042308	ENSRNOG0000004230	111.44741	57.114	208.808	163.06	135.1	1.1406369	0.00296	0.00881
ENSRNOG00000009358	Osgin2	176.4584	137.074	399.546	292.22	251.3	1.1408257	7.2E-05	0.00031

ENSRNOG00000043201	Coq8a	56.755625	56.0756	88.3418	160.31	90.37	1.1416986	0.00812	0.02134
ENSRNOG00000014084	Sp1	4362.9597	4642.85	11336.9	8539.4	7221	1.142059	5.1E-11	6.5E-10
ENSRNOG00000003861	Gorab	752.27001	793.366	2080.05	1333.8	1240	1.142835	2E-07	1.4E-06
ENSRNOG00000006806	Setmar	27.861852	32.1916	60.233	72.368	48.16	1.1435669	0.02933	0.06461
ENSRNOG00000016714	Nrap	34.053375	19.7303	72.2796	46.719	43.2	1.1439151	0.04906	0.09945
ENSRNOG00000036758	Iqck	29.925693	32.1916	72.2796	65.04	49.86	1.1440916	0.02586	0.05814
ENSRNOG00000007128	Nop56	3288.7305	3357.27	7946.74	6752.2	5336	1.1450971	4.4E-13	7.5E-12
ENSRNOG00000021379	Txnl4b	463.33228	499.488	1179.56	953.61	774	1.1473462	1.3E-08	1.1E-07
ENSRNOG00000019433	Rab3a	343.62951	292.839	757.932	653.14	511.9	1.1482828	1.6E-07	1.2E-06
ENSRNOG00000003951	Psmc1	6682.7169	7009.45	16557.1	13798	11012	1.1485332	3E-13	5.2E-12
ENSRNOG00000053015	Ccdc189	35.085295	43.6144	79.3068	95.269	63.32	1.1502255	0.01368	0.03351
ENSRNOG00000003643	ENSRNOG0000000364	248.69283	305.301	711.753	518.48	446.1	1.1504736	2.8E-06	1.6E-05
ENSRNOG00000009640	Psmf1	1457.0717	1285.59	3442.32	2649.2	2209	1.1510495	4.1E-10	4.5E-09
ENSRNOG00000059268	Ppp1r10	2516.854	2528.59	6563.39	4647.1	4064	1.151681	6.2E-10	6.7E-09
ENSRNOG00000025140	Zfat	215.67138	183.803	498.93	389.32	321.9	1.1522211	7E-06	3.7E-05
ENSRNOG00000007253	Cbll1	891.57927	1013.51	2581.99	1653.5	1535	1.1523779	1.2E-07	9.2E-07
ENSRNOG00000019332	Gtpbp2	925.63265	964.708	2250.71	1953	1524	1.1528775	2.9E-11	3.9E-10
ENSRNOG00000010244	Sarm1	68.10675	78.9212	155.602	171.3	118.5	1.15318	0.00109	0.00362
ENSRNOG00000058269	Snord83b	34.053375	47.7681	102.396	79.696	65.98	1.153398	0.01341	0.03294
ENSRNOG00000012001	Vps37a	656.30141	711.329	1812.01	1231.2	1103	1.1535445	7E-08	5.4E-07
ENSRNOG00000017784	Lrif1	274.49084	288.686	646.501	607.34	454.3	1.1545679	1E-07	7.7E-07
ENSRNOG00000008102	Dph7	433.40659	494.296	1209.68	856.51	748.5	1.154807	1.9E-07	1.4E-06

ENSRNOG00000050742	Dnmbp	498.41758	536.872	1287.98	1018.6	835.5	1.1554292	9.5E-09	8.5E-08
ENSRNOG00000005690	Lmcd1	1164.0063	1225.36	3070.88	2255.3	1929	1.1562716	1.1E-09	1.2E-08
ENSRNOG00000019544	Zbtb2	496.35374	417.452	1246.82	791.47	738	1.1568562	1.5E-06	8.9E-06
ENSRNOG00000029022	Zfp112	45.4045	49.845	114.443	98.017	76.93	1.1568832	0.00644	0.01748
ENSRNOG00000065794	Mafk	2748.0042	2808.97	8084.27	4311.8	4488	1.1573987	7.7E-07	4.9E-06
ENSRNOG00000019579	Edc3	751.23809	680.176	1926.45	1267.8	1156	1.1576649	1.2E-07	8.6E-07
ENSRNOG00000031855	Actr3b	67.07483	91.3825	176.684	176.8	128	1.1576871	0.00091	0.00306
ENSRNOG00000005528	Tbk1	1460.1674	1453.81	3907.11	2597.9	2355	1.158363	1E-08	8.9E-08
ENSRNOG00000064944	ENSRNOG0000006494	42.308739	55.0372	141.548	76.032	78.73	1.1586453	0.01217	0.03037
ENSRNOG00000030537	Slc26a11	240.43747	215.995	574.221	445.2	369	1.1586705	2.3E-06	1.3E-05
ENSRNOG00000000221	Abhd5	1695.4453	1719.65	3945.26	3686.2	2762	1.1599828	1.3E-13	2.4E-12
ENSRNOG00000028594	Ifnar1	3882.0848	3552.49	8191.69	8424	6013	1.1602274	1.2E-14	2.5E-13
ENSRNOG00000070241	ENSRNOG0000007024	70.170591	83.075	166.645	175.88	123.9	1.1606384	0.00081	0.00277
ENSRNOG00000069589	ENSRNOG0000006958	1486.9974	1508.85	3774.6	2926.8	2424	1.1613364	6.5E-11	8.2E-10
ENSRNOG00000004947	Zfp871	1481.8378	1563.89	4055.69	2759.1	2465	1.1617078	4.7E-09	4.4E-08
ENSRNOG00000002848	Maoa	3604.4981	3416.46	6714.98	8993.8	5682	1.1619055	4.3E-11	5.5E-10
ENSRNOG00000070210	ENSRNOG0000007021	23.73417	28.0378	69.268	46.719	41.94	1.1623291	0.04132	0.08615
ENSRNOG00000015989	Mrpl32	479.84301	450.682	1124.35	959.1	753.5	1.1625766	3.8E-09	3.6E-08
ENSRNOG00000002844	ENSRNOG0000000284	98.032443	93.4593	229.889	199.7	155.3	1.1651873	0.00023	0.0009
ENSRNOG00000002478	Insig2	454.045	455.874	1106.28	936.2	738.1	1.16623	3.8E-09	3.6E-08
ENSRNOG00000018936	ENSRNOG0000001893	232.1821	266.878	597.311	523.06	404.9	1.1664364	4.3E-07	2.9E-06
ENSRNOG00000037376	Mterf4	284.81005	247.148	676.618	518.48	431.8	1.1671673	9.6E-07	6E-06

ENSRNOG00000042826	Zfp52	142.40502	146.42	409.584	240	234.6	1.168207	0.00019	0.00074
ENSRNOG00000047960	Rab9b	274.49084	245.071	525.031	643.07	421.9	1.1691987	4.7E-07	3.1E-06
ENSRNOG00000054493	Rpph1	47.468341	29.0762	130.505	42.138	62.3	1.1707599	0.04216	0.08766
ENSRNOG00000070643	ENSRNOG0000007064	854.43014	812.058	2331.02	1421.7	1355	1.170784	2.2E-07	1.6E-06
ENSRNOG00000016187	Tmem67	238.37363	245.071	582.252	506.58	393.1	1.171027	3.4E-07	2.3E-06
ENSRNOG00000001657	Cldnd1	1322.922	1367.62	3607.96	2456.8	2189	1.1723558	4.1E-09	3.9E-08
ENSRNOG00000021081	Vps72	1120.6656	1103.86	2875.12	2139.9	1810	1.1725342	4.8E-10	5.3E-09
ENSRNOG00000068913	Mpzl3	402.44898	388.375	1024.97	760.32	644	1.1742426	7.8E-08	5.9E-07
ENSRNOG00000064917	Tsr2	1141.304	1126.7	2865.08	2257.1	1848	1.1751524	6.6E-11	8.4E-10
ENSRNOG00000043267	Mrm2	128.99006	119.42	300.161	261.07	202.4	1.1754214	4.2E-05	0.00019
ENSRNOG00000022619	Fth1	30764.645	31641.2	71623.1	69338	50842	1.1755391	9.2E-17	2.4E-15
ENSRNOG00000009513	Akr1b1	10924.942	10077	23663.5	23785	17113	1.1758472	5.3E-16	1.3E-14
ENSRNOG00000003253	Qdpr	1087.6442	917.978	2549.86	1983.2	1635	1.1762262	7.2E-10	7.8E-09
ENSRNOG00000013234	Ccdc115	848.23861	855.672	2382.22	1469.3	1389	1.176263	1.4E-07	1E-06
ENSRNOG00000012270	Med26	219.79906	206.649	618.392	346.27	347.8	1.1766904	5.4E-05	0.00024
ENSRNOG00000016596	Hspa4	10779.441	10775.9	28529.4	20225	17577	1.1774465	5.8E-11	7.4E-10
ENSRNOG00000001081	Ogfod2	133.11774	110.074	325.258	225.35	198.4	1.1779004	0.00015	0.00062
ENSRNOG00000003699	Endov	59.851386	37.3837	119.462	100.77	79.37	1.1786479	0.00705	0.01891
ENSRNOG00000007416	Clp1	425.15123	390.452	1182.57	665.05	665.8	1.1791121	5.7E-06	3.1E-05
ENSRNOG00000009104	AABR07029605.1	1583.9979	1562.85	4212.3	2916.7	2569	1.1796118	1.3E-09	1.3E-08
ENSRNOG00000033776	ENSRNOG0000003377	78.425955	52.9603	192.746	105.35	107.4	1.180228	0.00497	0.01397
ENSRNOG00000003985	Tmem19	368.3956	305.301	757.932	769.48	550.3	1.1808932	2.6E-08	2.2E-07

ENSRNOG00000023440	Phex	31.989534	31.1531	77.299	65.956	51.6	1.1812645	0.01982	0.04628
ENSRNOG00000022941	AABR07004746.1	42.308739	22.8456	100.388	47.635	53.29	1.1815184	0.03724	0.07904
ENSRNOG00000065578	ENSRNOG0000006557	34.053375	58.1525	143.555	65.956	75.43	1.1823449	0.01858	0.04381
ENSRNOG00000025179	ENSRNOG0000002517	54.691784	40.499	126.489	89.773	77.86	1.1825872	0.00716	0.01918
ENSRNOG00000066988	Tbpl1	91.84092	101.767	277.072	163.06	158.4	1.1834951	0.00074	0.00256
ENSRNOG00000028155	Dnaaf2	602.64155	474.566	1414.47	1033.3	881.2	1.1837748	7E-08	5.4E-07
ENSRNOG00000029956	Zfp654	633.59916	555.564	1547.99	1156.1	973.3	1.184805	8.9E-09	8E-08
ENSRNOG00000028752	Tmem81	35.085295	28.0378	93.3612	50.383	51.72	1.1851311	0.02842	0.06298
ENSRNOG00000017222	Abraxas2	1238.3045	1347.89	3165.24	2716.1	2117	1.1852011	2.8E-12	4.3E-11
ENSRNOG00000016873	Msrb2	16.510727	45.6912	89.3456	52.215	50.94	1.1853011	0.04524	0.09298
ENSRNOG00000003821	Rptor	1458.1036	1390.47	3505.56	2973.5	2332	1.1854194	1.6E-12	2.5E-11
ENSRNOG00000014254	Cpt1a	2046.2983	1975.11	4729.3	4417.2	3292	1.1854725	1.9E-14	3.9E-13
ENSRNOG00000007333	Wdr20	613.99267	611.639	1656.41	1132.2	1004	1.1856419	3.2E-08	2.7E-07
ENSRNOG00000007582	Zswim4	798.70643	714.445	2354.11	1089.2	1239	1.1858482	1.7E-05	8.5E-05
ENSRNOG00000009718	Pex12	189.87336	184.842	497.926	355.43	307	1.1865712	8E-06	4.2E-05
ENSRNOG00000001064	Sbno1	2527.1732	2818.32	6906.72	5264.5	4379	1.186996	2.1E-11	2.9E-10
ENSRNOG00000029197	Diablo	1492.157	1483.93	3860.94	2917.6	2439	1.1873953	6E-11	7.7E-10
ENSRNOG00000058842	Sptbn2	22.70225	25.9609	52.2019	58.627	39.87	1.1879714	0.03744	0.07936
ENSRNOG00000031540	ENSRNOG0000003154	101.1282	99.69	260.006	197.87	164.7	1.188223	0.00018	0.00074
ENSRNOG00000008564	Tmem222	548.98168	537.91	1484.74	993	891.2	1.1883714	8E-08	6.1E-07
ENSRNOG00000012016	Npc1	1932.787	1935.65	4738.33	4078.3	3171	1.188383	1.8E-13	3.2E-12
ENSRNOG00000006697	Pip4p2	1760.4563	1800.65	3846.88	4273.4	2920	1.1892704	6.2E-14	1.2E-12

ENSRNOG00000017889	Serac1	244.56515	218.072	578.237	477.26	379.5	1.1894884	5.9E-07	3.8E-06
ENSRNOG00000065656	ENSRNOG0000006565	20.638409	26.9994	59.2291	49.467	39.08	1.1895675	0.04065	0.08496
ENSRNOG00000021548	Rassf1	1221.7938	1074.78	3055.82	2183.9	1884	1.189763	1.7E-09	1.7E-08
ENSRNOG00000025459	Mars1	3772.7012	3667.76	7704.81	9268.6	6103	1.1898661	1E-13	1.9E-12
ENSRNOG00000043225	Zfp771	430.31083	391.491	1132.38	743.83	674.5	1.1903998	4.2E-07	2.8E-06
ENSRNOG00000069157	ENSRNOG0000006915	28.893773	24.9225	77.299	45.802	44.23	1.1918072	0.03534	0.07556
ENSRNOG00000016137	Rad23b	8919.9204	8796.6	21464	19010	14548	1.1918781	1.3E-15	2.9E-14
ENSRNOG00000001877	Med15	3101.9529	3307.42	7851.37	6791.6	5263	1.191903	3.9E-14	7.5E-13
ENSRNOG00000017858	Tsr3	435.47043	479.758	1150.45	944.45	752.5	1.1943868	3.5E-09	3.3E-08
ENSRNOG00000010756	Tp53	2904.8561	2725.9	6676.83	6220.9	4632	1.1956802	4.6E-15	9.8E-14
ENSRNOG00000000517	Kctd20	1428.1779	1518.2	4187.2	2564.9	2425	1.1962059	3.2E-08	2.6E-07
ENSRNOG00000013390	Kbtbd8	353.94872	419.529	1034	739.25	636.7	1.1965191	1.7E-07	1.3E-06
ENSRNOG00000000308	Zbtb24	296.16117	271.032	759.94	541.39	467.1	1.1974308	6.4E-07	4.1E-06
ENSRNOG00000063043	Cbx4	865.78126	725.868	2016.8	1639.7	1312	1.1997159	3.4E-10	3.8E-09
ENSRNOG00000048495	Rrp12	1782.1266	1737.31	4221.33	3863	2901	1.1997202	2.5E-14	5E-13
ENSRNOG00000064376	RGD1562660	75.330193	62.3062	170.66	145.65	113.5	1.1998543	0.00096	0.00323
ENSRNOG00000019298	Dctn4	2369.2894	2348.94	5891.79	4949.4	3890	1.2001244	1.3E-13	2.3E-12
ENSRNOG00000054957	Sfrp4	43.340659	33.23	72.2796	103.51	63.09	1.2001306	0.01224	0.03053
ENSRNOG00000017714	Usp3	783.22763	742.483	1990.7	1517.9	1259	1.2011259	5.1E-10	5.6E-09
ENSRNOG00000065992	Map2k3	2338.3318	2287.68	6335.51	4301.8	3816	1.2011607	5.7E-10	6.2E-09
ENSRNOG00000019834	Hsp90ab1	41502.809	40412.9	119141	69415	67618	1.2027696	1.5E-08	1.3E-07
ENSRNOG00000011839	Mrps31	448.8854	420.567	1036.01	965.52	717.7	1.2027726	5.5E-10	6E-09

ENSRNOG00000023533	Dipk1a	338.46991	361.376	805.115	806.12	577.8	1.2030842	3E-09	2.9E-08
ENSRNOG00000002750	Rc3h1	878.16431	911.748	2611.1	1515.1	1479	1.204627	2.2E-07	1.6E-06
ENSRNOG00000003247	Sde2	1458.1036	1551.43	4194.23	2746.3	2488	1.2053245	4E-09	3.8E-08
ENSRNOG00000005021	Orc4	1222.8257	1263.78	3185.32	2550.3	2056	1.2056065	8.1E-12	1.2E-10
ENSRNOG00000028556	Usp30	648.04605	586.717	1692.55	1156.1	1021	1.2056209	2.4E-08	2E-07
ENSRNOG00000003397	Adprm	292.03349	261.686	800.095	479.09	458.2	1.2072239	6.1E-06	3.2E-05
ENSRNOG00000018719	Cir1	1052.5589	1101.78	2974.51	2002.5	1783	1.2077816	3.6E-09	3.4E-08
ENSRNOG00000001436	Ywhag	9749.5845	9229.63	25687.4	18155	15705	1.2078794	2.7E-11	3.6E-10
ENSRNOG00000001629	Get1	640.8226	560.756	1351.23	1424.5	994.3	1.2079576	5.6E-11	7.1E-10
ENSRNOG00000011387	Tet3	993.7394	1094.51	2580.98	2244.3	1728	1.2082089	2.7E-12	4.1E-11
ENSRNOG00000063308	Prkrip1	695.51439	739.367	1896.34	1419.9	1188	1.208315	9.2E-10	9.7E-09
ENSRNOG00000000565	Sgpl1	4188.5651	4494.36	11967.3	8114.4	7191	1.2095593	2.4E-10	2.8E-09
ENSRNOG00000013961	Ghitm	7444.2742	7199.48	17327	16548	12130	1.2099061	6.2E-17	1.7E-15
ENSRNOG00000004239	ENSRNOG0000000423	87.713239	103.844	270.045	174.05	158.9	1.2119933	0.00039	0.00144
ENSRNOG00000024595	Cers6	1959.6169	2010.41	4999.34	4200.1	3292	1.2123021	1.4E-13	2.5E-12
ENSRNOG00000064275	Grpel2	505.64102	530.641	1407.44	994.83	859.6	1.2125727	1.6E-08	1.4E-07
ENSRNOG00000060817	Stam	1782.1266	1791.3	4876.87	3407.7	2965	1.2129617	2.3E-10	2.7E-09
ENSRNOG00000002241	Hspbap1	285.84197	337.492	844.266	601.84	517.4	1.2135863	3.6E-07	2.4E-06
ENSRNOG00000066471	ENSRNOG0000006647	47.468341	44.6528	109.423	104.43	76.49	1.2148072	0.00411	0.01183
ENSRNOG00000030862	Atp6v1h	2263.0016	2141.26	5990.17	4234	3657	1.2148806	1.1E-10	1.3E-09
ENSRNOG00000010712	Terf2ip	765.68498	814.135	2006.76	1662.6	1312	1.2155845	2.5E-11	3.4E-10
ENSRNOG00000070392	Spty2d1	1061.8461	1175.51	3126.09	2071.2	1859	1.2157308	5.2E-09	4.8E-08

ENSRNOG00000026519	ENSRNOG0000002651	541.75824	478.72	1299.03	1071.8	847.8	1.2158227	9.2E-10	9.7E-09
ENSRNOG00000014678	Fzd5	293.06541	384.222	878.398	695.28	562.7	1.2159924	1.3E-07	9.9E-07
ENSRNOG00000020778	Chrnb2	36.117216	28.0378	93.3612	55.879	53.35	1.2161382	0.02073	0.04815
ENSRNOG00000013945	Itpkc	529.37519	527.526	1443.58	1015	878.9	1.2175581	1.3E-08	1.2E-07
ENSRNOG00000049920	Psma2	3584.8917	3478.76	9271.87	7158	5873	1.2177619	9E-13	1.5E-11
ENSRNOG00000070277	ENSRNOG0000007027	119.70277	128.766	281.087	296.8	206.6	1.2179239	1.6E-05	7.7E-05
ENSRNOG00000005031	Surf6	1105.1868	1094.51	2983.54	2136.2	1830	1.2185412	3.4E-10	3.8E-09
ENSRNOG00000010540	Mrpl45	1215.6023	1146.43	2879.14	2618.1	1965	1.2185733	1.5E-13	2.8E-12
ENSRNOG00000004085	Tcaim	205.35217	192.111	449.74	475.43	330.7	1.2190165	3.5E-07	2.4E-06
ENSRNOG00000014093	Tom1	1885.3187	2110.1	4879.88	4429.1	3326	1.2202311	2.9E-14	5.7E-13
ENSRNOG00000017194	Prdx1	11687.531	10571.3	26536.7	25335	18533	1.2205669	1.1E-16	3E-15
ENSRNOG00000019590	Smg5	3421.8482	3299.11	8612.32	7050.8	5596	1.2205686	6.2E-14	1.2E-12
ENSRNOG00000002625	Ptpn4	712.02511	658.369	1909.39	1286.1	1141	1.2210906	1.3E-08	1.1E-07
ENSRNOG00000067071	ENSRNOG0000006707	230.11826	250.263	631.443	489.17	400.2	1.2215178	3.5E-07	2.3E-06
ENSRNOG00000015589	Smyd5	563.42857	496.373	1391.38	1086.4	884.4	1.2249217	1.7E-09	1.7E-08
ENSRNOG00000010227	Gpatch2l	449.91732	428.875	1100.26	954.52	733.4	1.2251328	4.8E-10	5.3E-09
ENSRNOG00000055222	AC094212.1	37.149136	29.0762	73.2835	81.528	55.26	1.2253268	0.01333	0.03281
ENSRNOG00000053438	Snora7a	36.117216	23.8841	76.2952	64.123	50.1	1.2258095	0.0199	0.04645
ENSRNOG00000016551	Bcl2l11	302.35269	300.108	786.041	623.83	503.1	1.226166	3.6E-08	2.9E-07
ENSRNOG00000003674	Pir	904.99424	804.789	1874.25	2127.1	1428	1.226777	4.3E-12	6.5E-11
ENSRNOG00000004377	Lpin1	222.89482	206.649	489.895	515.74	358.8	1.2273249	1.5E-07	1.1E-06
ENSRNOG00000022305	Abcc1	2027.7237	1910.72	4343.8	4888	3293	1.2290532	1.1E-14	2.4E-13

ENSRNOG00000066088	Ubb	12340.737	10372.9	33092	20159	18991	1.2292075	5.7E-09	5.3E-08
ENSRNOG00000004870	Dyrk3	43.340659	35.3069	98.3806	86.109	65.78	1.2294582	0.00723	0.01933
ENSRNOG00000037428	Zfp605	26.829932	43.6144	99.3845	65.956	58.95	1.229774	0.01553	0.03737
ENSRNOG00000026880	Usp38	1578.8383	1495.35	4395	2816.9	2572	1.2299739	3.2E-09	3.1E-08
ENSRNOG00000017489	Gse1	541.75824	554.525	1267.9	1305.4	917.4	1.2310376	1.3E-11	1.8E-10
ENSRNOG00000011550	Kcnab2	102.16013	93.4593	210.816	248.25	163.7	1.2311208	8.1E-05	0.00035
ENSRNOG00000025811	Cfp	97.000523	120.459	332.285	179.55	182.3	1.2336898	0.00045	0.00165
ENSRNOG00000003809	ENSRNOG0000000380	2321.821	2407.1	6934.83	4192.8	3964	1.2344312	7.3E-09	6.6E-08
ENSRNOG00000027955	Zfp133	301.32077	291.801	767.971	628.41	497.4	1.234875	2.1E-08	1.8E-07
ENSRNOG00000037113	Slfn2	1094.8676	1146.43	3306.79	1969.5	1879	1.2349382	3.6E-08	3E-07
ENSRNOG00000002203	Lin54	1144.3998	1165.13	3234.51	2204.9	1937	1.2356336	7.8E-10	8.4E-09
ENSRNOG00000000804	Mrps18b	700.67399	727.944	1824.06	1540.8	1198	1.2357252	1.1E-11	1.6E-10
ENSRNOG00000066186	ENSRNOG0000006618	31.989534	26.9994	93.3612	45.802	49.54	1.2359523	0.02718	0.06068
ENSRNOG00000025079	Fam126b	278.61852	286.609	826.196	506.58	474.5	1.2368067	2E-06	1.2E-05
ENSRNOG00000009068	Phlda3	3897.5636	3731.1	8534.01	9448.1	6403	1.2370926	3.2E-16	7.8E-15
ENSRNOG00000015977	Zfp609	1577.8064	1798.57	4135	3828.2	2835	1.2378305	2.8E-14	5.5E-13
ENSRNOG00000016767	Ggps1	351.88488	343.723	951.682	690.7	584.5	1.238919	4.1E-08	3.3E-07
ENSRNOG00000049497	Eif6	2441.5238	2453.83	6752.12	4823.9	4118	1.2415456	1.8E-11	2.5E-10
ENSRNOG00000000246	Amz2	720.28048	708.214	1689.54	1691.9	1202	1.2431571	5.9E-13	9.9E-12
ENSRNOG00000024632	Atf6	5155.4746	5022.92	12280.5	11818	8569	1.2434198	1.4E-17	4.1E-16
ENSRNOG00000048088	Mest	621.21611	736.252	1661.43	1554.5	1143	1.2442846	1.2E-11	1.7E-10
ENSRNOG00000008338	Bsdc1	950.39874	1025.98	2761.68	1924.6	1666	1.2453506	6E-10	6.5E-09

ENSRNOG00000032395	Tfcp2	742.98273	771.559	1898.34	1692.9	1276	1.2454536	1.3E-12	2.1E-11
ENSRNOG00000015336	Isl2	43.340659	44.6528	137.532	71.452	74.24	1.2460147	0.0083	0.02172
ENSRNOG00000059474	Mob1a	3582.8278	3595.07	9302.99	7747.9	6057	1.2481621	5.2E-15	1.1E-13
ENSRNOG00000039567	Trmt10c	802.83411	767.405	2175.42	1555.5	1325	1.2482235	5.4E-10	5.9E-09
ENSRNOG00000001699	Setd4	780.13186	738.329	2008.77	1601.3	1282	1.2491481	2.5E-11	3.3E-10
ENSRNOG00000047499	Rbm15	370.45944	380.068	1188.6	596.35	633.9	1.2492677	8.1E-06	4.2E-05
ENSRNOG00000065378	ENSRNOG0000006537	35.085295	31.1531	62.2408	95.269	55.94	1.2511264	0.01334	0.03283
ENSRNOG00000070636	ENSRNOG0000007063	40.244898	44.6528	112.435	89.773	71.78	1.2512849	0.00464	0.01314
ENSRNOG00000022288	Pafah2	115.57509	118.382	295.142	261.99	197.8	1.2514233	1.4E-05	7E-05
ENSRNOG00000011398	Trmt61a	596.45002	505.719	1456.64	1169.8	932.1	1.2524426	4.5E-10	5E-09
ENSRNOG00000050317	Uhrf1bp1l	4049.2559	4181.79	10749.6	8873.8	6964	1.2533759	5.2E-15	1.1E-13
ENSRNOG00000019009	Arrdc2	157.88383	153.689	422.635	320.62	263.7	1.2535726	3.9E-06	2.2E-05
ENSRNOG00000019582	Mthfd1l	1754.2648	1654.23	3281.7	4848.6	2885	1.2543392	2.2E-10	2.5E-09
ENSRNOG00000061862	Zbtb10	127.95814	183.803	413.6	330.69	264	1.2550481	1.1E-05	5.4E-05
ENSRNOG00000039656	Abca17	24.766091	19.7303	52.2019	54.047	37.69	1.255691	0.03278	0.07089
ENSRNOG00000030683	Vps26a	2971.9309	2996.93	8143.5	6115.5	5057	1.2562647	7.9E-13	1.3E-11
ENSRNOG00000002695	Tfb2m	735.75928	781.943	2026.84	1606.8	1288	1.2592745	2.1E-11	2.8E-10
ENSRNOG00000020730	Nbr1	3725.2328	3949.18	10746.6	7625.2	6512	1.2592926	7.1E-12	1E-10
ENSRNOG00000010752	Tmem41b	1183.6128	1197.32	3295.75	2404.6	2020	1.2593293	3.1E-11	4.1E-10
ENSRNOG00000042213	Zscan25	612.96075	663.561	1743.75	1315.4	1084	1.2606359	2.6E-10	3E-09
ENSRNOG00000052894	Epg5	1122.7295	1139.17	3110.03	2310.3	1921	1.2606376	1.9E-11	2.6E-10
ENSRNOG00000018456	ENSRNOG0000001845	2925.4945	3121.54	8236.86	6267.6	5138	1.2621196	4.6E-13	7.7E-12

ENSRNOG00000021144	ENSRNOG0000002114	1724.3391	1734.19	4163.11	4134.1	2939	1.2624682	2.5E-16	6.2E-15
ENSRNOG00000019145	Kdm2a	5322.6457	5544.22	14894.6	11177	9235	1.2624822	2.8E-13	4.8E-12
ENSRNOG00000024312	Xkr8	70.170591	60.2294	164.637	148.4	110.9	1.2629531	0.00053	0.00189
ENSRNOG00000006375	Vdac1	3777.8608	3605.45	8969.7	8750.1	6276	1.2630108	1.2E-17	3.6E-16
ENSRNOG00000016452	ENSRNOG0000001645	3675.7007	3533.8	9648.32	7656.3	6129	1.2631239	3.2E-14	6.4E-13
ENSRNOG00000006985	Ccnk	1306.4113	1460.04	3859.93	2784.8	2353	1.2640049	4.4E-11	5.7E-10
ENSRNOG00000004168	Slc35e4	590.2585	509.873	1555.02	1088.3	935.9	1.2642445	5.8E-09	5.3E-08
ENSRNOG00000067966	Ly96	275.52276	259.609	653.528	632.07	455.2	1.2643788	5.5E-09	5.1E-08
ENSRNOG00000020793	Ruvbl2	1570.5829	1478.73	3863.95	3465.4	2595	1.2651179	4.8E-15	1E-13
ENSRNOG00000005308	Tmx2	1363.1669	1247.16	3523.63	2755.5	2222	1.2661564	2.1E-12	3.2E-11
ENSRNOG00000010706	Ccdc117	454.045	473.527	1473.7	758.49	789.9	1.2663974	2.4E-06	1.4E-05
ENSRNOG00000020488	Luc7l	689.32286	747.675	1956.57	1502.3	1224	1.2669929	6.4E-11	8.1E-10
ENSRNOG00000026023	Taf4b	278.61852	227.418	655.536	563.37	431.2	1.2678869	4.8E-08	3.8E-07
ENSRNOG00000024410	Blvrb	3905.8189	3653.22	9317.04	8899.4	6444	1.268949	1.8E-17	5.1E-16
ENSRNOG00000021678	Alkbh3	572.71585	570.102	1611.23	1147.8	975.5	1.271195	1.2E-09	1.3E-08
ENSRNOG00000038735	AABR07064702.1	24.766091	36.3453	85.3301	62.291	52.18	1.2714848	0.01496	0.03621
ENSRNOG00000006114	Cfap69	147.56463	171.342	440.705	329.78	272.3	1.2719878	3.3E-06	1.9E-05
ENSRNOG00000010800	Hadhb	5896.3935	5630.41	13863.6	14032	9856	1.2750505	1.5E-18	5E-17
ENSRNOG00000043037	Zfp770	454.045	490.142	1324.12	961.85	807.5	1.2752681	2.4E-09	2.4E-08
ENSRNOG00000016698	Rnmt	836.88749	815.173	2291.87	1708.4	1413	1.2755739	4.1E-11	5.4E-10
ENSRNOG00000063609	Egln1	1585.0298	1597.12	3411.2	4293.5	2722	1.275876	1.7E-13	3E-12
ENSRNOG00000022701	Ceacam18	28.893773	28.0378	91.3534	46.719	48.75	1.2759514	0.02211	0.05099

ENSRNOG00000061129	Psmd14	3926.4573	3937.75	10695.4	8351.6	6728	1.276129	2.8E-14	5.6E-13
ENSRNOG00000008616	Ubr3	3012.1758	3119.47	8143.5	6713.7	5247	1.2767567	3.5E-15	7.7E-14
ENSRNOG00000008980	Rbm48	264.17164	245.071	643.489	590.85	435.9	1.2770917	8.1E-09	7.3E-08
ENSRNOG00000014274	Trit1	427.21507	370.722	1090.22	844.6	683.2	1.2774232	2.7E-09	2.7E-08
ENSRNOG00000009896	Tmem199	602.64155	659.408	1811.01	1249.5	1081	1.2776553	1.9E-09	1.9E-08
ENSRNOG00000018906	Ghdc	131.0539	117.343	322.247	280.31	212.7	1.2779923	6.4E-06	3.4E-05
ENSRNOG00000013493	Psma4	3517.8168	3395.69	9176.5	7596.8	5922	1.2786174	1.9E-15	4.3E-14
ENSRNOG00000059406	Ier5	1589.1575	1550.39	4496.39	3121.9	2689	1.2787419	4E-11	5.2E-10
ENSRNOG00000003889	Tbc1d15	2714.9827	2807.93	7670.67	5740	4733	1.2797864	4.4E-13	7.5E-12
ENSRNOG00000014986	Ube4b	4019.3302	4093.52	10906.2	8796.8	6954	1.2800824	4.2E-15	9.1E-14
ENSRNOG00000007506	Ndufaf4	503.57718	571.14	1390.38	1222.9	922	1.2817637	1.7E-11	2.3E-10
ENSRNOG00000064584	Rit1	1156.7828	1221.2	3221.46	2561.3	2040	1.2818524	5.9E-13	9.9E-12
ENSRNOG00000068511	ENSRNOG0000006851	37.149136	46.7297	101.392	102.6	71.97	1.2822893	0.00354	0.01033
ENSRNOG00000014935	Twnk	684.16326	683.292	1831.08	1495.9	1174	1.2824872	5.2E-12	7.7E-11
ENSRNOG00000003254	Il23a	22.70225	36.3453	83.3223	60.459	50.71	1.2830676	0.01631	0.039
ENSRNOG00000019063	Fbxo38	886.41967	887.864	2472.57	1852.3	1525	1.2851577	1.6E-11	2.2E-10
ENSRNOG00000017227	Ppme1	3051.3888	3139.2	9062.06	6028.5	5320	1.2854089	4.3E-11	5.6E-10
ENSRNOG00000002839	Slc19a2	367.36368	363.453	931.604	851.93	628.6	1.2869777	1.3E-10	1.5E-09
ENSRNOG00000014109	Psmd13	7251.305	6622.11	19155.1	14702	11933	1.2870839	4.2E-14	8.1E-13
ENSRNOG00000067659	ENSRNOG0000006765	25.798011	12.4612	41.1592	52.215	32.91	1.2876755	0.04956	0.10029
ENSRNOG00000010239	Cnbp	13427.349	13112.3	36007.3	28790	22834	1.2877551	1.1E-15	2.5E-14
ENSRNOG00000007213	Yars1	3140.1339	3017.7	7235.99	7801.1	5299	1.2880555	1.3E-17	3.9E-16

ENSRNOG00000034078	Mxi1	1723.3072	1671.88	4238.4	4054.4	2922	1.2883387	1.1E-16	2.7E-15
ENSRNOG00000010357	Lhx9	20.638409	23.8841	72.2796	36.642	38.36	1.2885003	0.03684	0.07828
ENSRNOG00000022533	Micall2	384.90633	476.643	1206.67	898.64	741.7	1.2886903	5.6E-09	5.2E-08
ENSRNOG00000063018	Ak6	530.40711	527.526	1510.84	1075.4	911.1	1.2892378	1.1E-09	1.1E-08
ENSRNOG00000033765	Eif1	7834.3401	7659.51	21825.4	16055	13343	1.2897095	1.5E-13	2.7E-12
ENSRNOG00000063715	ENSRNOG0000006371	20.638409	40.499	86.334	63.207	52.67	1.2897354	0.01686	0.04017
ENSRNOG00000070004	ENSRNOG0000007000	25.798011	37.3837	92.3573	62.291	54.46	1.2902668	0.01272	0.03157
ENSRNOG00000017895	Eno1	36351.462	34666.1	89383.8	84464	61216	1.2915764	1.6E-19	5.6E-18
ENSRNOG00000006986	RGD1304624	70.170591	46.7297	164.637	121.83	100.8	1.2920023	0.00131	0.00425
ENSRNOG00000007883	Timm10	310.60806	257.532	834.227	557.87	490.1	1.292275	2.6E-07	1.8E-06
ENSRNOG00000051578	Zfp54	301.32077	287.647	773.994	669.63	508.1	1.2931203	1.8E-09	1.8E-08
ENSRNOG00000066275	Cyb5d1	275.52276	245.071	740.866	535.89	449.3	1.293623	9.1E-08	6.9E-07
ENSRNOG00000006705	Mkks	212.57561	235.725	580.245	519.4	387	1.294276	2.3E-08	1.9E-07
ENSRNOG00000058186	Errfi1	1021.6013	1051.94	3110.03	1979.6	1791	1.2952091	1.3E-09	1.4E-08
ENSRNOG00000063207	ENSRNOG0000006320	111.44741	91.3825	272.052	227.18	175.5	1.2988166	2.7E-05	0.00013
ENSRNOG00000013828	Sirt3	163.04343	196.265	405.569	478.18	310.8	1.298887	3.3E-07	2.3E-06
ENSRNOG00000018000	Ranbp10	2395.0874	2424.75	7240.01	4629.7	4172	1.3001111	1.6E-10	1.9E-09
ENSRNOG00000010087	Zfp143	605.73731	533.757	1595.17	1211.9	986.6	1.3003447	1.6E-10	1.9E-09
ENSRNOG00000010258	Vhl	1399.2841	1313.62	4001.48	2687.7	2351	1.3017886	1E-10	1.3E-09
ENSRNOG00000042340	ENSRNOG0000004234	103.19205	125.651	360.394	204.28	198.4	1.3019275	0.00011	0.00047
ENSRNOG00000048929	Stk19	247.66091	229.495	700.711	476.35	413.6	1.3019339	2.8E-07	2E-06
ENSRNOG00000059348	Ube2b	1592.2533	1721.73	4943.12	3229.1	2872	1.3019996	1.8E-10	2.1E-09

ENSRNOG00000048981	Ahsa1	3703.5625	3510.96	10672.3	7125	6253	1.3026041	1.8E-11	2.5E-10
ENSRNOG00000019956	Kdm4a	2430.1727	2518.21	6205	6011.1	4291	1.303745	4.9E-18	1.5E-16
ENSRNOG00000013442	Ciz1	1675.8388	1706.15	4728.29	3628.5	2935	1.304939	1.7E-13	3.1E-12
ENSRNOG00000020979	Psmb4	3693.2433	3334.42	9619.21	7756.2	6101	1.3058653	5.2E-15	1.1E-13
ENSRNOG00000065376	ENSRNOG0000006537	120.73469	133.958	230.893	398.48	221	1.3063352	4.8E-05	0.00022
ENSRNOG00000053347	Ccdc28a	126.92622	148.497	341.32	340.77	239.4	1.3083832	1.3E-06	7.7E-06
ENSRNOG00000024763	ENSRNOG0000002476	126.92622	147.458	356.379	323.37	238.5	1.3085885	1.5E-06	9E-06
ENSRNOG00000034084	Ggct	61.915227	83.075	189.734	169.47	126	1.3086325	0.00021	0.00082
ENSRNOG00000009449	Trim35	2588.0565	2605.44	6613.58	6253	4515	1.3088212	3.5E-18	1.1E-16
ENSRNOG00000067620	ENSRNOG0000006762	534.5348	556.602	1585.13	1120.3	949.1	1.3096653	6.1E-10	6.7E-09
ENSRNOG00000011951	Plk2	4284.5337	4466.32	12969.2	8757.4	7619	1.3119038	6.2E-12	9.1E-11
ENSRNOG00000003923	Rab21	1799.6693	1734.19	5219.19	3557.9	3078	1.312351	1.8E-11	2.5E-10
ENSRNOG00000006913	Rpain	92.872841	121.497	231.897	300.46	186.7	1.3131195	2.6E-05	0.00012
ENSRNOG00000063974	ENSRNOG0000006397	399.35322	352.03	996.856	872.08	655.1	1.3143224	1.3E-10	1.6E-09
ENSRNOG00000010008	Polr3a	1000.9628	894.094	2481.6	2233.3	1652	1.3148735	2.2E-14	4.4E-13
ENSRNOG00000009430	Gnl2	2422.9492	2421.64	6994.06	5061.2	4225	1.3151081	5.8E-13	9.7E-12
ENSRNOG00000033433	Csrnp1	1104.1549	1093.47	3565.79	1904.5	1917	1.3154094	5.8E-08	4.5E-07
ENSRNOG00000019825	Zdhhc24	334.34223	306.339	840.251	754.82	558.9	1.3157032	2.9E-10	3.3E-09
ENSRNOG00000046283	Tmem41a	272.427	232.61	680.633	577.11	440.7	1.3159763	9.4E-09	8.4E-08
ENSRNOG00000001488	Psmb1	4980.0481	4711.39	13812.4	10331	8459	1.3168066	3.9E-14	7.6E-13
ENSRNOG00000016064	Tkt	4193.7247	4240.98	10414.3	10607	7364	1.3174438	1.6E-19	5.8E-18
ENSRNOG00000011422	Atmin	428.24699	443.413	1204.66	968.26	761.1	1.3174757	7.7E-11	9.7E-10

ENSRNOG00000005043	Cpeb2	1013.3459	1098.67	3056.83	2214.1	1846	1.3192286	9.8E-12	1.4E-10
ENSRNOG00000002469	Trim7	369.42752	339.569	785.037	983.84	619.5	1.3193408	6.2E-10	6.7E-09
ENSRNOG00000065817	Cep152	508.73678	593.986	1632.31	1124	964.8	1.3213173	1.6E-09	1.7E-08
ENSRNOG00000054989	Grhl1	139.30926	129.805	361.398	311.46	235.5	1.3216276	1.3E-06	7.9E-06
ENSRNOG00000004639	Traf6	501.51334	461.066	1392.39	1016.8	842.9	1.3231667	4.3E-10	4.7E-09
ENSRNOG00000012026	Psmc2	5290.6562	5019.81	14348.5	11456	9029	1.3234761	7.3E-16	1.7E-14
ENSRNOG00000002754	Areg	38.181057	31.1531	114.443	59.543	60.83	1.3252603	0.00973	0.02493
ENSRNOG00000057823	Ubc	1757.3605	1745.61	5458.11	3330.8	3073	1.3269373	5.4E-10	6E-09
ENSRNOG00000014089	Map3k2	289.96965	247.148	784.033	564.29	471.4	1.327236	4.2E-08	3.4E-07
ENSRNOG00000008145	Traf3	1298.1559	1268.97	3506.57	2939.6	2253	1.3281567	4.3E-15	9.2E-14
ENSRNOG00000040257	Chmp2b	1983.3511	2118.41	5893.8	4408	3601	1.3284742	1.5E-13	2.7E-12
ENSRNOG00000068617	ENSRNOG0000006861	66.042909	79.9597	205.796	161.22	128.3	1.3292028	0.00016	0.00065
ENSRNOG00000018630	Gapdh	59650.162	57795.3	153940	141571	1E+05	1.3312165	2E-20	7.6E-19
ENSRNOG00000004091	Cwc25	375.61905	398.76	1151.45	797.88	680.9	1.3313883	4.2E-09	4E-08
ENSRNOG00000005301	Eif2b4	1093.8357	1151.63	3297.76	2356.1	1975	1.3320133	6.3E-12	9.2E-11
ENSRNOG00000017621	Spns1	1430.2418	1542.08	4536.55	2947.8	2614	1.3321237	9.9E-11	1.2E-09
ENSRNOG00000004971	ENSRNOG0000000497	346.72527	327.108	960.717	739.25	593.5	1.3345867	1E-09	1.1E-08
ENSRNOG00000020317	Rab5if	3511.6253	3009.39	9605.16	6843.8	5742	1.3347453	1.4E-12	2.2E-11
ENSRNOG00000045941	Susd6	2922.3987	2894.12	8894.41	5808.7	5130	1.3377926	1.5E-11	2E-10
ENSRNOG00000065518	ENSRNOG0000006551	44.37258	50.8834	124.482	116.34	84.02	1.3379081	0.00108	0.00359
ENSRNOG00000029370	Abhd3	203.28833	308.416	661.559	632.99	451.6	1.3391144	6.4E-08	5E-07
ENSRNOG00000002914	Trmt1	1978.1915	2042.61	4939.11	5234.3	3549	1.339286	2.7E-18	8.5E-17

ENSRNOG00000026293	Jun	4355.7362	4265.9	12925	8891.2	7609	1.3393002	9E-13	1.5E-11
ENSRNOG00000020024	Taf7	218.76714	157.842	554.144	399.4	332.5	1.339493	1.1E-06	6.6E-06
ENSRNOG00000002512	Gpatch2	664.55677	723.791	2014.79	1500.5	1226	1.340001	1.4E-11	2E-10
ENSRNOG00000015063	Dhodh	551.04552	455.874	1224.74	1328.3	890	1.3423192	4.4E-12	6.6E-11
ENSRNOG00000053270	AABR07053741.1	480.87493	529.603	1476.71	1087.4	893.6	1.3430471	1.4E-10	1.7E-09
ENSRNOG00000007088	Xpot	5114.1978	5127.8	12677	13312	9058	1.3434195	2.8E-20	1E-18
ENSRNOG00000066159	Cpm	25.798011	32.1916	69.268	77.864	51.28	1.3438138	0.00884	0.02298
ENSRNOG00000018046	Lgals8	2032.8833	2140.22	5786.38	4808.3	3692	1.3440731	3.3E-16	8.1E-15
ENSRNOG00000020049	Slc7a6os	1068.0377	925.247	3076.9	1986.9	1764	1.3448155	4.1E-10	4.5E-09
ENSRNOG00000030245	Tango2	359.10832	396.683	1015.93	904.14	669	1.3449233	2.3E-11	3.1E-10
ENSRNOG00000014668	RGD621098	919.44113	859.826	2507.7	2018.1	1576	1.3466701	1.3E-13	2.3E-12
ENSRNOG00000046214	Cyp27b1	86.681318	82.0365	226.878	202.45	149.5	1.3470613	2.8E-05	0.00013
ENSRNOG00000012439	Bid	566.52433	643.831	1801.97	1279.7	1073	1.3479785	1.5E-10	1.8E-09
ENSRNOG00000003917	Uck2	2049.394	2014.57	5666.92	4692	3606	1.3498249	2.4E-16	6E-15
ENSRNOG00000050922	Nupr1l1	1549.9445	1430.97	3214.44	4387.9	2646	1.3508331	4.8E-13	8.1E-12
ENSRNOG00000003461	Zfp330	1591.2213	1603.35	4764.43	3387.6	2837	1.3513761	9.1E-13	1.5E-11
ENSRNOG00000001613	Zbtb11	643.91836	649.023	2109.16	1192.7	1149	1.3522363	2.4E-08	2E-07
ENSRNOG00000020862	Ptpn23	1863.6483	1892.03	5841.6	3752.1	3337	1.3528748	3.6E-11	4.7E-10
ENSRNOG00000064308	ENSRNOG0000006430	260.04395	275.186	825.192	543.22	475.9	1.353624	6.3E-08	4.9E-07
ENSRNOG00000026643	Chordc1	1924.5316	2064.41	6043.38	4157	3547	1.3544238	2.4E-12	3.8E-11
ENSRNOG00000048726	Clec2d	862.6855	898.248	2492.64	2010.7	1566	1.3544754	7E-14	1.3E-12
ENSRNOG00000071015	Tlcd1	40.244898	30.1147	90.3495	89.773	62.62	1.3559697	0.00397	0.01147

ENSRNOG00000060010	Ss18l1	313.70382	301.147	897.472	678.79	547.8	1.3577068	1.4E-09	1.5E-08
ENSRNOG00000000895	Rabgef1	810.05756	718.598	2339.05	1585.7	1363	1.3600177	1.2E-10	1.5E-09
ENSRNOG00000047386	Smg1	3736.584	3872.33	10917.2	8658.5	6796	1.3632483	2E-16	5.1E-15
ENSRNOG00000011891	Atp6v1b2	2312.5337	2010.41	6748.11	4384.2	3864	1.3645384	2.7E-11	3.6E-10
ENSRNOG00000065543	ENSRNOG0000006554	17.542648	21.8072	61.2369	40.306	35.22	1.3662678	0.02811	0.06244
ENSRNOG00000004662	AC115277.1	57.787545	57.114	183.711	113.59	103.1	1.3701129	0.00064	0.00224
ENSRNOG00000006086	Lynx1	75.330193	122.536	212.823	298.63	177.3	1.3710624	6.1E-05	0.00027
ENSRNOG00000031495	Tmem170b	224.95866	195.226	631.443	456.19	377	1.3714121	6.7E-08	5.2E-07
ENSRNOG00000028255	Usp36	1323.9539	1343.74	3941.25	2967.1	2394	1.3725829	5.5E-14	1.1E-12
ENSRNOG00000067700	ENSRNOG0000006770	300.28885	395.645	862.336	942.61	625.2	1.3751619	2.2E-10	2.5E-09
ENSRNOG00000006247	Copa	13951.565	13819.5	41220.5	30834	24956	1.3754905	7.3E-16	1.7E-14
ENSRNOG00000003599	Utp3	1548.9126	1510.93	4505.43	3437	2751	1.375984	1.5E-14	3E-13
ENSRNOG00000000585	Amd1	1301.2517	1304.28	4204.26	2560.4	2343	1.3762305	2.4E-10	2.7E-09
ENSRNOG00000070695	ENSRNOG0000007069	94.936682	106.959	354.371	170.39	181.7	1.3766964	0.00018	0.0007
ENSRNOG00000014616	Iars1	3399.146	3445.53	8790	8989.2	6156	1.3771438	7.7E-21	3.1E-19
ENSRNOG00000001114	Wipi2	1027.7928	1084.13	3396.14	2092.3	1900	1.3775914	3E-10	3.4E-09
ENSRNOG00000005248	Slc1a4	6452.5986	6214.01	16464.7	16489	11405	1.3794042	1.1E-21	4.8E-20
ENSRNOG00000016960	Polr3e	1337.3689	1369.7	3848.89	3195.2	2438	1.3795579	3.6E-16	8.8E-15
ENSRNOG00000049911	Cbr1	301.32077	330.223	924.577	720.01	569	1.3803726	3.8E-10	4.2E-09
ENSRNOG00000062500	ENSRNOG0000006250	31.989534	28.0378	81.3146	75.116	54.11	1.3814565	0.00561	0.01552
ENSRNOG00000032364	Tbcel	301.32077	313.608	849.285	756.66	555.2	1.3847131	3.2E-11	4.2E-10
ENSRNOG00000027161	RGD1359158	328.1507	348.915	879.402	888.57	611.3	1.3847675	5E-12	7.4E-11

ENSRNOG00000047276	ENSRNOG0000004727	216.7033	249.225	623.412	593.6	420.7	1.3851072	8.5E-10	9E-09
ENSRNOG00000006751	Psmd6	2817.1428	2735.24	8510.92	5995.5	5015	1.3854232	9.1E-14	1.7E-12
ENSRNOG00000006681	Rdh10	70.170591	65.4215	192.746	162.14	122.6	1.3874808	8E-05	0.00035
ENSRNOG00000018404	Aars1	12289.141	12248.4	33824.9	30471	22208	1.3897169	2.3E-21	9.7E-20
ENSRNOG00000002545	RGD1309748	1306.4113	1417.47	4538.56	2601.6	2466	1.3901009	1.1E-09	1.1E-08
ENSRNOG00000003100	Dusp12	233.21402	252.34	678.625	594.52	439.7	1.3904289	5.4E-10	5.9E-09
ENSRNOG00000057347	Cebpb	616.05651	557.641	1483.74	1599.4	1064	1.3934275	1.5E-14	3.1E-13
ENSRNOG00000012121	Sdf2	983.42019	915.902	2920.3	2072.1	1723	1.3940066	1.8E-12	2.9E-11
ENSRNOG00000054725	Gtf3c4	2276.4165	2277.29	7254.06	4718.6	4132	1.3945095	3.4E-12	5.2E-11
ENSRNOG00000066573	C4a	37.149136	40.499	126.489	77.864	70.5	1.3945459	0.00277	0.0083
ENSRNOG00000046181	Anks4b	35.085295	41.5375	116.45	85.193	69.57	1.3949857	0.00223	0.00687
ENSRNOG00000006999	Dnai4	127.95814	133.958	375.452	314.2	237.9	1.396307	3.5E-07	2.4E-06
ENSRNOG00000022466	Chchd4	116.60701	100.728	339.313	233.59	197.6	1.3973749	7.5E-06	4E-05
ENSRNOG00000021056	Kcnj14	92.872841	125.651	378.464	197.87	198.7	1.3979991	7.7E-05	0.00033
ENSRNOG00000011363	Napepld	210.51177	187.957	571.21	480.01	362.4	1.3990806	6.3E-09	5.8E-08
ENSRNOG00000004218	Klhl28	165.10727	167.188	530.051	348.1	302.6	1.4011542	4.9E-07	3.2E-06
ENSRNOG00000022335	Rad21l1	17.542648	17.6534	51.1981	42.138	32.13	1.4062776	0.0272	0.0607
ENSRNOG00000011329	Pkm	25340.871	23873.7	65829.7	64623	44917	1.4063652	5.4E-23	2.7E-21
ENSRNOG00000020624	Acadsb	2887.3134	2962.66	8210.76	7296.3	5339	1.4063854	5.8E-20	2.1E-18
ENSRNOG00000064281	ENSRNOG0000006428	65.010989	61.2678	198.769	136.49	115.4	1.4075104	0.00018	0.0007
ENSRNOG00000001335	Zkscan1	811.08948	776.751	2480.6	1743.2	1453	1.4112091	2.8E-12	4.3E-11
ENSRNOG00000018845	Snapc4	252.82051	315.685	845.27	667.8	520.4	1.4118984	9.5E-10	9.9E-09

ENSRNOG00000019862	Pinlyp	34.053375	16.615	79.3068	55.879	46.46	1.4142939	0.01377	0.03367
ENSRNOG00000064385	Ftl1	36501.09	34944.4	101444	89103	65498	1.4152317	1.4E-21	6.2E-20
ENSRNOG00000053606	Scarna6	23.73417	19.7303	58.2252	57.711	39.85	1.4152767	0.01386	0.03387
ENSRNOG00000012089	Cstf3	1201.1554	1163.05	3959.32	2347.8	2168	1.4154134	2.2E-10	2.6E-09
ENSRNOG00000022736	Cdkn2aip	676.93982	683.292	2162.37	1468.4	1248	1.4161125	1.7E-11	2.3E-10
ENSRNOG00000000924	Slc7a1	4799.462	4652.2	12218.3	13017	8672	1.4168192	5.2E-22	2.4E-20
ENSRNOG00000038445	Rnf225	14.446886	14.5381	49.1903	28.398	26.64	1.4185423	0.04664	0.09532
ENSRNOG00000013064	Lrp8	1259.9749	1162.01	3524.64	2957.9	2226	1.4202382	1.1E-16	2.8E-15
ENSRNOG00000020878	Keap1	693.45055	793.366	2088.08	1892.6	1367	1.4206933	1.3E-15	3E-14
ENSRNOG00000017395	Duox2	155.81999	166.15	474.837	387.49	296.1	1.4208328	3.3E-08	2.7E-07
ENSRNOG00000037795	Ldlrad1	20.638409	12.4612	63.2447	25.649	30.5	1.4223198	0.04658	0.09526
ENSRNOG00000018162	Nhej1	40.244898	38.4222	118.458	92.521	72.41	1.4223811	0.00133	0.00432
ENSRNOG00000000857	Msh5	21.67033	16.615	42.1631	60.459	35.23	1.4236329	0.02194	0.05061
ENSRNOG00000030963	ENSRNOG0000003096	254.88435	251.302	675.614	682.46	466.1	1.4238354	3.6E-11	4.7E-10
ENSRNOG00000069827	ENSRNOG0000006982	169.23495	143.304	471.825	367.34	287.9	1.4242547	9.4E-08	7.1E-07
ENSRNOG00000010819	Hspa4l	713.05703	606.447	1668.45	1874.2	1216	1.4249473	9.9E-15	2.1E-13
ENSRNOG00000015072	Ptgr1	6516.5777	6065.51	13345.6	20449	11594	1.4254628	3.4E-13	6E-12
ENSRNOG00000028543	AABR07052523.1	216.7033	164.073	533.062	491.92	351.4	1.428295	9.3E-09	8.4E-08
ENSRNOG00000050072	Jmjd7	59.851386	65.4215	145.563	191.45	115.6	1.4286588	9.9E-05	0.00042
ENSRNOG00000000796	Ranbp2	7511.349	7734.28	24306	16793	14086	1.4306755	9.9E-15	2.1E-13
ENSRNOG00000017195	Ctr9	4216.427	4200.48	12945.1	9788	7787	1.4333757	7.8E-17	2.1E-15
ENSRNOG00000014354	Cep95	130.02198	170.304	450.744	360.92	278	1.4339576	1.6E-07	1.2E-06

ENSRNOG00000024460	Tars3	812.1214	775.713	2506.7	1791.8	1472	1.4364999	5.6E-13	9.4E-12
ENSRNOG00000014338	Slc25a25	1201.1554	1190.05	4363.88	2112.4	2217	1.4372149	2.4E-08	2E-07
ENSRNOG00000051711	Kpna1	2234.1078	2252.37	7005.1	5146.4	4159	1.4373726	1.6E-15	3.6E-14
ENSRNOG00000069659	ENSRNOG0000006965	19.606489	17.6534	66.2563	34.81	34.58	1.4373841	0.02564	0.05772
ENSRNOG00000001350	Naa25	1677.9027	1640.73	5007.37	3983	3077	1.4376715	5.6E-17	1.5E-15
ENSRNOG00000018304	Lars1	3751.0309	3892.06	9542.92	11166	7088	1.4380477	3.2E-20	1.2E-18
ENSRNOG00000019325	Rnf185	1051.5269	1032.21	3422.24	2227.8	1933	1.4388665	5.6E-12	8.2E-11
ENSRNOG00000069802	ENSRNOG0000006980	61.915227	45.6912	164.637	127.33	99.89	1.4390771	0.00028	0.00105
ENSRNOG00000068079	Larp4	1784.1905	1931.49	6256.2	3828.2	3450	1.4402871	1.5E-11	2.1E-10
ENSRNOG00000009434	RGD1310507	24.766091	23.8841	76.2952	55.879	45.21	1.4407724	0.00877	0.02283
ENSRNOG00000053054	Ccnt1	925.63265	1130.86	3248.57	2335	1910	1.4408345	8.5E-13	1.4E-11
ENSRNOG00000064211	ENSRNOG0000006421	1740.8498	1734.19	5267.38	4172.6	3229	1.4416427	4.4E-17	1.2E-15
ENSRNOG00000019463	Mad2l1bp	446.82156	485.989	1676.49	859.25	867.1	1.4422839	6.8E-08	5.3E-07
ENSRNOG00000019068	Ctrb1	46.43642	44.6528	145.563	102.6	84.81	1.4447639	0.00061	0.00214
ENSRNOG00000012786	Pgrmc1	7355.529	7398.86	21618.6	18571	13736	1.44566	2.4E-21	9.9E-20
ENSRNOG00000011745	Psma1	5230.8048	4908.69	15850.3	11774	9441	1.4459037	1.4E-16	3.6E-15
ENSRNOG00000007899	Mterf1	198.12873	339.569	855.309	610.09	500.8	1.4461387	1.7E-07	1.3E-06
ENSRNOG00000026793	Nt5dc3	2126.7881	2399.83	7422.72	4929.3	4220	1.4481347	4.1E-13	7E-12
ENSRNOG00000019048	Sod2	2816.1109	2712.4	8848.23	6238.3	5154	1.4482082	6.3E-15	1.3E-13
ENSRNOG00000012739	Brf2	337.43799	277.263	1065.12	613.75	573.4	1.4488813	5.6E-08	4.4E-07
ENSRNOG00000021042	Psmd4	2708.7912	2700.97	8311.15	6476.5	5049	1.4506744	1.4E-17	4E-16
ENSRNOG00000001878	Klhl22	1401.348	1375.93	3894.06	3708.2	2595	1.4527143	2.3E-20	8.9E-19

ENSRNOG00000064955	ENSRNOG0000006495	327.11878	273.109	983.806	663.22	561.8	1.4556865	2.3E-09	2.3E-08
ENSRNOG00000017093	Pxdc1	582.00314	566.987	1813.01	1342	1076	1.4569637	4.9E-13	8.2E-12
ENSRNOG00000056853	Psma7	3783.0204	3533.8	11329.8	8760.2	6852	1.4571281	1.3E-17	3.7E-16
ENSRNOG00000048760	AABR07047219.1	15.478807	13.4997	29.1126	50.383	27.12	1.4577575	0.03982	0.08349
ENSRNOG00000062709	ENSRNOG0000006270	72.234432	46.7297	210.816	116.34	111.5	1.4578296	0.0005	0.00181
ENSRNOG00000018818	Txnl1	3451.7739	3299.11	10719.5	7834.1	6326	1.4584723	3.5E-16	8.6E-15
ENSRNOG00000059404	Snora64	19.606489	15.5766	60.233	36.642	33.01	1.4593551	0.02432	0.05526
ENSRNOG00000066227	ENSRNOG0000006622	459.2046	404.99	1214.7	1164.3	810.8	1.4608209	2.3E-14	4.7E-13
ENSRNOG00000019551	Psmb6	1900.7975	1667.73	5446.07	4385.1	3350	1.4619294	4.1E-17	1.1E-15
ENSRNOG00000007390	Nfkbia	494.2899	460.028	1617.26	1014.1	896.4	1.462798	3.3E-10	3.8E-09
ENSRNOG00000018484	Plk3	472.61957	429.913	1652.39	836.35	847.8	1.4628679	6.2E-08	4.9E-07
ENSRNOG00000036696	Hgs	3354.7734	3299.11	10880.1	7480.5	6254	1.4642638	8E-15	1.7E-13
ENSRNOG00000039300	Ahcyl2	1136.1444	1157.86	3778.62	2555.8	2157	1.4651403	3.1E-13	5.3E-12
ENSRNOG00000029667	Tbce	910.15384	1127.74	2928.33	2706	1918	1.4671372	1.3E-16	3.3E-15
ENSRNOG00000064112	Arl5b	314.73574	396.683	1172.54	796.05	670	1.4679736	7.6E-10	8.1E-09
ENSRNOG00000012004	Ubap1	977.22867	888.902	3141.15	2024.5	1758	1.468635	6.2E-12	9E-11
ENSRNOG00000009222	Epha2	2377.5447	2436.17	8571.16	4762.5	4537	1.4697444	9.7E-11	1.2E-09
ENSRNOG00000063195	ENSRNOG0000006319	222.89482	268.955	814.15	548.71	463.7	1.4697912	6.7E-09	6.2E-08
ENSRNOG00000027022	ENSRNOG0000002702	19.606489	12.4612	53.2058	35.726	30.25	1.4698851	0.02947	0.06489
ENSRNOG00000007447	Pla2g4b	173.36264	202.495	532.058	509.32	354.3	1.4702023	7.2E-10	7.8E-09
ENSRNOG00000030355	Mroh1	1006.1224	1079.97	3046.79	2733.5	1967	1.4702532	1.2E-18	4E-17
ENSRNOG00000031653	Mix23	317.8315	416.413	1102.26	933.45	692.5	1.4710389	1.1E-11	1.5E-10

ENSRNOG00000000808	Hsf2	1535.4976	1721.73	5385.84	3656	3075	1.4728298	1.2E-13	2.2E-12
ENSRNOG00000051291	Dnhd1	152.72423	104.882	444.72	271.15	243.4	1.4734857	4.5E-06	2.5E-05
ENSRNOG00000047848	Cbr1	117.63893	136.035	356.379	348.1	239.5	1.4735826	5.2E-08	4.1E-07
ENSRNOG00000008106	Shmt2	2497.2475	2565.98	6246.16	7843.2	4788	1.4765576	1.2E-18	3.9E-17
ENSRNOG00000067040	ENSRNOG0000006704	217.73522	180.688	515.996	592.68	376.8	1.4766997	7.5E-10	8E-09
ENSRNOG00000061139	Taf1a	330.21455	348.915	1232.77	662.3	643.6	1.4799089	3.8E-08	3.1E-07
ENSRNOG00000012582	Eif4ebp1	1415.7949	1408.12	3591.9	4300.9	2679	1.4829393	7.3E-19	2.5E-17
ENSRNOG00000059857	Rnd1	1272.3579	1268.97	4845.75	2269.1	2414	1.4850439	1.5E-08	1.3E-07
ENSRNOG00000011784	Mocs1	553.10936	526.488	1536.95	1488.6	1026	1.4866345	1.1E-16	2.8E-15
ENSRNOG00000005929	Them6	25.798011	22.8456	64.2485	72.368	46.32	1.4901734	0.00562	0.01556
ENSRNOG00000033522	Prdm2	1682.0303	1829.73	6013.26	3866.6	3348	1.4921716	3.9E-13	6.7E-12
ENSRNOG00000011032	Lhfpl2	137.24542	134.997	403.561	362.76	259.6	1.4927536	1.3E-08	1.1E-07
ENSRNOG00000020107	Stk11ip	620.18419	679.138	2027.84	1634.2	1240	1.4946967	2.2E-15	4.9E-14
ENSRNOG00000018997	Myh7b	45.4045	75.8059	156.606	185.04	115.7	1.4957221	0.0001	0.00043
ENSRNOG00000019414	Tmem79	10.319205	21.8072	66.2563	24.733	30.78	1.4996358	0.04125	0.08603
ENSRNOG00000020289	Akt1s1	1640.7535	1556.62	5265.37	3777.8	3060	1.499798	1.3E-15	3E-14
ENSRNOG00000069919	ENSRNOG0000006991	82.553636	109.036	310.2	232.68	183.6	1.5019945	2.4E-06	1.4E-05
ENSRNOG00000020769	Crebrf	266.23548	295.955	910.522	682.46	538.8	1.5021537	5E-11	6.3E-10
ENSRNOG00000002393	Eprs	14582.068	14677.3	39644.4	43248	28038	1.5023421	2.9E-25	1.8E-23
ENSRNOG00000007753	Plaa	1993.6703	2006.26	6151.8	5219.6	3843	1.5072954	8.6E-21	3.5E-19
ENSRNOG00000018266	Slc23a3	70.170591	57.114	225.874	136.49	122.4	1.5079726	8.9E-05	0.00038
ENSRNOG00000001979	Rcan1	1104.1549	1073.74	3922.17	2276.4	2094	1.5087712	3.1E-11	4.1E-10

ENSRNOG0000 0008961	Mapre3	1455.0078	1551.43	4696.17	3863.9	2892	1.5094696	4.6E-19	1.6E-17
ENSRNOG0000 0018107	Zfand5	5314.3903	5032.27	17988.6	11497	9958	1.510811	3.7E-14	7.2E-13
ENSRNOG0000 0069709	Tmem167b	692.41863	695.753	2177.42	1783.5	1337	1.5124647	1.2E-16	3.2E-15
ENSRNOG0000 0068086	ENSRNOG0 000006808	11.351125	12.4612	33.1282	34.81	22.94	1.5127634	0.04296	0.08909
ENSRNOG0000 0014964	Hp	68.10675	80.9981	173.672	251.91	143.7	1.5142221	1.5E-05	7.3E-05
ENSRNOG0000 0019861	Tollip	3117.4317	2931.51	11077.9	6212.6	5835	1.5151383	1.5E-11	2.1E-10
ENSRNOG0000 0009491	Phc3	529.37519	624.101	1917.42	1381.4	1113	1.5156891	5E-13	8.4E-12
ENSRNOG0000 0049849	ENSRNOG0 000004984	479.84301	391.491	1317.1	1176.2	841.2	1.5165482	2.6E-14	5.2E-13
ENSRNOG0000 0020651	Cars	3284.6028	3386.34	9296.97	9831	6450	1.519743	1.2E-24	7E-23
ENSRNOG0000 0006420	Rbm38	826.56828	830.75	2805.85	1948.4	1603	1.5201294	4.8E-14	9.3E-13
ENSRNOG0000 0010833	Mthfd2	2857.3877	2771.59	7652.6	8495.4	5444	1.5204499	2.2E-23	1.1E-21
ENSRNOG0000 0025110	Vwa3a	29.925693	41.5375	107.416	98.017	69.22	1.5232762	0.00083	0.00281
ENSRNOG0000 0006947	Pdhx	708.92935	786.097	2681.37	1617.7	1449	1.5235855	2.5E-11	3.4E-10
ENSRNOG0000 0028341	Alkbh5	518.02407	529.603	1650.38	1364	1016	1.5245024	9.3E-16	2.2E-14
ENSRNOG0000 0052802	Aldoa	1114.4741	1121.51	3290.73	3146.6	2168	1.5255222	1.5E-21	6.4E-20
ENSRNOG0000 0028557	Hibch	468.49189	395.645	1251.84	1236.7	838.2	1.5258837	2.6E-15	5.7E-14
ENSRNOG0000 0066402	ENSRNOG0 000006640	35.085295	45.6912	129.501	103.51	78.45	1.5278238	0.00043	0.00157
ENSRNOG0000 0017822	Oga	6162.629	6125.74	19946.2	15538	11943	1.5298492	2.9E-20	1.1E-18
ENSRNOG0000 0002294	Snx29	283.77813	337.492	958.709	836.35	604.1	1.5305634	5.3E-13	8.9E-12
ENSRNOG0000 0055716	Oscar	45.4045	49.845	188.73	87.025	92.75	1.5318054	0.00077	0.00264
ENSRNOG0000 0062231	FAM120C	168.20303	153.689	532.058	400.31	313.6	1.533666	4.5E-09	4.2E-08
ENSRNOG0000 0053586	Snord19	10.319205	19.7303	53.2058	33.894	29.29	1.5341765	0.02817	0.0625

ENSRNOG00000008696	Nopchap1	535.56672	396.683	1458.64	1242.2	908.3	1.5343487	2.6E-13	4.6E-12
ENSRNOG00000026742	Ypel5	2171.1606	2114.26	6798.3	5654.8	4185	1.5389173	3.6E-21	1.5E-19
ENSRNOG00000015495	Slc25a37	145.50078	174.457	503.95	426.88	312.7	1.5403237	1.6E-09	1.6E-08
ENSRNOG00000046891	Hk1	5246.2836	5228.53	16675.5	13813	10241	1.5413132	1.6E-22	7.4E-21
ENSRNOG00000009244	Abhd4	2095.8304	2152.68	6568.41	5803.2	4155	1.541949	6.7E-23	3.3E-21
ENSRNOG00000051204	Dop1b	346.72527	372.799	1053.07	1043.4	704	1.5428446	2.3E-15	5.2E-14
ENSRNOG00000011815	Sgk1	373.5552	388.375	1460.65	763.99	746.6	1.5453137	8.3E-09	7.5E-08
ENSRNOG00000009006	Ccdc47	2711.887	2787.17	8823.13	7235	5389	1.5459854	2.1E-21	8.9E-20
ENSRNOG00000020255	Sars1	4697.3019	4501.62	14360.6	12513	9018	1.5466314	5.2E-24	2.9E-22
ENSRNOG00000003879	Rnf167	383.87441	380.068	1285.97	947.19	749.3	1.5471479	5.4E-13	9.1E-12
ENSRNOG00000066094	ENSRNOG0000006609	225.99058	188.996	665.575	549.63	407.5	1.5496054	7.9E-11	9.9E-10
ENSRNOG00000007000	Grhl2	77.394034	55.0372	207.804	180.46	130.2	1.5512016	1.2E-05	6.2E-05
ENSRNOG00000070263	5_8S_rRNA	26.829932	8.3075	80.3107	22.901	34.59	1.5514235	0.04208	0.08751
ENSRNOG00000000840	Atp6v1g2	44.37258	28.0378	114.443	98.017	71.22	1.5521565	0.00073	0.00252
ENSRNOG00000007478	Cry2	948.3349	1013.51	3444.32	2315.8	1930	1.5536682	3E-14	6E-13
ENSRNOG00000012843	Aspg	28.893773	38.4222	116.45	81.528	66.32	1.5553376	0.00106	0.00351
ENSRNOG00000015416	Nabp1	359.10832	413.298	1115.31	1155.1	760.7	1.5556492	1.5E-15	3.5E-14
ENSRNOG00000020770	Arl4d	173.36264	154.727	608.353	357.26	323.4	1.5564364	9.8E-08	7.4E-07
ENSRNOG00000066585	Prdx6	4615.7802	4544.2	14090.5	12860	9027	1.5568482	8.8E-26	5.8E-24
ENSRNOG00000003625	Ubxn4	6134.7671	6371.85	21417.9	15391	12329	1.5573436	3.1E-18	9.7E-17
ENSRNOG00000069385	ENSRNOG0000006938	19.606489	17.6534	83.3223	26.565	36.79	1.5573779	0.02281	0.05237
ENSRNOG00000006178	Dync1h1	14318.928	14650.3	45127.6	40219	28579	1.5588082	3.3E-26	2.3E-24

ENSRNOG00000058914	Rbx1	15.478807	12.4612	52.2019	30.23	27.59	1.558818	0.0276	0.06151
ENSRNOG00000016010	Mul1	592.32234	631.37	1991.7	1617.7	1208	1.5603286	8.4E-17	2.2E-15
ENSRNOG00000011937	Sgtb	227.0225	214.956	727.816	578.03	437	1.5624545	1.9E-11	2.6E-10
ENSRNOG00000034066	Hspa8	31156.774	30202.9	118277	63235	60718	1.5646884	6.4E-12	9.3E-11
ENSRNOG00000002894	Mnt	758.46153	826.596	2374.18	2316.7	1569	1.5653145	1.6E-20	6.5E-19
ENSRNOG00000053325	AABR07009105.1	28.893773	37.3837	118.458	77.864	65.65	1.5654517	0.00113	0.00373
ENSRNOG00000010489	Samd4a	1006.1224	950.17	3198.37	2612.6	1942	1.5704905	4.5E-19	1.5E-17
ENSRNOG00000033169	Cpeb4	1675.8388	1690.58	6380.68	3625.7	3343	1.5714962	3.3E-12	4.9E-11
ENSRNOG00000008898	Trmt44	301.32077	374.876	1245.82	764.9	671.7	1.5717356	4.7E-10	5.2E-09
ENSRNOG00000066929	ENSRNOG0000006692	72.234432	71.6522	288.115	140.16	143	1.5720905	5.8E-05	0.00026
ENSRNOG00000002950	Lyl1	27.861852	17.6534	84.3262	51.299	45.29	1.5733505	0.00631	0.01717
ENSRNOG00000007508	Lrtm2	31.989534	30.1147	104.404	80.612	61.78	1.5739565	0.001	0.00335
ENSRNOG00000061099	AABR07033324.1	46.43642	39.4606	185.718	70.536	85.54	1.5747963	0.00149	0.00478
ENSRNOG00000034242	Vcp	17419.849	17356.4	58958.1	44771	34626	1.5766323	5.6E-21	2.3E-19
ENSRNOG00000011732	Psmb7	4596.1737	4200.48	15067.3	11220	8771	1.5792757	2.9E-19	1E-17
ENSRNOG00000000848	Gpank1	199.16065	241.956	711.753	609.17	440.5	1.5820862	9.1E-12	1.3E-10
ENSRNOG00000019199	Cracr2b	24.766091	13.4997	80.3107	34.81	38.35	1.586377	0.01555	0.03741
ENSRNOG00000004226	Irak3	846.17477	823.481	2675.35	2342.3	1672	1.5873441	1.7E-20	6.5E-19
ENSRNOG00000015948	Slc1a5	821.40868	742.483	1985.68	2713.3	1566	1.5874346	6.5E-16	1.5E-14
ENSRNOG00000018454	Apoe	133.11774	153.689	499.934	362.76	287.4	1.5881302	7E-09	6.4E-08
ENSRNOG00000033568	Mapk8ip3	1765.6159	2007.3	5657.89	5700.6	3783	1.590033	5.1E-24	2.8E-22
ENSRNOG00000014258	Rab32	1623.2109	1576.35	5172.01	4464.8	3209	1.5906047	7.5E-23	3.7E-21

ENSRNOG00000042289	Plcxd2	376.65097	339.569	1199.64	960.94	719.2	1.5925836	2E-14	4E-13
ENSRNOG00000001548	Nfe2l2	4311.3637	4158.94	14697.9	10934	8526	1.5973993	5.6E-20	2E-18
ENSRNOG00000029082	AABR07005031.1	34.053375	29.0762	95.3689	96.185	63.67	1.6012926	0.00061	0.00214
ENSRNOG00000031290	Trappc3l	43.340659	60.2294	164.637	150.23	104.6	1.6040481	3E-05	0.00014
ENSRNOG00000047295	Prr22	36.117216	45.6912	133.517	115.42	82.69	1.6051276	0.00014	0.00057
ENSRNOG00000009749	Wdr45	531.43903	528.564	1769.85	1457.4	1072	1.6060171	1.6E-17	4.7E-16
ENSRNOG00000067497	SNORD14	12.383045	20.7687	66.2563	34.81	33.55	1.6064202	0.01627	0.03891
ENSRNOG00000020579	Col7a1	34.053375	33.23	114.443	90.689	68.1	1.6074163	0.00044	0.00162
ENSRNOG00000036572	Sfxn4	61.915227	80.9981	239.928	196.03	144.7	1.60863	2.2E-06	1.3E-05
ENSRNOG00000048256	Fhip1a	13.414966	22.8456	60.233	50.383	36.72	1.6087897	0.00915	0.02367
ENSRNOG00000015020	Idh1	2353.8106	2295.98	6639.69	7550.1	4710	1.6096593	5.2E-25	3.2E-23
ENSRNOG00000037613	Kdm6b	1208.3789	1413.31	4556.63	3448	2657	1.6102212	6E-18	1.8E-16
ENSRNOG00000033697	Casp4	421.02355	477.681	1143.42	1600.3	910.6	1.6106244	6.7E-14	1.3E-12
ENSRNOG00000004094	Ptger1	34.053375	49.845	170.66	86.109	85.17	1.6122525	0.00056	0.002
ENSRNOG00000030228	ENSRNOG0000003022	48.500261	43.6144	200.777	81.528	93.6	1.6137963	0.00064	0.00223
ENSRNOG00000066854	ENSRNOG0000006685	158.91575	138.112	503.95	405.81	301.7	1.6143245	5.6E-10	6.1E-09
ENSRNOG00000006104	Tg	43.340659	41.5375	146.567	113.59	86.26	1.6150587	9.8E-05	0.00042
ENSRNOG00000014710	Prom2	40.244898	46.7297	89.3456	176.8	88.28	1.6153406	0.00036	0.00136
ENSRNOG00000050655	P4ha1	10749.515	11434.2	34946.2	33022	22538	1.6153474	6.6E-29	6.1E-27
ENSRNOG00000013946	Rnf149	1473.5824	1376.97	4857.79	3886.8	2899	1.6170289	8.9E-21	3.6E-19
ENSRNOG00000036661	Rab40b	25.798011	23.8841	44.1709	108.09	50.49	1.6181	0.00539	0.01499
ENSRNOG00000016448	Eef2k	565.49241	627.216	1764.83	1898.1	1214	1.6188496	9.3E-20	3.4E-18

ENSRNOG00000007606	Rnf43	12.383045	17.6534	51.1981	41.222	30.61	1.6209374	0.01459	0.03541
ENSRNOG00000045649	Arrdc3	1084.5484	1205.63	3771.59	3279.5	2335	1.6223019	6.3E-22	2.8E-20
ENSRNOG00000015588	Nol3	1461.1994	1592.96	4559.64	4846.8	3115	1.6229208	8.2E-25	5E-23
ENSRNOG00000060185	Kansl2	4378.4385	4383.24	15349.4	11647	8939	1.6234216	2.1E-21	9.1E-20
ENSRNOG00000068394	ENSRNOG0000006839	570.65201	670.83	2426.39	1401.6	1267	1.6242017	1.5E-11	2.1E-10
ENSRNOG00000025269	Slc25a44	525.24751	547.256	2026.84	1280.6	1095	1.6243933	6.5E-13	1.1E-11
ENSRNOG00000010101	AC118490.1	8.2553636	17.6534	56.2175	23.817	26.49	1.6251003	0.03465	0.07436
ENSRNOG00000017215	RGD1308601	793.54683	834.903	2949.41	2079.4	1664	1.6265003	3.4E-16	8.3E-15
ENSRNOG00000051048	AABR07039058.1	101.1282	86.1903	330.278	249.17	191.7	1.6283616	1.3E-07	9.7E-07
ENSRNOG00000011624	Eef1a2	11.351125	7.26906	31.1204	26.565	19.08	1.6304533	0.04915	0.09958
ENSRNOG00000026813	Ak6	47.468341	44.6528	133.517	152.06	94.43	1.6326893	3.4E-05	0.00016
ENSRNOG00000008432	Slc22a5	124.86238	136.035	457.771	352.68	267.8	1.6346627	2E-09	2E-08
ENSRNOG00000004488	Bdkrb1	149.62847	158.881	557.155	403.06	317.2	1.637397	6.5E-10	7E-09
ENSRNOG00000013090	Gadd45g	2555.035	2409.17	9155.42	6293.3	5103	1.6377566	8.2E-18	2.5E-16
ENSRNOG00000007440	Dzank1	114.54317	178.611	528.043	384.74	301.5	1.6382078	2.1E-08	1.8E-07
ENSRNOG00000028103	Psmd3	4661.1847	4555.62	16971.7	11768	9489	1.6406789	8.7E-19	2.9E-17
ENSRNOG00000015024	Mcoln3	24.766091	51.9219	139.54	99.849	79.02	1.6417617	0.00052	0.00187
ENSRNOG00000018796	Herpud1	2170.1287	1991.72	6839.46	6156.8	4290	1.6427437	1.3E-25	8.5E-24
ENSRNOG00000017801	Atf4	9383.2527	9179.78	30377.5	27668	19152	1.6447503	2.1E-29	2E-27
ENSRNOG00000046036	Itprip	910.15384	884.748	3508.57	2106	1852	1.6450285	1.9E-13	3.3E-12
ENSRNOG00000002764	Mettl16	398.3213	406.029	1407.44	1110.3	830.5	1.6458974	4.1E-16	9.8E-15
ENSRNOG00000019735	Dph2	369.42752	289.724	1204.66	859.25	680.8	1.6462257	1.5E-12	2.4E-11

ENSRNOG00000012994	Pus3	231.15018	252.34	776.002	738.34	499.5	1.6470628	1.5E-14	3E-13
ENSRNOG00000024239	Fam89b	2157.7457	2068.57	7873.46	5392.8	4373	1.6501838	7.9E-18	2.3E-16
ENSRNOG00000070031	ENSRNOG0000007003	641.85452	653.177	2639.21	1429	1341	1.6510996	2.3E-11	3E-10
ENSRNOG00000000432	Fkbpl	101.1282	98.6515	350.355	277.56	206.9	1.6515349	1.9E-08	1.6E-07
ENSRNOG00000037871	Sfxn5	29.925693	40.499	125.485	96.185	73.02	1.6535899	0.00026	0.001
ENSRNOG00000000521	Cdkn1a	4672.5358	4480.86	16147.5	12674	9494	1.6547008	2.3E-23	1.2E-21
ENSRNOG00000000247	Mfsd11	1047.3993	1150.59	3878	3044	2280	1.6548807	3.7E-20	1.4E-18
ENSRNOG00000025704	Yod1	119.70277	144.343	505.957	326.11	274	1.6551474	1.9E-08	1.6E-07
ENSRNOG00000002419	Plp1	22.70225	21.8072	56.2175	84.277	46.25	1.6596699	0.00276	0.00828
ENSRNOG00000003942	Asnsd1	2932.7179	2808.97	9955.51	8188.6	5971	1.6598942	1.3E-24	7.7E-23
ENSRNOG00000039091	Pnpla8	1654.1685	1700.96	5878.74	4724.1	3489	1.6599134	1.1E-22	5.1E-21
ENSRNOG00000060031	Snord35a	10.319205	26.9994	60.233	57.711	38.82	1.6605387	0.00848	0.02215
ENSRNOG00000068822	ENSRNOG0000006882	134.14966	176.534	636.462	349.01	324	1.6645991	7.4E-08	5.6E-07
ENSRNOG00000067655	ENSRNOG0000006765	63.979068	51.9219	174.676	193.29	121	1.6668564	3.2E-06	1.8E-05
ENSRNOG00000021007	Snx15	774.97226	748.713	3095.98	1746.9	1592	1.6680216	1.8E-12	2.9E-11
ENSRNOG00000014127	Rnf151	36.117216	31.1531	130.505	84.277	70.51	1.6733861	0.00034	0.00129
ENSRNOG00000059552	Zkscan5	824.50444	807.904	3183.31	2031.8	1712	1.675446	5.7E-15	1.2E-13
ENSRNOG00000024104	Mbd1	116.60701	110.074	453.755	271.15	237.9	1.6760976	8.4E-08	6.4E-07
ENSRNOG00000000728	Clic2	41.276818	43.6144	115.447	155.73	89.02	1.676517	4.9E-05	0.00022
ENSRNOG00000020206	Ctsd	15794.574	15040.7	56508.6	42371	32429	1.6810759	6.2E-23	3.1E-21
ENSRNOG00000067100	ENSRNOG0000006710	10.319205	12.4612	28.1087	44.886	23.94	1.6817931	0.02544	0.05732
ENSRNOG00000007900	Tspan33	111.44741	149.535	419.623	417.72	274.6	1.6819939	3.9E-10	4.3E-09

ENSRNOG00000030109	Kpna5	696.54631	820.365	2974.51	1903.6	1599	1.6849402	1.8E-14	3.6E-13
ENSRNOG00000070409	Spatc1l	20.638409	8.3075	49.1903	43.97	30.53	1.6854521	0.01509	0.03648
ENSRNOG00000011459	Rhbdf2	118.67085	85.1518	422.635	233.59	215	1.6857005	8.7E-07	5.5E-06
ENSRNOG00000027341	Tenm2	7.2234432	10.3844	30.1165	26.565	18.57	1.6864522	0.04425	0.09124
ENSRNOG00000005227	Tfap4	119.70277	105.921	341.32	385.66	238.2	1.688251	8.2E-10	8.7E-09
ENSRNOG00000013825	Rap1gap	17.542648	45.6912	112.435	91.605	66.82	1.6900112	0.00108	0.00359
ENSRNOG00000000529	Pim1	164.07535	196.265	766.967	396.65	381	1.6904164	2.7E-08	2.3E-07
ENSRNOG00000031706	RGD1563601	165.10727	207.687	599.318	604.59	394.2	1.6913939	9E-13	1.5E-11
ENSRNOG00000022256	Cxcl10	16.510727	13.4997	44.1709	53.131	31.83	1.6975385	0.00891	0.02314
ENSRNOG00000016621	Actl7b	14.446886	14.5381	49.1903	44.886	30.77	1.6981948	0.00958	0.02461
ENSRNOG00000011170	RGD1359634	187.80952	163.035	626.423	512.99	372.6	1.6989061	3.3E-12	4.9E-11
ENSRNOG00000068535	ENSRNOG0000006853	19.606489	14.5381	88.3418	22.901	36.35	1.7008697	0.01802	0.04265
ENSRNOG00000029861	Gsta2	30.957614	35.3069	118.458	97.101	70.46	1.7011942	0.00017	0.00067
ENSRNOG00000063284	ENSRNOG0000006328	37.149136	30.1147	102.396	116.34	71.5	1.7015729	0.00015	0.00061
ENSRNOG00000020457	Tacc2	3334.135	3309.5	11444.3	10165	7063	1.7016006	3.9E-29	3.6E-27
ENSRNOG00000011921	Dusp4	25.798011	30.1147	127.493	54.963	59.59	1.7042402	0.00173	0.00548
ENSRNOG00000022218	Ifi44	153.75615	197.303	620.4	524.9	374.1	1.7057026	5.7E-12	8.3E-11
ENSRNOG00000020136	Tgm1	11.351125	30.1147	81.3146	54.047	44.21	1.7061813	0.00541	0.01503
ENSRNOG00000000250	Jmjd6	577.87545	617.87	2191.48	1712.1	1275	1.7066644	4.4E-19	1.5E-17
ENSRNOG00000018163	Ipcef1	139.30926	131.882	543.101	344.43	289.7	1.7096194	3.3E-09	3.2E-08
ENSRNOG00000018567	Slc20a1	5405.1993	5320.95	20833.6	14274	11458	1.7106051	5.2E-20	1.9E-18
ENSRNOG00000008012	Abcb1b	13.414966	17.6534	42.1631	59.543	33.19	1.7122832	0.00792	0.02089

ENSRNOG00000036698	Nploc4	2931.686	2893.09	11354.9	7772.7	6238	1.7153098	1.3E-19	4.8E-18
ENSRNOG00000019996	Slc16a1	2217.5971	2199.41	7844.35	6662.4	4731	1.7155259	5E-27	3.8E-25
ENSRNOG00000020929	Lin37	509.7687	484.95	1703.59	1565.5	1066	1.7164171	2.1E-21	8.8E-20
ENSRNOG00000066548	Rnf113a1	125.8943	84.1134	384.487	307.79	225.6	1.7202309	1.5E-08	1.3E-07
ENSRNOG00000018421	Aen	960.71794	1004.17	3280.69	3198.8	2111	1.7214322	1.1E-26	7.6E-25
ENSRNOG00000069483	ENSRNOG0000006948	29.925693	13.4997	91.3534	52.215	46.75	1.7231303	0.00392	0.01134
ENSRNOG00000063050	ENSRNOG0000006305	10.319205	13.4997	29.1126	49.467	25.6	1.72404	0.01928	0.04518
ENSRNOG00000009074	AABR07017902.1	8.2553636	11.4228	31.1204	33.894	21.17	1.7247071	0.02859	0.06327
ENSRNOG00000005811	ENSRNOG0000000581	63.979068	34.2684	208.808	116.34	105.8	1.7250193	9.3E-05	0.0004
ENSRNOG00000038319	Akr1c19	9.2872841	7.26906	29.1126	25.649	17.83	1.7251361	0.04328	0.08967
ENSRNOG00000027921	Rlf	997.86708	1020.78	3967.35	2710.6	2174	1.7258239	5.4E-18	1.6E-16
ENSRNOG00000043095	Etv3	920.47305	1046.74	3860.94	2656.5	2121	1.7279861	1.2E-17	3.5E-16
ENSRNOG00000014852	Fbxo30	2213.4694	2162.03	9041.98	5457.8	4719	1.7284136	4.4E-16	1E-14
ENSRNOG00000019992	Cnbd2	47.468341	39.4606	178.691	110.84	94.12	1.7343856	4.1E-05	0.00019
ENSRNOG00000014981	Usp14	3464.157	3586.76	13351.7	10164	7642	1.7376612	4E-24	2.2E-22
ENSRNOG00000047394	Ufd1	2303.2465	2340.64	8869.31	6739.4	5063	1.7488695	8.9E-24	4.8E-22
ENSRNOG00000005569	Phospho1	124.86238	140.189	426.651	464.44	289	1.749565	8.5E-12	1.2E-10
ENSRNOG00000066092	ENSRNOG0000006609	478.81109	541.026	2156.34	1279.7	1114	1.752083	1.8E-13	3.3E-12
ENSRNOG00000046971	Ccer2	7.2234432	9.34593	31.1204	24.733	18.11	1.7524603	0.03939	0.08278
ENSRNOG00000063660	ENSRNOG0000006366	22.70225	30.1147	90.3495	87.941	57.78	1.7552525	0.00039	0.00144
ENSRNOG00000070689	Tnfrsf22	333.31031	248.186	1187.59	784.14	638.3	1.7610872	1.8E-12	2.9E-11
ENSRNOG00000002218	Stbd1	1258.943	1153.7	3930.2	4251.4	2649	1.7618012	8E-28	6.7E-26

ENSRNOG00000070982	ENSRNOG0000007098	7.2234432	8.3075	26.101	26.565	17.05	1.7619071	0.04314	0.08945
ENSRNOG00000065142	ENSRNOG0000006514	57.787545	42.5759	208.808	131.91	110.3	1.7619802	1.1E-05	5.9E-05
ENSRNOG00000070627	ENSRNOG0000007062	8.2553636	15.5766	38.1476	43.054	26.26	1.7694693	0.01479	0.03586
ENSRNOG00000008135	Pla2g4f	14.446886	20.7687	77.299	43.054	38.89	1.7713933	0.00438	0.0125
ENSRNOG00000049976	Gzmbl2	27.861852	20.7687	77.299	88.857	53.7	1.772879	0.00056	0.00199
ENSRNOG00000001171	Coq5	456.10884	411.221	1634.32	1331.9	958.4	1.7737154	1.6E-19	5.6E-18
ENSRNOG00000049985	Gprasp1	623.27995	673.946	2590.02	1849.5	1434	1.7747378	1.3E-18	4.2E-17
ENSRNOG00000020298	Bag3	7576.36	7708.32	33712.4	18622	16905	1.7756552	1.8E-15	4.1E-14
ENSRNOG00000016949	Slc25a33	211.54369	202.495	777.006	641.23	458.1	1.7758736	5.7E-15	1.2E-13
ENSRNOG00000007097	Gpatch3	169.23495	175.496	706.734	476.35	382	1.77834	1.1E-11	1.5E-10
ENSRNOG00000022414	Zfp142	1553.0403	1759.11	6895.68	4478.6	3672	1.7798192	3.7E-18	1.2E-16
ENSRNOG00000012864	Ern1	422.05547	443.413	1865.22	1107.5	959.5	1.7798354	1.3E-13	2.3E-12
ENSRNOG00000030988	AC136588.2	12.383045	7.26906	41.1592	26.565	21.84	1.7831172	0.02513	0.05677
ENSRNOG00000062605	ENSRNOG0000006260	158.91575	117.343	555.148	398.48	307.5	1.786657	1.9E-10	2.2E-09
ENSRNOG00000014373	Trim66	107.31973	101.767	234.909	487.34	232.8	1.7895033	2.5E-07	1.7E-06
ENSRNOG00000017728	Edrf1	670.7483	712.368	3104.01	1679.1	1542	1.7897598	2.8E-13	4.9E-12
ENSRNOG00000034150	AABR07041778.1	18.574568	35.3069	121.47	65.04	60.1	1.7901098	0.00093	0.00312
ENSRNOG00000065005	ENSRNOG0000006500	99.064364	92.4209	260.006	402.15	213.4	1.7908432	1.1E-08	9.9E-08
ENSRNOG00000068419	ENSRNOG0000006841	13.414966	17.6534	79.3068	28.398	34.69	1.7910276	0.0099	0.02534
ENSRNOG00000063293	Pdrg1	962.78178	728.983	3328.88	2540.2	1890	1.7944158	5.6E-19	1.9E-17
ENSRNOG00000014626	Katnb1	1123.7614	1074.78	4637.94	2997.3	2458	1.7959484	4.9E-18	1.5E-16
ENSRNOG00000001151	Sirt4	54.691784	66.46	211.819	208.86	135.5	1.7959819	1.5E-07	1.1E-06

ENSRNOG0000 0069578	Hist1h2bq	376.65097	401.875	1632.31	1074.5	871.3	1.7974022	2.4E-15	5.4E-14
ENSRNOG0000 0004458	Ston2	18.574568	17.6534	69.268	56.795	40.57	1.7982284	0.00198	0.00617
ENSRNOG0000 0006355	Akap8l	788.38723	805.827	3252.58	2295.6	1786	1.7989631	9.5E-20	3.4E-18
ENSRNOG0000 0011463	Psmb2	2345.5552	2071.68	8785.99	6588.2	4948	1.7992118	1.6E-23	8.2E-22
ENSRNOG0000 0003287	Trim16	1105.1868	1112.17	4533.54	3204.3	2489	1.8029391	6.9E-21	2.8E-19
ENSRNOG0000 0003950	Lvrn	24.766091	19.7303	91.3534	64.123	49.99	1.8036197	0.00082	0.00278
ENSRNOG0000 0061474	Ctdp1	1142.3359	1106.97	5145.91	2724.3	2530	1.806743	6.7E-14	1.3E-12
ENSRNOG0000 0010037	Epb41	1065.9738	976.131	3892.06	3258.4	2298	1.8078486	7.3E-26	4.9E-24
ENSRNOG0000 0054963	Rhbdd1	759.49345	715.483	2867.09	2314.9	1664	1.8126196	1.3E-23	6.8E-22
ENSRNOG0000 0015187	Acot8	330.21455	283.493	1249.84	908.72	693.1	1.8139924	6.3E-16	1.5E-14
ENSRNOG0000 0018325	Cnppd1	2305.3103	2310.52	8992.79	7242.3	5213	1.8143865	5.1E-28	4.5E-26
ENSRNOG0000 0017852	Nars1	5302.0073	5647.02	21439.9	17081	12367	1.8148004	1.6E-28	1.4E-26
ENSRNOG0000 0063843	ENSRNOG0 000006384	233.21402	174.457	791.06	644.9	460.9	1.8160811	5.5E-14	1.1E-12
ENSRNOG0000 0027204	Map6	2562.2585	2590.9	10867	7288.1	5827	1.8167714	5.4E-21	2.2E-19
ENSRNOG0000 0007645	Kcnj9	8.2553636	7.26906	32.1243	22.901	17.64	1.8242249	0.03518	0.07531
ENSRNOG0000 0004500	Myc	822.4406	857.749	2930.34	3020.2	1908	1.8244341	5.1E-29	4.8E-27
ENSRNOG0000 0013971	Psat1	3510.5934	3671.91	11761.5	13685	8157	1.8249354	6.4E-32	7.1E-30
ENSRNOG0000 0023318	Tigd3	15.478807	19.7303	88.3418	36.642	40.05	1.8254765	0.00442	0.01259
ENSRNOG0000 0006118	Klf10	488.09838	411.221	1613.24	1575.6	1022	1.8260488	1.7E-22	7.8E-21
ENSRNOG0000 0068106	Tcap	8.2553636	6.23062	22.0854	29.314	16.47	1.8279184	0.0411	0.08578
ENSRNOG0000 0003117	Psmd12	3130.8467	3000.04	12711.2	9101.9	6986	1.8309591	7.3E-24	4E-22
ENSRNOG0000 0063116	ENSRNOG0 000006311	142.40502	161.996	589.28	494.67	347.1	1.8319303	1.3E-13	2.4E-12

ENSRNOG00000019328	Phgdh	1428.1779	1278.32	4320.71	5333.2	3090	1.8347794	2.8E-26	2E-24
ENSRNOG00000026607	Tnfsf18	36.117216	29.0762	138.536	94.353	74.52	1.8355403	6E-05	0.00027
ENSRNOG00000005538	Psmd11	3306.2731	3448.65	13719.1	10393	7717	1.8357251	1.8E-26	1.3E-24
ENSRNOG00000002159	Gpat3	239.40555	264.801	1083.19	718.18	576.4	1.83651	4.3E-14	8.3E-13
ENSRNOG00000061595	Tfec	26.829932	16.615	81.3146	74.2	49.74	1.8391637	0.00066	0.00231
ENSRNOG00000007546	Asns	2062.809	2118.41	6562.39	8411.2	4789	1.8404982	4.1E-27	3.1E-25
ENSRNOG00000070937	ENSRNOG0000007093	16.510727	10.3844	56.2175	40.306	30.85	1.8420844	0.00643	0.01747
ENSRNOG00000035620	Mir22	40.244898	49.845	209.812	113.59	103.4	1.8425235	1.5E-05	7.6E-05
ENSRNOG00000062741	Ldhal6b	38.181057	21.8072	126.489	88.857	68.83	1.8425497	0.00015	0.00063
ENSRNOG00000008565	Nkiras1	84.617477	115.267	368.425	349.93	229.6	1.8455753	1.4E-10	1.7E-09
ENSRNOG00000067743	ENSRNOG0000006774	7.2234432	6.23062	30.1165	18.321	15.47	1.8461896	0.047	0.09585
ENSRNOG00000018752	ENSRNOG0000001875	275.52276	302.185	1187.59	892.23	664.4	1.8476853	4E-17	1.1E-15
ENSRNOG00000017965	Afg3l2	1175.3574	1272.09	4727.29	4095.7	2818	1.8499158	3.6E-29	3.4E-27
ENSRNOG00000008604	Nsfl1c	3013.2077	2996.93	12206.2	9480.2	6924	1.8512673	5.2E-28	4.6E-26
ENSRNOG00000055598	Mir568	7.2234432	13.4997	39.1515	35.726	23.9	1.8534031	0.01468	0.03562
ENSRNOG00000013713	ENSRNOG0000001371	10.319205	6.23062	38.1476	21.985	19.17	1.8591078	0.02929	0.06456
ENSRNOG00000022392	Hspb8	91.84092	89.3056	431.67	226.26	209.8	1.8596304	6.5E-08	5E-07
ENSRNOG00000015095	Spryd7	198.12873	182.765	762.952	622.91	441.7	1.862903	9.6E-16	2.2E-14
ENSRNOG00000070677	ENSRNOG0000007067	5.1596023	20.7687	56.2175	38.474	30.15	1.8683842	0.01226	0.03056
ENSRNOG00000032959	Adh7	71.202511	40.499	168.652	239.09	129.9	1.8685333	1.5E-06	8.8E-06
ENSRNOG00000050834	Fam102a	275.52276	326.069	1203.66	995.75	700.2	1.8700401	6.7E-19	2.3E-17
ENSRNOG00000052573	Snord17	5.1596023	9.34593	31.1204	21.985	16.9	1.8714518	0.03695	0.07848

ENSRNOG00000016575	Tnfrsf1b	5.1596023	9.34593	32.1243	21.069	16.92	1.873589	0.03728	0.07912
ENSRNOG00000055984	Adrm1	3264.9963	3146.46	13334.6	10176	7481	1.8745559	7.4E-28	6.3E-26
ENSRNOG00000018824	Slc7a5	4438.2899	4460.09	17171.4	15479	10387	1.8754867	1.8E-36	2.7E-34
ENSRNOG00000018166	Prkab2	269.33124	293.878	1173.54	895.9	658.2	1.8771433	7.6E-18	2.3E-16
ENSRNOG00000018809	Psmd5	2233.0759	2192.14	9361.21	6902.4	5172	1.8777518	7.3E-26	4.9E-24
ENSRNOG00000064485	ENSRNOG0000006448	87.713239	126.689	422.635	366.42	250.9	1.8796703	6.3E-11	7.9E-10
ENSRNOG00000015290	Tpi1	8410.1517	8262.84	29520.2	32041	19558	1.8845079	1.6E-38	2.7E-36
ENSRNOG00000014387	Chac1	577.87545	543.103	1878.27	2265.4	1316	1.8863149	9.9E-25	6E-23
ENSRNOG00000018183	Ubr4	4950.1224	5302.26	19650	18276	12045	1.8872239	2.4E-37	3.9E-35
ENSRNOG00000055506	ENSRNOG0000005550	7.2234432	6.23062	36.1398	13.741	15.83	1.8873171	0.04792	0.09747
ENSRNOG00000063969	ENSRNOG0000006396	16.510727	9.34593	60.233	35.726	30.45	1.8898381	0.0067	0.01808
ENSRNOG00000064025	ENSRNOG0000006402	13.414966	10.3844	45.1748	43.054	28.01	1.889969	0.00648	0.01757
ENSRNOG00000019868	Psma5	2494.1517	2325.06	10696.4	7231.3	5687	1.8952383	9.7E-23	4.7E-21
ENSRNOG00000024809	Ntmt1	895.70695	736.252	3870.98	2209.5	1928	1.8973473	1.3E-15	2.9E-14
ENSRNOG00000021318	Epas1	7.2234432	7.26906	34.132	20.153	17.19	1.9034046	0.03216	0.06983
ENSRNOG00000010799	Noct	206.38409	187.957	933.612	544.13	468	1.9051826	2.9E-12	4.4E-11
ENSRNOG00000002046	Cd200r1l	20.638409	26.9994	60.233	119.09	56.74	1.914372	0.00042	0.00154
ENSRNOG00000007203	Psmc6	3225.7833	3157.89	13892.7	10213	7622	1.9168662	2.3E-27	1.7E-25
ENSRNOG00000005049	Tbr1	9.2872841	8.3075	49.1903	17.405	21.05	1.9172023	0.02443	0.05546
ENSRNOG00000031031	Zfp292	868.87702	808.943	3406.18	2935	2005	1.9180447	2.8E-29	2.7E-27
ENSRNOG00000068687	Ubxn2a	778.06802	713.406	3497.53	2140.8	1782	1.918287	6.5E-18	2E-16
ENSRNOG00000036697	Mafg	2175.2883	2165.14	9710.57	6714.6	5191	1.9199187	2.9E-24	1.6E-22

ENSRNOG00000010421	Wdr91	407.60858	410.183	1638.34	1466.6	980.7	1.9246144	2.8E-25	1.8E-23
ENSRNOG00000027480	Tmem116	20.638409	32.1916	105.408	95.269	63.38	1.9254267	7.6E-05	0.00033
ENSRNOG00000058681	Tsga10	14.446886	21.8072	80.3107	57.711	43.57	1.9278954	0.00089	0.00302
ENSRNOG00000010345	Ssmem1	65.010989	57.114	227.882	237.26	146.8	1.9293356	5.5E-09	5.1E-08
ENSRNOG00000065753	ENSRNOG0000006575	8.2553636	8.3075	53.2058	10.077	19.96	1.930194	0.03931	0.08264
ENSRNOG00000010176	Map2k1	2800.6321	2698.9	11785.6	9231.9	6629	1.9341567	1.3E-30	1.3E-28
ENSRNOG00000019525	Hspa9	7714.6373	7788.28	32875.2	26410	18697	1.9351061	1.6E-33	2E-31
ENSRNOG00000004006	Dnajb9	708.92935	684.33	3403.17	1931.9	1682	1.936799	1.8E-16	4.6E-15
ENSRNOG00000028120	AABR07011698.1	33.021455	22.8456	111.431	102.6	67.47	1.9372126	3.7E-05	0.00017
ENSRNOG00000007372	Casp1	11.351125	3.11531	27.1049	28.398	17.49	1.9391832	0.03412	0.07339
ENSRNOG00000030160	Zfp819	41.276818	56.0756	241.936	133.74	118.3	1.9470368	2E-06	1.2E-05
ENSRNOG00000007964	Tp53inp1	444.75772	445.49	2027.84	1416.2	1084	1.9515135	2.5E-20	9.5E-19
ENSRNOG00000067060	MGC105649	24.766091	23.8841	103.4	85.193	59.31	1.9540841	7.3E-05	0.00032
ENSRNOG00000007850	Mok	62.947148	65.4215	322.247	175.88	156.6	1.9549851	1.2E-07	9.1E-07
ENSRNOG00000023026	Fbxo48	18.574568	4.15375	33.1282	54.963	27.7	1.9550722	0.01289	0.0319
ENSRNOG00000048004	Garem2	9.2872841	3.11531	27.1049	21.069	15.14	1.9560221	0.04312	0.0894
ENSRNOG00000060849	Rcor2	177.49032	147.458	524.027	742.92	398	1.9635721	3.4E-14	6.6E-13
ENSRNOG00000020811	Il6r	210.51177	148.497	801.099	600.93	440.3	1.9648583	2.4E-14	4.8E-13
ENSRNOG00000012736	Znrd2	809.02564	815.173	3777.61	2575	1994	1.9674285	1.8E-22	8.3E-21
ENSRNOG00000053811	Arg2	21.67033	18.6919	89.3456	68.704	49.6	1.968315	0.00026	0.00099
ENSRNOG00000006589	Mif	2819.2067	2666.71	10312.9	11196	6749	1.971145	3.9E-39	7E-37
ENSRNOG00000009754	Nampt	1306.4113	1252.36	5045.52	5008	3153	1.9741754	2.7E-37	4.3E-35

ENSRNOG00000033153	Sfn	14.446886	34.2684	120.466	71.452	60.16	1.9772207	0.00033	0.00123
ENSRNOG00000019697	Ankrd28	1083.5165	1150.59	4625.9	4189.1	2762	1.9802129	8.3E-35	1.1E-32
ENSRNOG00000019822	Gadd45b	15432.37	14077.1	77503.8	38975	36497	1.9808086	1.5E-16	3.7E-15
ENSRNOG00000046502	Lonp1	3621.0089	3434.11	14001.2	13850	8727	1.9809763	8.6E-42	1.7E-39
ENSRNOG00000001490	Pdcd2	390.06593	333.338	1579.11	1279.7	895.6	1.9822532	1.4E-22	6.9E-21
ENSRNOG00000020975	Mrpl49	1185.6766	1095.55	5060.58	4027.9	2842	1.994102	4.1E-30	4.2E-28
ENSRNOG00000015109	Ubxn8	287.90581	322.954	1481.73	960.02	763.2	1.9986002	2.7E-17	7.5E-16
ENSRNOG00000023376	Riok3	1996.7661	2119.45	9516.82	6948.2	5145	1.9999434	2.3E-28	2E-26
ENSRNOG00000013018	ENSRNOG0000001301	92.872841	104.882	399.546	392.07	247.3	2.0011096	1.7E-13	3.1E-12
ENSRNOG00000001517	Pdk1	682.09942	589.832	2189.47	2905.7	1592	2.0022809	1.1E-24	6.7E-23
ENSRNOG00000024878	Rbm44	150.66039	161.996	618.392	637.57	392.2	2.0062476	4.7E-18	1.5E-16
ENSRNOG00000059579	Gpt2	1206.315	1222.24	4641.96	5145.4	3054	2.0108869	8.5E-37	1.3E-34
ENSRNOG00000068164	ENSRNOG0000006816	8.2553636	6.23062	52.2019	6.4123	18.28	2.0126189	0.04847	0.09842
ENSRNOG00000013484	Gsta1	842.04709	780.905	2710.49	3842.8	2044	2.0137767	1.6E-24	9.6E-23
ENSRNOG00000010736	Pbx4	12.383045	1.03844	24.0932	30.23	16.94	2.0165462	0.04454	0.0917
ENSRNOG00000028688	Ctns	351.88488	315.685	1361.27	1359.4	847.1	2.0269356	1.8E-26	1.3E-24
ENSRNOG00000007152	Bhlhe40	2431.2046	2550.4	10158.3	10158	6324	2.027964	9.7E-43	2.2E-40
ENSRNOG00000042607	Rhof	62.947148	55.0372	322.247	160.31	150.1	2.0306963	1.8E-07	1.3E-06
ENSRNOG00000014915	Gsr	1501.4443	1405.01	5891.79	6043.2	3710	2.0378681	5.4E-40	1E-37
ENSRNOG00000013009	Ldha	24444.132	23360.7	100672	96047	61131	2.0409089	1.4E-46	4.3E-44
ENSRNOG00000058249	ENSRNOG0000005824	17729.425	17541.3	75106.6	70484	45215	2.0453765	1.2E-46	3.8E-44
ENSRNOG00000022764	Evi2a	61.915227	35.3069	240.932	163.06	125.3	2.0537507	2.4E-07	1.7E-06

ENSRNOG00000011944	Snx10	660.42909	669.792	3111.04	2413.8	1714	2.0540836	1.5E-28	1.3E-26
ENSRNOG00000037375	RGD1565410	8.2553636	6.23062	51.1981	9.1605	18.71	2.0550507	0.03476	0.07455
ENSRNOG00000011112	Fam161b	15.478807	26.9994	81.3146	95.269	54.77	2.0564429	1E-04	0.00042
ENSRNOG00000043486	Tnfrsf26	88.745159	68.5369	425.647	232.68	203.9	2.0642609	3.2E-09	3.1E-08
ENSRNOG00000019914	Tlcd3b	5.1596023	8.3075	38.1476	18.321	17.48	2.066027	0.02315	0.05298
ENSRNOG00000016760	Kctd19	18.574568	43.6144	119.462	141.07	80.68	2.0676639	1.4E-05	6.8E-05
ENSRNOG00000021578	Wfikkn1	44.37258	37.3837	247.959	96.185	106.5	2.0718297	1E-05	5.2E-05
ENSRNOG00000010134	Acot2	350.85295	389.414	1464.67	1658	965.7	2.0768745	7.7E-28	6.5E-26
ENSRNOG00000008548	ENSRNOG0000000854	3.0957614	10.3844	35.1359	21.985	17.65	2.082529	0.02408	0.05485
ENSRNOG00000037580	Psmd8	5479.4976	5205.69	27198.2	18212	14024	2.0873717	7E-28	6.1E-26
ENSRNOG00000019296	Gnat2	10.319205	16.615	40.1553	74.2	35.32	2.0881665	0.00164	0.00522
ENSRNOG00000037076	ENSRNOG0000003707	23.73417	31.1531	125.485	108.09	72.12	2.0890985	5E-06	2.7E-05
ENSRNOG00000022738	AC098459.1	66.042909	87.2287	345.336	306.88	201.4	2.0891282	5.5E-12	8.1E-11
ENSRNOG00000015354	Aox1	106.28781	152.65	470.821	632.07	340.5	2.0912828	3.5E-14	6.8E-13
ENSRNOG00000000569	Vsir	6.1915227	7.26906	19.0738	38.474	17.75	2.0984164	0.0193	0.0452
ENSRNOG00000054561	Isg20	209.47985	280.378	1325.13	783.22	649.6	2.105278	2.4E-15	5.4E-14
ENSRNOG00000013141	Eno2	1385.8692	1360.35	5468.15	6366.5	3645	2.1075669	4.8E-39	8.5E-37
ENSRNOG00000000925	Psph	641.85452	606.447	2661.3	2719.8	1657	2.1079247	2.4E-36	3.3E-34
ENSRNOG00000007439	Unc13d	19.606489	16.615	93.3612	64.123	48.43	2.1189782	0.00014	0.00056
ENSRNOG00000009329	Nr1d1	1156.7828	1228.47	5997.2	4367.7	3188	2.1193835	4E-30	4.1E-28
ENSRNOG00000015271	Zfp418	239.40555	262.725	1318.1	870.25	672.6	2.1232759	5.1E-19	1.7E-17
ENSRNOG00000026928	RGD1560402	33.021455	38.4222	169.656	141.99	95.77	2.1245927	1.4E-07	1E-06

ENSRNOG00000065186	ENSRNOG0000006518	6.1915227	5.19219	35.1359	14.657	15.29	2.1260954	0.02909	0.06419
ENSRNOG00000001582	Bach1	1158.8467	1163.05	5771.33	4367.7	3115	2.1264332	2.2E-32	2.5E-30
ENSRNOG00000036677	Slc16a3	11.351125	13.4997	59.2291	49.467	33.39	2.1284518	0.00103	0.00344
ENSRNOG00000020729	Stc2	1412.6991	1238.86	5831.56	5794.9	3570	2.1324834	3.8E-41	7.4E-39
ENSRNOG00000017429	Lat	18.574568	13.4997	80.3107	60.459	43.21	2.1326848	0.00025	0.00098
ENSRNOG00000015052	Star	24.766091	31.1531	170.66	75.116	75.42	2.1342356	2.7E-05	0.00013
ENSRNOG00000071141	ENSRNOG0000007114	25.798011	37.3837	150.583	127.33	85.27	2.1368236	7.4E-07	4.7E-06
ENSRNOG00000028357	Lrrc14b	26.829932	28.0378	135.524	106.26	74.16	2.1389756	2.3E-06	1.4E-05
ENSRNOG00000036664	Narf	403.4809	400.837	1949.54	1616.8	1093	2.1484118	7.1E-30	7E-28
ENSRNOG00000009734	Akr1b10	655.26949	646.946	2832.96	2940.5	1769	2.1485014	1.4E-38	2.5E-36
ENSRNOG00000058497	Fdxr	185.74568	145.381	845.27	623.83	450.1	2.148902	1.1E-17	3.2E-16
ENSRNOG00000024578	Ttyh2	318.86342	346.838	1773.86	1183.5	905.8	2.151036	1.5E-21	6.5E-20
ENSRNOG00000037415	AABR07002938.1	10.319205	6.23062	35.1359	38.474	22.54	2.1529957	0.00626	0.01708
ENSRNOG00000023150	Gpi	6320.5128	6077.97	29722	25673	16948	2.1595802	7.7E-46	2.2E-43
ENSRNOG00000001214	Pfkl	2316.6614	2182.79	9380.29	10743	6156	2.161106	1.2E-43	3.1E-41
ENSRNOG00000068243	Cdk5r1	40.244898	43.6144	285.103	93.437	115.6	2.1727806	6.8E-06	3.6E-05
ENSRNOG00000012108	Thsd1	7.2234432	5.19219	34.132	21.985	17.13	2.1745113	0.01602	0.03839
ENSRNOG00000017073	Ca9	166.13919	239.879	730.827	1110.3	561.8	2.1815081	1.2E-16	3E-15
ENSRNOG00000019458	Shpk	28.893773	31.1531	152.59	120	83.16	2.1819115	4.5E-07	2.9E-06
ENSRNOG00000020279	Syt11	813.15332	793.366	4417.09	2892.9	2229	2.1857442	4.7E-26	3.2E-24
ENSRNOG00000050647	ENSRNOG0000005064	24.766091	36.3453	231.897	46.719	84.93	2.1870294	0.00028	0.00106
ENSRNOG00000067652	ENSRNOG0000006765	22.70225	30.1147	156.606	84.277	73.42	2.1878756	8.8E-06	4.6E-05

ENSRNOG00000045844	Impact	2013.2768	2009.38	11643	6748.5	5604	2.1927458	1.3E-23	6.8E-22
ENSRNOG00000001873	P2rx6	7.2234432	9.34593	29.1126	46.719	23.1	2.1960404	0.0053	0.01477
ENSRNOG00000000321	Cd24	6.1915227	3.11531	16.0621	26.565	12.98	2.1965676	0.0349	0.07479
ENSRNOG00000027433	Akr1b8	14439.663	14308.6	61878.4	70413	40260	2.2021775	4.9E-50	1.6E-47
ENSRNOG00000067231	ENSRNOG0000006723	134.14966	159.919	920.561	436.04	412.7	2.2050879	3.2E-12	4.9E-11
ENSRNOG00000018494	Ppp1r3c	88.745159	99.69	395.53	473.6	264.4	2.2059862	7.6E-16	1.8E-14
ENSRNOG00000025327	Tert	14.446886	10.3844	77.299	37.558	34.92	2.2073099	0.00109	0.00361
ENSRNOG00000048682	Zwint	2077.2559	2133.99	12672	6863	5937	2.2136727	7.7E-22	3.4E-20
ENSRNOG00000009088	Txnrd1	7201.7729	7258.68	37249.1	29930	20410	2.2158855	1.7E-43	4.1E-41
ENSRNOG00000011873	Atg14	273.45892	285.57	1635.33	973.76	792	2.2221211	2.9E-19	1E-17
ENSRNOG00000065190	ENSRNOG0000006519	10.319205	37.3837	146.567	76.948	67.8	2.2276167	0.00012	0.00049
ENSRNOG00000026112	Tmem202	22.70225	16.615	96.3728	88.857	56.14	2.2355703	1.4E-05	7.1E-05
ENSRNOG00000069906	ENSRNOG0000006990	4.1276818	4.15375	17.066	21.985	11.83	2.2383277	0.03645	0.07764
ENSRNOG00000070930	Hilpda	340.53375	319.839	1669.46	1447.4	944.3	2.2385206	1.4E-31	1.5E-29
ENSRNOG00000006304	Mdm2	3052.4207	3144.39	16523.9	12870	8898	2.2459065	5.9E-41	1.1E-38
ENSRNOG00000011007	Ube2o	2278.4804	2122.57	12908.9	8020	6332	2.2495089	1.7E-27	1.4E-25
ENSRNOG00000015674	Acap1	10.319205	13.4997	61.2369	52.215	34.32	2.2515877	0.00049	0.00179
ENSRNOG00000007415	Ptgs1	130.02198	105.921	420.627	702.61	339.8	2.2518083	2E-14	4E-13
ENSRNOG00000051816	Tigar	1514.8592	1334.39	7802.18	5820.6	4118	2.2572659	4.7E-35	6.3E-33
ENSRNOG00000007793	Pnrc1	222.89482	190.034	1259.87	727.34	600	2.2662023	1.9E-17	5.4E-16
ENSRNOG00000002343	Uchl1	70.170591	77.8828	413.6	301.38	215.8	2.2711422	1.1E-13	2E-12
ENSRNOG00000010883	Pard6b	155.81999	170.304	838.243	741.08	476.4	2.2756388	4.3E-24	2.4E-22

ENSRNOG00000013279	ENSRNOG0000001327	6.1915227	5.19219	38.1476	17.405	16.73	2.2842899	0.0149	0.03607
ENSRNOG00000051405	AABR07021465.1	11.351125	10.3844	71.2757	34.81	31.96	2.2849478	0.00111	0.00366
ENSRNOG00000042691	Armc7	222.89482	192.111	973.767	1053.5	610.6	2.2883577	1.8E-27	1.4E-25
ENSRNOG00000021255	Smox	17.542648	15.5766	92.3573	69.62	48.77	2.2890493	3.5E-05	0.00016
ENSRNOG00000059163	AABR07069454.1	9.2872841	11.4228	49.1903	52.215	30.53	2.2921056	0.00077	0.00265
ENSRNOG00000008297	Oser1	1548.9126	1381.12	9418.44	5099.6	4362	2.3087553	6.7E-23	3.3E-21
ENSRNOG00000017163	Pfkp	1685.1261	1854.65	8983.75	8737.3	5315	2.3237353	2.2E-52	8.9E-50
ENSRNOG00000066847	Gnrh1	62.947148	102.805	577.233	253.75	249.2	2.3250313	7.6E-10	8.1E-09
ENSRNOG00000065046	ENSRNOG0000006504	2.0638409	6.23062	25.0971	16.489	12.47	2.3253612	0.03154	0.06873
ENSRNOG00000066874	ENSRNOG0000006687	7.2234432	10.3844	48.1864	40.306	26.53	2.3290497	0.00154	0.00492
ENSRNOG00000067259	ENSRNOG0000006725	2.0638409	9.34593	38.1476	19.237	17.2	2.32947	0.01653	0.03948
ENSRNOG00000042785	Sesn2	1202.1873	1260.66	7210.9	5228.8	3726	2.3364562	1.4E-36	2.1E-34
ENSRNOG00000015499	Serinc4	6.1915227	2.07687	28.1087	13.741	12.53	2.3364903	0.03269	0.07078
ENSRNOG00000019598	Vegfa	2632.4291	2814.16	14090.5	13429	8242	2.3370383	7.5E-56	3.5E-53
ENSRNOG00000071131	ENSRNOG0000007113	431.34275	522.334	2687.4	2138.1	1445	2.3389697	2.9E-33	3.5E-31
ENSRNOG00000005964	Nr4a3	137.24542	132.92	704.726	662.3	409.3	2.3389843	6.3E-24	3.4E-22
ENSRNOG00000053889	Celsr3	2.0638409	5.19219	22.0854	14.657	11	2.3395627	0.03991	0.08363
ENSRNOG00000063033	ENSRNOG0000006303	9.2872841	3.11531	42.1631	21.069	18.91	2.3472057	0.01025	0.02609
ENSRNOG00000061507	AABR07032328.1	5.1596023	2.07687	25.0971	11.909	11.06	2.3513058	0.04127	0.08604
ENSRNOG00000062373	ENSRNOG0000006237	7.2234432	3.11531	29.1126	23.817	15.82	2.3546536	0.01371	0.03355
ENSRNOG00000013692	Abhd18	66.042909	58.1525	315.219	322.45	190.5	2.3601619	9.8E-15	2E-13
ENSRNOG00000030264	Fam83h	2.0638409	6.23062	15.0583	27.481	12.71	2.3613296	0.02917	0.06436

ENSRNOG00000047028	Mmp24	0	12.4612	44.1709	20.153	19.2	2.367581	0.02486	0.05626
ENSRNOG00000051680	Ccdc186	2042.1706	2109.07	13701	7980.6	6458	2.3847932	5.8E-28	5E-26
ENSRNOG00000067089	ENSRNOG0000006708	13.414966	0	39.1515	31.146	20.93	2.3878411	0.01691	0.04028
ENSRNOG00000067752	ENSRNOG0000006775	4.1276818	8.3075	37.1437	28.398	19.49	2.3976825	0.00564	0.0156
ENSRNOG00000018505	Cidea	6.1915227	5.19219	38.1476	21.985	17.88	2.399229	0.00783	0.02067
ENSRNOG00000064682	ENSRNOG0000006468	1.0319205	8.3075	30.1165	19.237	14.67	2.401725	0.02305	0.0528
ENSRNOG00000021800	Zfp541	4.1276818	7.26906	41.1592	19.237	17.95	2.4040193	0.00913	0.02363
ENSRNOG00000071119	ENSRNOG0000007111	887.45159	840.096	5916.89	3270.3	2729	2.4107384	6.7E-25	4.1E-23
ENSRNOG00000003334	Klhl21	1549.9445	1434.08	9596.12	6284.1	4716	2.4118052	2.3E-33	2.7E-31
ENSRNOG00000021735	Akr1c15	3.0957614	3.11531	11.0427	21.985	9.81	2.4130541	0.04467	0.0919
ENSRNOG00000002255	Fam162a	978.26059	1060.24	5122.82	5744.5	3226	2.4144879	2E-50	6.8E-48
ENSRNOG00000003233	Serpinf2	9.2872841	2.07687	38.1476	22.901	18.1	2.4229603	0.01029	0.02618
ENSRNOG00000052038	Haus8	494.2899	466.258	3085.94	2073.9	1530	2.4251577	1.3E-30	1.3E-28
ENSRNOG00000019587	Ptprn	137.24542	100.728	723.8	555.13	379.2	2.4254569	4E-20	1.5E-18
ENSRNOG00000019559	Eaf1	1748.0733	1767.42	12400	6613.9	5632	2.435174	1.9E-25	1.2E-23
ENSRNOG00000036877	Clca4l	4.1276818	3.11531	22.0854	17.405	11.68	2.4457469	0.02542	0.05729
ENSRNOG00000063044	ENSRNOG0000006304	110.41549	94.4978	594.299	523.06	330.6	2.4466462	9.6E-22	4.2E-20
ENSRNOG00000015113	Mocos	196.06489	176.534	1180.57	855.59	602.2	2.4496875	7.1E-26	4.7E-24
ENSRNOG00000020552	Fosl1	1320.8582	1437.2	9738.67	5417.5	4479	2.4580937	6.4E-27	4.7E-25
ENSRNOG00000013123	Scube2	5.1596023	23.8841	85.3301	74.2	47.14	2.4581217	0.00013	0.00054
ENSRNOG00000006789	Ddit3	441.66195	469.374	3244.55	1767.1	1481	2.4594415	1.8E-23	9.5E-22
ENSRNOG00000007587	Tcp11l2	189.87336	253.379	1427.52	1013.2	721	2.4608348	4.2E-25	2.6E-23

ENSRNOG00000063481	1700092M07Rik	4.1276818	4.15375	18.0699	27.481	13.46	2.46097	0.01663	0.03969
ENSRNOG00000069730	ENSRNOG0000006973	1.0319205	6.23062	21.0816	19.237	11.9	2.4738646	0.02953	0.065
ENSRNOG00000060430	Y_RNA	5.1596023	7.26906	60.233	9.1605	20.46	2.4779429	0.01159	0.02907
ENSRNOG00000067619	ENSRNOG0000006761	10.319205	17.6534	99.3845	56.795	46.04	2.4800144	4.1E-05	0.00019
ENSRNOG00000036703	Itgax	165.10727	150.573	1013.92	754.82	521.1	2.4857113	1E-25	6.6E-24
ENSRNOG00000049221	Kdm4b	879.19623	897.21	5190.08	4774.4	2935	2.4877989	3.6E-55	1.5E-52
ENSRNOG00000010348	Cacna1f	3.0957614	6.23062	12.0466	40.306	15.42	2.4925433	0.01573	0.0378
ENSRNOG00000052730	Psmb3	2450.8111	2152.68	15064.3	10913	7645	2.4963873	3.6E-42	7.6E-40
ENSRNOG00000071190	Pspn	43.340659	67.4984	515.996	109.93	184.2	2.4972966	0.01663	0.03969
ENSRNOG00000069152	ENSRNOG0000006915	39.212977	46.7297	359.39	127.33	143.2	2.5003285	3.2E-08	2.7E-07
ENSRNOG00000070359	ENSRNOG0000007035	6.1915227	5.19219	35.1359	29.314	18.96	2.5004144	0.00404	0.01164
ENSRNOG00000016791	Chka	459.2046	491.181	3583.86	1801.9	1584	2.5023195	4.1E-22	1.9E-20
ENSRNOG00000011228	Layn	17.542648	21.8072	131.509	91.605	65.62	2.5024885	5.1E-07	3.4E-06
ENSRNOG00000020812	Gys1	1197.0277	1173.43	5717.12	7791.9	3970	2.5107744	3.8E-43	8.7E-41
ENSRNOG00000038047	Mt1	2.0638409	4.15375	29.1126	6.4123	10.44	2.5112128	0.04406	0.09099
ENSRNOG00000069549	ENSRNOG0000006954	20.638409	5.19219	129.501	18.321	43.41	2.5138813	0.00223	0.00686
ENSRNOG00000064137	ENSRNOG0000006413	1.0319205	6.23062	29.1126	12.825	12.3	2.5286382	0.0281	0.06244
ENSRNOG00000028113	Whamm	635.663	613.716	4405.04	2806.8	2115	2.5289613	2.1E-32	2.4E-30
ENSRNOG00000009639	Zrsr1	686.2271	748.713	4756.4	3541.4	2433	2.5316259	2E-41	4.1E-39
ENSRNOG00000017706	Slc66a1	190.90528	158.881	1257.87	767.65	593.8	2.5331744	3.1E-22	1.4E-20
ENSRNOG00000024631	Chadl	2.0638409	3.11531	19.0738	10.993	8.811	2.535891	0.04635	0.09486
ENSRNOG00000058522	Fam214a	331.24647	374.876	2560.91	1539	1201	2.5373175	8.2E-27	5.9E-25

ENSRNOG00000069365	ENSRNOG0000006936	24.766091	36.3453	183.711	171.3	104	2.5384745	3.3E-10	3.7E-09
ENSRNOG00000015382	Arid5a	102.16013	95.5362	811.138	338.94	336.9	2.5395117	3.7E-13	6.3E-12
ENSRNOG00000008364	Cat	2512.7263	2637.63	13683.9	16440	8819	2.5482179	1.2E-57	6.3E-55
ENSRNOG00000033570	Arhgap8	5.1596023	1.03844	18.0699	18.321	10.65	2.5525103	0.0321	0.06971
ENSRNOG00000019824	Ell	579.9393	577.371	3942.25	2858.1	1989	2.5546548	9.5E-40	1.7E-37
ENSRNOG00000065859	SNORD63	3.0957614	4.15375	29.1126	13.741	12.53	2.5613675	0.01884	0.04431
ENSRNOG00000013591	ENSRNOG0000001359	29.925693	20.7687	173.672	127.33	87.92	2.5687396	7.1E-09	6.5E-08
ENSRNOG00000047682	AABR07065031.1	12.383045	10.3844	78.3029	57.711	39.7	2.5775935	3.4E-05	0.00016
ENSRNOG00000019428	Higd1a	1023.6651	963.67	6194.97	5700.6	3471	2.5814642	4.6E-60	2.8E-57
ENSRNOG00000071065	ENSRNOG0000007106	154.78807	177.573	1384.36	611.92	582.2	2.5859723	1.2E-16	3E-15
ENSRNOG00000053404	Ppcdc	1.0319205	6.23062	28.1087	15.573	12.74	2.588038	0.02138	0.04947
ENSRNOG00000013240	Ptger4	2.0638409	5.19219	28.1087	15.573	12.73	2.5887277	0.01758	0.04172
ENSRNOG00000023991	Rab20	142.40502	131.882	1041.03	637.57	488.2	2.6128872	2E-22	9.5E-21
ENSRNOG00000018262	Ampd3	20.638409	23.8841	147.571	125.5	79.4	2.6163134	6.6E-09	6E-08
ENSRNOG00000021161	Fermt3	13.414966	16.615	146.567	39.39	54	2.6283764	5.3E-05	0.00024
ENSRNOG00000018487	Slc3a2	4492.9817	4466.32	31227.8	24245	16108	2.6303028	1.4E-56	6.7E-54
ENSRNOG00000021693	Fam149a	3.0957614	5.19219	32.1243	19.237	14.91	2.6304341	0.00868	0.02264
ENSRNOG00000043378	Nkpd1	7.2234432	3.11531	37.1437	27.481	18.74	2.6423194	0.00344	0.01008
ENSRNOG00000034106	AC127887.1	4.1276818	7.26906	39.1515	32.062	20.65	2.6434028	0.00192	0.006
ENSRNOG00000010262	Hdc	5.1596023	6.23062	42.1631	29.314	20.72	2.6486733	0.00182	0.00573
ENSRNOG00000017332	Dapk2	3.0957614	2.07687	15.0583	17.405	9.409	2.6499364	0.03178	0.06915
ENSRNOG00000015677	Cda	50.564102	43.6144	308.192	283.06	171.4	2.6499604	3.6E-16	8.7E-15

ENSRNOG00000069023	ENSRNOG0000006902	8.2553636	11.4228	86.334	37.558	35.89	2.6525561	0.00014	0.00059
ENSRNOG00000069282	ENSRNOG0000006928	6.1915227	5.19219	59.2291	12.825	20.86	2.6587319	0.00493	0.01386
ENSRNOG00000063189	ENSRNOG0000006318	4.1276818	6.23062	28.1087	37.558	19.01	2.6657226	0.00263	0.00793
ENSRNOG00000033206	Entpd5	1047.3993	1062.32	6381.69	7129.6	3905	2.6790904	7.8E-65	6.1E-62
ENSRNOG00000069130	ENSRNOG0000006913	10.319205	9.34593	107.416	19.237	36.58	2.6843885	0.00073	0.0025
ENSRNOG00000052880	Prph	3.0957614	2.07687	25.0971	8.2444	9.629	2.6847704	0.03441	0.07391
ENSRNOG00000050426	Dnah3	42.308739	68.5369	299.157	414.05	206	2.6867229	1.2E-15	2.7E-14
ENSRNOG00000033581	AABR07044388.2	13.414966	4.15375	59.2291	54.047	32.71	2.6875514	0.00018	0.0007
ENSRNOG00000017434	Mgat3	36.117216	41.5375	227.882	273.9	144.9	2.6925086	2.2E-14	4.5E-13
ENSRNOG00000063502	U6	1.0319205	3.11531	16.0621	10.993	7.8	2.7054084	0.04949	0.10016
ENSRNOG00000000201	Gsta5	46.43642	30.1147	190.738	308.71	144	2.7065017	3.7E-12	5.6E-11
ENSRNOG00000066992	ENSRNOG0000006699	7.2234432	6.23062	53.2058	35.726	25.6	2.7232509	0.00044	0.00162
ENSRNOG00000019161	Cpeb1	8.2553636	14.5381	100.388	51.299	43.62	2.7331668	1.9E-05	9.4E-05
ENSRNOG00000014061	Dusp5	384.90633	387.337	3368.03	1769.8	1478	2.7337755	3.2E-27	2.5E-25
ENSRNOG00000013564	Dok3	8.2553636	10.3844	91.3534	32.978	35.74	2.735554	0.00015	0.00061
ENSRNOG00000069723	ENSRNOG0000006972	3.0957614	2.07687	19.0738	15.573	9.955	2.7426393	0.02362	0.05392
ENSRNOG00000020706	Kcnn3	4.1276818	4.15375	28.1087	27.481	15.97	2.7468078	0.00427	0.01221
ENSRNOG00000018716	Dennd2c	77.394034	107.997	797.084	451.61	358.5	2.7512582	6.2E-19	2.1E-17
ENSRNOG00000026605	Ifi27l2b	198.12873	148.497	1094.23	1244.9	671.4	2.7545499	1.7E-35	2.3E-33
ENSRNOG00000007866	Clec2dl1	91.84092	99.69	705.73	587.19	371.1	2.7546903	7.2E-28	6.2E-26
ENSRNOG00000036856	AC097183.1	2.0638409	2.07687	16.0621	11.909	8.028	2.7549394	0.04043	0.08457
ENSRNOG00000015678	Spata1	8.2553636	11.4228	87.3379	45.802	38.2	2.7568129	3.6E-05	0.00017

ENSRNOG00000007044	L3mbtl1	2.0638409	2.07687	17.066	10.993	8.05	2.7590017	0.04052	0.08472
ENSRNOG00000052293	SNORD15	1.0319205	4.15375	27.1049	8.2444	10.13	2.7670953	0.03069	0.06719
ENSRNOG00000023449	Atg9b	10.319205	14.5381	84.3262	85.193	48.59	2.7700674	1.2E-06	7.5E-06
ENSRNOG00000031335	Ankrd37	33.021455	26.9994	245.951	164.89	117.7	2.7739083	5.8E-12	8.5E-11
ENSRNOG00000026323	Eqtn	8.2553636	26.9994	151.586	90.689	69.38	2.7805109	6.5E-07	4.2E-06
ENSRNOG00000000158	Cdo1	2.0638409	4.15375	42.1631	0.916	12.32	2.789359	0.04292	0.08904
ENSRNOG00000017899	Akr7a3	4.1276818	2.07687	11.0427	32.062	12.33	2.798711	0.01484	0.03595
ENSRNOG00000049955	Npc1l1	2.0638409	3.11531	15.0583	21.069	10.33	2.8038516	0.01949	0.0456
ENSRNOG00000021090	Pygm	47.468341	61.2678	555.148	206.11	217.5	2.8066199	6.8E-12	9.8E-11
ENSRNOG00000065542	ENSRNOG0000006554	1.0319205	6.23062	20.0777	31.146	14.62	2.8209	0.00817	0.02145
ENSRNOG00000037406	RGD1564171	3.0957614	1.03844	22.0854	7.3284	8.387	2.8267118	0.04068	0.08501
ENSRNOG00000016112	Cd274	16.510727	35.3069	213.827	155.73	105.3	2.8341643	1.4E-10	1.7E-09
ENSRNOG00000033487	AABR07043626.1	2.0638409	2.07687	20.0777	10.077	8.574	2.8621996	0.0314	0.06852
ENSRNOG00000013409	Gclm	518.02407	477.681	4062.72	3258.4	2079	2.8781202	9.6E-57	4.7E-54
ENSRNOG00000020030	Crlf1	4.1276818	6.23062	33.1282	43.054	21.64	2.8799334	0.00069	0.00239
ENSRNOG00000018706	Il15ra	6.1915227	17.6534	107.416	68.704	49.99	2.8845596	2.8E-06	1.6E-05
ENSRNOG00000036631	Ptgdr2	1.0319205	3.11531	15.0583	15.573	8.695	2.8859592	0.02922	0.06445
ENSRNOG00000065306	ENSRNOG0000006530	0	4.15375	20.0777	10.993	8.806	2.9033485	0.03839	0.08113
ENSRNOG00000006116	Hk2	935.95185	910.709	7384.57	6468.2	3925	2.9071291	1.3E-73	1.9E-70
ENSRNOG00000070591	ENSRNOG0000007059	17.542648	3.11531	109.423	46.719	44.2	2.9154228	5.3E-05	0.00024
ENSRNOG00000014125	Evi2b	5.1596023	4.15375	41.1592	29.314	19.95	2.9183773	0.00097	0.00325
ENSRNOG00000028721	Otor	1.0319205	3.11531	24.0932	7.3284	8.892	2.9193482	0.03212	0.06974

ENSRNOG00000007814	Kdm3a	1521.0508	1451.74	12842.7	9722	6384	2.9241221	7.3E-63	4.9E-60
ENSRNOG00000014850	Amhr2	4.1276818	3.11531	44.1709	10.993	15.6	2.9255221	0.00627	0.01709
ENSRNOG00000057291	AABR07019088.1	1.0319205	5.19219	28.1087	19.237	13.39	2.9275366	0.00822	0.02155
ENSRNOG00000058906	Ptprh	2.0638409	3.11531	34.132	5.4963	11.2	2.9322757	0.0212	0.04906
ENSRNOG00000067055	ENSRNOG0000006705	5.1596023	1.03844	31.1204	16.489	13.45	2.9381995	0.00855	0.02234
ENSRNOG00000063571	ENSRNOG0000006357	11.351125	13.4997	84.3262	108.09	54.32	2.9538068	9.6E-08	7.2E-07
ENSRNOG00000013290	Nrip3	30.957614	61.2678	490.899	229.93	203.3	2.9658741	2.7E-13	4.7E-12
ENSRNOG00000019486	Trpv1	2.0638409	1.03844	17.066	7.3284	6.874	2.9718092	0.04848	0.09842
ENSRNOG00000014963	Adgrg1	374.58713	337.492	2983.54	2670.3	1591	2.9889872	3.2E-63	2.3E-60
ENSRNOG00000062369	ENSRNOG0000006236	1.0319205	2.07687	11.0427	13.741	6.973	2.9964387	0.04259	0.08841
ENSRNOG00000028812	Trim80	4.1276818	0	11.0427	21.985	9.289	3.0001054	0.0298	0.0655
ENSRNOG00000042070	Ticam2	8.2553636	3.11531	64.2485	27.481	25.78	3.0090698	0.00036	0.00135
ENSRNOG00000002691	Shcbp1l	3.0957614	7.26906	46.1786	37.558	23.53	3.0143943	0.00031	0.00117
ENSRNOG00000068087	ENSRNOG0000006808	4.1276818	5.19219	67.2602	9.1605	21.44	3.0325584	0.00272	0.00817
ENSRNOG00000031167	ENSRNOG0000003116	212.57561	229.495	2001.74	1639.7	1021	3.0420098	1.8E-52	7.2E-50
ENSRNOG00000068651	ENSRNOG0000006865	9.2872841	16.615	148.575	65.04	59.88	3.0426213	3E-07	2E-06
ENSRNOG00000011923	Mgarp	10.319205	4.15375	71.2757	49.467	33.8	3.058591	2.1E-05	0.0001
ENSRNOG00000063182	ENSRNOG0000006318	75.330193	79.9597	912.53	385.66	363.4	3.0627354	1.6E-18	5.2E-17
ENSRNOG00000062764	ENSRNOG0000006276	80.489795	83.075	878.398	489.17	382.8	3.0630089	4.2E-24	2.3E-22
ENSRNOG00000047697	Ggt1	348.78911	313.608	2646.24	2904.8	1553	3.0670185	1.9E-66	1.8E-63
ENSRNOG00000018276	Mgat4a	2.0638409	1.03844	15.0583	10.993	7.288	3.0681199	0.0355	0.07584
ENSRNOG00000006462	Ero1a	2641.7164	2551.44	19823.7	23795	12203	3.0702926	2.3E-84	4.2E-81

ENSRNOG00000005176	ENSRNOG0000000517	30.957614	34.2684	299.157	249.17	153.4	3.0711472	4.4E-18	1.4E-16
ENSRNOG00000057078	Ddit4	349.82103	373.837	2980.53	3110.9	1704	3.0734636	1.3E-71	1.6E-68
ENSRNOG00000011973	Il7	2.0638409	5.19219	39.1515	21.985	17.1	3.0740268	0.00192	0.00601
ENSRNOG00000014819	Hap1	37.149136	23.8841	240.932	274.81	144.2	3.0789013	1.1E-16	2.8E-15
ENSRNOG00000067412	ENSRNOG0000006741	0	3.11531	13.0505	13.741	7.477	3.1066326	0.03955	0.08309
ENSRNOG00000057249	Gm50449	2.0638409	1.03844	14.0544	12.825	7.495	3.1139998	0.03098	0.06774
ENSRNOG00000024382	Fcgr3a	41.276818	58.1525	392.518	469.93	240.5	3.1173628	2.9E-24	1.6E-22
ENSRNOG00000017061	Patl2	2.0638409	1.03844	15.0583	11.909	7.517	3.1182329	0.03079	0.06739
ENSRNOG00000004753	Napb	192.96913	195.226	2033.87	1354.8	944.2	3.1255575	3E-42	6.4E-40
ENSRNOG00000045553	Proser2	17.542648	17.6534	163.633	144.74	85.89	3.1308282	4.7E-12	7E-11
ENSRNOG00000009227	Aplnr	2.0638409	1.03844	18.0699	9.1605	7.583	3.1309221	0.03125	0.06823
ENSRNOG00000033722	Rnf207	145.50078	136.035	1222.73	1244	687.1	3.1311841	2.8E-51	1.1E-48
ENSRNOG00000016167	Spata2L	165.10727	184.842	1596.17	1470.3	854.1	3.1313119	2.9E-55	1.3E-52
ENSRNOG00000005299	Kif5a	11.351125	20.7687	169.656	115.42	79.3	3.1494613	2.4E-10	2.7E-09
ENSRNOG00000056350	Gm24644	1.0319205	3.11531	23.0893	13.741	10.24	3.1502064	0.01287	0.03187
ENSRNOG00000067247	ENSRNOG0000006724	0	3.11531	17.066	10.993	7.793	3.1719304	0.03393	0.07304
ENSRNOG00000042717	Ciart	31.989534	35.3069	262.014	353.6	170.7	3.1941804	9.3E-20	3.4E-18
ENSRNOG00000010210	Slc7a11	162.01151	136.035	1167.52	1595.8	765.3	3.2129915	1.8E-44	4.7E-42
ENSRNOG00000020124	Lrrc51	5.1596023	8.3075	67.2602	57.711	34.61	3.2141555	4.6E-06	2.5E-05
ENSRNOG00000047545	Adra2a	0	3.11531	16.0621	12.825	8.001	3.2145442	0.02954	0.06502
ENSRNOG00000003147	Sqstm1	8802.2815	8693.8	96161	67191	45212	3.2228746	1.2E-70	1.3E-67
ENSRNOG00000056457	Gpd1	2.0638409	1.03844	20.0777	9.1605	8.085	3.2332944	0.02387	0.05442

ENSRNOG00000064974	ENSRNOG0000006497	1.0319205	2.07687	23.0893	6.4123	8.153	3.2437566	0.02616	0.05873
ENSRNOG00000007545	Angptl4	8.2553636	12.4612	112.435	87.941	55.27	3.273609	9.6E-09	8.6E-08
ENSRNOG00000012886	Maff	779.09994	768.443	9673.42	5508.2	4182	3.294177	2.9E-48	9.4E-46
ENSRNOG00000019741	Isyna1	15.478807	14.5381	188.73	107.18	81.48	3.2999191	4.9E-11	6.2E-10
ENSRNOG00000016361	Plcd4	8.2553636	8.3075	99.3845	65.956	45.48	3.3182642	1.4E-07	1E-06
ENSRNOG00000017209	Tubb3	167.17111	182.765	2141.28	1376.8	967	3.329353	6E-45	1.6E-42
ENSRNOG00000016926	Plekhs1	2.0638409	1.03844	14.0544	17.405	8.64	3.3419577	0.01576	0.03785
ENSRNOG00000001499	Mia	2.0638409	3.11531	38.1476	14.657	14.5	3.3477568	0.00265	0.00798
ENSRNOG00000016837	Ckm	3.0957614	0	5.01942	26.565	8.67	3.3520879	0.0277	0.06165
ENSRNOG00000004177	Myo1a	2.0638409	1.03844	18.0699	13.741	8.728	3.3563983	0.01508	0.03645
ENSRNOG00000011723	Slc44a3	7.2234432	3.11531	53.2058	53.131	29.17	3.3615556	1.6E-05	7.8E-05
ENSRNOG00000027030	Adm	149.62847	174.457	1725.68	1699.3	937.3	3.4017177	6.6E-66	5.4E-63
ENSRNOG00000000983	Zfp394	282.7462	213.918	3528.65	1789	1454	3.4201527	1.1E-36	1.7E-34
ENSRNOG00000028958	AABR07024795.1	1.0319205	1.03844	13.0505	9.1605	6.07	3.4221547	0.03861	0.08149
ENSRNOG00000059865	Dnah12	1.0319205	1.03844	16.0621	6.4123	6.136	3.4375867	0.03961	0.08318
ENSRNOG00000046112	Dedd2	188.84144	172.381	2944.39	984.75	1073	3.4431928	4.3E-06	2.4E-05
ENSRNOG00000024426	AABR07032520.1	1.0319205	1.03844	6.0233	16.489	6.146	3.4456838	0.03964	0.08322
ENSRNOG00000016894	Dnd1	11.351125	6.23062	154.598	39.39	52.89	3.4612026	9.9E-07	6.1E-06
ENSRNOG00000015408	Vil1	3.0957614	2.07687	35.1359	21.985	15.57	3.4631334	0.00112	0.00369
ENSRNOG00000042105	Lce1l	4.1276818	0	21.0816	24.733	12.49	3.4704835	0.00492	0.01386
ENSRNOG00000009370	Tbkbp1	2.0638409	0	11.0427	11.909	6.254	3.4729096	0.03966	0.08325
ENSRNOG00000007601	Inhbe	5.1596023	3.11531	25.0971	66.872	25.06	3.4761039	9.4E-05	0.0004

ENSRNOG00000007393	Ndrg1	4.1276818	2.07687	30.1165	39.39	18.93	3.485736	0.00032	0.0012
ENSRNOG00000029678	Znf454	3.0957614	0	10.0388	24.733	9.467	3.4894876	0.0145	0.03523
ENSRNOG00000013940	RGD1565989	2.0638409	2.07687	42.1631	5.4963	12.95	3.5211671	0.00614	0.01678
ENSRNOG00000027230	Fhod3	12.383045	2.07687	67.2602	98.933	45.16	3.5220512	4.2E-07	2.8E-06
ENSRNOG00000063146	Rab39a	19.606489	28.0378	317.227	236.34	150.3	3.5381165	4.5E-20	1.7E-18
ENSRNOG00000023365	AABR07013086.1	0	2.07687	14.0544	10.077	6.552	3.5399263	0.03374	0.07273
ENSRNOG00000067729	ENSRNOG0000006772	2.0638409	3.11531	45.1748	15.573	16.48	3.549855	0.00107	0.00354
ENSRNOG00000046667	Fosb	61.915227	57.114	1221.73	190.54	382.8	3.568514	0.00212	0.00655
ENSRNOG00000053362	Gabarapl1	1526.2104	1577.39	22146.7	14802	10013	3.5734911	1.4E-76	2.3E-73
ENSRNOG00000019920	Doc2a	21.67033	23.8841	228.885	314.2	147.2	3.5763552	2.6E-20	9.8E-19
ENSRNOG00000019484	Slc6a9	528.34327	578.409	5713.1	7570.2	3598	3.5853217	7.6E-85	1.6E-81
ENSRNOG00000015157	Smtnl2	9.2872841	13.4997	97.3767	175.88	74.01	3.5857328	4E-11	5.2E-10
ENSRNOG00000022983	Trim17	1.0319205	1.03844	14.0544	10.993	6.779	3.5958814	0.02484	0.05624
ENSRNOG00000021729	Iqub	7.2234432	12.4612	169.656	68.704	64.51	3.5968338	4.1E-09	3.9E-08
ENSRNOG00000061360	U3	1.0319205	1.03844	17.066	8.2444	6.845	3.6095608	0.02521	0.05692
ENSRNOG00000006899	Akap14	4.1276818	0	28.1087	22.901	13.78	3.6243453	0.00246	0.00748
ENSRNOG00000007319	Trib3	401.41706	361.376	4546.59	5048.3	2589	3.6529214	2E-102	6E-99
ENSRNOG00000067094	ENSRNOG0000006709	1.0319205	2.07687	17.066	22.901	10.77	3.6862076	0.00463	0.01311
ENSRNOG00000070261	ENSRNOG0000007026	1.0319205	4.15375	41.1592	26.565	18.23	3.7074068	0.00033	0.00124
ENSRNOG00000008449	Neurod4	0	3.11531	31.1204	10.077	11.08	3.7249462	0.00689	0.01855
ENSRNOG00000030616	Slc25a45	17.542648	12.4612	245.951	152.06	107	3.7281719	1.2E-15	2.8E-14
ENSRNOG00000023937	Slc49a3	13.414966	25.9609	319.235	218.94	144.4	3.7726421	1.8E-19	6.2E-18

ENSRNOG00000018656	Ampd1	3.0957614	0	31.1204	11.909	11.53	3.7920612	0.00501	0.01408
ENSRNOG00000026309	Lrrc23	1.0319205	1.03844	15.0583	13.741	7.717	3.7977712	0.01405	0.03426
ENSRNOG00000003745	Atf3	87.713239	83.075	1889.31	555.13	653.8	3.8391288	3.7E-06	2.1E-05
ENSRNOG00000064855	ENSRNOG0000006485	1.0319205	3.11531	44.1709	16.489	16.2	3.8693981	0.00069	0.0024
ENSRNOG00000063564	ENSRNOG0000006356	4.1276818	2.07687	43.167	47.635	24.25	3.8706923	1.7E-05	8.6E-05
ENSRNOG00000062665	ENSRNOG0000006266	1.0319205	1.03844	17.066	13.741	8.219	3.8946064	0.01053	0.02674
ENSRNOG00000001342	ENSRNOG0000000134	1.0319205	1.03844	22.0854	9.1605	8.329	3.9132102	0.01108	0.02798
ENSRNOG00000055221	Acot1	1.0319205	1.03844	23.0893	8.2444	8.351	3.9169482	0.01139	0.02864
ENSRNOG00000009870	Tmem88	1.0319205	3.11531	40.1553	22.901	16.8	3.9262417	0.00037	0.00138
ENSRNOG00000032917	Zfand2a	3140.1339	2707.21	61121.5	30457	24356	3.9691326	2E-59	1.2E-56
ENSRNOG00000005615	Gadd45a	922.53689	881.633	16476.7	12139	7605	3.9873741	4E-109	1E-105
ENSRNOG00000068958	ENSRNOG0000006895	0	5.19219	38.1476	44.886	22.06	4.0023877	7E-05	0.00031
ENSRNOG00000004067	Nrcam	60.883307	61.2678	717.777	1256.8	524.2	4.0153451	2.1E-42	4.4E-40
ENSRNOG00000016073	Taar1	1.0319205	1.03844	32.1243	1.8321	9.007	4.0317551	0.01474	0.03574
ENSRNOG00000007284	Slc2a1	884.35583	819.327	14735	13289	7432	4.0399028	3E-148	1E-144
ENSRNOG00000038499	ENSRNOG0000003849	3.0957614	2.07687	55.2136	32.062	23.11	4.0745252	2.3E-05	0.00011
ENSRNOG00000021996	Nlrp4	2.0638409	1.03844	33.1282	19.237	13.87	4.0746672	0.0009	0.00304
ENSRNOG00000029147	Rbm11	3.0957614	2.07687	52.2019	35.726	23.28	4.0856791	1.8E-05	8.9E-05
ENSRNOG00000065584	ENSRNOG0000006558	2.0638409	0	22.0854	13.741	9.473	4.1133675	0.00637	0.01732
ENSRNOG00000067248	ENSRNOG0000006724	2.0638409	4.15375	103.4	4.5802	28.55	4.1165223	0.00024	0.00094
ENSRNOG00000020078	Vstm4	1.0319205	0	6.0233	11.909	4.741	4.1181229	0.04975	0.1006
ENSRNOG00000028330	AABR07067600.1	21.67033	11.4228	327.266	249.17	152.4	4.1211996	2.8E-23	1.5E-21

ENSRNOG0000 0034102	Plk5	1.0319205	0	9.03495	9.1605	4.807	4.1371938	0.04664	0.09532
ENSRNOG0000 0063491	ENSRNOG0 000006349	1.0319205	0	10.0388	8.2444	4.829	4.143492	0.04616	0.09453
ENSRNOG0000 0039559	Fam219a	246.62899	322.954	6690.88	3454.4	2679	4.154697	4.8E-58	2.6E-55
ENSRNOG0000 0065556	ENSRNOG0 000006555	0	1.03844	14.0544	4.5802	4.918	4.1654725	0.04707	0.09597
ENSRNOG0000 0030719	Csmd1	12.383045	9.34593	153.594	237.26	103.1	4.1695186	2.9E-17	8.1E-16
ENSRNOG0000 0030914	Lexm	26.829932	15.5766	388.503	379.24	202.5	4.1775395	6.3E-31	6.7E-29
ENSRNOG0000 0013320	ENSRNOG0 000001332	2.0638409	0	34.132	3.6642	9.965	4.1886495	0.0092	0.02378
ENSRNOG0000 0045738	Ak4	4.1276818	2.07687	54.2097	59.543	29.99	4.1957177	8.9E-07	5.6E-06
ENSRNOG0000 0029830	Adm2	10.319205	10.3844	120.466	259.24	100.1	4.1983124	3.4E-15	7.5E-14
ENSRNOG0000 0026196	Lrrd1	2.0638409	0	32.1243	6.4123	10.15	4.2168003	0.00657	0.01778
ENSRNOG0000 0070060	ENSRNOG0 000007006	0	2.07687	27.1049	11.909	10.27	4.2322868	0.00456	0.01296
ENSRNOG0000 0045973	Gzmbl1	1.0319205	0	14.0544	5.4963	5.146	4.2381899	0.04038	0.08451
ENSRNOG0000 0018414	Csf1r	4.1276818	15.5766	178.691	194.2	98.15	4.2439236	2.2E-16	5.4E-15
ENSRNOG0000 0070622	Hsd3b1	1.0319205	0	16.0621	3.6642	5.19	4.2502031	0.0422	0.08772
ENSRNOG0000 0014471	B3gnt6	0	1.03844	19.0738	1.8321	5.486	4.3301633	0.04119	0.08594
ENSRNOG0000 0065560	ENSRNOG0 000006556	1.0319205	4.15375	97.3767	9.1605	27.93	4.3595685	7.4E-05	0.00032
ENSRNOG0000 0067571	ENSRNOG0 000006757	0	3.11531	54.2097	10.993	17.08	4.387432	0.00058	0.00206
ENSRNOG0000 0011268	Chd5	0	1.03844	8.03107	13.741	5.703	4.3946698	0.02828	0.0627
ENSRNOG0000 0017243	Bnip3	1073.1973	1048.82	23339.3	22265	11931	4.4256358	3E-196	2E-192
ENSRNOG0000 0021796	RGD15651 66	2.0638409	1.03844	32.1243	34.81	17.51	4.4305295	0.00012	0.0005
ENSRNOG0000 0057952	Gm23130	1.0319205	1.03844	29.1126	24.733	13.98	4.7004002	0.00049	0.00179
ENSRNOG0000 0033262	Reep6	7.2234432	6.23062	230.893	121.83	91.55	4.710958	5.1E-16	1.2E-14

ENSRNOG0000 0054809	AABR07026 032.1	3.0957614	0	59.2291	27.481	22.45	4.8028455	2.4E-05	0.00012
ENSRNOG0000 0065164	ENSRNOG0 000006516	2.0638409	3.11531	124.482	21.985	37.91	4.8199561	7.2E-07	4.6E-06
ENSRNOG0000 0005093	Lgr6	4.1276818	2.07687	90.3495	88.857	46.35	4.850993	4.2E-10	4.7E-09
ENSRNOG0000 0028016	Ncf2	3.0957614	5.19219	124.482	127.33	65.03	4.9261023	1.9E-13	3.5E-12
ENSRNOG0000 0065360	ENSRNOG0 000006536	1.0319205	0	12.0466	20.153	8.308	4.9616154	0.00745	0.01983
ENSRNOG0000 0016768	Oprl1	2.0638409	2.07687	66.2563	65.04	33.86	4.9867518	7.3E-08	5.6E-07
ENSRNOG0000 0006146	Trim54	2.0638409	2.07687	48.1864	90.689	35.75	5.0693181	6.7E-08	5.2E-07
ENSRNOG0000 0037621	ENSRNOG0 000003762	2.0638409	0	33.1282	37.558	18.19	5.0948612	8.6E-05	0.00037
ENSRNOG0000 0009528	Sdcbp2	0	6.23062	133.517	85.193	56.23	5.1343315	0.00286	0.00854
ENSRNOG0000 0062654	ENSRNOG0 000006265	2.0638409	2.07687	114.443	32.062	37.66	5.1427135	1.7E-07	1.2E-06
ENSRNOG0000 0032745	Slc17a3	1.0319205	1.03844	30.1165	44.886	19.27	5.1801146	5.1E-05	0.00023
ENSRNOG0000 0025736	Tcf23	0	1.03844	22.0854	18.321	10.36	5.2851861	0.00293	0.00873
ENSRNOG0000 0017602	Il34	1.0319205	0	11.0427	29.314	10.35	5.2882167	0.00337	0.00989
ENSRNOG0000 0017766	Ca12	4.1276818	5.19219	138.536	234.51	95.59	5.3243642	2.1E-17	6E-16
ENSRNOG0000 0033923	Aox2	1.0319205	1.03844	52.2019	33.894	22.04	5.3768096	1.8E-05	8.7E-05
ENSRNOG0000 0001115	Slc29a4	0	1.03844	25.0971	18.321	11.11	5.3885842	0.00218	0.00674
ENSRNOG0000 0013886	Fyb1	1.0319205	1.03844	51.1981	44.886	24.54	5.5359814	6.3E-06	3.4E-05
ENSRNOG0000 0004756	Syt2	0	2.07687	45.1748	57.711	26.24	5.6348923	4.1E-06	2.3E-05
ENSRNOG0000 0015590	Dnaaf1	0	1.03844	18.0699	41.222	15.08	5.8411985	0.0006	0.00212
ENSRNOG0000 0068001	Abcb4	1.0319205	0	39.1515	22.901	15.77	5.9047694	0.00045	0.00163
ENSRNOG0000 0045677	Nlrp6	1.0319205	1.03844	102.396	24.733	32.3	5.9379338	2.1E-06	1.2E-05
ENSRNOG0000 0019661	Gdf15	1.0319205	1.03844	130.505	16.489	37.27	6.1474945	1.7E-06	1E-05

ENSRNOG0000 0014117	Hmox1	2756.2595	2895.16	226618	177085	1E+05	6.158536	3E-306	4E-302
ENSRNOG0000 0053769	Soat2	1.0319205	1.03844	22.0854	126.41	37.64	6.1666925	9.3E-07	5.9E-06
ENSRNOG0000 0046195	AABR07029 596.1	1.0319205	0	50.1942	29.314	20.13	6.2622861	0.00013	0.00054
ENSRNOG0000 0014948	Osgin1	48.500261	34.2684	7605.42	3868.5	2889	7.1145872	3E-124	1E-120
ENSRNOG0000 0004133	ENSRNOG0 000000413	0	0	7.02718	5.4963	3.131	7.3604423	0.04804	0.09766
ENSRNOG0000 0014465	Oca2	0	0	7.02718	5.4963	3.131	7.3604423	0.04804	0.09766
ENSRNOG0000 0012371	Car7	0	0	8.03107	4.5802	3.153	7.3696546	0.04781	0.09726
ENSRNOG0000 0047304	Btnl5	0	0	9.03495	3.6642	3.175	7.3788386	0.04779	0.09724
ENSRNOG0000 0068650	ENSRNOG0 000006865	0	0	11.0427	1.8321	3.219	7.3972336	0.04874	0.09886
ENSRNOG0000 0002350	Eaf2	0	0	9.03495	4.5802	3.404	7.4798585	0.04282	0.08884
ENSRNOG0000 0021285	Celsr1	0	0	9.03495	4.5802	3.404	7.4798585	0.04282	0.08884
ENSRNOG0000 0065218	Olr1615	0	0	12.0466	1.8321	3.47	7.5055023	0.0439	0.09072
ENSRNOG0000 0063737	ENSRNOG0 000006373	0	0	12.0466	2.7481	3.699	7.5981911	0.03911	0.08228
ENSRNOG0000 0017237	Lama1	0	0	14.0544	0.916	3.743	7.6143718	0.04126	0.08604
ENSRNOG0000 0001270	Hvcn1	0	2.07687	230.893	177.71	102.7	7.6238333	2.2E-12	3.4E-11
ENSRNOG0000 0059926	Snord71	0	0	6.0233	9.1605	3.796	7.6402435	0.03608	0.07695
ENSRNOG0000 0063870	ENSRNOG0 000006387	0	0	8.03107	7.3284	3.84	7.6553839	0.03535	0.07557
ENSRNOG0000 0063176	ENSRNOG0 000006317	0	0	10.0388	5.4963	3.884	7.6703797	0.03506	0.07508
ENSRNOG0000 0066987	ENSRNOG0 000006698	0	0	10.0388	5.4963	3.884	7.6703797	0.03506	0.07508
ENSRNOG0000 0046746	AC130391. 1	0	0	6.0233	10.077	4.025	7.7250094	0.03308	0.07149
ENSRNOG0000 0020942	Plekha4	0	0	10.0388	6.4123	4.113	7.7534449	0.03201	0.06958
ENSRNOG0000 0052688	Dnah2	0	0	11.0427	5.4963	4.135	7.7604865	0.03198	0.06952

ENSRNOG00000056955	Bicdl1	0	0	14.0544	2.7481	4.201	7.7816346	0.03262	0.07066
ENSRNOG00000018694	Lipg	0	0	6.0233	11.909	4.483	7.8809084	0.02818	0.06251
ENSRNOG00000050509	Ebi3	0	0	14.0544	4.5802	4.659	7.931715	0.02712	0.06058
ENSRNOG00000048430	Myo18b	0	0	15.0583	3.6642	4.681	7.9380361	0.02735	0.061
ENSRNOG00000070875	Nat8f4	0	0	11.0427	8.2444	4.822	7.9833297	0.02498	0.05651
ENSRNOG00000058422	Lilrb3a	2.0638409	0	228.885	310.54	135.4	8.0262611	8.2E-14	1.5E-12
ENSRNOG00000068754	Cd244	0	0	7.02718	13.741	5.192	8.0926954	0.02244	0.05166
ENSRNOG00000016606	Snorc	0	0	17.066	4.5802	5.412	8.1475689	0.02176	0.05025
ENSRNOG00000018236	Cym	0	1.03844	169.656	141.07	77.94	8.2290033	4.1E-08	3.3E-07
ENSRNOG00000005348	Pamr1	0	0	0	22.901	5.725	8.2355142	0.02514	0.05678
ENSRNOG00000066887	ENSRNOG0000006688	0	0	12.0466	10.993	5.76	8.2403513	0.01887	0.04435
ENSRNOG00000000446	Btnl7	0	0	24.0932	3.6642	6.939	8.5058506	0.01536	0.03702
ENSRNOG00000026653	Hcar2	0	0	25.0971	3.6642	7.19	8.5571035	0.01458	0.0354
ENSRNOG00000015201	Tmem266	0	0	21.0816	9.1605	7.561	8.630932	0.01252	0.03111
ENSRNOG00000024210	Nlrc3	0	0	15.0583	15.573	7.658	8.651616	0.01197	0.02992
ENSRNOG00000016920	Spire2	0	0	17.066	14.657	7.931	8.7016126	0.01133	0.02852
ENSRNOG00000040183	Olr1684	0	0	12.0466	25.649	9.424	8.9528756	0.00872	0.02272
ENSRNOG00000036670	Tex19.1	0	0	35.1359	10.993	11.53	9.2395706	0.00647	0.01755
ENSRNOG00000017064	Tdrd1	0	0	36.1398	13.741	12.47	9.3527046	0.00561	0.01554
ENSRNOG00000007102	Acss1	0	0	24.0932	27.481	12.89	9.4035516	0.00512	0.01432
ENSRNOG00000015903	Add2	0	0	40.1553	12.825	13.25	9.4394368	0.00515	0.0144
ENSRNOG00000056279	AC115369.1	0	0	28.1087	27.481	13.9	9.5112888	0.00452	0.01284

ENSRNOG0000 0064738	Btnl8	0	0	61.2369	0.916	15.54	9.6717684	0.02628	0.05898
ENSRNOG0000 0026791	Pgbd5	0	0	33.1282	32.062	16.3	9.7410768	0.00346	0.01014
ENSRNOG0000 0049208	AABR07054 456.2	0	0	53.2058	41.222	23.61	10.275046	0.00186	0.00584
ENSRNOG0000 0064010	Olr1345	0	0	62.2408	49.467	27.93	10.517555	0.00139	0.0045
ENSRNOG0000 0064634	Sorl1	0	0	97.3767	29.314	31.67	10.697584	0.0012	0.00394
ENSRNOG0000 0063133	Olr1343	0	0	73.2835	63.207	34.12	10.806852	0.00098	0.00329
ENSRNOG0000 0068148	ENSRNOG0 000006814	0	0	281.087	38.474	79.89	12.034265	0.00095	0.00317

Table S2.Table showing list of differentially expressed genes between Hypoxia and Hypoxia + VEGFB

Gene_ID	Gene Name	Hyp1_Normalized.Read.Count	Hyp2_Normalized.Read.Count	HypPosVB1_Normalized.Read.Count	HypPosVB2_Normalized.Read.Count	baseMean	log2FoldChange	pvalue	padj
ENSRNOG00000052026	Gm25777	0	0	2.90718	12.8685	3.9439	7.72046234484469	0.0356898940844133	0.978863622852093
ENSRNOG00000047304	Btnl5	9.58039	3.87866	0	0	3.3648	-7.54253173170921	0.0427551788766234	0.978863622852093
ENSRNOG00000066594	ENSRNOG00000066594	5.32244	7.75733	0	0	3.2699	-7.50489519765688	0.0439555756993978	0.978863622852093
ENSRNOG00000065360	ENSRNOG00000065360	12.7738	21.3327	0	0.98989	8.7741	-5.11986559084417	0.00564940878284964	0.978863622852093
ENSRNOG00000064738	Btnl8	64.9337	0.96967	1.93812	0.98989	17.208	-4.48694773059751	0.00199794500057555	0.978863622852093
ENSRNOG00000053830	AC133055.1	17.0318	4.84833	0.96906	0	5.7123	-4.48336097069794	0.0292832850383616	0.978863622852093
ENSRNOG00000057264	Tex49	0	0.96967	6.78342	11.8787	4.9079	4.22254823810814	0.0449497041851906	0.978863622852093
ENSRNOG00000007338	Fbln2	0	1.93933	12.59778	19.7978	8.5837	4.02123027532164	0.0104167422056115	0.978863622852093
ENSRNOG00000027228	Mogat2	26.6122	3.87866	0.96906	0.98989	8.1124	-3.95622524799516	0.0157630638050129	0.978863622852093
ENSRNOG00000000446	Btnl7	25.5477	3.87866	0.96906	0.98989	7.8463	-3.90494632384803	0.0178552076210618	0.978863622852093
ENSRNOG00000067571	ENSRNOG00000067571	57.4823	11.636	3.87624	0.98989	18.496	-3.82061384673296	0.00074437819549995	0.978863622852093
ENSRNOG00000067248	ENSRNOG00000067248	109.642	4.84833	8.72154	2.96966	31.545	-3.28716256310236	0.00161525245044825	0.978863622852093

ENSRNOG00000063604	ENSRNOG00000063604	35.1281	8.72699	4.8453	0.98989	12.423	-2.90264106218535	0.0173393413715209	0.978863622852093
ENSRNOG00000068148	ENSRNOG00000068148	298.056	40.726	34.88616	10.8888	96.139	-2.88702536356062	0.0390502919145303	0.978863622852093
ENSRNOG00000002978	Faslg	40.4505	1.93933	3.87624	1.97978	12.061	-2.85003070912266	0.0341618240722131	0.978863622852093
ENSRNOG00000062665	ENSRNOG00000062665	18.0963	14.545	1.93812	2.96966	9.3873	-2.73416355641169	0.029513228653859	0.978863622852093
ENSRNOG00000024631	Chadl	20.2253	11.636	3.87624	0.98989	9.1818	-2.70545095280572	0.0378564541843024	0.978863622852093
ENSRNOG00000014201	ENSRNOG00000014201	15.9673	14.545	1.93812	2.96966	8.855	-2.63725497831306	0.0393714415123333	0.978863622852093
ENSRNOG00000037375	RGD1565410	54.2889	9.69666	8.72154	1.97978	18.672	-2.57378675891105	0.0145450164378855	0.978863622852093
ENSRNOG00000065164	ENSRNOG00000065164	131.996	23.272	15.50496	12.8685	45.91	-2.44918771775072	0.00095272909346374	0.978863622852093
ENSRNOG00000001499	Mia	40.4505	15.5147	9.6906	0.98989	16.661	-2.38347848475311	0.0244811415844634	0.978863622852093
ENSRNOG00000015366	Neurl3	17.0318	22.3023	4.8453	2.96966	11.787	-2.33107745837954	0.0335563507866941	0.978863622852093
ENSRNOG00000064855	ENSRNOG00000064855	46.8374	17.454	9.6906	3.95955	19.485	-2.23096320481604	0.0156350464164138	0.978863622852093
ENSRNOG00000018656	Ampd1	32.9991	12.6057	5.81436	3.95955	13.845	-2.21819682649376	0.0338413197892429	0.978863622852093
ENSRNOG00000015903	Add2	42.5795	13.5753	9.6906	2.96966	17.204	-2.14372243991623	0.0312658879178837	0.978863622852093
ENSRNOG00000020981	Prss46	10.6449	2.909	39.73146	17.818	17.776	2.09819621124855	0.0291578154622859	0.978863622852093

ENSRNOG00000054493	Rpph1	138.383	44.6046	30.04086	15.8382	57.217	-1.99257190304355	0.00134518480590245	0.978863622852093
ENSRNOG00000003494	Ppfia4	4.25795	7.75733	18.41214	28.7067	14.784	1.96478727035835	0.0445715687205136	0.978863622852093
ENSRNOG00000001193	Hsf2bp	3.19346	17.454	31.97898	48.5045	25.283	1.95038312797741	0.0198774074629569	0.978863622852093
ENSRNOG00000045677	Nlrp6	108.578	26.181	23.25744	12.8685	42.721	-1.89560582323545	0.00796302091350977	0.978863622852093
ENSRNOG00000069549	ENSRNOG00000069549	137.319	19.3933	23.25744	18.8079	49.694	-1.89445986678251	0.0103647190988466	0.978863622852093
ENSRNOG00000017484	Gja5	10.6449	17.454	30.04086	73.2517	32.848	1.87328950851302	0.00884357709790357	0.978863622852093
ENSRNOG00000056279	AC115369.1	29.8056	29.09	11.62872	4.94944	18.868	-1.82698557409147	0.0355953048769749	0.978863622852093
ENSRNOG00000069130	ENSRNOG00000069130	113.9	20.363	22.28838	17.818	43.592	-1.73990234451873	0.0175880058815138	0.978863622852093
ENSRNOG00000065560	ENSRNOG00000065560	103.255	9.69666	22.28838	11.8787	36.78	-1.72131169532538	0.0451613512706561	0.978863622852093
ENSRNOG00000005998	Smoc1	14.9028	16.4843	29.0718	70.282	32.685	1.6609631149677	0.0176990206228	0.978863622852093
ENSRNOG00000016894	Dnd1	163.931	41.6956	35.85522	29.6966	67.795	-1.64664246681618	0.00599987565581296	0.978863622852093
ENSRNOG00000049383	Krtap3-3	12.7738	8.72699	31.00992	33.6562	21.542	1.59214462055267	0.0445270875636616	0.978863622852093
ENSRNOG00000025327	Tert	81.9655	39.7563	28.10274	12.8685	40.673	-1.56759121691452	0.0161828441182271	0.978863622852093
ENSRNOG00000042084	Acsm1	11.7094	13.5753	29.0718	42.5652	24.23	1.50077795512641	0.0457071098642336	0.978863622852093

ENSRNOG00000021161	Fermt3	155.415	41.6956	47.48394	22.7674	66.841	-1.48531029860148	0.0159473091288708	0.978863622852093
ENSRNOG00000070263	5_8S_rRNA	85.159	24.2416	27.13368	11.8787	37.103	-1.48363030840825	0.0427799007566032	0.978863622852093
ENSRNOG00000070328	RGD1559667	50.0309	31.0293	12.59778	16.8281	27.622	-1.46068984325768	0.0406205820568736	0.978863622852093
ENSRNOG00000051372	Mycn	20.2253	26.181	42.63864	80.1809	42.306	1.40169541118161	0.020428795111096	0.978863622852093
ENSRNOG00000048924	Islr	23.4187	32.9686	69.77232	79.191	51.338	1.39861538520388	0.00835496505285351	0.978863622852093
ENSRNOG00000031834	Nkain4	21.2897	37.817	39.73146	114.827	53.416	1.38284219891554	0.0242683588915798	0.978863622852093
ENSRNOG00000069861	ENSRNOG00000069861	11.7094	43.635	67.8342	77.2113	50.097	1.38270045068303	0.0256377433278887	0.978863622852093
ENSRNOG00000050647	ENSRNOG00000050647	245.897	49.453	66.86514	52.4641	103.67	-1.30523242790669	0.021635262850183	0.978863622852093
ENSRNOG00000069152	ENSRNOG00000069152	381.087	134.784	148.2662	65.3326	182.37	-1.27033795983743	0.00628250782521928	0.978863622852093
ENSRNOG00000049770	Cryba4	70.2562	47.5136	108.5347	171.251	99.389	1.25048994046861	0.00335223129474579	0.978863622852093
ENSRNOG00000005277	Ptprv	26.6122	44.6046	61.05078	105.918	59.546	1.22554759185307	0.021019608766903	0.978863622852093
ENSRNOG00000010666	Ccn5	120.287	198.782	274.244	471.187	266.12	1.22218802449558	0.00033938098580425	0.978863622852093
ENSRNOG00000001514	Cdca7	39.386	79.5126	86.24634	190.058	98.801	1.21291132464787	0.012897934922309	0.978863622852093
ENSRNOG00000017912	Atp2a3	29.8056	128.966	121.1325	247.472	131.84	1.211087356736	0.0229958640394569	0.978863622852093

ENSRNOG00000007601	Inhbe	26.6122	70.7856	61.05078	163.331	80.445	1.19959822518072	0.0336087147311071	0.978863622852093
ENSRNOG00000030228	ENSRNOG00000030228	212.897	86.3003	89.15352	44.545	108.22	-1.15973535468755	0.0176462734831148	0.978863622852093
ENSRNOG00000033734	Tnnt2	41.515	43.635	96.906	93.0495	68.776	1.15724075077218	0.0108486363508471	0.978863622852093
ENSRNOG00000003081	ENSRNOG00000003081	19.1608	58.18	80.43198	92.0596	62.458	1.15143459276473	0.0380172897272295	0.978863622852093
ENSRNOG00000063182	ENSRNOG00000063182	967.619	408.229	387.624	239.553	500.76	-1.13248507725318	0.00117566163291647	0.978863622852093
ENSRNOG00000029141	Trabd2b	28.7412	97.9363	108.5347	159.372	98.646	1.07586974684789	0.0351844960206428	0.978863622852093
ENSRNOG00000014508	Mgll	81.9655	208.478	245.1722	365.269	225.22	1.06876095111327	0.00644350555069928	0.978863622852093
ENSRNOG00000035620	Mir22	222.478	120.239	93.99882	70.282	126.75	-1.05893902439872	0.00866435983099682	0.978863622852093
ENSRNOG00000065190	ENSRNOG00000065190	155.415	81.4519	76.55574	37.6157	87.76	-1.05048074794159	0.0314160145584018	0.978863622852093
ENSRNOG00000067619	ENSRNOG00000067619	105.384	60.1193	50.39112	29.6966	61.398	-1.04471307588639	0.0464936672963001	0.978863622852093
ENSRNOG00000007582	Zswim4	2496.22	1152.93	1061.121	710.739	1355.3	-1.04190245954911	0.00054302988333895	0.978863622852093
ENSRNOG00000013463	Kcnj8	65.9982	94.0576	114.3491	213.816	122.06	1.03374128812859	0.0123504497318355	0.978863622852093
ENSRNOG00000047746	AABR07000398.1	282.089	89.2093	87.2154	94.0393	138.14	-1.03271306365468	0.0259349535034237	0.978863622852093
ENSRNOG00000026607	Tnfsf18	146.899	99.8756	90.12258	31.6764	92.143	-1.01681000762088	0.0409699275444881	0.978863622852093

ENSRNOG00000066573	C4a	134.125	82.4216	58.1436	49.4944	81.046	-1.00663120374277	0.024761711812326	0.978863622852093
ENSRNOG00000000275	Fam13c	88.3525	181.328	225.791	316.764	203.06	1.00575392835497	0.00610094447937835	0.978863622852093
ENSRNOG00000043151	Cntln	47.9019	160.965	133.7303	285.088	156.92	1.00050618501411	0.0408834192944215	0.978863622852093
ENSRNOG00000046307	Glyctk	29.8056	66.907	91.09164	102.948	72.688	1.00008528257541	0.0427764340594544	0.978863622852093
ENSRNOG00000068243	Cdk5r1	302.314	98.9059	143.4209	57.4135	150.51	-0.996399656103475	0.0465543752835995	0.978863622852093
ENSRNOG00000021090	Pygm	588.662	218.175	265.5224	142.544	303.73	-0.98218349715569	0.0150127219505285	0.978863622852093
ENSRNOG00000021578	Wfikkn1	262.928	101.815	111.4419	73.2517	137.36	-0.979621360501535	0.0280919307136957	0.978863622852093
ENSRNOG00000008915	Prima1	62.8048	125.087	140.5137	227.674	139.02	0.967432344893533	0.018680913544915	0.978863622852093
ENSRNOG00000071065	ENSRNOG00000071065	1467.93	647.737	674.4658	409.814	799.99	-0.9637548628786	0.00329228763124722	0.978863622852093
ENSRNOG00000049614	AABR07028488.1	128.803	254.052	317.8517	425.652	281.59	0.955304895343956	0.00423689738501207	0.978863622852093
ENSRNOG00000024178	Cenpt	58.5468	216.236	195.7501	337.552	202.02	0.953804087550962	0.0395878458319853	0.978863622852093
ENSRNOG00000013589	Cxcl12	150.093	344.231	357.5831	598.882	362.7	0.950402895794785	0.00812704217887074	0.978863622852093
ENSRNOG00000057501	Fam81a	54.2889	72.7249	79.46292	165.311	92.947	0.944542057920232	0.0397118399198798	0.978863622852093
ENSRNOG00000017072	Slc16a14	37.2571	94.0576	124.0397	127.696	95.762	0.934680480276923	0.0439876796892306	0.978863622852093

ENSRNOG00000024688	Erfe	78.7721	81.4519	107.5657	197.978	116.44	0.930728794513913	0.0217028866388054	0.978863622852093
ENSRNOG00000007882	Ablim2	36.1926	65.9373	84.30822	110.867	74.326	0.930722993861926	0.0496329689220304	0.978863622852093
ENSRNOG00000055716	Oscar	200.124	92.1183	103.6894	49.4944	111.36	-0.929615029559292	0.0494689256415476	0.978863622852093
ENSRNOG00000017206	Igfbp5	138.383	397.563	362.4284	658.275	389.16	0.927659037747974	0.0186319878767981	0.978863622852093
ENSRNOG00000028344	Mmp11	61.7403	171.631	178.307	266.28	169.49	0.926630365634159	0.030342742501926	0.978863622852093
ENSRNOG00000003794	Nmral1	84.0945	155.147	183.1523	270.239	173.16	0.91962328481796	0.013392661488814	0.978863622852093
ENSRNOG00000006859	Insig1	3309.49	1633.89	1621.237	999.787	1891.1	-0.91507362702044	0.00183507069140983	0.978863622852093
ENSRNOG00000062541	ENSRNOG00000062541	213.962	417.926	501.004	688.962	455.46	0.911592842216636	0.00314588331715771	0.978863622852093
ENSRNOG00000062741	Ldhal6b	134.125	94.0576	66.86514	54.4438	87.373	-0.909956329254267	0.0322497123316384	0.978863622852093
ENSRNOG00000008862	Abcg4	122.416	86.3003	57.17454	54.4438	80.084	-0.901597978137915	0.0384927369268691	0.978863622852093
ENSRNOG00000004094	Ptger1	180.963	91.1486	93.99882	51.4742	104.4	-0.901163521737817	0.0490745179036669	0.978863622852093
ENSRNOG00000008055	Ccne2	193.737	448.955	387.624	812.698	460.75	0.899842690943456	0.0159676128313413	0.978863622852093
ENSRNOG00000063266	ENSRNOG00000063266	150.093	81.4519	72.6795	51.4742	88.925	-0.896915932996877	0.0478528483007634	0.978863622852093
ENSRNOG00000065861	Samd1	227.8	448.955	426.3864	833.486	484.16	0.895284212086064	0.00916579964733595	0.978863622852093

ENSRNOG00000018262	Ampd3	156.48	132.844	62.01984	95.0292	111.59	-0.881450852216015	0.0237317303542379	0.978863622852093
ENSRNOG00000057284	Cenpb	87.288	190.055	236.4506	274.199	197	0.877859093537076	0.0168651635594398	0.978863622852093
ENSRNOG00000033262	Reep6	244.832	128.966	142.4518	61.373	144.41	-0.873067651142367	0.0475998895422295	0.978863622852093
ENSRNOG00000059997	Brip1	133.061	243.386	262.6153	425.652	266.18	0.868517968248496	0.0118123759975708	0.978863622852093
ENSRNOG00000030616	Slc25a45	260.799	160.965	166.6783	64.3427	163.2	-0.866880423723761	0.0429663508196279	0.978863622852093
ENSRNOG00000026143	Ckap2l	240.574	474.167	508.7565	794.88	504.59	0.865666684617597	0.00630471540931344	0.978863622852093
ENSRNOG00000015275	Ska1	145.835	315.141	336.2638	503.853	325.27	0.863922434726169	0.0127275300450869	0.978863622852093
ENSRNOG00000031916	Timeless	497.116	1140.33	1195.82	1783.78	1154.3	0.862931743488776	0.00560912983410022	0.978863622852093
ENSRNOG00000020022	Psmc3ip	63.8692	123.148	138.5756	201.937	131.88	0.861476218609534	0.0331756319381935	0.978863622852093
ENSRNOG00000000983	Zfp394	3741.67	1893.76	1845.09	1257.16	2184.4	-0.860949043256545	0.00203225455222919	0.978863622852093
ENSRNOG00000013215	Dctd	88.3525	196.842	200.5954	317.754	200.89	0.859337585761705	0.0268954483982609	0.978863622852093
ENSRNOG00000043486	Tnfrsf26	451.343	246.295	217.0694	167.291	270.5	-0.858685321084762	0.00883906111740417	0.978863622852093
ENSRNOG00000051915	Spred3	737.69	395.624	324.6351	300.926	439.72	-0.856392301928885	0.00364835458099865	0.978863622852093
ENSRNOG00000025811	Cfp	352.345	190.055	158.9258	140.564	210.47	-0.855375135293227	0.0127315797797169	0.978863622852093

ENSRNOG00000063590	Fzd4	216.091	511.984	552.3642	760.234	510.17	0.84878574692969	0.0108365885120236	0.978863622852093
ENSRNOG00000003397	Adprm	848.397	507.135	456.4273	296.966	527.23	-0.846554436558315	0.00382446436939741	0.978863622852093
ENSRNOG00000049976	Gzmbl2	81.9655	94.0576	140.5137	176.2	123.18	0.846423743818386	0.0193151442112304	0.978863622852093
ENSRNOG00000000142	Plxdc2	523.728	940.576	1094.069	1535.32	1023.4	0.843716886837246	0.00194337698317574	0.978863622852093
ENSRNOG00000029651	Rdh16	87.288	65.9373	125.9778	148.483	106.92	0.842490158439458	0.0287946627830365	0.978863622852093
ENSRNOG00000049862	Rnf157	129.867	368.473	335.2948	559.287	348.23	0.842199630580493	0.0311412279769302	0.978863622852093
ENSRNOG00000018716	Dennd2c	845.203	478.045	467.0869	271.229	515.39	-0.840929027357919	0.00722066423217894	0.978863622852093
ENSRNOG00000053309	Tubd1	72.3851	76.6036	131.7922	134.625	103.85	0.838102821742312	0.0267318222579215	0.978863622852093
ENSRNOG00000006731	Spc25	216.091	459.622	430.2627	776.072	470.51	0.834762100789148	0.0154845604957447	0.978863622852093
ENSRNOG00000002751	Zdhhc15	65.9982	76.6036	148.2662	104.928	98.949	0.827645627362566	0.0396765213242329	0.978863622852093
ENSRNOG00000024428	Kif20a	479.019	812.58	876.0303	1417.52	896.29	0.827636668917721	0.00340580042314385	0.978863622852093
ENSRNOG00000013148	Rad54l	111.771	196.842	219.0076	327.653	213.82	0.822620697802803	0.0183962163949859	0.978863622852093
ENSRNOG00000045844	Impact	12345.9	7143.53	6568.289	4452.52	7627.6	-0.822384768697264	0.00107025591554189	0.978863622852093
ENSRNOG00000027894	Iqgap3	197.995	517.802	512.6328	752.315	495.19	0.819977590824218	0.0204243694081674	0.978863622852093

ENSRNOG00000027936	Ccdc8	237.381	541.074	515.5399	859.223	538.3	0.819185755050635	0.0162401604571035	0.978863622852093
ENSRNOG00000004921	Nusap1	292.734	515.862	518.4471	906.737	558.45	0.816542149440375	0.00869490114101944	0.978863622852093
ENSRNOG00000018484	Plk3	1752.15	885.305	908.0092	589.973	1033.9	-0.815613739113877	0.00579568049482952	0.978863622852093
ENSRNOG00000017307	Prss23	566.307	890.153	1041.74	1513.54	1002.9	0.810298620355237	0.00168131792842179	0.978863622852093
ENSRNOG00000042607	Rhof	341.7	169.692	167.6474	123.736	200.69	-0.809851462628201	0.0272730163804006	0.978863622852093
ENSRNOG00000005659	Aurkb	350.216	768.945	841.1441	1120.55	770.21	0.808628979966903	0.00822311823849992	0.978863622852093
ENSRNOG00000018931	Dis3l2	96.8684	185.206	249.0484	244.502	193.91	0.804602352744694	0.0208624395766675	0.978863622852093
ENSRNOG00000064232	ENSRNOG00000064232	59.6113	83.3913	107.5657	142.544	98.278	0.804386310908288	0.0483741905714561	0.978863622852093
ENSRNOG00000015308	Pbk	126.674	264.719	266.4915	417.733	268.9	0.80372765881499	0.0246627766632507	0.978863622852093
ENSRNOG00000030160	Zfp819	256.541	141.571	138.5756	90.0798	156.69	-0.798189872425299	0.0381785004799438	0.978863622852093
ENSRNOG00000024849	Tor1aip2	3338.23	1695.95	1674.536	1222.51	1982.8	-0.796881310703444	0.00363619348744745	0.978863622852093
ENSRNOG00000061474	Ctdp1	5456.56	2883.79	2795.738	2005.51	3285.4	-0.796515139681658	0.00257195242344193	0.978863622852093
ENSRNOG00000015520	ENSRNOG00000015520	266.122	540.104	600.8172	798.839	551.47	0.794470381368054	0.0090415919165354	0.978863622852093
ENSRNOG00000014041	Cep78	207.575	415.987	502.9422	579.084	426.4	0.793482051305086	0.00941840206235563	0.978863622852093

ENSRNOG00000012739	Brf2	1129.42	649.676	642.4868	384.076	701.42	-0.792654638300529	0.00753832620641161	0.978863622852093
ENSRNOG00000011332	Clspn	335.314	673.918	683.1873	1064.13	689.14	0.790817360938421	0.0103505264935502	0.978863622852093
ENSRNOG00000007286	Mdm1	294.863	580.83	657.0227	858.233	597.74	0.789802861368775	0.00756710175019304	0.978863622852093
ENSRNOG00000047741	E2f2	98.9973	255.992	259.7081	354.38	242.27	0.788211894252885	0.0404027129470953	0.978863622852093
ENSRNOG00000064281	ENSRNOG00000064281	210.769	144.48	127.9159	78.2011	140.34	-0.783805152645213	0.0390901665665321	0.978863622852093
ENSRNOG00000046428	Lrrc75b	150.093	307.384	305.2539	483.065	311.45	0.783184538037462	0.0239587642935182	0.978863622852093
ENSRNOG00000002750	Rc3h1	2768.73	1603.83	1397.385	1143.32	1728.3	-0.782923439690575	0.00154497946226223	0.978863622852093
ENSRNOG00000025704	Yod1	536.502	345.201	299.4395	212.826	348.49	-0.78234873402346	0.00805706290447086	0.978863622852093
ENSRNOG00000015382	Arid5a	860.106	358.776	424.4483	284.098	481.86	-0.781666709583938	0.023709724646679	0.978863622852093
ENSRNOG00000032917	Zfand2a	64811.3	32239.5	34363.84	22263.6	38420	-0.777222558849611	0.00473928605642052	0.978863622852093
ENSRNOG00000026951	Susd5	2767.67	4651.49	4838.517	7862.68	5030.1	0.775476760101201	0.00287056153463431	0.978863622852093
ENSRNOG00000012886	Maff	10257.4	5830.6	5537.209	3874.42	6374.9	-0.773372672225969	0.00210645227366442	0.978863622852093
ENSRNOG00000032446	Recql4	352.345	538.165	639.5796	881	602.77	0.770881524656401	0.00351444065673597	0.978863622852093
ENSRNOG00000013647	Polm	327.862	812.58	804.3198	1140.35	771.28	0.768908659349204	0.0194126119306647	0.978863622852093

ENSRNOG00000067725	Junb	3203.04	1774.49	1680.35	1241.32	1974.8	-0.768344947493203	0.00308152185760086	0.978863622852093
ENSRNOG00000060356	Kif15	226.736	552.71	531.0449	797.85	527.08	0.768338183368491	0.0248844142674243	0.978863622852093
ENSRNOG00000016791	Chka	3800.22	1907.33	1998.202	1352.19	2264.5	-0.768286864307994	0.00615514885563538	0.978863622852093
ENSRNOG00000069555	ENSRNOG00000069555	697.239	1382.74	1406.106	2130.24	1404.1	0.765101999452494	0.00784545829560908	0.978863622852093
ENSRNOG00000011873	Atg14	1734.05	1030.75	947.7407	681.043	1098.4	-0.7629046752987	0.00351106868680249	0.978863622852093
ENSRNOG00000071108	Hoxd8	248.026	407.26	483.561	625.609	441.11	0.757883525677305	0.00705850249045877	0.978863622852093
ENSRNOG00000018294	Hspa5	70463.7	34305.8	36308.74	25666.8	41686	-0.757432966463635	0.00517465286307454	0.978863622852093
ENSRNOG00000024365	Ect2	477.955	964.818	965.1838	1474.93	970.72	0.757303262127746	0.0111159159927415	0.978863622852093
ENSRNOG00000010744	Nrp1	152.222	266.658	267.4606	440.5	281.71	0.755345991157916	0.0253587324608392	0.978863622852093
ENSRNOG00000016103	Nkd2	237.381	561.437	564.962	783.991	536.94	0.754534656410527	0.0229038781061949	0.978863622852093
ENSRNOG00000049517	Tnfaip3	1247.58	595.375	666.7133	425.652	733.83	-0.753879881961869	0.016462934191121	0.978863622852093
ENSRNOG00000007793	Pnrc1	1335.93	769.915	721.9497	528.6	839.1	-0.751233256826773	0.0055545431255183	0.978863622852093
ENSRNOG00000011937	Sgtb	771.753	611.859	502.9422	320.724	551.82	-0.747721985877128	0.00451824727340421	0.978863622852093
ENSRNOG00000001825	Pkp2	118.158	129.935	176.3689	239.553	166	0.744723490245823	0.0235146450755511	0.978863622852093

ENSRNOG00000068766	ENSRNOG00000068766	74.5141	103.754	130.8231	168.281	119.34	0.744630257050941	0.0483097411378847	0.978863622852093
ENSRNOG00000034066	Hspa8	125417	66936	69560.1	45391.3	76826	-0.742722711884494	0.00487693065756539	0.978863622852093
ENSRNOG00000004006	Dnajb9	3608.61	2045.03	1897.42	1483.84	2258.7	-0.74135818733863	0.00304380509489839	0.978863622852093
ENSRNOG00000009946	Ldlr	10003	6114.71	5684.506	3957.57	6439.9	-0.741136599698317	0.00213949341008144	0.978863622852093
ENSRNOG00000014338	Slc25a25	4627.33	2236.05	2405.207	1703.6	2743	-0.739982669441358	0.00837307799842212	0.978863622852093
ENSRNOG00000005573	Ntn4	252.284	480.954	544.6117	681.043	489.72	0.739769964995385	0.0123663950604427	0.978863622852093
ENSRNOG00000000121	Pigv	100.062	120.239	193.812	174.22	147.08	0.73942315126685	0.0269489230465989	0.978863622852093
ENSRNOG00000013167	Hmgb2l1	559.92	942.515	958.4004	1547.19	1002	0.737157919327046	0.00816599906665418	0.978863622852093
ENSRNOG00000019559	Eaf1	13148.5	7000.99	7289.27	4822.73	8065.4	-0.734232755169744	0.00572210728332949	0.978863622852093
ENSRNOG00000048411	Uhrf1	2307.81	4838.63	4943.175	6942.08	4757.9	0.733683027146312	0.00833191241077555	0.978863622852093
ENSRNOG00000058522	Fam214a	2715.51	1629.04	1563.094	1050.27	1739.5	-0.732980188907418	0.00457141356164583	0.978863622852093
ENSRNOG00000022911	Hjurp	530.115	1138.39	1096.007	1671.92	1109.1	0.729534291720607	0.0165383343309582	0.978863622852093
ENSRNOG00000048682	Zwint	13437	7264.74	7431.721	5055.36	8297.2	-0.729242754390823	0.00515349181391782	0.978863622852093
ENSRNOG00000039470	AC131360.1	77.7076	104.724	153.1115	149.473	121.25	0.728328541163386	0.0457470058875602	0.978863622852093

ENSRNOG00000010121	Lef1	91.5459	81.4519	126.9469	159.372	114.83	0.727356307959482	0.0495953597548697	0.978863622852093
ENSRNOG00000048838	Gjc1	218.22	432.471	418.6339	658.275	431.9	0.725383868969155	0.0256161580771416	0.978863622852093
ENSRNOG00000010183	Gask1b	1049.58	1949.03	1985.604	2965.7	1987.5	0.723097311164787	0.00784992753399797	0.978863622852093
ENSRNOG00000057794	Adamts5	127.738	268.597	258.739	394.965	262.51	0.719800620693543	0.0442971948957529	0.978863622852093
ENSRNOG00000000420	Nelfe	209.704	328.717	317.8517	568.196	356.12	0.717344894519806	0.0258427394368319	0.978863622852093
ENSRNOG00000004493	Tasp1	105.384	125.087	183.1523	195.998	152.41	0.717183252501544	0.0288659333456891	0.978863622852093
ENSRNOG00000000632	Cdk1	965.49	1797.76	1901.296	2637.06	1825.4	0.715338276983228	0.00732490371161622	0.978863622852093
ENSRNOG00000067723	Pclaf	236.316	471.258	441.8914	720.638	467.53	0.71495123934112	0.0282982621989032	0.978863622852093
ENSRNOG00000012864	Ern1	1977.82	1172.33	1109.574	810.718	1267.6	-0.713664350412506	0.00554316565892234	0.978863622852093
ENSRNOG00000051548	Lmod1	1633.99	3114.57	3370.391	4417.87	3134.2	0.713522271762836	0.006178666383226	0.978863622852093
ENSRNOG00000070643	ENSRNOG00000070643	2471.74	1504.92	1271.407	1153.22	1600.3	-0.713456946191842	0.00245135176764169	0.978863622852093
ENSRNOG00000020322	Asb1	431.117	277.324	244.2031	188.079	285.18	-0.711497745926703	0.0184229744230121	0.978863622852093
ENSRNOG00000028415	Cdc20	364.055	792.217	744.2381	1146.29	761.7	0.708332751801448	0.0249943090199543	0.978863622852093
ENSRNOG00000043225	Zfp771	1200.74	787.369	701.5995	514.742	801.11	-0.708261590201319	0.00526752730431765	0.978863622852093

ENSRNOG00000033169	Cpeb4	6765.88	3837.94	3741.541	2748.92	4273.6	-0.708048225832932	0.00453435449600763	0.978863622852093
ENSRNOG00000026502	Dscc1	197.995	390.775	431.2317	529.59	387.4	0.704883197288056	0.0233502204999664	0.978863622852093
ENSRNOG00000028624	Kif26b	982.522	1597.04	1900.327	2287.63	1691.9	0.698628091765102	0.0031064320386074	0.978863622852093
ENSRNOG00000011815	Sgk1	1548.83	808.701	863.4325	589.973	952.73	-0.697281734850632	0.0154908378235256	0.978863622852093
ENSRNOG00000009681	Flot2	359.797	629.313	711.2901	892.879	648.32	0.6965195860284	0.0105606799257547	0.978863622852093
ENSRNOG00000014786	Ccne1	336.378	631.253	551.3952	1017.6	634.16	0.69633790454223	0.0299427561092557	0.978863622852093
ENSRNOG00000027787	Cdc6	328.927	674.888	604.6935	1022.55	657.77	0.695905822422953	0.0301994743185606	0.978863622852093
ENSRNOG00000013774	Lmnb1	421.537	853.306	776.2171	1289.82	835.22	0.695692871570668	0.025097203647722	0.978863622852093
ENSRNOG00000012181	Lpl	227.8	327.747	361.4594	538.499	363.88	0.694705362958848	0.0165042022515585	0.978863622852093
ENSRNOG00000056716	Zbtb20	428.988	505.196	259.7081	317.754	377.91	-0.694551731315849	0.00570253105231376	0.978863622852093
ENSRNOG00000064475	ENSRNOG00000064475	646.144	433.441	342.0782	324.683	436.59	-0.694419931693698	0.00771469957347968	0.978863622852093
ENSRNOG00000037225	Tyms	296.992	599.254	563.9929	886.94	586.79	0.693850539577899	0.0277361180796927	0.978863622852093
ENSRNOG00000036682	Pycr1	156.48	323.868	313.0064	464.257	314.4	0.692452633673822	0.0429297914566236	0.978863622852093
ENSRNOG00000038035	Kif4a	562.049	1035.6	1000.07	1582.83	1045.1	0.692336774248974	0.0159732463105	0.978863622852093

ENSRNOG00000047499	Rbm15	1260.35	631.253	662.8371	507.812	765.56	-0.691626389312388	0.0177559354469392	0.978863622852093
ENSRNOG00000039284	Haus4	510.954	912.456	987.4722	1312.59	930.87	0.691497802583281	0.010201666046327	0.978863622852093
ENSRNOG00000010079	Ca3	5377.79	8110.29	8905.662	12865.6	8814.8	0.690647987481695	0.00265025123136602	0.978863622852093
ENSRNOG00000012051	Ncaph	484.342	1005.54	956.4622	1448.21	973.64	0.689847585433301	0.0221325182175847	0.978863622852093
ENSRNOG00000054286	Rrm2	3420.2	6644.15	6543.093	9682.09	6572.4	0.688846387117574	0.0105286085252525	0.978863622852093
ENSRNOG00000000529	Pim1	813.268	419.865	471.9322	293.007	499.52	-0.687995431123673	0.0310884546524162	0.978863622852093
ENSRNOG00000049033	Racgap1	1032.55	2140.05	2080.572	3032.03	2071.3	0.68797843971226	0.016037244852212	0.978863622852093
ENSRNOG00000071119	ENSRNOG00000071119	6274.09	3461.71	3618.47	2424.24	3944.6	-0.687951621121705	0.00879781403023828	0.978863622852093
ENSRNOG00000004242	Pole2	163.931	245.325	311.0683	347.451	266.94	0.684579323910876	0.019225508657636	0.978863622852093
ENSRNOG00000060100	Knl1	955.91	1898.61	1762.72	2818.21	1858.9	0.68196518168153	0.0183811428747229	0.978863622852093
ENSRNOG00000051680	Ccdc186	14528.1	8447.73	8707.004	5624.54	9326.9	-0.680858958771648	0.00764053622370129	0.978863622852093
ENSRNOG00000011167	Wdhd1	441.762	777.672	767.4955	1187.87	793.7	0.680356677245829	0.0172017958540736	0.978863622852093
ENSRNOG00000067231	ENSRNOG00000067231	976.135	461.561	561.0858	336.562	583.84	-0.678715795962907	0.0389708800692176	0.978863622852093
ENSRNOG00000043105	Lyrm2	240.574	430.532	410.8815	664.215	436.55	0.678512729907474	0.0307642159656371	0.978863622852093

ENSRNOG00000025764	AC128848.1	554.598	376.23	281.0274	300.926	378.2	-0.676827902523073	0.0114070254914227	0.978863622852093
ENSRNOG00000009173	Smad6	171.382	294.778	295.5633	450.399	303.03	0.676581143505781	0.0360326844888734	0.978863622852093
ENSRNOG00000007029	Dnaja1	18975.6	9738.36	10651.91	7322.2	11672	-0.675775106764353	0.0112753223303741	0.978863622852093
ENSRNOG00000029510	Plxnb1	253.348	336.474	367.2737	575.125	383.06	0.675058001753197	0.0182135456107138	0.978863622852093
ENSRNOG00000014061	Dusp5	3571.36	1873.39	2092.201	1317.54	2213.6	-0.674943341476603	0.016313476386204	0.978863622852093
ENSRNOG00000033433	Csrnp1	3781.06	2015.94	2252.096	1380.89	2357.5	-0.67390546608481	0.0164213460269395	0.978863622852093
ENSRNOG00000056174	ENSRNOG00000056174	125.61	173.57	193.812	283.108	194.02	0.67115060545734	0.0427048325475799	0.978863622852093
ENSRNOG00000058186	Errfi1	3297.78	2095.45	1983.666	1404.65	2195.4	-0.670325195956317	0.00560818620011828	0.978863622852093
ENSRNOG00000008297	Oser1	9987.02	5398.13	5616.672	4052.6	6263.6	-0.669959068334351	0.00900048100114999	0.978863622852093
ENSRNOG00000002141	Cd200	144.77	270.537	306.223	355.37	269.22	0.669735821821006	0.0375372079579761	0.978863622852093
ENSRNOG00000070904	ENSRNOG00000070904	517.341	299.627	295.5633	217.775	332.58	-0.66918618092211	0.0301282649573109	0.978863622852093
ENSRNOG00000019861	Tollip	11746.6	6576.27	6740.782	4793.04	7464.2	-0.667692999053327	0.00798442614591156	0.978863622852093
ENSRNOG00000017728	Edrf1	3291.4	1777.4	1894.512	1299.72	2065.8	-0.665889002050209	0.0133964659996287	0.978863622852093
ENSRNOG00000020063	Nfkbib	1512.64	878.517	843.0822	665.205	974.86	-0.664254018405555	0.0106646446087282	0.978863622852093

ENSRNOG00000052498	Grb14	618.467	1154.87	1065.966	1744.18	1145.9	0.663543456780907	0.022519660268096	0.978863622852093
ENSRNOG00000014080	Kif23	572.694	875.608	919.638	1372.97	935.23	0.661942647296467	0.0106828306368681	0.978863622852093
ENSRNOG00000015423	Ccna2	631.241	1374.02	1272.376	1901.57	1294.8	0.661874816599968	0.0283197849353722	0.978863622852093
ENSRNOG00000042826	Zfp52	434.311	254.052	263.5843	171.251	280.8	-0.661405066468558	0.0442190808123291	0.978863622852093
ENSRNOG00000062764	ENSRNOG00000062764	931.427	517.802	534.9211	381.107	591.31	-0.661010053203321	0.0224530989102766	0.978863622852093
ENSRNOG00000068822	ENSRNOG00000068822	674.885	369.443	394.4074	266.28	426.25	-0.659507302579139	0.033488515191345	0.978863622852093
ENSRNOG00000018214	Bok	413.021	694.281	750.0525	998.797	714.04	0.658385971459444	0.0139840270616248	0.978863622852093
ENSRNOG00000016706	Fanca	236.316	501.317	459.3345	705.79	475.69	0.658129025742559	0.0449128646471806	0.978863622852093
ENSRNOG00000064142	ENSRNOG00000064142	466.246	929.91	919.638	1283.88	899.92	0.657500628089133	0.0236388963550989	0.978863622852093
ENSRNOG00000051615	Hmgn2	451.343	845.549	804.3198	1241.32	835.63	0.656642797922181	0.0246891137859183	0.978863622852093
ENSRNOG00000001979	Rcan1	4158.95	2409.62	2493.391	1673.9	2684	-0.656251856930862	0.0110867843329535	0.978863622852093
ENSRNOG00000027035	Sgo2	388.538	590.527	617.2912	924.555	630.23	0.654264542273315	0.0157313281128008	0.978863622852093
ENSRNOG00000023546	Hspb1	31785.6	18248.1	18566.22	13233.8	20458	-0.653841598648888	0.00772150002052134	0.978863622852093
ENSRNOG00000021903	Atad5	257.606	507.135	497.1278	706.78	492.16	0.653332746230452	0.0347333806332269	0.978863622852093

ENSRNOG00000068394	ENSRNOG00000068394	2572.87	1483.59	1489.445	1089.87	1658.9	-0.652888788247696	0.010961650199162	0.978863622852093
ENSRNOG00000011346	Ehd2	689.788	1324.56	1296.602	1871.88	1295.7	0.652865217063971	0.0197437314804616	0.978863622852093
ENSRNOG00000005115	Asf1b	343.829	562.406	584.3432	841.405	583	0.652692304899783	0.0190641084382059	0.978863622852093
ENSRNOG00000015895	B4galt6	380.022	541.074	618.2603	830.516	592.47	0.652486485618657	0.0101808349210749	0.978863622852093
ENSRNOG00000021007	Snx15	3282.88	1849.15	1995.295	1276.96	2101.1	-0.648976086138123	0.0162027890497963	0.978863622852093
ENSRNOG00000006921	Rbl1	516.276	905.668	894.4424	1334.37	912.69	0.647631344723359	0.019644519080163	0.978863622852093
ENSRNOG00000006867	Etv1	359.797	531.377	633.7653	761.224	571.54	0.645443993671283	0.00998209593928244	0.978863622852093
ENSRNOG00000006198	Prr11	180.963	260.84	250.9865	440.5	283.32	0.644982788416948	0.0477277006296052	0.978863622852093
ENSRNOG00000010706	Ccdc117	1562.67	802.883	829.5154	683.023	969.52	-0.644646816093585	0.020096963245956	0.978863622852093
ENSRNOG00000054561	Isg20	1405.12	829.064	854.7109	574.135	915.76	-0.644341678086184	0.0183067669491382	0.978863622852093
ENSRNOG00000058539	Ccnb1	424.731	736.946	644.4249	1172.03	744.53	0.644103120237869	0.034550435944992	0.978863622852093
ENSRNOG00000016182	Tgfa	1901.17	3045.72	3394.617	4334.72	3169.1	0.643554799880939	0.00539664355289572	0.978863622852093
ENSRNOG00000006759	Ankrd24	420.473	644.828	673.4967	990.878	682.42	0.642838727995903	0.0161159304212303	0.978863622852093
ENSRNOG00000021137	Kctd15	828.171	1503.95	1452.621	2188.64	1493.3	0.642278561116084	0.0189093348168739	0.978863622852093

ENSRNOG00000069117	Mmgt2	252.284	362.655	409.9124	548.398	393.31	0.638836117211088	0.0197259247594604	0.978863622852093
ENSRNOG00000067338	Maml3	229.929	387.866	471.9322	490.984	395.18	0.638799054705099	0.0230035485144775	0.978863622852093
ENSRNOG00000007415	Ptgs1	446.02	743.734	695.7851	1156.19	760.43	0.637584105149265	0.0269255238688043	0.978863622852093
ENSRNOG00000042340	ENSRNOG00000042340	382.151	216.236	200.5954	184.119	245.78	-0.635927167301694	0.0483225043706694	0.978863622852093
ENSRNOG00000008118	Sync	688.723	1166.51	1317.922	1565.01	1184.5	0.63527156220424	0.0104650787109003	0.978863622852093
ENSRNOG00000018815	Plk1	578.017	941.546	897.3496	1463.05	969.99	0.634713131373955	0.0217640827053321	0.978863622852093
ENSRNOG00000026647	Cxcl16	416.215	287.021	256.8009	195.998	289.01	-0.634044988721092	0.0305912628992892	0.978863622852093
ENSRNOG00000058388	Zfp36	2620.77	1491.35	1521.424	1131.44	1691.2	-0.631986831609843	0.0139321650232144	0.978863622852093
ENSRNOG00000029055	Ttk	487.535	890.153	945.8026	1188.86	878.09	0.630888669214044	0.0195126436852098	0.978863622852093
ENSRNOG00000068687	Ubxn2a	3708.67	2266.11	2237.56	1630.35	2460.7	-0.627096412166808	0.0100032624493019	0.978863622852093
ENSRNOG00000004118	Frmpd4	250.155	372.352	431.2317	530.58	396.08	0.626370878418102	0.0207118067023942	0.978863622852093
ENSRNOG00000025142	Mex3b	328.927	590.527	547.5189	872.091	584.77	0.625572279508402	0.0376658712369567	0.978863622852093
ENSRNOG00000060773	Sertad4	1538.18	2253.5	2705.616	3144.87	2410.5	0.625390418681269	0.00340802241522608	0.978863622852093
ENSRNOG00000063477	ENSRNOG00000063477	188.414	266.658	318.8207	383.087	289.24	0.623776007299765	0.0279784461750718	0.978863622852093

ENSRNOG00000052407	ENSRNOG00000052407	659.982	997.786	1153.181	1398.71	1052.4	0.621653890753411	0.00813273934968348	0.978863622852093
ENSRNOG00000063594	ENSRNOG00000063594	175.64	248.234	277.1512	375.167	269.05	0.620506208959202	0.037355935271632	0.978863622852093
ENSRNOG00000012835	Espl1	642.95	1230.51	1176.439	1704.59	1188.6	0.62023184735941	0.0275648656838781	0.978863622852093
ENSRNOG00000007576	Zfp597	326.798	218.175	188.9667	165.311	224.81	-0.620067420773718	0.0448614076266159	0.978863622852093
ENSRNOG00000029535	Nrbp2	315.088	554.649	550.4261	786.961	551.78	0.619603508656397	0.0323909538002276	0.978863622852093
ENSRNOG00000021579	Mpp6	290.605	545.922	493.2516	792.9	530.67	0.61940006330207	0.0467617819046516	0.978863622852093
ENSRNOG00000007587	Tcp11l2	1513.7	1072.45	962.2766	721.628	1067.5	-0.618554061707894	0.00820288311200306	0.978863622852093
ENSRNOG00000002926	Uap1	3455.33	1971.33	2000.14	1536.31	2240.8	-0.617496900181675	0.0136782807939661	0.978863622852093
ENSRNOG00000000479	Kifc1	480.084	932.819	899.2877	1269.04	895.31	0.61708419070282	0.0319237671491436	0.978863622852093
ENSRNOG00000065264	Sox12	516.276	961.909	903.1639	1365.06	936.6	0.616963081659831	0.0313706276060016	0.978863622852093
ENSRNOG00000037198	Usp18	233.123	228.841	156.0187	145.514	190.87	-0.615322196670782	0.0387309265408118	0.978863622852093
ENSRNOG00000002418	Tgfb2	4731.65	6974.81	7905.592	10009.7	7405.4	0.613780066439293	0.00401830614341185	0.978863622852093
ENSRNOG00000011800	F3	893.105	1599.95	1801.483	2011.45	1576.5	0.612456479837813	0.0141274029775266	0.978863622852093
ENSRNOG00000058039	Acta2	53566.1	78173.5	90016.95	111281	83259	0.611635269921121	0.00304014099883567	0.978863622852093

ENSRNOG00000009683	Sdcbp	30444.3	17157.3	17918.89	13237.8	19690	-0.611436619197713	0.012628844615491	0.978863622852093
ENSRNOG00000049215	Cbx2	242.703	448.955	474.8394	582.054	437.14	0.61024064273036	0.038756126918363	0.978863622852093
ENSRNOG00000004165	Prkd1	378.958	777.672	814.9795	950.292	730.48	0.6089318625654	0.03581499044252	0.978863622852093
ENSRNOG00000009656	Rspo1	287.412	551.74	514.5709	766.173	529.97	0.608754264414617	0.0473282662132965	0.978863622852093
ENSRNOG00000020140	Pigq	353.41	543.013	560.1167	807.748	566.07	0.608664909067509	0.0252182588622008	0.978863622852093
ENSRNOG00000003031	Atp2b4	713.207	515.862	503.9112	301.916	508.72	-0.608311989431767	0.0314107944248081	0.978863622852093
ENSRNOG00000021962	Fzd2	1098.55	1769.64	1784.04	2589.55	1810.4	0.608264440511031	0.0150974670507183	0.978863622852093
ENSRNOG00000054217	Runx3	234.187	428.592	476.7775	534.539	418.52	0.608135821515302	0.037216846695818	0.978863622852093
ENSRNOG00000070046	Cdc45	258.67	444.107	453.5201	618.68	443.74	0.608085517149296	0.0368664033722016	0.978863622852093
ENSRNOG00000063116	ENSRNOG00000063116	624.854	523.62	436.077	317.754	475.58	-0.606842750563492	0.0150569830396551	0.978863622852093
ENSRNOG00000011007	Ube2o	13688.2	8489.43	8590.717	5973.97	9185.6	-0.606565746510021	0.0108974774044213	0.978863622852093
ENSRNOG00000015036	Ccn2	15418	21389.9	25953.37	30079.7	23210	0.606228583004492	0.00184901369710183	0.978863622852093
ENSRNOG00000045829	Thbs1	44190.1	76739.4	80370.93	103706	76251	0.606128602125608	0.010517420265956	0.978863622852093
ENSRNOG00000060949	Anxa8	290.605	354.898	415.7268	567.206	407.11	0.605881214392745	0.0196214115114571	0.978863622852093

ENSRNOG00000024809	Ntmt1	4104.66	2338.83	2466.258	1766.95	2669.2	-0.605867547854385	0.0173072503311204	0.978863622852093
ENSRNOG00000002607	Sox9	197.995	201.691	250.0175	358.339	252.01	0.605759032533903	0.0381141618986213	0.978863622852093
ENSRNOG00000006789	Ddit3	3440.42	1870.49	2080.572	1410.59	2200.5	-0.6049833082024	0.0243027532763343	0.978863622852093
ENSRNOG00000020298	Bag3	35747.6	19712.3	20974.34	15531.3	22991	-0.603297027683171	0.0150606538871436	0.978863622852093
ENSRNOG00000022414	Zfp142	7311.96	4740.7	4663.117	3270.59	4996.6	-0.603166701627894	0.00982583268962123	0.978863622852093
ENSRNOG00000004861	Itga4	227.8	255.992	364.3666	370.218	304.59	0.602021001766523	0.0187587789591121	0.978863622852093
ENSRNOG00000008445	Dact1	588.662	829.064	925.4523	1226.47	892.41	0.601377881788837	0.0113417113188853	0.978863622852093
ENSRNOG00000061139	Taf1a	1307.19	701.069	705.4757	617.69	832.86	-0.60132487584864	0.0268412012051756	0.978863622852093
ENSRNOG00000031993	Prim1	1365.74	2453.25	2401.331	3393.34	2403.4	0.601192990678521	0.0206837681812997	0.978863622852093
ENSRNOG00000032546	Dot1l	63889.5	42352.1	42069.8	27972.3	44071	-0.601041311183775	0.00966465345867043	0.978863622852093
ENSRNOG00000012439	Bid	1910.76	1354.62	1263.654	890.899	1355	-0.599501963080042	0.0109346870528125	0.978863622852093
ENSRNOG00000069827	ENSRNOG00000069827	500.309	388.836	344.0163	242.523	368.92	-0.599399889919482	0.0280020037207043	0.978863622852093
ENSRNOG00000002545	RGD1309748	4812.55	2753.85	2887.799	2109.45	3140.9	-0.598278026982426	0.0170369144784672	0.978863622852093
ENSRNOG00000004667	Gen1	230.994	372.352	412.8196	500.883	379.26	0.597294748762798	0.0344643422499728	0.978863622852093

ENSRNOG00000016774	Telo2	263.993	379.139	439.9533	533.55	404.16	0.596867010550918	0.0240149602561211	0.978863622852093
ENSRNOG00000053362	Gabarapl1	23483.7	15668.8	15582.49	10307.7	16261	-0.596662521919215	0.0104933165106493	0.978863622852093
ENSRNOG00000010150	Strbp	464.117	771.854	791.722	1077.99	776.42	0.596292781118825	0.0248563180091321	0.978863622852093
ENSRNOG00000009694	Bmp4	485.406	705.917	833.3916	968.11	748.21	0.59580682285424	0.0117304414007814	0.978863622852093
ENSRNOG00000023991	Rab20	1103.87	674.888	683.1873	493.954	738.98	-0.594920408526579	0.0259255750504778	0.978863622852093
ENSRNOG00000019463	Mad2l1bp	1777.69	909.547	1084.378	694.901	1116.6	-0.594343232423238	0.0427281179247294	0.978863622852093
ENSRNOG00000014721	Ahrr	571.63	1112.21	1121.202	1420.49	1056.4	0.593292663383833	0.031789112823657	0.978863622852093
ENSRNOG00000016620	Csnk1g1	727.045	556.588	430.2627	420.702	533.65	-0.592496956950712	0.0110835258234528	0.978863622852093
ENSRNOG00000001314	Fam20c	786.656	1236.32	1302.417	1747.15	1268.1	0.591554047634041	0.015637197557635	0.978863622852093
ENSRNOG00000027125	Mboat1	161.802	219.145	255.8318	318.744	238.88	0.591525053003246	0.0464242232735514	0.978863622852093
ENSRNOG00000014079	Stat4	3795.96	6900.14	6804.74	9281.19	6695.5	0.588582947472067	0.0195002800598939	0.978863622852093
ENSRNOG00000009334	Knstrn	233.123	379.139	371.15	550.378	383.45	0.588511745964392	0.049479061334257	0.978863622852093
ENSRNOG00000025079	Fam126b	876.073	536.225	480.6538	459.308	588.07	-0.586622508990757	0.023986000420084	0.978863622852093
ENSRNOG00000054176	Mogs	402.376	687.493	706.4448	930.495	681.7	0.585851892380669	0.0305451846476317	0.978863622852093

ENSRNOG00000004753	Napb	2156.65	1434.14	1458.435	934.454	1495.9	-0.58520697126119	0.020748251073448	0.978863622852093
ENSRNOG00000001215	Cfap410	230.994	233.69	313.9754	383.087	290.44	0.584860553444077	0.0273834710464969	0.978863622852093
ENSRNOG00000010840	Adamtsl3	588.662	899.85	906.0711	1327.44	930.51	0.584762954881778	0.0229906219491452	0.978863622852093
ENSRNOG00000061862	Zbtb10	438.569	350.049	277.1512	248.462	328.56	-0.584620373602393	0.0245028004869798	0.978863622852093
ENSRNOG00000004218	Klhl28	562.049	368.473	348.8616	271.229	387.65	-0.584573082033292	0.0362868494934618	0.978863622852093
ENSRNOG00000003872	NEWGENE_620180	1462.61	2892.51	2686.234	3843.73	2721.3	0.584057084676861	0.0332467300686809	0.978863622852093
ENSRNOG00000019834	Hsp90ab1	126333	73477.4	75840.58	57568.9	83305	-0.58276488483968	0.0139164862076842	0.978863622852093
ENSRNOG00000006178	Dync1h1	47851.9	42573.2	35110.98	25303.5	37710	-0.581818584641819	0.00248989590431102	0.978863622852093
ENSRNOG00000009491	Phc3	2033.17	1462.26	1393.508	941.383	1457.6	-0.581785356144862	0.0144463161689946	0.978863622852093
ENSRNOG00000008898	Trmt44	1321.03	809.671	836.2988	587.993	888.75	-0.580509863456675	0.0279223228485526	0.978863622852093
ENSRNOG00000014626	Katnb1	4917.93	3172.75	3265.732	2148.06	3376.1	-0.579454360893616	0.0176557811241661	0.978863622852093
ENSRNOG00000067262	Aldh18a1	2922.02	5257.53	5345.335	6872.79	5099.4	0.578757383848843	0.0191247507429652	0.978863622852093
ENSRNOG00000013867	Fgf1	699.368	1072.45	1127.017	1519.48	1104.6	0.578232096224867	0.0181240046380968	0.978863622852093
ENSRNOG00000018239	Dhrs4	377.893	574.042	657.9918	763.204	593.28	0.577149847286661	0.021341914214954	0.978863622852093

ENSRNOG00000064308	ENSRNOG00000064308	875.009	575.012	568.8382	402.884	605.44	-0.576724747392153	0.0303444297293756	0.978863622852093
ENSRNOG00000020084	Pcdhb5	219.284	276.355	295.5633	443.47	308.67	0.575370895027987	0.0471620998430743	0.978863622852093
ENSRNOG00000049137	Zfp35	1167.74	767.975	696.7542	603.832	809.08	-0.573119743940141	0.0172965354741876	0.978863622852093
ENSRNOG00000012791	Ajuba	2126.85	3932	4104.938	4899.94	3765.9	0.571430033097662	0.0215667649012332	0.978863622852093
ENSRNOG00000039859	Dnmt1	1461.54	2440.65	2481.763	3316.12	2425	0.570910267729544	0.0192227815126736	0.978863622852093
ENSRNOG00000066092	ENSRNOG00000066092	2286.52	1354.62	1346.993	1103.72	1523	-0.570810490352242	0.0200280437887782	0.978863622852093
ENSRNOG00000006947	Pdhx	2843.25	1712.43	1785.978	1281.9	1905.9	-0.570119087885063	0.0225625711635843	0.978863622852093
ENSRNOG00000007164	Cln6	374.7	719.492	704.5066	919.606	679.58	0.568742799525608	0.0480868014432805	0.978863622852093
ENSRNOG00000000572	Chst3	267.186	399.502	431.2317	558.297	414.05	0.568496148752033	0.0371392408461751	0.978863622852093
ENSRNOG00000040205	Zcchc24	644.015	1322.62	1273.345	1643.21	1220.8	0.567881665816838	0.0449811556845682	0.978863622852093
ENSRNOG00000063720	ENSRNOG00000063720	893.105	1147.11	1386.725	1636.28	1265.8	0.566820728167371	0.00628100902554486	0.978863622852093
ENSRNOG00000004606	Meis1	2126.85	2998.21	3357.793	4227.81	3177.7	0.565465608265463	0.00749964728876689	0.978863622852093
ENSRNOG00000052894	Epg5	3297.78	2445.5	2228.838	1653.11	2406.3	-0.564883265365041	0.00888286523556674	0.978863622852093
ENSRNOG00000019681	Pold1	467.31	894.032	851.8038	1162.13	843.82	0.564120188639741	0.0471095410031771	0.978863622852093

ENSRNOG00000017932	St3gal2	821.784	1461.29	1445.838	1930.28	1414.8	0.56383856085417	0.0303850828518365	0.978863622852093
ENSRNOG00000019202	Pvr	11850.9	6933.11	7468.546	5246.41	7874.8	-0.562902108395613	0.0221706432667045	0.978863622852093
ENSRNOG00000003861	Gorab	2205.62	1411.83	1422.58	1031.46	1517.9	-0.559452559945836	0.0216993418787557	0.978863622852093
ENSRNOG00000020736	Nadsyn1	210.769	339.383	385.6859	425.652	340.37	0.558956248594019	0.0493622642970196	0.978863622852093
ENSRNOG00000011498	Psip1	1007.01	1576.68	1581.506	2225.27	1597.6	0.55869124488024	0.0225700955548348	0.978863622852093
ENSRNOG00000015442	Sfxn3	900.556	1524.31	1616.392	1955.03	1499.1	0.55806132619917	0.0226647377137673	0.978863622852093
ENSRNOG00000025679	Stk40	1807.5	1207.23	1151.243	896.838	1265.7	-0.557344388155784	0.0174435527056812	0.978863622852093
ENSRNOG00000027784	Tsku	1302.93	2011.09	2088.324	2789.5	2048	0.557290237767501	0.0173773228162736	0.978863622852093
ENSRNOG00000058560	Col2a1	3692.71	5283.71	5798.855	7405.35	5545.2	0.556647405271474	0.00838493232719476	0.978863622852093
ENSRNOG00000026880	Usp38	4660.33	2981.72	2970.169	2227.25	3209.9	-0.555979500396326	0.0163519571101456	0.978863622852093
ENSRNOG00000010799	Noct	989.973	575.982	576.5907	488.015	657.64	-0.555977176522566	0.0375410381262027	0.978863622852093
ENSRNOG00000039902	Lbh	327.862	476.106	539.7664	642.437	496.54	0.555199232130753	0.0288297372412429	0.978863622852093
ENSRNOG00000022356	Zc2hc1a	459.859	830.034	844.0513	1052.25	796.55	0.555029864147707	0.0401875571039432	0.978863622852093
ENSRNOG00000013851	Spry4	2324.84	1198.51	1290.788	1107.68	1480.5	-0.554437101513987	0.0373801637484244	0.978863622852093

ENSRNOG00000014589	Arpin	334.249	486.772	510.6946	695.891	506.9	0.55441502349047	0.0360448155759637	0.978863622852093
ENSRNOG00000066630	Fam32a	4878.55	2600.64	2867.449	2225.27	3143	-0.554247378546186	0.0312056054094347	0.978863622852093
ENSRNOG00000037113	Slfn2	3506.42	2084.78	2082.51	1726.36	2350	-0.553537980307136	0.0201396187185207	0.978863622852093
ENSRNOG00000017579	Mylip	814.333	606.041	494.2206	473.166	596.94	-0.553525311675043	0.0168723572410931	0.978863622852093
ENSRNOG00000003075	Tmem39a	3483	2041.15	2155.189	1609.56	2322.2	-0.552940949512763	0.0258320101421425	0.978863622852093
ENSRNOG00000054725	Gtf3c4	7691.99	4994.75	5027.483	3623.98	5334.5	-0.552192250156444	0.0164090743294585	0.978863622852093
ENSRNOG00000011631	Fst	2016.14	2439.68	2915.902	3618.04	2747.4	0.552073823232253	0.00430363826592242	0.978863622852093
ENSRNOG00000070392	Spty2d1	3314.81	2192.41	1972.037	1783.78	2315.8	-0.551957731213472	0.0109263779493124	0.978863622852093
ENSRNOG00000004766	Glis2	470.503	614.768	686.0945	905.747	669.28	0.551938007504514	0.0205694605256193	0.978863622852093
ENSRNOG00000028211	Akap17a	3194.53	2225.38	2078.634	1620.45	2279.7	-0.550862654682383	0.0119951847581209	0.978863622852093
ENSRNOG00000003809	ENSRNOG00000003809	7353.48	4438.16	4550.706	3508.16	4962.6	-0.548988520971415	0.0194854875797079	0.978863622852093
ENSRNOG00000047314	Tk1	985.715	1595.1	1551.465	2225.27	1589.4	0.54885519614452	0.0291136709731711	0.978863622852093
ENSRNOG00000033522	Prdm2	6376.28	4092.96	4015.785	3140.91	4406.5	-0.548651860502399	0.0147774680197051	0.978863622852093
ENSRNOG00000042389	Bcl10	1764.92	1069.54	1091.162	846.354	1193	-0.548405894929856	0.0283881956120944	0.978863622852093

ENSRNOG00000017488	Snx24	378.958	500.348	606.6316	680.053	541.5	0.54837855893693	0.0198970302221167	0.978863622852093
ENSRNOG00000059348	Ube2b	5241.54	3418.07	3269.609	2651.91	3645.3	-0.548169980781949	0.0131422247467553	0.978863622852093
ENSRNOG00000009225	Copz2	353.41	548.831	542.6736	777.062	555.49	0.547645751069235	0.0450329058788995	0.978863622852093
ENSRNOG00000014628	Pomk	293.799	472.227	512.6328	607.791	471.61	0.547339331881892	0.0423387528663543	0.978863622852093
ENSRNOG00000024602	Plekha7	2183.26	3506.31	3743.479	4564.37	3499.4	0.545915896498811	0.0164050536267715	0.978863622852093
ENSRNOG00000033658	Kntc1	692.981	1262.51	1201.634	1654.1	1202.8	0.545707370321676	0.0428904129561028	0.978863622852093
ENSRNOG00000010524	Cryab	9419.65	5789.88	5882.194	4537.65	6407.3	-0.545543218724356	0.01815028385191	0.978863622852093
ENSRNOG00000005287	Syne3	446.02	381.079	310.0992	256.381	348.39	-0.545453934432454	0.0325387187564945	0.978863622852093
ENSRNOG00000045913	Prdm16	324.669	547.861	585.3123	688.962	536.7	0.545218095785001	0.0438983657556744	0.978863622852093
ENSRNOG00000007097	Gpatch3	749.399	504.226	486.4681	372.198	528.07	-0.54514181236266	0.0372802663826804	0.978863622852093
ENSRNOG00000012004	Ubap1	3330.78	2142.96	2120.303	1631.34	2306.3	-0.544755200098857	0.0186880076352081	0.978863622852093
ENSRNOG00000047699	Snai2	381.087	605.072	626.9818	811.708	606.21	0.543848733095585	0.0388977432158114	0.978863622852093
ENSRNOG00000015131	Ube2c	339.572	399.502	469.0251	608.781	454.22	0.543672234788075	0.0269880891367353	0.978863622852093
ENSRNOG00000020525	Col5a3	2108.75	2977.84	3091.301	4317.89	3123.9	0.54239661901958	0.0149946459274727	0.978863622852093

ENSRNOG00000028113	Whamm	4670.97	2971.06	3095.178	2152.02	3222.3	-0.542227782842323	0.0238949863377682	0.978863622852093
ENSRNOG00000016152	Dek	1015.52	1899.58	1721.051	2525.2	1790.3	0.542213047067152	0.0468011855831699	0.978863622852093
ENSRNOG00000002171	Phldb2	4563.46	6447.31	7022.778	9011.94	6761.4	0.542171922157073	0.00947742413348018	0.978863622852093
ENSRNOG00000027491	Vldlr	9315.33	13339.7	14075.6	18908.8	13910	0.541899535126589	0.0111519417266175	0.978863622852093
ENSRNOG00000020552	Fosl1	10326.6	5734.6	6265.942	4766.31	6773.4	-0.541756503325557	0.028736439106495	0.978863622852093
ENSRNOG00000064355	Fbxl12	1126.23	749.552	729.7022	559.287	791.19	-0.540647895987766	0.0293543312145647	0.978863622852093
ENSRNOG00000021201	Txnip	1195.42	2449.38	2338.342	2963.72	2236.7	0.54033958344912	0.0480208138586211	0.978863622852093
ENSRNOG00000040287	Cyp1b1	9511.2	17689.6	18615.64	20924.2	16685	0.539604305667689	0.025795140621207	0.978863622852093
ENSRNOG00000050277	Uckl1	268.251	403.381	475.8085	500.883	412.08	0.538996181749132	0.0404619542128746	0.978863622852093
ENSRNOG00000065794	Mafk	8572.32	4564.22	5004.226	4040.72	5545.4	-0.538283913577977	0.0322817233380901	0.978863622852093
ENSRNOG00000032307	Dsel	3645.87	2543.43	2414.898	1852.08	2614.1	-0.536350865080333	0.0143745502359408	0.978863622852093
ENSRNOG00000027204	Map6	11523.1	7714.66	7725.347	5540.4	8125.9	-0.536159404620405	0.0170441779984459	0.978863622852093
ENSRNOG00000013326	Hmbox1	2706.99	1927.7	1724.927	1470.97	1957.6	-0.535981459007831	0.0110439242382343	0.978863622852093
ENSRNOG00000064955	ENSRNOG00000064955	1043.2	702.038	662.8371	541.469	737.39	-0.534592134988815	0.0284004579391367	0.978863622852093

ENSRNOG00000019466	Agpat2	450.278	616.708	651.2083	894.859	653.26	0.534290023530668	0.0314067105369379	0.978863622852093
ENSRNOG00000012514	Abhd17b	1387.03	948.333	896.3805	715.689	986.86	-0.534234724040175	0.022930097328623	0.978863622852093
ENSRNOG00000010591	Kdm5a	6980.91	5087.84	4504.191	3829.88	5100.7	-0.534073734946542	0.00719952482251968	0.978863622852093
ENSRNOG00000059878	Rad51ap1	352.345	502.287	543.6427	693.911	523.05	0.53313023043271	0.0362308676791246	0.978863622852093
ENSRNOG00000024239	Fam89b	8348.78	5708.42	5713.578	4001.13	5943	-0.532969962673601	0.0183303952802438	0.978863622852093
ENSRNOG00000001613	Zbtb11	2236.49	1262.51	1401.261	1016.61	1479.2	-0.53280941123289	0.0422272371034546	0.978863622852093
ENSRNOG00000014867	Synpo2	2166.23	4385.8	4254.174	5220.67	4006.7	0.531940915626618	0.0446060007273626	0.978863622852093
ENSRNOG00000018454	Apoe	530.115	383.988	349.8307	282.118	386.51	-0.531678560954082	0.0446123944145783	0.978863622852093
ENSRNOG00000015113	Mocos	1251.84	905.668	873.1231	619.67	912.57	-0.530862813906654	0.0278999039193629	0.978863622852093
ENSRNOG00000057713	Cav2	629.112	774.763	877.9684	1150.25	858.02	0.530298443809541	0.0177459897927958	0.978863622852093
ENSRNOG00000003300	Btg2	4249.43	2469.74	2637.781	2014.42	2842.8	-0.530152858867486	0.0308354293302159	0.978863622852093
ENSRNOG00000004085	Tcaim	476.89	503.257	375.9953	302.906	414.76	-0.529765128344845	0.0283234381086608	0.978863622852093
ENSRNOG00000020617	Mus81	609.951	772.824	899.2877	1097.79	844.96	0.529758881118168	0.0159027690821689	0.978863622852093
ENSRNOG00000070031	ENSRNOG00000070031	2798.54	1512.68	1738.494	1247.26	1824.2	-0.529674053077501	0.0470459495665852	0.978863622852093

ENSRNOG00000059552	Zkscan5	3375.49	2150.72	2265.662	1563.03	2338.7	-0.529193758123346	0.0298847202476086	0.978863622852093
ENSRNOG00000009131	Zc3h12a	851.59	501.317	476.7775	460.298	572.5	-0.529036455922956	0.0473635170496659	0.978863622852093
ENSRNOG00000008614	Zfyve1	1863.92	1237.29	1208.418	940.393	1312.5	-0.528888481595819	0.0242023342167529	0.978863622852093
ENSRNOG00000016021	Lims2	661.047	931.849	1080.502	1218.55	972.99	0.528748595924709	0.0175955709502234	0.978863622852093
ENSRNOG00000007906	Bub1b	589.726	807.732	805.2889	1211.62	853.59	0.528741232173806	0.0348665663376767	0.978863622852093
ENSRNOG00000059714	Hsp90aa1	66877.5	39107.6	40960.23	32539.6	44871	-0.528033426443802	0.023379629653312	0.978863622852093
ENSRNOG00000011417	Pde3b	342.765	510.044	547.5189	683.023	520.84	0.527943623729898	0.0408282756308318	0.978863622852093
ENSRNOG00000027914	Plscr3	465.181	684.584	749.0834	909.707	702.14	0.52793878904746	0.0298318624603018	0.978863622852093
ENSRNOG00000064112	Arl5b	1243.32	842.64	824.6701	621.65	883.07	-0.527780746644509	0.0305430200087116	0.978863622852093
ENSRNOG00000027282	Cep68	695.11	1136.45	1177.408	1464.04	1118.3	0.52760630791593	0.0325469469781856	0.978863622852093
ENSRNOG00000020279	Syt11	4683.74	3062.21	3202.743	2170.82	3279.9	-0.527387837782819	0.0274341410160966	0.978863622852093
ENSRNOG00000047386	Smg1	11576.3	9165.28	7930.787	6463.97	8784.1	-0.526925597134623	0.00533304257711281	0.978863622852093
ENSRNOG00000018183	Ubr4	20836.3	19345.8	15928.44	11960.8	17018	-0.526825787299325	0.00442379263248167	0.978863622852093
ENSRNOG00000005917	Pawr	568.436	876.578	911.8855	1171.04	881.98	0.526778967764612	0.0326781541574924	0.978863622852093

ENSRNOG00000002618	ENSRNOG00000002618	2410	3551.89	3600.058	4985.08	3636.8	0.525870586402079	0.0200805110385165	0.978863622852093
ENSRNOG00000057823	Ubc	5787.62	3525.71	3567.11	2901.36	3945.4	-0.525712957155119	0.0228272411096213	0.978863622852093
ENSRNOG00000039668	Col8a1	2427.03	3642.07	3787.087	4947.46	3700.9	0.525042589141898	0.0182797445259074	0.978863622852093
ENSRNOG00000020730	Nbr1	11395.3	8071.5	7857.139	5674.04	8249.5	-0.52466184248448	0.0153938434315685	0.978863622852093
ENSRNOG00000019868	Psma5	11342.1	7654.54	7484.051	5730.46	8052.8	-0.523546881342615	0.0157397467814328	0.978863622852093
ENSRNOG00000047692	Hells	753.657	1194.63	1251.056	1550.16	1187.4	0.523242136985691	0.0296967693810829	0.978863622852093
ENSRNOG00000002052	Ccdc80	9613.39	14007.8	14464.19	19474.1	14390	0.522777645201541	0.0155277188093914	0.978863622852093
ENSRNOG00000017621	Spns1	4810.42	3120.39	3068.044	2451.95	3362.7	-0.522620962765664	0.0194278112565359	0.978863622852093
ENSRNOG00000030034	Sox11	580.146	817.428	894.4424	1113.62	851.41	0.522189020857493	0.0251064566104303	0.978863622852093
ENSRNOG00000025269	Slc25a44	2149.2	1355.59	1378.972	1062.15	1486.5	-0.521408290540678	0.0302416856058186	0.978863622852093
ENSRNOG00000011910	Hnrnpr	712.142	1110.27	1119.264	1496.71	1109.6	0.520884450544925	0.0342838942252569	0.978863622852093
ENSRNOG00000000517	Kctd20	4439.98	2715.06	2909.118	2077.77	3035.5	-0.520620973682248	0.0323391951125292	0.978863622852093
ENSRNOG00000028156	Pld1	681.272	1021.06	1042.709	1399.7	1036.2	0.520168214752369	0.0321005014559257	0.978863622852093
ENSRNOG00000027234	Zfp367	410.892	647.737	646.363	872.091	644.27	0.519461276192428	0.0488638868497536	0.978863622852093

ENSRNOG000 00020049	Slc7a6os	3262.65	2103.21	2098.984	1644.2	2277.3	- 0.5192858 32166059	0.02393 356064 53744	0.97886 362285 2093
ENSRNOG000 00033119	Plcb4	590.791	1040.45	1027.204	1311.6	992.51	0.5190609 19263795	0.04814 550601 86519	0.97886 362285 2093
ENSRNOG000 00001114	Wipi2	3601.16	2214.72	2191.045	1868.91	2469	- 0.5182906 79933543	0.02459 297405 21327	0.97886 362285 2093
ENSRNOG000 00028103	Psmd3	17996.2	12457.3	12372.96	8911.96	12935	- 0.5167316 63614231	0.01807 344688 50134	0.97886 362285 2093
ENSRNOG000 00005464	Lgalsl	749.399	1150.02	1152.212	1566	1154.4	0.5165148 76411725	0.03474 930464 04789	0.97886 362285 2093
ENSRNOG000 00015271	Zfp418	1397.67	921.183	862.4634	759.244	985.14	- 0.5153903 71801092	0.02766 262249 75953	0.97886 362285 2093
ENSRNOG000 00020024	Taf7	587.597	422.774	393.4384	313.794	429.4	- 0.5137966 17103248	0.04845 311066 55359	0.97886 362285 2093
ENSRNOG000 00024578	Ttyh2	1880.95	1252.81	1282.066	912.677	1332.1	- 0.5134491 50191105	0.03426 334553 07223	0.97886 362285 2093
ENSRNOG000 00007752	Zmynd19	649.337	900.82	922.5451	1290.81	940.88	0.5132292 32451097	0.03267 774482 45977	0.97886 362285 2093
ENSRNOG000 00006048	Ezh2	1508.38	2402.83	2354.816	3228.02	2373.5	0.5130644 27448989	0.03223 174068 15277	0.97886 362285 2093
ENSRNOG000 00000796	Ranbp2	25773.4	17775.9	17352.96	13176.4	18520	- 0.5124189 09991988	0.01594 882747 31944	0.97886 362285 2093
ENSRNOG000 00020585	Tbxa2r	499.245	409.199	312.0373	324.683	386.29	- 0.5122420 07276962	0.03635 245398 47067	0.97886 362285 2093
ENSRNOG000 00001711	Plaat1	536.502	769.915	912.8545	948.313	791.9	0.5098433 60768022	0.02572 951510 46949	0.97886 362285 2093
ENSRNOG000 00016989	Dolk	360.861	551.74	632.7962	667.184	553.15	0.5093919 68849989	0.04251 932270 74491	0.97886 362285 2093

ENSRNOG00000037376	Mterf4	717.465	548.831	530.0758	359.329	538.93	-0.509053112918095	0.0497203255472299	0.978863622852093
ENSRNOG00000019824	Ell	4180.24	3025.36	2774.419	2289.61	3067.4	-0.508654824067104	0.013464109111261	0.978863622852093
ENSRNOG00000020519	Olfm2	306.572	351.019	399.2527	536.519	398.34	0.508371334793009	0.0465175388850156	0.978863622852093
ENSRNOG00000060707	Atg2a	4886	3361.83	3354.886	2443.04	3511.4	-0.508315043900171	0.0231067640006786	0.978863622852093
ENSRNOG00000008249	Brms1l	447.085	584.709	699.6613	767.163	624.65	0.50680029671269	0.025698757394311	0.978863622852093
ENSRNOG00000014066	Jade1	744.077	1300.32	1316.953	1588.77	1237.5	0.506601403850688	0.0454246437694509	0.978863622852093
ENSRNOG00000061499	Kmt2d	9330.23	8183.98	7113.87	5219.68	7461.9	-0.505888927908861	0.0090067234280233	0.978863622852093
ENSRNOG00000021179	Naa40	559.92	884.335	870.2159	1180.94	873.85	0.505361535529635	0.0475421740723985	0.978863622852093
ENSRNOG00000003334	Klhl21	10175.4	6651.91	6788.265	5071.2	7171.7	-0.50468301721571	0.0243794725940314	0.978863622852093
ENSRNOG00000045752	Rrm1	4625.2	6964.14	7129.375	9311.87	7007.6	0.504408682442716	0.0215883459868474	0.978863622852093
ENSRNOG00000015796	Msh2	536.502	831.973	836.2988	1105.7	827.62	0.50420269844914	0.0451517917031243	0.978863622852093
ENSRNOG00000003247	Sde2	4447.43	2907.06	2897.489	2295.55	3136.9	-0.501855111599884	0.0248901365917508	0.978863622852093
ENSRNOG00000015109	Ubxn8	1571.18	1016.21	1006.853	820.617	1103.7	-0.501172868625555	0.0356186187306271	0.978863622852093
ENSRNOG00000019048	Sod2	9382.39	6603.43	6484.95	4812.83	6820.9	-0.500667553422546	0.019823724667088	0.978863622852093

Supplementary Table S3. qRT-PCR Primer Sequences.

Gene	Forward	Reverse	Species
Gpx4	AGTTCGGGAGGCAGGAG	CCACGCAGCCGTTCTTA	Rat
ACSL4	TCCGCTTGTGACTTTAT	ACTTGGAGGAATGCTTG	Rat
β actin	GGAGATTACTGCCCTGGCTCCTAGC	GGCCGGACTCATCGTACTCCTGCTT	Rat
AIFM2	CGGGTTCGCCAAAAAGACATT	CTATGCCAATCACTTTGCCCT	Rat
FTH1	CAAGTGCGCCAGAACTACCA	ACAGATAGACGTAGGAGGCATAC	Rat
ki67	ATCATTGACCGCTCCTTTAGGT	GCTCGCCTTGATGGTTCCT	Rat
Cyclin D1	CATCAAGTGTGACCCGGACTG	CCTCCTCCTCAGTGGCCTTG	Human
HMOX1	AAGACTGCGTTCCTGCTCAAC	AAAGCCCTACAGCAACTGTCG	Human
Bnip3	CAGGGCTCCTGGGTAGAACT	CTACTCCGTCCAGACTCATGC	Human
Bach1	TCTGAGTGAGAACTCGGTTTTTG	TCTGAGTGAGAACTCGGTTTTTG	Human
ACTA2	GTGTTGCCCCTGAAGAGCAT	GCTGGGACATTGAAAGTCTCA	Human
18s rRNA	CATGTACGTTGCTATCCAGGC	CTCCTTAATGTCACGCACGAT	Human
